# Identification and validation of long non-coding RNA associated ceRNAs in intrauterine adhesion

**DOI:** 10.1080/21655979.2021.2017578

**Published:** 2021-12-30

**Authors:** Jingni Zhang, Peng Jiang, Yuan Tu, Ning Li, Yuzhen Huang, Shan Jiang, Wei Kong, Rui Yuan

**Affiliations:** Department of Gynecology, The First Affiliated Hospital of Chongqing Medical University, Chongqing, China

**Keywords:** Intrauterine adhesion, competitive endogenous RNA network, bioinformatics analysis, correlation analysis

## Abstract

Intrauterine adhesion (IUA) is an endometrial fibrotic disease with unclear pathogenesis. Increasing evidence suggested the important role of competitive endogenous RNA (ceRNA) in diseases. This study aimed to identify and verify the key long non-coding RNA (lncRNA) associated-ceRNAs in IUA. The lncRNA/mRNA expression file was obtained by transcriptome sequencing of IUA and normal samples. The microRNAs expression date was downloaded from the Gene Expression Omnibus database. Differential expressions of mRNAs, lncRNAs and miRNAs were analyzed using the DESeq2 (2010) R package. Protein interaction network was constructed to explore hub genes. TargetScan and miRanda databases were used to predicate the interaction. Enrichment analysis in Gene Ontology and Kyoto Encyclopedia of Genes and Genomes were performed to identify the biological functions of ceRNAs. Regression analysis of ceRNAs’ expression level was performed. There were 915 mRNAs and 418 lncRNAs differentially expressed. AURKA, CDC20, IL6, ASPM, CDCA8, BIRC5, UBE2C, H2AFX, RRM2 and CENPE were identified as hub genes. The ceRNAs network, including 28 lncRNAs, 28 miRNAs, and 299 mRNAs, was constructed. Regression analysis showed a good positive correlation between ceRNAs expression levels (r > 0.700, p < 0.001). The enriched functions include ion transmembrane transport, focal adhesion, cAMP signaling pathway and cGMP-PKG signaling pathway. The novel lncRNA-miRNA-mRNA network in IUA was excavated. Crucial lncRNAs such as ADIRF-AS1, LINC00632, DIO3OS, MBNL1-AS1, MIR1-1HG-AS1, AC100803.2 was involved in the development of IUA. cGMP-PKG signaling pathway and ion transport might be new directions for IUA pathogenesis research.

## 1. Introduction

Intrauterine adhesion (IUA) is an endometrial fibrotic disease caused by endometrial injury and disorders of regenerative repair. It is often manifested as hypomenorrhea, amenorrhea, and secondary infertility, and can lead to miscarriage and placental implantation [1]. At present, transcervical resection of adhesions (TCRA) is the ‘gold’ method, but the postoperative recurrence rate is as high as 60% [2]. Unfortunately, there is no ideal treatment for severe IUA.

The pathogenesis of IUA is still unclear. In recent years, microarray analysis and transcriptome sequencing have been widely used to detect differentially expressed gene in tissues or cells and further study its biological functions. In the past, scholars have used this method to analyze the differential genes and pathways in the development of IUA [3,4]. Long non-coding (lncRNA) is an endogenous RNA molecule with length greater than 200 nucleotides [5]. The lncRNA that affects the expression of mRNA by competitively binding microRNA (miRNA) is called competing endogenous RNA (ceRNA), which has been widely used to explore the development of diseases. Previous studies have indicated that some lncRNAs regulate the expression pattern and biological characteristics of microRNAs (miRNAs) to further affect the expression of mRNAs during IUA development [6,7], but its role in IUA has not been widely studied.

Multiple ceRNAs may play important and complex roles in IUA. Thus, this study performed transcriptome sequencing on the endometrium of IUA patients and normal endometrium, and then conducted a comprehensive bioinformatics analysis to construct the lncRNA-miRNA-mRNA network and excavate the crucial ceRNAs in IUA, aiming to supply a theoretical basis for deeply investigating the pathogenesis of IUA and promote the development of molecular targeted therapy for IUA.

## 2. Materials and methods

### 2.1 Sample collection and preparation

This study was conducted after being approved by the Ethics Committee of the First Affiliated Hospital of Chongqing Medical University. All participants signed consent forms. The endometrium of three severe intrauterine adhesion (American Fertility Society score >8 [8]) patients were obtained when TCRA was performed. Normal endometrium was obtained from patients who underwent a transcervical resection of the septum. The endometrial tissues all come from the early-mid menstrual cycle (Supplementary Table 1). None of the participants received hormone therapy or had a pregnancy in the past 6 months. The sample tissue was immersed in RNA stabilization solution (Beyotime, Shanghai, China) and then transferred to liquid nitrogen for storage.

### 2.2 Library preparation for mRNA/lncRNA and sequencing

TRIzol reagent (Qiagen, Hilden, Germany) was used to isolate total RNA from samples. The spectrophotometer (IMPLEN, CA, USA) was used to assess RNA purity. RNA integrity was measured using the RNA Nano 6000 Assay Kit of the Bioanalyzer 2100 system (Agilent Technologies, CA, USA).

Samples with RNA integrity number >7.0 were selected for sequencing. Firstly, 3 μg RNA per sample was used to obtain the rRNA free residue. Briefly, rRNA Removal Kit (Epicenter, USA) was used to remove ribosomal RNA from total RNA sample, then the products were purified by ethanol precipitation. Subsequently, sequencing libraries were established using rRNA free residue by Directional RNA Library Prep Kit for Illumina (NEB, USA) following manufacturer’s instructions, and libraries quality was checked on the Agilent Bioanalyzer 2100 system. Then, TruSeq PE Cluster Kit v3-cBot-HS (Illumina) was used to generate the cluster on a cBot Cluster Generation System according to the manufacturer’s instructions. Lastly, the libraries were sequenced on an Illumina Hiseq 4000 platform and 150 bp paired-end.

### 2.3 Gene expression file acquisition and differential expression analysis

Reads numbers mapped to each gene were counted using HTSeq v0.13.5. Fragments per kilobase per million of both lncRNAs and coding genes were calculated based on the length of genes and reads count, and used for differential expression analysis by the DESeq2 (2010) R package [9]. Use ‘intrauterine adhesion’ and ‘Genome-wide Expression Profiles’ as keywords to search the IUA-associated miRNAs expression dataset in the Gene Expression Omnibus (GEO) database, then GSE160634 dataset was obtained. Differential expression analysis of miRNAs in GSE160634 dataset were also performed using the DESeq2 (2010) R package. |log2(fold change) |>1 and p-value <0.05 were the thresholds for significantly differential expression by default.

### 2.4 Constructing the ceRNAs network

MiRanda database was used to screen miRNAs targeted by differentially expressed lncRNAs (DELs). The obtained miRNAs were further intersected with the differentially expressed miRNAs (DEMs) in GSE160634 for improving the accuracy. Then, TargetScan was used to predict the targeted genes of the intersecting miRNAs. Similarly, targeted genes were taken at intersection with the differentially expressed mRNA (DEGs) in sequencing file. According to the ceRNAs theory [10], this study overlapped the predicted targets of up-regulated DELs, down-regulated DEMs, and up-regulated DEGs, then up-regulated ceRNAs network was constructed. Cytoscape was used to visualize the lncRNA-miRNA-mRNA network.

### 2.5 Regression analysis of the expression level of ceRNAs

Several gene expression data of fibrotic disease were analyzed (data accessible at NCBI GEO database, GSE84044, Wang et al., 2016; GSE130955, Zhu et al., 2019; GSE148602, Guo et al., 2020). Regression analysis and correlation coefficient calculation were implemented using SPSS Statistics v25.0 software package (IBM, Armonk, NY, USA).

### 2.6 Enrichment analysis of function and pathway

Gene Ontology (GO) enrichment analysis of DEGs was performed using the clusterProfiler R package (v3.12.0), aiming to explore the enriched biological processes of DEGs. Kyoto Encyclopedia of Genes and Genomes (KEGG) enrichment analysis of DEGs was used KOBAS v3.0 software, aiming to obtain the enriched pathway of DEGs. The p-value calculated by Fisher’s exact test represented the enrichment degree. P-value <0.05 was considered significantly for both GO terms and KEGG pathways.

### 2.7 Protein–protein interaction (PPI) network generation and hub gene analysis

The protein interaction network was constructed to further explore the hub genes of IUA. We import the differential gene TOP500 into the String database, limit the research species to ‘Homo Sapiens’ to obtain the protein interaction relationship, set the connection score > 0.5, and finally export the differential gene protein interaction network data file. We use the network analyzer in Cytoscape software to visualize and construct a PPI network diagram, and use cytoHubba to get the top 10 hub gene based on degree.

## 3. Results

In this study, we hypothesized that multiple ceRNAs may play important and complex roles in IUA. First, we performed differential expression analysis and constructed ceRNA network according to interaction, and then regression analysis validated good positive correlation between lnRNA-mRNA expression levels. Enrichment analysis identified critical functions of ceRNA were transmembrane transport, cAMP signaling pathway and cGMP-PKG signaling pathway.

### 3.1 Differential expression of lncRNAs, miRNAs and mRNAs

There were 915 DEMs and 418 DELs identified. Among them, 353 mRNAs and 249 lncRNAs were overexpressed, while 562 mRNAs and 169 lncRNAs were under-expressed (Supplementary Table 2). In GSE160634 dataset, 183 miRNAs were differentially expressed, in which 14 miRNAs were over-expressed and 169 miRNAs were under-expressed.

### 3.2 GO and KEGG pathway enrichment analysis of the DEGs

The GO analysis of 915 DEGs identified that many genes were significantly enriched in the DNA packaging (GO:0006323 p = 1.11E-10), extracellular matrix organization (GO:0030198 p = 3.46E-08), acute inflammatory response (GO:0002526 p = 8.96E-07), positive regulation of leukocyte cell–cell adhesion (GO:1903039 p = 2.89E-05), muscle system process (GO:0003012 p = 1.25E-07), and regulation of T cell activation (GO:0050863 p = 2.17E-06) (Table 1). KEGG pathway enrichment analysis identified several DEGs-associated signaling pathways, including the systemic lupus erythematosus (p = 6.09192E-20), NOD-like receptor signaling pathway (p = 2.26949E-09), cytokine–cytokine receptor interaction (p = 9.2017E-09), NF-kappa B signaling pathway (p = 9.02412E-08), TNF signaling pathway (p = 1.18615E-07), necroptosis (p = 1.383E-06), toll-like receptor signaling pathway (p = 3.27057E-06) (Table 2).

**Table 1.** Top 15 terms of Gene Ontology analysis of the 915 differentially expressed genes

Term	Description	Count	*p* Value
GO:0006323	DNA packaging	36	1.11E-10
GO:0034401	chromatin organization involved in regulation of transcription	25	4.08305E-09
GO:0045814	negative regulation of gene expression, epigenetic	22	2.58071E-08
GO:0030198	extracellular matrix organization	44	3.47E-08
GO:0003012	muscle system process	46	1.25E-07
GO:0071824	protein-DNA complex subunit organization	34	2.5606E-07
GO:0002526	acute inflammatory response	19	8.97E-07
GO:0050863	regulation of T cell activation	39	2.17E-06
GO:0040029	regulation of gene expression, epigenetic	26	3.25783E-06
GO:0010959	regulation of metal ion transport	39	3.38777E-06
GO:0009311	oligosaccharide metabolic process	12	6.77125E-06
GO:1903039	positive regulation of leukocyte cell-cell adhesion	29	2.88932E-05
GO:0062197	cellular response to chemical stress	34	3.01087E-05
GO:0007249	I-kappaB kinase/NF-kappaB signaling	30	3.03254E-05
GO:0007080	mitotic metaphase plate congression	10	4.30341E-05

**Table 2.** Top 15 terms of Kyoto Encyclopedia of Genes and Genomes pathway analysis of the 915 differentially expressed genes

Term	Description	Input number	*p−*Value
hsa05322	Systemic lupus erythematosus	37	6.09192E-20
hsa05169	Epstein-Barr virus infection	32	1.94502E-11
hsa04621	NOD-like receptor signaling pathway	27	2.26949E-09
hsa04060	Cytokine-cytokine receptor interaction	34	9.2017E-09
hsa05200	Pathways in cancer	48	1.53534E-08
hsa05202	Transcriptional misregulation in cancer	26	1.65094E-08
hsa05167	Kaposi sarcoma-associated herpesvirus infection	25	7.4316E-08
hsa04064	NF-kappa B signaling pathway	18	9.02412E-08
hsa04668	TNF signaling pathway	19	1.18615E-07
hsa04217	Necroptosis	21	1.383E-06
hsa05161	Hepatitis B	21	1.5109E-06
hsa05323	Rheumatoid arthritis	15	1.89847E-06
hsa05166	Human T-cell leukemia virus 1 infection	24	2.92875E-06
hsa04620	Toll-like receptor signaling pathway	16	3.27057E-06
hsa05152	Tuberculosis	21	3.82689E-06

### 3.3 PPI network of the DEGs

**Figure 1.**
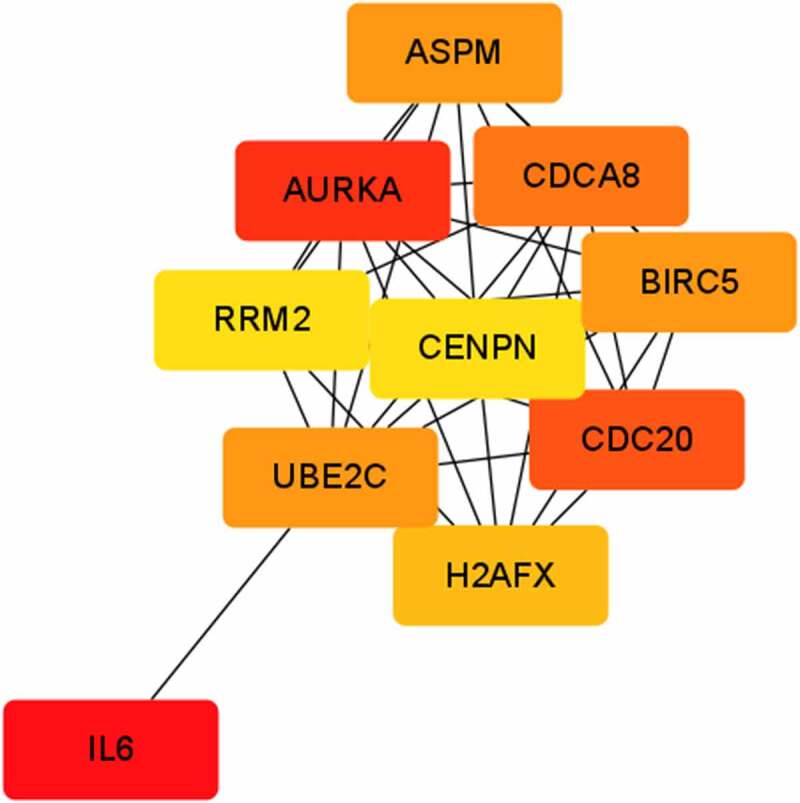
The protein–protein interaction network of differentially expressed genes.

The PPI network was established using STRING and Cytoscape consisting of 420 nodes and 1802 edges. The top 10 genes according to degree in cytoHubba were AURKA, CDC20, IL6, ASPM, CDCA8, BIRC5, UBE2C, H2AFX, RRM2 and CENPE, which were identified as hub genes (Figure 1).

### 3.4 Analysis of the ceRNAs network

**Figure 2.**
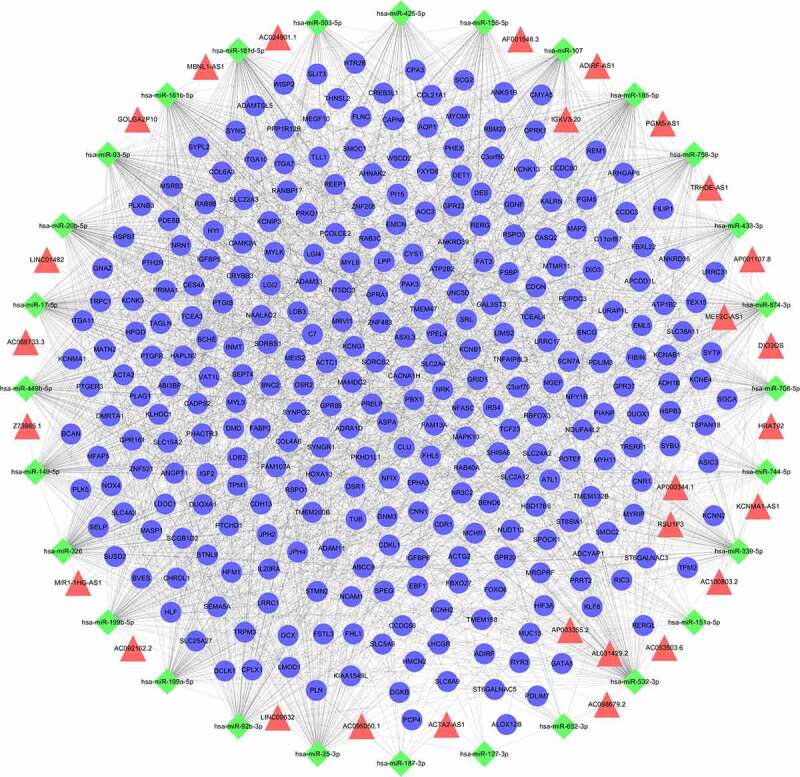
The ceRNAs network in IUA. Diamond-shaped nodes represent miRNAs; Triangle nodes represent lncRNAs; Circular nodes represent mRNAs; Every edge indicates target interaction.

The final integrated lncRNA-miRNA-mRNA interaction film (Supplementary Table 3) was imported into Cytoscape, then ceRNAs network was constructed (Figure 2). It included 355 nodes (28 lncRNAs, 28 miRNAs, and 299 mRNAs) and 2315 edges. Each edge represents the relationship between lncRNA and miRNA, miRNA and mRNA. The top 5 lncRNAs in ceRNAs network according to the degree were ADIRF-AS1, LINC00632, DIO3OS, MIR1-1HG-AS1, AC100803.2, and the top 5 miRNAs were miR-326, miR-185-5p, miR-532-3p, miR-93-5p, miR-149-5p.

**Figure 3.**
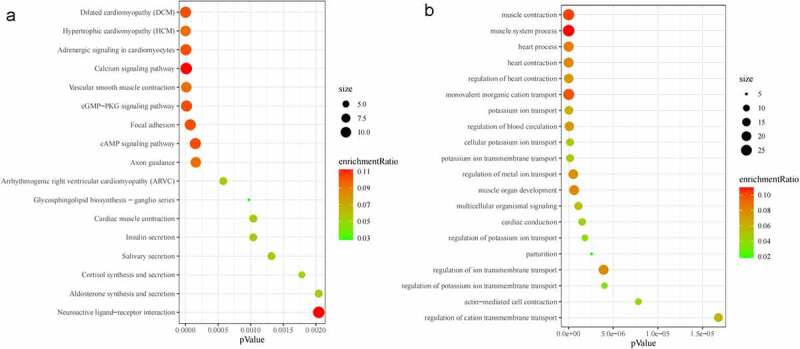
Kyoto Encyclopedia of Genes and Genomes (KEGG) pathway and Gene Ontology (GO) term enrichment analysis of differentially expressed genes (DEGs). (a) All enriched KEGG pathways of DEGs in ceRNAs network. (b) TOP20 enriched GO biological processes of DEGs in ceRNAs network.

GO and KEGG enrichment analyses were performed. The significantly enriched biological processes included regulation of blood circulation, muscle organ development, multicellular organismal signaling, regulation of ion transmembrane transport, and actin-mediated cell contraction. The significantly enriched KEGG pathways included hypertrophic cardiomyopathy (HCM), vascular smooth muscle contraction, cGMP-PKG signaling pathway, focal adhesion, and cAMP signaling pathway. Figure 3 lists the top 20 statistically enriched GO terms and all statistically enriched KEGG pathway (p < 0.05).

### 3.5 Positive correlation between the expression levels of ceRNAs

**Figure 4.**
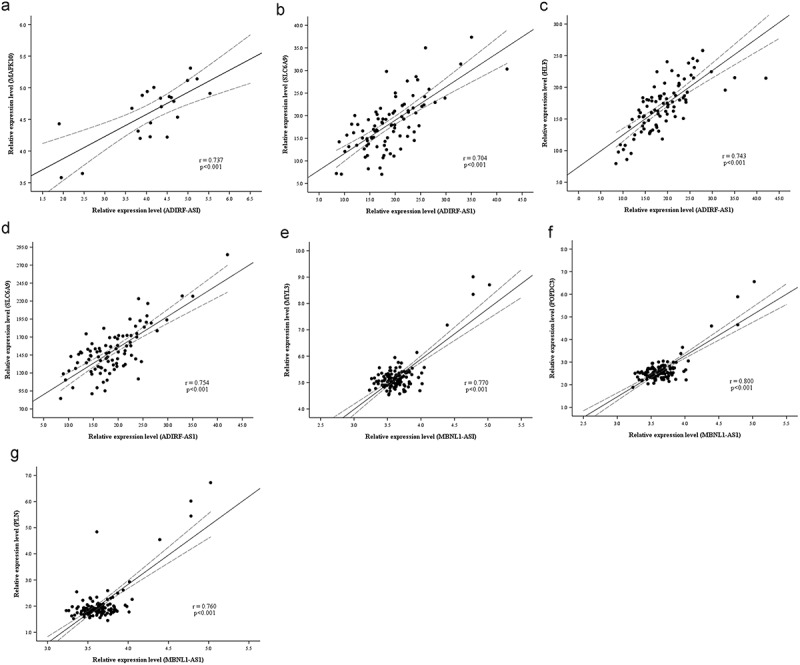
Linear regression of ceRNAs’ expression level. Dashed lines represent 95% confidence interval. (a) ADIRF-AS1 vs MAPK10 (hypertrophic cardiomyopathy, n = 22). (b) ADIRF-AS1 vs SLC6A9 (systemic sclerosis, n = 91). (c) ADIRF-AS1 vs HLF (systemic sclerosis, n = 91). (d) ADIRF-AS1 vs AHNAK2 (systemic sclerosis, n = 91). (e) MBNL1-AS1 vs MYL3 (liver fibrosis, n = 124). (E) MBNL1-AS1 vs POPDC3 (liver fibrosis, n = 124). (f) MBNL1-AS1 vs PLN (liver fibrosis, n = 124).

The ceRNAs network constructed in the present study revealed the interaction of lncRNAs and mRNA in IUA. To confirm this finding, regression analysis was performed using several gene expression datasets of fibrotic disease including liver fibrosis, heart fibrosis, and skin fibrosis. The results showed a very good positive correlation between lnRNA-mRNA expression levels (Figure 4). For example, ADIRF-AS1 interacted with MAPK10 mediated by miRNA-339-5p (r = 0.737 p < 0.001) (Figure 4(a)), interacted with SLC6A9 (r = 0.704 p < 0.001) and HLF (r = 0.743 p < 0.001) mediated by miRNA-874-3p (Figure 4(b,c)), interacted with AHNAK2 mediated by miRNA-326 (r = 0.754 p < 0.001) (Figure 4(d)); MBNL1-AS1 interacted with MYL3 mediated by miRNA-503-5p (r = 0.770 p < 0.001) (Figure 4(e)), interacted with POPDC3 mediated by miRNA-149-5p (r = 0.800 p < 0.001) (Figure 4(f)), interacted with PLN mediated by miRNA-149-5p (r = 0.760 p < 0.001) (Figure 4(g)).

## 4. Discussion

Intrauterine adhesion is a fibrotic endometrial disease that seriously threatens women’s reproductive health, and the pathogenesis is still unclear. Recent microarray analysis has identified some lncRNAs, miRNAs, and mRNAs associated with IUA. Previous study confirmed that miRNAs mediating the interactions of lncRNAs and mRNAs were important to the development of many diseases. Thus, deeply exploring lncRNA-miRNA-mRNA ceRNAs network will improve the understanding of the occurrence and development of IUA.

This study identified a total of 915 DEGs. Among the DEGs, AURKA, CDC20, IL6, ASPM, CDCA8, BIRC5, UBE2C, H2AFX, RRM2 and CENPE were identified as hub genes. IL6 is a recognized inflammatory factor associated to fibrosis [11], and its relationship with intrauterine adhesions has been confirmed [12]. CDC20 inhibition was confirmed to suppress the expression of profibrotic markers in radiation-induced fibrosis [13], and CENPE [14] was a reactive oxygen species associated gene involved in radiation-induced fibrosis. ASPM is highly expressed in liver cirrhosis [15]. AURKA inhibition was sufficient to normalize megakaryocytes and reduce bone marrow fibrosis [16]. H2AFX [17] was also a fibrosis-associated signature in marrow fibrosis.

LncRNAs were involved in various biological functions of fibrotic diseases [18–21]. This study identified 249 up-regulated and 169 down-regulated DELs. Although the relationship between most DELs and IUA has not yet been demonstrated, many of them were confirmed to be associated with other fibrotic diseases. For example, OTUD6B-AS1 was down-regulated in the skin tissue of patients with systemic sclerosis [22], which manifests as fibrosis of skin and multiple organs. MEG8 was confirmed to suppress hepatic stellate cells activation and epithelial–mesenchymal transition (EMT) of hepatocytes in liver fibrosis via the Notch pathway [23]. ADAMTS9-AS2 inhibited AKT signaling pathway to suppress progression of oral submucous fibrosis [24]. IL7-AC083837.1 gene fusion might contribute to the development of fibrosis of IPF patients [25]. And many of them participated in fibrosis-associated biological processes, such as EMT, fibroblast proliferation, stem cell differentiation and so on. For example, MBNL1-AS1 was upregulated upon vascular smooth muscle cells differentiation [26]. PGM5-AS1 promotes EMT of osteosarcoma cells [27]. TRHDE-AS1 inhibits the scar fibroblasts proliferation [28]. DIO3OS was associated with the reproduction and development of mouse endometrial stromal cells [29]. All of the above indicates the crucial role of DELs in fibrosis diseases, including IUA. Therefore, the lncRNAs identified in this study might be new directions for exploring the pathogenesis of IUA or serve as potential biomarkers of IUA therapy.

The ceRNAs network of lncRNA–miRNA–mRNA was established. Among the miRNAs in ceRNAs network, miRNA-326 could target block the 3ʹUTR of transforming growth factor-beta1 (TGF-β1) RNA, further suppressing the activation of the TGF-β1/Smads signaling pathway in endometrial stromal cells from patients with IUA [30]. The miRNA-155-5p was found to be implicated in fibrosis of chronic kidney disease patients [31]. Nayan J Sarma found miRNA-107 was participated in HCV-induced liver fibrosis [32]. In addition, miRNA-455-5p and miRNA-223-3p were confirmed to attenuate endometrial injury and promote repair of damaged endometrium in IUA [33,34]. Collectively, ceRNAs network mainly controlled critical functions such as transmembrane transport, cAMP signaling pathway and cGMP-PKG signaling pathway. Among the identified pathways, cGMP-PKG signaling pathway was known as a fibrosis-associated pathway because it was involved in the development of various organ fibrosis, such as pulmonary, cardiac, and kidney [35–37], but its role in the development of IUA has not been completely recognized. Of the screened biological processes, ion transport was found to be implicated in IUA. However, the research about ion transport mainly focuses on cystic fibrosis [38,39], and its mechanism in IUA is still worth further investigation.

Although this study provides important molecular information about the occurrence and development of IUA, there are some limitations. First of all, the number of samples used for transcriptome sequencing is relatively small. Second, this study only verified the correlation of ceRNA in the GEO data set, luciferase reporter assays and co-immunoprecipitation analysis are still needed for further verification. In the future, we will conduct deep mechanism studies on the critical lncRNAs in the ceRNA network, especially lncRNAs associated with ion transport, aiming to provide new direction for the pathogenesis research of IUA.

## 5. Conclusion

This study established a novel lncRNA-miRNA-mRNA network in IUA based on the ceRNAs mechanism. Some potential genes such as MYL3, MAPK10, POPDC3, PLN, HLF, SLC6A9, lncRNAs such as ADIRF-AS1, LINC00632, DIO3OS, MBNL1-AS1, and miRNAs such as miRNA-326, miRNA-155-5p, miRNA-874-3p, miRNA-503-5p, miRNA-149-5p that might be involved in the occurrence and development of IUA were excavated. cGMP-PKG signaling pathway and ion transport might be new directions for exploring the pathogenesis of IUA.

## Supplementary Material

Supplemental Material

## Data Availability

Part of the data described in this manuscript can be obtained from public databases, and others are available from the corresponding author upon reasonable request.

## Author information

Jingni Zhang and Peng Jiang contributed equally to the study. Correspondence to Rui Yuan.
