# Supplementary material for: Identification and validation of long non-coding RNA associated ceRNAs in intrauterine adhesion
Source: Bioengineered. 2021 Dec 30;13(1):1039–48. doi: 10.1080/21655979.2021.2017578 (PMC8805920; doi:10.1080/21655979.2021.2017578)
Supplement: Supplemental Material [file KBIE_A_2017578_SM8799.zip › supplementary/Supplementary Table 1.docx]

**Supplementary Table 1** Information about the sample of patients with IUA and controls analyzed in our study

| Group | Age (years) | Sample date (from LMP) | History of gestation | Score of IUA |
| --- | --- | --- | --- | --- |
| IUA1 | 33 | 12 | G3P1 | 12 |
| IUA2 | 35 | 10 | G3P2 | 10 |
| IUA3 | 34 | 10 | G6P1 | 9 |
| Control1 | 31 | 12 | G0P0 | - |
| Control2 | 30 | 13 | G2P0 | - |
| Control3 | 35 | 10 | G1P0 | - |

LMP, last menstrual period; IUA, intrauterine adhesion; history of gestation, G for gestation, P for parturition.
