# Supplementary material for: Identification and validation of long non-coding RNA associated ceRNAs in intrauterine adhesion
Source: Bioengineered. 2021 Dec 30;13(1):1039–48. doi: 10.1080/21655979.2021.2017578 (PMC8805920; doi:10.1080/21655979.2021.2017578)
Supplement: Supplemental Material [file KBIE_A_2017578_SM8799.zip › supplementary/Supplementary Table 2.docx]

**Supplementary Table 2: The differentially expressed mRNAs and lncRNAs in the IUA tissue**

| **Gene name** | **log2FoldChange** | **p-value** |
| --- | --- | --- |
| DES | 6.566408577 | 1.32234E-11 |
| ACTG2 | 7.884647078 | 7.74353E-11 |
| PLN | 5.722565082 | 1.00225E-10 |
| MEGF10 | 3.25427422 | 1.34521E-10 |
| CDR1 | 3.63389061 | 4.09639E-10 |
| RSPO3 | 2.349129956 | 1.58737E-09 |
| HSPB7 | 5.547106692 | 6.69188E-09 |
| JPH2 | 5.498630586 | 1.8611E-08 |
| MYH11 | 6.213213257 | 8.51649E-08 |
| ZNRF2 | -1.030450854 | 8.88039E-08 |
| RERG | 3.643207795 | 1.92881E-07 |
| SLC6A8 | -1.802763147 | 3.66396E-07 |
| CAMK2A | 3.509932122 | 5.87098E-07 |
| TRIM31 | -4.313673229 | 7.79796E-07 |
| PRELP | 6.790279088 | 1.05106E-06 |
| FAT3 | 4.654054762 | 1.77761E-06 |
| SYNC | 2.004626796 | 1.82247E-06 |
| PTTG1 | -2.462479543 | 1.87319E-06 |
| CHRDL2 | 6.432956878 | 3.50695E-06 |
| DEFB4B | -7.422614802 | 4.18259E-06 |
| MAPK10 | 1.549120572 | 5.26894E-06 |
| ENOX1 | 2.64608059 | 5.38697E-06 |
| PTGFR | 4.292007409 | 7.14914E-06 |
| ACTA2 | 3.344595103 | 7.3617E-06 |
| MTMR11 | 1.11856159 | 7.9121E-06 |
| ST14 | -1.071170709 | 8.38069E-06 |
| NEXN | 2.247710653 | 9.8718E-06 |
| KCNH2 | 3.649192691 | 1.26917E-05 |
| FHL5 | 3.569070896 | 1.87361E-05 |
| SLC24A2 | 8.436936916 | 2.65484E-05 |
| UNC5D | 5.053312765 | 2.92404E-05 |
| TEX15 | 4.893291049 | 2.96585E-05 |
| SCN7A | 4.22784082 | 4.24539E-05 |
| MSRB3 | 2.231546341 | 4.31457E-05 |
| PKHD1L1 | 4.114644115 | 4.33306E-05 |
| TPM2 | 2.157109832 | 4.35614E-05 |
| SGCA | 3.78315894 | 4.87163E-05 |
| NFASC | 3.487286859 | 4.97537E-05 |
| SERTM2 | 4.74049141 | 5.05205E-05 |
| DNM3 | 1.177564485 | 5.29347E-05 |
| DMD | 3.152572566 | 5.36308E-05 |
| CHRDL1 | 5.277106663 | 6.17638E-05 |
| MAMDC2 | 3.303258106 | 6.2848E-05 |
| TNFRSF10C | -5.423652595 | 6.78873E-05 |
| ZNF676 | 2.242414277 | 7.74034E-05 |
| SPEG | 2.914080851 | 8.03106E-05 |
| CA2 | -3.443664654 | 9.07665E-05 |
| TCF23 | 5.496489287 | 0.000100686 |
| HTR7 | -2.487847094 | 0.000118757 |
| KPNA2 | -1.315614066 | 0.000119378 |
| RNF157 | -1.044563104 | 0.000119689 |
| HIF3A | 5.356083449 | 0.000120165 |
| NCAPH | -2.400057363 | 0.000135093 |
| ZMIZ2 | -1.011418698 | 0.000137391 |
| MYLK | 2.88475194 | 0.00014102 |
| KALRN | 1.302729005 | 0.00014159 |
| HIST3H2BB | -1.660283359 | 0.000150998 |
| ADCYAP1 | 4.491285298 | 0.000172833 |
| HIST1H3H | -2.111482925 | 0.000190603 |
| CXCR2 | -6.3708916 | 0.000192223 |
| TCEAL2 | 6.458852246 | 0.000197422 |
| GPR22 | 4.115895039 | 0.000212255 |
| ADH1B | 5.06914419 | 0.00022597 |
| LRG1 | -2.845473034 | 0.000228226 |
| PDLIM3 | 3.196689473 | 0.000237735 |
| BCHE | 3.749618407 | 0.000253848 |
| LFNG | -2.058659186 | 0.000261831 |
| CAPN6 | 4.670322673 | 0.000264121 |
| HIST1H2AJ | -2.692916164 | 0.000267771 |
| RBFOX3 | 6.27218834 | 0.00027916 |
| OPRK1 | 5.19307524 | 0.000300426 |
| PGM5 | 3.747232467 | 0.00030443 |
| HPSE2 | -1.465063029 | 0.000309796 |
| SPOCK1 | 2.342434285 | 0.000327572 |
| CXCR1 | -6.16204027 | 0.00032866 |
| TLL1 | 2.596538641 | 0.000367857 |
| CCDC102B | 1.069470021 | 0.00037055 |
| AFF2 | -2.046931339 | 0.000378139 |
| GDA | -1.719185996 | 0.00040484 |
| FGB | -7.308673756 | 0.000408134 |
| TNFAIP8L3 | 2.426926378 | 0.000410076 |
| CDC20 | -2.873090911 | 0.000412445 |
| S1PR5 | -4.322288629 | 0.000419509 |
| ABI3BP | 2.119631885 | 0.000443723 |
| PADI4 | -6.452866958 | 0.000476423 |
| FAM83B | -1.544758971 | 0.000481268 |
| WSCD2 | 3.899424062 | 0.000511036 |
| LGI4 | 3.856364718 | 0.00053631 |
| KCNG1 | 3.613196175 | 0.000562173 |
| MYL9 | 2.237032161 | 0.00059988 |
| FCGR3B | -6.302126189 | 0.000619468 |
| BNC2 | 2.226128382 | 0.000636682 |
| LHCGR | 6.533630159 | 0.000639356 |
| PI15 | 5.233364995 | 0.000640004 |
| SCGB1D2 | 5.530683349 | 0.000650101 |
| BTNL9 | 3.682665096 | 0.000650349 |
| ELOVL3 | -5.729118543 | 0.000652229 |
| NPY1R | 3.346071219 | 0.000674315 |
| MUC15 | 4.336205917 | 0.000706464 |
| PDE8B | 2.533369337 | 0.000731801 |
| HIGD1B | 2.680194897 | 0.000734312 |
| LIMS2 | 2.480864973 | 0.000740638 |
| NRK | 3.348036881 | 0.000756986 |
| ST8SIA1 | 2.020858302 | 0.000831494 |
| C8orf88 | 1.444411148 | 0.000834735 |
| SYT9 | 4.09870711 | 0.00083839 |
| HSPB3 | 5.899108678 | 0.000875629 |
| ANGPT1 | 2.533343927 | 0.000879031 |
| PPP1R12B | 1.838582124 | 0.000887122 |
| PBX1 | 1.460443566 | 0.000891833 |
| TACC3 | -1.74311572 | 0.000900945 |
| MGAM | -5.036386309 | 0.000919308 |
| C2orf40 | 3.784159727 | 0.000930434 |
| E2F2 | -2.41634181 | 0.000941577 |
| FBXO27 | 1.01419446 | 0.000978629 |
| GAL3ST3 | 6.236750157 | 0.001013827 |
| TCEAL4 | 1.609013105 | 0.001020336 |
| EPOP | -1.976224302 | 0.00103599 |
| CENPN | -1.5850373 | 0.001181894 |
| MRVI1 | 2.210492073 | 0.001200868 |
| GCH1 | -4.343735641 | 0.001253131 |
| TENT5B | 3.148902739 | 0.001272476 |
| OVOL1 | -2.964359553 | 0.001289703 |
| CTSG | 3.457456355 | 0.00130812 |
| NFAM1 | -4.039881051 | 0.00133116 |
| ERCC6L | -2.394993434 | 0.001336246 |
| SHISAL1 | 2.08113713 | 0.001366721 |
| SCG2 | 3.907496026 | 0.001393664 |
| SPAG4 | -1.703876911 | 0.00139756 |
| CCDC80 | 2.44519468 | 0.00139978 |
| SLC4A4 | -1.770108335 | 0.001472409 |
| SAA2 | -6.261342057 | 0.001475999 |
| PADI2 | -4.421562182 | 0.001527441 |
| SEPT4 | 1.71411107 | 0.001537044 |
| SV2C | -3.105933858 | 0.00153942 |
| FBXL22 | 2.806213519 | 0.001554317 |
| RAB40A | 1.709864113 | 0.001559373 |
| HAPLN2 | 5.369711976 | 0.001578021 |
| PAK3 | 2.056407878 | 0.00161326 |
| UBE2S | -1.488210625 | 0.001636113 |
| FILIP1 | 2.037511544 | 0.001638154 |
| MFAP5 | 3.480030401 | 0.001695059 |
| CLU | 1.276231577 | 0.00178935 |
| LDB3 | 3.441988608 | 0.001809163 |
| KCNIP3 | 1.044275732 | 0.001813702 |
| DCX | 5.312312964 | 0.001834453 |
| F13A1 | -2.990100817 | 0.001835181 |
| MMP25 | -4.158678773 | 0.001876336 |
| HIST1H2AL | -1.851772446 | 0.00190854 |
| ADAM33 | 1.078729266 | 0.002029592 |
| ICAM1 | -4.608728376 | 0.002033895 |
| CCDC7 | 1.218573669 | 0.002037028 |
| SERPINB3 | -6.860628693 | 0.00210102 |
| MEIS2 | 2.455809834 | 0.002155273 |
| PHEX | 1.18446841 | 0.002190804 |
| CCDC3 | 1.859927349 | 0.002199126 |
| PAGE4 | 2.086986437 | 0.002238578 |
| MCHR1 | 4.351604821 | 0.002262202 |
| CLC | -6.639352263 | 0.002327252 |
| H3F3A | -1.025693088 | 0.002328349 |
| HIST1H2BJ | -1.515891496 | 0.002342933 |
| RPS2 | -1.01339258 | 0.002362337 |
| BTNL8 | -6.186901757 | 0.002366728 |
| KCNB1 | 3.94724193 | 0.002416295 |
| ADGRE3 | -5.096959511 | 0.002464359 |
| SORBS1 | 2.032256931 | 0.002486442 |
| PRR7 | -1.411014441 | 0.002517824 |
| CCNE1 | -1.582853762 | 0.002522168 |
| REEP1 | 3.129752861 | 0.002551603 |
| ADRA1D | 2.83651609 | 0.002552295 |
| SLC38A11 | 2.340386537 | 0.002575062 |
| LRRC61 | -1.019423178 | 0.002577332 |
| HIST1H2BI | -1.862759085 | 0.002624229 |
| TCEAL3 | 1.417101796 | 0.002636432 |
| H2AFX | -1.321089716 | 0.002636528 |
| PRR5L | -1.746783927 | 0.002679833 |
| RBP4 | -5.593511883 | 0.002730414 |
| ITGA10 | 1.274487068 | 0.002737321 |
| RAB3C | 4.331743023 | 0.002767107 |
| REM1 | 2.827586277 | 0.002785604 |
| KCNMA1 | 2.877622299 | 0.002942213 |
| HIST1H2BM | -1.722589509 | 0.002970689 |
| PFKFB3 | -3.075428138 | 0.003006954 |
| BAK1 | -1.302190081 | 0.003037888 |
| HIST1H3F | -2.234643382 | 0.003051718 |
| TAGLN | 2.492137453 | 0.003098872 |
| CTSS | -3.935207973 | 0.003138357 |
| IFFO2 | -1.285533792 | 0.00317406 |
| ITGB1BP2 | 1.199623973 | 0.003175235 |
| SYNPO2 | 1.418736615 | 0.003180013 |
| GBP1 | -3.755384015 | 0.003187877 |
| INMT | 2.614028472 | 0.003198347 |
| CAVIN3 | 1.271783234 | 0.003270492 |
| TNFSF14 | -2.431721299 | 0.003308243 |
| FCN1 | -4.183163686 | 0.003330982 |
| NINJ1 | -3.839234226 | 0.003449674 |
| ANKRD35 | 2.557628176 | 0.003467762 |
| TIFA | -1.874509321 | 0.003471305 |
| HMOX1 | -4.313841614 | 0.003471835 |
| TNFRSF1B | -2.743297902 | 0.003474753 |
| LMOD1 | 2.518634455 | 0.003481139 |
| TNFSF15 | -3.418489893 | 0.003516384 |
| ENPP3 | 3.54054812 | 0.003571781 |
| HIST3H2A | -1.123338943 | 0.003600343 |
| ELOVL7 | -3.266244803 | 0.003615805 |
| LYN | -3.41337097 | 0.003630016 |
| MYOM1 | 2.493747416 | 0.003637956 |
| LRRC25 | -3.592079824 | 0.003689059 |
| CNN1 | 3.625184014 | 0.003695753 |
| TRIM25 | -1.576608126 | 0.003749382 |
| E2F3 | -1.528026903 | 0.003755827 |
| AHNAK2 | 2.851139641 | 0.003768108 |
| APOBEC3A | -5.732514839 | 0.003797466 |
| NGEF | 1.344658882 | 0.003850493 |
| GPR20 | 3.812972521 | 0.003866815 |
| HIST1H2BO | -2.46941446 | 0.003869707 |
| ERP27 | -1.815290395 | 0.003898722 |
| GBP4 | -3.504612735 | 0.003940242 |
| TMEM71 | -4.21466643 | 0.003955474 |
| GNA15 | -4.034263923 | 0.003969001 |
| SCGB1D4 | 5.886274279 | 0.003978046 |
| LDOC1 | 1.592880251 | 0.004063904 |
| EPB42 | -6.595843769 | 0.004148361 |
| ITGA11 | 2.764326837 | 0.004180491 |
| CDH13 | 2.118817674 | 0.004220462 |
| WNT4 | -1.202309546 | 0.004243544 |
| REC8 | -1.203599401 | 0.004334911 |
| CCL20 | -5.338628528 | 0.004343764 |
| ICAM5 | -4.968279253 | 0.004377747 |
| WISP2 | 2.511415326 | 0.004382018 |
| RIPOR3 | 1.110063814 | 0.004465156 |
| PRKG1 | 1.849051755 | 0.004477972 |
| CDON | 2.021685779 | 0.004507026 |
| HIST1H4K | -2.7490902 | 0.004561084 |
| PTGIS | 2.749096137 | 0.004584014 |
| SLC43A2 | -3.299943434 | 0.004706602 |
| MNDA | -3.964387792 | 0.004719429 |
| PLAG1 | 1.295709629 | 0.004738417 |
| ORM1 | -3.484091546 | 0.004750084 |
| PCP4 | 3.435313269 | 0.004799506 |
| KCNK5 | -1.638929184 | 0.004843483 |
| ST6GALNAC3 | 1.213305607 | 0.004860245 |
| FAM83D | -2.251411368 | 0.004979728 |
| TRPC1 | 1.134742294 | 0.005066303 |
| LYZ | -4.133379038 | 0.005072272 |
| TMEM47 | 1.860160208 | 0.005164763 |
| HIST1H4A | -2.039924992 | 0.005173403 |
| TNFAIP6 | -5.03480139 | 0.005301135 |
| PDLIM1 | -1.26682483 | 0.005317831 |
| LPP | 1.506271357 | 0.00536678 |
| DMRTA1 | 1.866273885 | 0.005379228 |
| TNFAIP2 | -3.018733993 | 0.005406388 |
| HIST1H2AI | -1.872068959 | 0.005437237 |
| TUBG2 | 1.046122837 | 0.005564633 |
| RND1 | -4.259943884 | 0.0056093 |
| DGKB | 3.279844997 | 0.005620049 |
| KIR3DL2 | -4.062775476 | 0.00563377 |
| RERGL | 3.113672644 | 0.005688822 |
| DUOX1 | 3.76776746 | 0.005733931 |
| AOC3 | 2.716395011 | 0.005792088 |
| HIST1H3B | -2.218839729 | 0.00586547 |
| PHACTR3 | 3.652998968 | 0.005868061 |
| TK1 | -1.792153607 | 0.005938371 |
| CYP27B1 | -3.76071785 | 0.006001238 |
| DUOXA1 | 3.544088245 | 0.00602504 |
| HIST1H1B | -2.125176318 | 0.006025372 |
| SERPINA3 | -2.971787975 | 0.006073169 |
| NFIX | 1.413801799 | 0.006102362 |
| PRIMA1 | 3.144241992 | 0.006108942 |
| MYBL2 | -2.818921417 | 0.006152518 |
| HIST1H3C | -2.22709391 | 0.006181809 |
| COL9A2 | -1.643727494 | 0.006339625 |
| SLC7A11 | -3.217498201 | 0.006488227 |
| KIF18A | -1.772894701 | 0.006489183 |
| IRF1 | -3.343301854 | 0.006557925 |
| FPR3 | -3.72967803 | 0.006606502 |
| ADAP1 | -2.355754401 | 0.006665512 |
| AUNIP | -2.497180303 | 0.006671282 |
| COL26A1 | -3.379916391 | 0.006692206 |
| SLC4A3 | 1.730921531 | 0.006718208 |
| CPLX1 | 1.217138828 | 0.006763564 |
| UBE2C | -2.458248574 | 0.006771045 |
| SLC16A10 | -4.412245667 | 0.006832179 |
| HIST1H2AG | -1.792397226 | 0.00700674 |
| ETV7 | -4.123907482 | 0.007053958 |
| SMOC2 | 2.691119097 | 0.007106174 |
| TNFRSF4 | -2.733211301 | 0.007106851 |
| PHACTR1 | -2.892171032 | 0.00711106 |
| NFE2L3 | -2.737701541 | 0.007185029 |
| IRS4 | 3.786126379 | 0.007274377 |
| CREB3L1 | 1.886718765 | 0.007658441 |
| TMEM132B | 1.990512996 | 0.007721822 |
| ATP2B2 | 3.120683098 | 0.007725333 |
| DBX2 | -3.499860462 | 0.00773118 |
| SLC16A6 | -2.696436433 | 0.007733066 |
| BCAN | 3.412722687 | 0.007754878 |
| PRR11 | -1.432102097 | 0.007781292 |
| SUGCT | 1.28660871 | 0.007791162 |
| KIFC1 | -2.015210165 | 0.007862483 |
| GLA | -1.938276594 | 0.008015649 |
| HIST1H2BL | -1.811347312 | 0.008145139 |
| PWP2 | -2.712319183 | 0.00816763 |
| CNTFR | -1.891726713 | 0.008264547 |
| C7 | 2.848403385 | 0.008287357 |
| DCLK1 | 2.624935598 | 0.00838324 |
| EPHA3 | 2.459739884 | 0.008403319 |
| LMNB1 | -2.174877591 | 0.008464643 |
| SLC7A3 | 3.41823739 | 0.008501758 |
| PDE4B | -4.186457375 | 0.008632065 |
| FAM107A | 1.586000271 | 0.00864123 |
| IGF2 | 1.942930929 | 0.008773002 |
| RIC3 | 2.17329998 | 0.008874425 |
| PLPP7 | 1.356489113 | 0.00910656 |
| IFI6 | -2.11166367 | 0.009114138 |
| ADIRF | 2.28871036 | 0.009241719 |
| MMP12 | -5.438101481 | 0.009259974 |
| BBC3 | -1.669263334 | 0.009276451 |
| SLC2A3 | -4.101210692 | 0.009291763 |
| NCAM1 | 2.714011147 | 0.009531172 |
| HAPLN3 | -2.299909082 | 0.009549808 |
| CD83 | -3.534443285 | 0.009571495 |
| IL17C | -6.831889434 | 0.009588362 |
| WNT16 | -3.359738102 | 0.009677874 |
| MT1A | -2.889827464 | 0.009684437 |
| LTK | -2.896232235 | 0.009687831 |
| IRAK2 | -3.788763175 | 0.009718308 |
| ID2 | -1.27860072 | 0.009731234 |
| PDLIM7 | 1.007131351 | 0.009747115 |
| SDS | -3.7684337 | 0.009748106 |
| GATA5 | 3.530920473 | 0.009751628 |
| CMTM2 | -3.507481154 | 0.009846892 |
| TGFBI | -2.34320787 | 0.009870563 |
| RELT | -3.130923519 | 0.00996001 |
| FAM129A | -3.205712485 | 0.009964564 |
| KCNJ2 | -3.033745112 | 0.010020874 |
| SLC25A37 | -2.403698532 | 0.010155913 |
| SNCG | 1.24115143 | 0.010183572 |
| SIPA1L1 | -1.542441572 | 0.010242089 |
| HIST1H2AH | -1.769933251 | 0.010263265 |
| TMEM158 | 1.153635606 | 0.010272638 |
| CST1 | 2.979334548 | 0.010283429 |
| NCMAP | -1.762871358 | 0.010321913 |
| ARHGAP6 | 1.985524445 | 0.010484247 |
| SLC2A1 | -1.471858323 | 0.010489484 |
| TRAF3 | -1.12518678 | 0.010526963 |
| CENPE | -1.946562987 | 0.010567576 |
| CYS1 | 1.247420951 | 0.010594211 |
| HMCN2 | 3.463911954 | 0.010607433 |
| ATF5 | -2.198638181 | 0.010733014 |
| BID | -2.305061163 | 0.010914909 |
| P2RY8 | -3.398770153 | 0.010931636 |
| LAMB3 | -3.78402006 | 0.011031423 |
| CMYA5 | 2.210349405 | 0.011286562 |
| FIBIN | 4.289078948 | 0.011314578 |
| HFM1 | 1.702688714 | 0.011342756 |
| RSPO1 | 2.215701036 | 0.011422081 |
| RARA | -1.062569461 | 0.011427245 |
| HIST1H4I | -1.092453002 | 0.011428293 |
| TLR1 | -2.45706575 | 0.011523816 |
| HIST1H4L | -1.378548755 | 0.011544027 |
| DTX1 | -2.323052158 | 0.011609843 |
| TESC | -1.299666745 | 0.011646195 |
| LGI2 | 2.097561214 | 0.01194159 |
| CDCA5 | -2.087039549 | 0.012051006 |
| OAS3 | -2.67272793 | 0.012064075 |
| SKA1 | -2.348492498 | 0.012080977 |
| STIL | -1.368843352 | 0.012085085 |
| B4GALT1 | -1.683000226 | 0.012118376 |
| FLNC | 2.181698883 | 0.012401547 |
| RYR3 | 3.410389403 | 0.012516361 |
| MCTP1 | -2.925496441 | 0.0125718 |
| KLHDC1 | 1.079348087 | 0.012604889 |
| HTR2B | 1.60911279 | 0.012621082 |
| C3orf70 | 2.324965 | 0.012691141 |
| KIF14 | -2.17477015 | 0.012865066 |
| CYBA | -2.048214732 | 0.012971404 |
| ZSWIM6 | -1.095809188 | 0.013053062 |
| ABO | 1.733788663 | 0.013095696 |
| PAG1 | -2.288008168 | 0.013175631 |
| HASPIN | -2.272276374 | 0.013225506 |
| GAS2L3 | -1.563613912 | 0.013252187 |
| PCOLCE2 | 2.499655496 | 0.01331053 |
| ITGA7 | 1.898808036 | 0.013310618 |
| HTR1B | 2.711036788 | 0.013369013 |
| SH2B2 | -2.143419313 | 0.013394179 |
| HIST1H2AB | -1.972635673 | 0.013448634 |
| GPD2 | -1.385077644 | 0.013598186 |
| GCA | -2.367920413 | 0.013623104 |
| ESPL1 | -1.972391068 | 0.013686936 |
| CARD16 | -2.718521014 | 0.013788927 |
| LITAF | -2.748742458 | 0.013867218 |
| NEIL3 | -2.154059406 | 0.013884662 |
| WWC1 | -1.042321345 | 0.013950107 |
| RILPL2 | -1.689720282 | 0.013962113 |
| BIRC5 | -2.477873662 | 0.013975946 |
| ANKRD36 | 1.058389603 | 0.014029774 |
| PKMYT1 | -1.553036322 | 0.014144127 |
| CCDC112 | 1.130076397 | 0.01414978 |
| LRRC31 | 3.367785808 | 0.014222387 |
| CKS2 | -1.803985144 | 0.014318636 |
| DUSP4 | -2.425143443 | 0.014372283 |
| FBP1 | -3.615900413 | 0.014384325 |
| SCGB2A1 | 2.752566667 | 0.014400059 |
| FAM241B | -1.395830251 | 0.014586975 |
| STMN2 | 2.572015497 | 0.014616865 |
| PLEK2 | -1.313136028 | 0.01463472 |
| SLC2A12 | 1.972975477 | 0.014657555 |
| GDF15 | -2.938413887 | 0.014681013 |
| ICAM4 | -4.074054744 | 0.014728109 |
| CASQ2 | 3.439371188 | 0.014782918 |
| HIST1H3G | -2.447266841 | 0.014910644 |
| PTGER3 | 2.325489327 | 0.015041525 |
| MAPK13 | -1.154476804 | 0.015070178 |
| ICOS | -3.753518105 | 0.015096294 |
| IFIT1B | -5.710789057 | 0.015111295 |
| PLAUR | -3.676120622 | 0.015295272 |
| DTX3L | -1.223724957 | 0.015297573 |
| KIAA1210 | -1.691268392 | 0.015351897 |
| KCNAB1 | 2.771160284 | 0.015363791 |
| SLC6A14 | -3.710128752 | 0.015477137 |
| HIST2H2AA3 | -1.845872769 | 0.015546483 |
| THNSL2 | 1.428025488 | 0.015654402 |
| APOL4 | -1.463809552 | 0.015692371 |
| ADGRB3 | 1.294050907 | 0.015753518 |
| BEND6 | 1.224682102 | 0.015764724 |
| RRM2 | -2.219816628 | 0.015779424 |
| CCNJL | -1.536239837 | 0.015781183 |
| BCL3 | -2.293115685 | 0.015797204 |
| TNIP1 | -1.869935097 | 0.015824386 |
| CAMK2N2 | -2.026786224 | 0.015841395 |
| TCEAL1 | 1.056080748 | 0.015842751 |
| PNPLA1 | -4.284764645 | 0.015862674 |
| MATN2 | 2.650079217 | 0.015880702 |
| SOD2 | -3.049139626 | 0.015911042 |
| RBM47 | -2.001180294 | 0.016105089 |
| LGALS9 | -1.537023808 | 0.016158852 |
| HIST1H2BE | -1.285753934 | 0.016178739 |
| TYMS | -1.829857096 | 0.016178938 |
| SCARF1 | -2.785767099 | 0.016222164 |
| ESPN | -2.33012064 | 0.016358553 |
| SPATA25 | 1.53045015 | 0.016387046 |
| SYBU | 2.383212135 | 0.016405854 |
| KIF18B | -2.276138116 | 0.016409398 |
| ATL1 | 1.343232596 | 0.01643644 |
| COL28A1 | 2.076781428 | 0.016610372 |
| CSTA | -4.504757898 | 0.016613242 |
| CMTM6 | -1.469574371 | 0.016778342 |
| DLEU7 | -3.275453134 | 0.016778543 |
| AQP1 | 1.498965193 | 0.016920733 |
| OSR1 | 3.0779456 | 0.016971947 |
| LGMN | -1.527054098 | 0.016993132 |
| SLC16A3 | -2.985537885 | 0.017005508 |
| CDCA8 | -1.77628897 | 0.017096836 |
| AURKA | -1.561612541 | 0.017240767 |
| RASGRP1 | -2.066153896 | 0.017279794 |
| KIAA1549L | 2.407049972 | 0.017316663 |
| C3orf80 | 2.497613598 | 0.017448383 |
| MFSD12 | -1.139682149 | 0.017472891 |
| E2F1 | -1.387851759 | 0.017501963 |
| CXorf21 | -2.996063022 | 0.017511355 |
| TPM1 | 1.346466623 | 0.017601329 |
| ASPM | -2.197650319 | 0.017606967 |
| HOXA13 | 2.630179889 | 0.017655425 |
| RAB9B | 1.414778297 | 0.017701433 |
| CDCA4 | -1.153840053 | 0.017743267 |
| GBP3 | -1.257120842 | 0.017790269 |
| ALOX12B | 3.630749656 | 0.017919542 |
| GRID1 | 2.474331348 | 0.018128107 |
| MICB | -2.200527736 | 0.018256255 |
| NLRP2 | -2.89201948 | 0.01829764 |
| ADAMTSL5 | 1.701707113 | 0.018301578 |
| CLDN23 | -1.233100953 | 0.018316118 |
| PLXNB3 | 2.810776056 | 0.018383261 |
| SIRPA | -2.82724603 | 0.018421416 |
| COL6A3 | 1.197788207 | 0.018432228 |
| HBEGF | -3.809637132 | 0.018551472 |
| MELK | -2.00904127 | 0.018686776 |
| IL6 | -4.836608393 | 0.018719015 |
| ACTC1 | 4.500140266 | 0.018745219 |
| EHD1 | -2.730837524 | 0.018815338 |
| EIF4A3 | -1.408445289 | 0.018862512 |
| SPARCL1 | 1.704347117 | 0.018957945 |
| GADD45B | -2.413592184 | 0.018995608 |
| GDNF | 2.700679006 | 0.01902403 |
| HIST1H2BB | -1.862678149 | 0.019056036 |
| OLFM4 | -3.456955216 | 0.019057827 |
| FHL1 | 1.681881513 | 0.019191414 |
| ZNF521 | 1.337621477 | 0.019214839 |
| JAK3 | -2.572359036 | 0.019317147 |
| BRCA2 | -1.240786802 | 0.019366967 |
| RHOV | -1.884319156 | 0.019369436 |
| OR2W3 | -4.746122938 | 0.019470121 |
| SPC24 | -1.870582037 | 0.01951295 |
| IGFBP5 | 2.809195014 | 0.019544082 |
| E2F7 | -2.368270331 | 0.019563337 |
| SIRPB2 | -3.653406795 | 0.019578343 |
| CD300LB | -3.377689394 | 0.019639382 |
| SIGLEC9 | -3.241001964 | 0.019735888 |
| BVES | 1.697588428 | 0.019811432 |
| PREX1 | -2.314844744 | 0.019976586 |
| TMEM176B | -2.140121754 | 0.020013947 |
| DRAM1 | -2.423639319 | 0.020032523 |
| HK1 | -1.052164503 | 0.020056724 |
| IL4R | -2.220540636 | 0.02011128 |
| VAT1L | 2.747766487 | 0.020153646 |
| LIMK2 | -2.512169014 | 0.020246744 |
| POTEF | 2.844681772 | 0.020341954 |
| FSBP | 1.863742355 | 0.020365312 |
| CDKN1A | -2.456603275 | 0.02037864 |
| SERPINB1 | -2.281974443 | 0.020629022 |
| GNMT | 1.36943987 | 0.020645208 |
| RCC2 | -1.032164835 | 0.020735842 |
| DIO3 | 1.338743048 | 0.020979205 |
| RGS18 | -3.036076502 | 0.021057498 |
| SCO2 | -2.995596873 | 0.021082658 |
| PARP14 | -1.564454614 | 0.0211189 |
| SLC22A3 | 1.915669983 | 0.021133133 |
| IL20RA | 2.558611149 | 0.021169412 |
| NFKB1 | -2.109534852 | 0.021182404 |
| SLC7A5 | -3.307661175 | 0.021227077 |
| C16orf54 | -2.808819661 | 0.021239099 |
| MYCN | -3.157234431 | 0.021307307 |
| TSPAN18 | 1.526159325 | 0.021335495 |
| VCAN | -1.575308396 | 0.021340188 |
| HSD17B6 | 2.980279725 | 0.021354055 |
| NAALAD2 | 1.384842419 | 0.021528258 |
| STC2 | -1.574804961 | 0.021548283 |
| CD69 | -3.689658537 | 0.021634124 |
| CCDC9B | 1.900127441 | 0.021707791 |
| PTGES | -2.765326267 | 0.021710663 |
| CD1A | -3.890121908 | 0.021767065 |
| FABP3 | 2.292952558 | 0.021803931 |
| EDN2 | -2.647855148 | 0.021834802 |
| HP | -1.990900639 | 0.021897243 |
| SLC2A4 | 2.932552779 | 0.021953124 |
| LDB2 | 1.121695337 | 0.022019347 |
| SYPL2 | 1.590003503 | 0.022095933 |
| MREG | -1.808782303 | 0.022287808 |
| CUX2 | -2.48202154 | 0.022348142 |
| ZC3H12A | -2.976015882 | 0.022416515 |
| C10orf55 | -2.607953975 | 0.02249369 |
| MRGPRF | 1.543106162 | 0.022548441 |
| SORCS2 | 2.023360321 | 0.02260519 |
| TLE3 | -1.713551865 | 0.022657289 |
| SMIM32 | -5.357372196 | 0.022681267 |
| PLK5 | 2.873989021 | 0.022724964 |
| LILRA1 | -3.125728755 | 0.022832285 |
| TMEM176A | -2.035329759 | 0.023013217 |
| PGD | -1.895736957 | 0.023070144 |
| APCDD1L | 2.737792866 | 0.023172055 |
| CDK1 | -1.965674378 | 0.023213197 |
| CASP1 | -2.626151884 | 0.023260263 |
| IL15RA | -1.730646495 | 0.023263608 |
| EBF1 | 1.27925799 | 0.023501681 |
| SKA3 | -2.071882836 | 0.023592165 |
| AURKB | -2.257793843 | 0.023609218 |
| GRK6 | -1.186047892 | 0.023620553 |
| DET1 | 1.245069788 | 0.023636725 |
| TRERF1 | 1.000726448 | 0.023699357 |
| NRN1 | 1.182664071 | 0.023715531 |
| HIST2H3D | -1.524104144 | 0.023829979 |
| SHISA6 | 2.124054176 | 0.023987265 |
| APOBR | -2.777198877 | 0.024035882 |
| CENPA | -2.024577403 | 0.024096309 |
| RBKS | -2.420958378 | 0.024133168 |
| CXCR4 | -2.840912256 | 0.024217794 |
| FXYD6 | 2.029711046 | 0.024741788 |
| PLAU | -2.145871777 | 0.024743495 |
| IL27RA | -1.592592399 | 0.024799764 |
| GNAZ | 1.688934243 | 0.024914755 |
| GGT1 | -1.383976496 | 0.024999872 |
| ABHD17C | -1.048107197 | 0.025121539 |
| MSLN | -2.218997926 | 0.025163312 |
| ST6GALNAC2 | -1.418597895 | 0.025201148 |
| SRL | 2.167868898 | 0.025291242 |
| PRSS12 | -2.387036791 | 0.025366501 |
| ZNF728 | 2.410197502 | 0.02539322 |
| NKX3-1 | -1.761402516 | 0.025482759 |
| TCN2 | -1.524180447 | 0.025496241 |
| JPH4 | 1.404372136 | 0.025528539 |
| MX1 | -2.057204584 | 0.025534572 |
| ASXL3 | 1.855558271 | 0.025558631 |
| GRN | -1.904823212 | 0.025605548 |
| NFKBIE | -2.490232208 | 0.025615119 |
| ELF3 | -1.503142919 | 0.025655432 |
| KLF8 | 1.146863502 | 0.025749357 |
| KLF16 | -1.085740691 | 0.025764751 |
| GAREM2 | 1.095002866 | 0.025831711 |
| CD300A | -2.65143192 | 0.025976994 |
| CEACAM4 | -3.453170527 | 0.026176915 |
| OSR2 | 1.333731924 | 0.026231415 |
| CPA3 | 2.868624407 | 0.026444781 |
| LGALS2 | -3.171766618 | 0.026458914 |
| LTB | -2.682878178 | 0.026506118 |
| GPRC5A | -2.124290354 | 0.026507509 |
| CACNA1H | 1.511735564 | 0.026971427 |
| PLSCR2 | -2.810843121 | 0.026997294 |
| THBD | -2.542884399 | 0.027119169 |
| GSTA1 | -3.126102384 | 0.027187194 |
| SELPLG | -2.909680614 | 0.027327433 |
| ASF1B | -2.101613643 | 0.027494915 |
| KIF11 | -1.855890042 | 0.027570932 |
| RNASE2 | -3.517549958 | 0.027668644 |
| RGS16 | -1.809180709 | 0.027712797 |
| TRPV4 | -1.311824513 | 0.027793015 |
| BTBD3 | -1.205055178 | 0.027881335 |
| CADPS2 | 2.079862164 | 0.027965377 |
| NEURL1 | 1.954725541 | 0.028048102 |
| NLRP6 | -3.775371871 | 0.028139407 |
| CCL4L2 | -3.948662684 | 0.02819867 |
| HLF | 1.88065554 | 0.02837073 |
| GPAT3 | -1.790375764 | 0.028396214 |
| CHST15 | -2.129714165 | 0.028415152 |
| SGO1 | -1.987664486 | 0.028447092 |
| SIPA1L2 | -1.5014516 | 0.028551476 |
| MS4A14 | -2.362241409 | 0.028565721 |
| HBM | -7.579533031 | 0.02867084 |
| KCNN2 | 2.760480062 | 0.02869361 |
| RUNX3 | -2.803427946 | 0.028801625 |
| PTGS1 | -1.667063843 | 0.028875058 |
| WNT11 | -1.975846024 | 0.028909391 |
| FANCD2 | -1.121853444 | 0.029298071 |
| E2F8 | -2.227923275 | 0.029318767 |
| RIPK2 | -2.229297441 | 0.029411406 |
| CAPG | -2.972354624 | 0.029454015 |
| BCL11A | -1.28047696 | 0.02953727 |
| KCNE4 | 1.840992431 | 0.029552388 |
| SYNGR1 | 1.274770144 | 0.029577038 |
| SUSD2 | 1.203290454 | 0.029613497 |
| KCNK3 | 1.647701423 | 0.029722001 |
| RRAGD | -2.640111398 | 0.02982382 |
| PRKCD | -1.727795331 | 0.02984583 |
| GPR37 | 1.979676188 | 0.029880535 |
| PTPRE | -2.50342193 | 0.030042506 |
| SLC35F3 | -1.62086787 | 0.030233639 |
| GSTM2 | 1.815891823 | 0.030299868 |
| ADAM11 | 1.091544112 | 0.030433219 |
| CNR1 | 2.619569212 | 0.030444758 |
| IGSF3 | -1.363894888 | 0.030526958 |
| TMEM200B | 1.647901622 | 0.030620034 |
| TGM3 | -2.276896399 | 0.030651548 |
| CCNB1 | -1.663318099 | 0.030660211 |
| MYL3 | 1.071407392 | 0.030689899 |
| NEK2 | -2.355691238 | 0.030798914 |
| HYI | 1.001671687 | 0.03100938 |
| SNTG2 | 1.736551982 | 0.031131296 |
| GP1BA | -2.517352009 | 0.03130653 |
| ST6GALNAC5 | 1.845895443 | 0.031489097 |
| PLSCR1 | -2.15475301 | 0.031498132 |
| PEAK3 | -2.890077326 | 0.031619355 |
| NCOA4 | -1.388740374 | 0.031659027 |
| CGAS | -1.750886225 | 0.031669244 |
| LILRB5 | -2.431119992 | 0.031902915 |
| HIST1H2BH | -1.573921621 | 0.03191234 |
| ZNF208 | 1.458826697 | 0.032038157 |
| KCNK13 | 2.746205801 | 0.032061679 |
| METRNL | -2.253320233 | 0.032205894 |
| SNN | -1.785593129 | 0.032267269 |
| GAA | -1.373484225 | 0.032285904 |
| PDZK1 | -2.761506656 | 0.032314013 |
| SUSD1 | -1.006374004 | 0.032501424 |
| C17orf107 | -2.397167671 | 0.032575351 |
| ANK1 | -2.600368706 | 0.03260127 |
| OAS2 | -2.394816204 | 0.032641005 |
| IGFBP2 | -1.427343726 | 0.032667287 |
| PRRT2 | 1.392860932 | 0.032668885 |
| FOXO6 | 2.736183188 | 0.032673263 |
| FGFBP2 | -3.853253563 | 0.032686791 |
| IFIT3 | -2.344797229 | 0.032690344 |
| RANBP17 | 1.372513699 | 0.032776855 |
| TNFRSF9 | -2.906146746 | 0.032799354 |
| TFRC | -1.545710298 | 0.033000787 |
| BLM | -1.286207595 | 0.033182506 |
| AGAP2 | -1.517716437 | 0.033224028 |
| TBXAS1 | -2.423145036 | 0.033288556 |
| PTCHD1 | 2.411932602 | 0.033344068 |
| PLA2G7 | -1.930493448 | 0.033353264 |
| P2RY6 | -1.255818981 | 0.033371841 |
| HJURP | -2.329741896 | 0.033449288 |
| UBD | -6.785733176 | 0.033517436 |
| BUB1 | -1.921448232 | 0.03358706 |
| NME8 | -2.566787196 | 0.033728719 |
| HPGD | 1.708017515 | 0.03384039 |
| HAS3 | -1.582765036 | 0.033897122 |
| CD209 | -1.922526106 | 0.033931766 |
| IFNAR2 | -1.226528202 | 0.033940881 |
| DPEP2 | -2.943845046 | 0.033945312 |
| STAT4 | -2.853290446 | 0.033951872 |
| TNFRSF18 | -2.207439773 | 0.034106787 |
| MYRIP | 1.292350206 | 0.034112999 |
| COL15A1 | 1.010230102 | 0.034122891 |
| NRTN | -1.096054629 | 0.034383446 |
| NT5DC3 | 1.187934049 | 0.034399754 |
| HS3ST3B1 | -3.271682554 | 0.034414277 |
| HLA-B | -2.101827208 | 0.034576609 |
| HLA-DQB1 | -2.796456328 | 0.034585395 |
| HR | -1.568458098 | 0.034609328 |
| HRH2 | -2.561748286 | 0.034672376 |
| MCM10 | -1.731017149 | 0.034716218 |
| MAP2 | 1.480431982 | 0.034820365 |
| TGFA | -1.457550437 | 0.034822562 |
| MYORG | 1.444006066 | 0.034883503 |
| HELB | -1.43498403 | 0.034919492 |
| C6orf223 | -3.32802143 | 0.034919907 |
| PDK1 | -1.395839118 | 0.034947869 |
| SLC25A27 | 1.083256484 | 0.035048384 |
| VEGFA | -1.921997216 | 0.035164531 |
| TET3 | -1.109913464 | 0.035233015 |
| NKD2 | -1.78825066 | 0.035295175 |
| RAMP3 | 1.623701689 | 0.035299709 |
| INAVA | -1.83210288 | 0.035349302 |
| LURAP1L | 1.097786918 | 0.035354282 |
| CXCL1 | -3.573121374 | 0.035443278 |
| DYSF | -1.760569638 | 0.035525418 |
| LRRC26 | 2.998168841 | 0.035527532 |
| NOX4 | 1.748348712 | 0.035606635 |
| CIT | -1.279707785 | 0.03560928 |
| TLR6 | -1.606665259 | 0.035675998 |
| ARHGAP11A | -1.688601623 | 0.035737288 |
| TRAF1 | -2.640350251 | 0.035760593 |
| SPAG5 | -1.398840644 | 0.035814525 |
| HIST1H2BG | -1.044341082 | 0.03582037 |
| ST8SIA2 | -2.11628667 | 0.035844867 |
| IL23A | -2.146473996 | 0.035857069 |
| EML5 | 1.420105379 | 0.035857613 |
| HELZ2 | -1.673341839 | 0.035928587 |
| DCAF12 | -1.443703371 | 0.035956123 |
| DNAJB1 | -1.708427716 | 0.035985858 |
| CST7 | -2.796324646 | 0.036055811 |
| TUB | 1.284312142 | 0.036094274 |
| NR1H3 | -2.119778447 | 0.036110205 |
| HIST1H3A | -1.537135653 | 0.036181182 |
| ADAMTS9 | -1.321519259 | 0.036233289 |
| NFKB2 | -2.14187604 | 0.036253686 |
| SAA1 | -3.853520437 | 0.036338161 |
| DEPDC1B | -1.841335239 | 0.036429242 |
| GFRA1 | 1.774843969 | 0.036479729 |
| NCR3LG1 | -1.948939903 | 0.036781318 |
| TMEM171 | -7.021366748 | 0.036825205 |
| SNAI1 | -2.052161393 | 0.036907449 |
| NFKBID | -2.608054496 | 0.037205939 |
| GPR88 | 3.19798487 | 0.037288529 |
| DIAPH3 | -1.89195813 | 0.037371421 |
| TNFAIP3 | -3.014292685 | 0.037389892 |
| CXCL8 | -4.934658858 | 0.037630607 |
| MAD2L1 | -1.552251661 | 0.037696052 |
| ATP1B2 | 1.651162849 | 0.037811141 |
| SH3TC1 | -2.037770315 | 0.038005706 |
| SHISA8 | 2.444093419 | 0.038064882 |
| ABCC9 | 1.729603579 | 0.038092849 |
| HCLS1 | -2.631389305 | 0.038128516 |
| CDT1 | -1.421584445 | 0.038140222 |
| PTH2R | 2.377362208 | 0.038143212 |
| OSGIN1 | -2.307972852 | 0.038164199 |
| FAM110A | -1.367747271 | 0.038288276 |
| IL31RA | -3.343037916 | 0.038349484 |
| HIVEP3 | -1.48237983 | 0.03837125 |
| FLOT2 | -1.234783485 | 0.038464398 |
| YPEL4 | 1.18440994 | 0.038521114 |
| GLUL | -2.101174311 | 0.038647612 |
| CD47 | -1.152348521 | 0.038675965 |
| CASP4 | -1.944463138 | 0.038836296 |
| FCHO1 | -2.216840849 | 0.038844364 |
| LRRC17 | 2.341927998 | 0.038852879 |
| KNL1 | -1.851131402 | 0.038909168 |
| CCL3L1 | -3.583786431 | 0.039134344 |
| EXO1 | -1.988790182 | 0.039146002 |
| IL32 | -1.813184602 | 0.039226847 |
| SEMA5A | 1.801050985 | 0.03923264 |
| NECTIN2 | -1.217119684 | 0.039336863 |
| PDE3B | -2.06540007 | 0.039411622 |
| LAT2 | -2.326212116 | 0.039596571 |
| GPSM3 | -2.548730683 | 0.03960746 |
| SLC5A9 | 2.289311791 | 0.039615118 |
| HAVCR1 | -3.182636271 | 0.039671077 |
| TOP2A | -2.204443581 | 0.039749459 |
| CEP55 | -1.855050699 | 0.039807803 |
| STEAP3 | -1.553461651 | 0.039976618 |
| SDC4 | -1.466475208 | 0.04020425 |
| CTSL | -2.904492103 | 0.040275139 |
| DLGAP5 | -2.345066389 | 0.040331839 |
| SAMHD1 | -1.62325093 | 0.040514283 |
| RBM20 | 1.452749162 | 0.040527756 |
| IFNGR2 | -1.472479761 | 0.040629879 |
| PLEKHO2 | -1.868715414 | 0.040801998 |
| KIF4A | -1.801860346 | 0.04082365 |
| ISG20 | -2.260207448 | 0.040840033 |
| SBNO2 | -1.85882976 | 0.040937549 |
| DEFB124 | 2.13652692 | 0.040984953 |
| JPT1 | -1.135920632 | 0.041056688 |
| CORIN | 2.003270719 | 0.041071913 |
| NXT1 | -1.03997048 | 0.041103343 |
| ASCL2 | -1.562931844 | 0.041152967 |
| GINS4 | -1.359405714 | 0.041231068 |
| SLIT3 | 1.954684179 | 0.041361361 |
| TRPM3 | 2.548032036 | 0.04154264 |
| IRF7 | -2.079692907 | 0.041558749 |
| NUDT13 | 1.237759574 | 0.041631253 |
| GPR161 | 1.711796702 | 0.041638506 |
| BAZ1A | -1.400017281 | 0.041684081 |
| PTGS2 | -3.811367206 | 0.041848775 |
| HILPDA | -1.502319354 | 0.041900387 |
| B4GALT5 | -1.566466318 | 0.041986149 |
| CCDC68 | 1.259909294 | 0.042037074 |
| ZBTB32 | -2.328639992 | 0.042077934 |
| SMPD3 | -1.681378588 | 0.042123718 |
| KDM6B | -2.203768047 | 0.042130965 |
| CCDC192 | 3.037074658 | 0.042150494 |
| COL21A1 | 2.083706392 | 0.042161114 |
| SELP | 1.139848059 | 0.042284331 |
| CSF2 | -4.1125023 | 0.042435336 |
| VNN1 | -2.983875412 | 0.042709068 |
| CRYBB3 | 1.564459281 | 0.042765154 |
| MKI67 | -1.856897134 | 0.042813042 |
| LCP1 | -2.688202267 | 0.042823054 |
| IL1A | -4.675248107 | 0.043071155 |
| FSTL3 | 1.107830512 | 0.04307995 |
| SLC15A2 | 2.17371938 | 0.043095372 |
| IGFBP6 | 2.489318452 | 0.043151884 |
| SLC29A3 | -1.390484206 | 0.043167637 |
| PLEKHB2 | -1.500662085 | 0.043262211 |
| SULT4A1 | -1.030060669 | 0.04343591 |
| CBARP | -1.496941688 | 0.043466536 |
| APOBEC3H | -2.152309633 | 0.043482041 |
| NR3C2 | 1.580127677 | 0.043500302 |
| NDC80 | -1.858976862 | 0.043514187 |
| ANKRD39 | 1.146743477 | 0.04358443 |
| SH2B3 | -1.508420734 | 0.043779943 |
| PIANP | 1.583179169 | 0.043866242 |
| TRIB3 | -1.485075976 | 0.043930027 |
| MASP1 | 2.271134614 | 0.043932191 |
| CTSB | -2.359557936 | 0.043934042 |
| ZNF710 | -1.057926676 | 0.043960242 |
| DOT1L | -1.023846998 | 0.04400636 |
| C15orf39 | -1.245005286 | 0.044042854 |
| NDUFA4L2 | 1.634447927 | 0.04405917 |
| SLC6A9 | 1.691912024 | 0.044119406 |
| CDCA2 | -2.158532557 | 0.044141431 |
| ZNF483 | 1.560642517 | 0.04414981 |
| POPDC3 | 2.15507289 | 0.04418333 |
| LY86 | -2.351842892 | 0.044208908 |
| SMOC1 | 1.932148913 | 0.04423786 |
| MYD88 | -1.882157713 | 0.044272659 |
| CPVL | -1.895667841 | 0.044316823 |
| MCOLN2 | -2.553447692 | 0.044377759 |
| ABCA1 | -2.087828104 | 0.044503547 |
| TMPRSS3 | -1.598833371 | 0.044519586 |
| HSH2D | -2.001084761 | 0.044596804 |
| LACTB | -2.045363766 | 0.044599608 |
| SOX15 | 1.938496176 | 0.044621779 |
| LAP3 | -1.922524206 | 0.044637453 |
| IL1RAP | -2.029632334 | 0.044654981 |
| ADA2 | -1.684604557 | 0.044656915 |
| SCGB3A1 | 2.994349223 | 0.044700007 |
| ADGRE2 | -2.853888044 | 0.044745939 |
| CCDC136 | 1.391868341 | 0.044749762 |
| CDKL1 | 1.355577206 | 0.04476062 |
| C11orf87 | 4.430902101 | 0.044779133 |
| EVI2A | -2.404183761 | 0.044869097 |
| CREM | -2.129376908 | 0.044945116 |
| ASPA | 2.053370153 | 0.044983516 |
| TPX2 | -1.882923641 | 0.045048694 |
| TNFRSF11A | -1.243017118 | 0.045065472 |
| GTSE1 | -2.206376098 | 0.045201174 |
| STAT1 | -2.003558854 | 0.04538754 |
| KIAA0040 | -1.102673631 | 0.045469339 |
| LAMP1 | -1.076913802 | 0.045595222 |
| SEMA6B | -1.970541219 | 0.045918983 |
| HIST1H4J | -1.092825672 | 0.045966427 |
| PGLYRP1 | -2.912541207 | 0.045969813 |
| RBM38 | -1.738215475 | 0.046110114 |
| AP2S1 | -1.049656807 | 0.046213072 |
| ARNTL2 | -2.262087586 | 0.046467598 |
| SECTM1 | -2.630184334 | 0.046476171 |
| EFHD2 | -2.055140104 | 0.046518191 |
| MAP2K3 | -1.878498291 | 0.046606428 |
| NECAP2 | -1.110448813 | 0.046647214 |
| COL4A6 | 3.071724603 | 0.047004556 |
| ASIC3 | 1.280539936 | 0.047009168 |
| RGS19 | -1.523111698 | 0.04717305 |
| LRRC1 | 1.609239237 | 0.047273505 |
| KCNE3 | -1.285484727 | 0.047324992 |
| SOCS3 | -3.10469903 | 0.047389791 |
| ESCO2 | -1.989813016 | 0.047399683 |
| NFKBIA | -2.346752524 | 0.047686383 |
| INAFM2 | -1.280995532 | 0.047726494 |
| SPOCK2 | -2.301229705 | 0.047813413 |
| SUSD6 | -1.942757715 | 0.04785298 |
| CCNB2 | -1.771011147 | 0.047891581 |
| NCAPG | -1.975069943 | 0.048249784 |
| MEGF9 | -1.005602395 | 0.04826446 |
| PLCXD2 | -1.682366246 | 0.048273624 |
| B2M | -1.952620714 | 0.048285496 |
| CDKN2D | -2.026478167 | 0.04832401 |
| ISG15 | -1.990963558 | 0.048390978 |
| CES4A | 1.912689737 | 0.04853571 |
| SYTL3 | -1.998542778 | 0.048569652 |
| DUSP10 | -2.284642822 | 0.048688255 |
| DUSP2 | -2.485979915 | 0.048746284 |
| HIST1H3I | -1.086457576 | 0.04884251 |
| ACSL4 | -1.222991623 | 0.048915169 |
| IQGAP3 | -2.051525512 | 0.048925267 |
| KIF15 | -1.813880868 | 0.048998637 |
| HIVEP2 | -1.240253426 | 0.049070499 |
| CHSY1 | -1.09736838 | 0.049148194 |
| FAM13A | 1.797427959 | 0.049233232 |
| EMCN | 1.345091119 | 0.049268541 |
| FANCA | -1.236044573 | 0.049370922 |
| CD5 | -2.241815563 | 0.049380228 |
| PTGIR | -2.547158787 | 0.049386882 |
| ANKS1B | 2.128208063 | 0.04942391 |
| ST8SIA4 | -2.124863951 | 0.049595246 |
| FCRL2 | -2.441338147 | 0.049630361 |
| TCEA3 | 1.692002385 | 0.049650501 |
| GZMH | -2.551557211 | 0.049809583 |
| LINC00632 | 3.353052657 | 1.40896E-13 |
| AC053503.4 | 6.463095285 | 7.75259E-07 |
| MAPK6P5 | 7.759141628 | 3.94127E-06 |
| FRMD6-AS2 | 7.178911467 | 5.3268E-06 |
| AL450405.1 | -5.697962824 | 7.27946E-06 |
| MBNL1-AS1 | 1.982507102 | 2.0158E-05 |
| ADIRF-AS1 | 1.073933593 | 2.50355E-05 |
| AC092162.2 | 5.117650072 | 3.29673E-05 |
| AF064858.1 | -2.458924348 | 4.09066E-05 |
| AF001548.1 | 5.419655256 | 4.52586E-05 |
| AF001548.3 | 5.571090586 | 4.75397E-05 |
| AL161457.1 | 3.476055603 | 5.23159E-05 |
| AC053503.6 | 5.131266179 | 8.57322E-05 |
| RPL24P8 | 3.144955776 | 0.00012142 |
| MIR1-1HG-AS1 | 8.825989943 | 0.000187579 |
| AF064858.3 | -1.993452959 | 0.000205151 |
| RPL7P18 | -4.955476408 | 0.00020752 |
| AC100803.2 | 5.038390701 | 0.000328058 |
| AC011511.5 | -5.497631784 | 0.000355846 |
| AL031429.2 | 2.925582753 | 0.000570641 |
| RERG-IT1 | 5.083627725 | 0.000614087 |
| AC017099.1 | 1.639114535 | 0.000764948 |
| RSU1P3 | 1.936517328 | 0.000839481 |
| KCNMA1-AS1 | 3.136812964 | 0.00089898 |
| AC083855.2 | -1.912528665 | 0.000957745 |
| AC002511.1 | -3.429253667 | 0.000960308 |
| VN1R84P | -6.202821392 | 0.000987437 |
| AC068733.3 | 1.417033215 | 0.001023048 |
| AP001056.1 | -6.150295632 | 0.001048723 |
| LINC01482 | 1.52807027 | 0.001062155 |
| AC008760.2 | -2.331864069 | 0.001102213 |
| AADACP1 | 1.477284651 | 0.001232545 |
| AC120498.6 | 2.0313296 | 0.001247628 |
| AC117498.3 | 2.763063955 | 0.001284466 |
| AL354718.1 | 1.783512776 | 0.001403822 |
| ACTA2-AS1 | 2.142173917 | 0.001475636 |
| RPL21P99 | 1.808503713 | 0.001491002 |
| AC092718.4 | -1.782223992 | 0.001519276 |
| AC098679.2 | 1.545904916 | 0.00158574 |
| AP000344.1 | 2.515182961 | 0.001587789 |
| AC097515.1 | 3.332784518 | 0.0016048 |
| AP000962.2 | 1.857663973 | 0.00183007 |
| GOLGA2P10 | 1.839450536 | 0.001848572 |
| TRAV1-2 | -6.425267037 | 0.001864463 |
| SERPINB9P1 | -3.401750817 | 0.001986954 |
| MEF2C-AS1 | 2.175717565 | 0.001999042 |
| DIO2-AS1 | -3.431877608 | 0.002431265 |
| AC124312.5 | 1.092254872 | 0.0025104 |
| Z73965.1 | 2.1257512 | 0.0025507 |
| AP001107.8 | 1.328579298 | 0.002574086 |
| PGM5-AS1 | 6.687896433 | 0.002642345 |
| TRHDE-AS1 | 4.0537395 | 0.002676468 |
| AC005180.1 | 3.009980039 | 0.00294519 |
| AL357568.2 | 1.563116742 | 0.003050424 |
| PTCHD1-AS | 2.941777612 | 0.003179858 |
| AC025580.2 | -4.628684997 | 0.003328085 |
| AC090740.1 | 6.373631653 | 0.003330948 |
| IGKV3-20 | 3.900707755 | 0.003591051 |
| AC024901.1 | 3.379814174 | 0.003685553 |
| AP003355.2 | 3.349346678 | 0.003797939 |
| AC095050.1 | 3.126976309 | 0.003845397 |
| DIO3OS | 1.783288509 | 0.003858516 |
| RRM2P3 | 1.292461971 | 0.003900852 |
| U62317.1 | -7.133322811 | 0.004018416 |
| HRAT92 | 2.050329343 | 0.004059193 |
| SDCBPP1 | -5.092551283 | 0.004079007 |
| AC053513.1 | 1.100931657 | 0.004370573 |
| IL12A-AS1 | 4.344427098 | 0.004960637 |
| IGLC2 | 2.309693686 | 0.005092653 |
| SULT1C2P1 | 4.192683343 | 0.005146753 |
| AC012409.3 | 1.781207193 | 0.005313461 |
| AC140479.4 | -3.482970375 | 0.00539724 |
| AC073174.1 | -3.518773069 | 0.005403727 |
| GK-AS1 | -6.522961012 | 0.005543508 |
| AP000640.2 | -2.794544509 | 0.005902921 |
| AL359504.2 | 1.197211777 | 0.005913943 |
| AC103724.3 | -5.952267023 | 0.00601387 |
| RPL21P5 | -1.751218703 | 0.006129648 |
| AC245140.3 | 2.340979583 | 0.006203715 |
| AC241585.2 | -1.201451524 | 0.006351961 |
| AC007336.3 | 1.061698622 | 0.006404865 |
| MIR99AHG | 2.043557965 | 0.006428735 |
| AL391832.2 | -3.782246601 | 0.006449897 |
| LINC01116 | 1.218218508 | 0.006454425 |
| AL391001.1 | 1.835745565 | 0.006526237 |
| AC015912.3 | -3.271948139 | 0.006540142 |
| PEBP1P3 | -5.371986194 | 0.006607626 |
| AL355297.3 | 1.780344543 | 0.00661742 |
| NR2F2-AS1 | 1.301934708 | 0.007079423 |
| AC019117.1 | -2.746368822 | 0.007085273 |
| AL354798.1 | -3.129650398 | 0.007425604 |
| AC116552.1 | 1.024611463 | 0.007608101 |
| SORD2P | 3.317145408 | 0.007669893 |
| AC018552.2 | -4.669180716 | 0.00767377 |
| AP003465.1 | 1.198520765 | 0.007804151 |
| AC011472.4 | 4.099858165 | 0.007812485 |
| AC124798.1 | -2.654780869 | 0.007823263 |
| AC026310.3 | -3.508033663 | 0.00786623 |
| AC091185.1 | 1.003077114 | 0.007887984 |
| FGF14-IT1 | 2.088358369 | 0.008010918 |
| AL117336.2 | -3.118163518 | 0.008369828 |
| AL590560.1 | 1.651905848 | 0.008485783 |
| AC022306.2 | 1.241570451 | 0.008514177 |
| AC011511.2 | -2.713529692 | 0.008515544 |
| TRGV6 | -6.163272852 | 0.008544995 |
| AC021739.3 | -1.600343971 | 0.008661158 |
| AC007563.2 | 3.040410967 | 0.008682591 |
| AC129492.1 | 4.220344907 | 0.009032355 |
| AP002800.1 | 3.714649423 | 0.009051056 |
| AC020656.1 | -4.505729964 | 0.009087707 |
| ADORA2A-AS1 | -3.666602591 | 0.009104495 |
| MYLK-AS1 | 1.0061781 | 0.009266489 |
| AC091057.2 | 1.836013481 | 0.009351852 |
| AC090825.1 | 1.316417236 | 0.010046714 |
| PRR7-AS1 | -1.574298225 | 0.010168304 |
| MEIS1-AS3 | 1.851894787 | 0.010600273 |
| AC015912.2 | -2.52163179 | 0.01078054 |
| AC079328.2 | 1.655284029 | 0.01099753 |
| AC012409.5 | 1.755706601 | 0.011146842 |
| CD300H | -4.744664599 | 0.011157128 |
| FAM106A | 1.337107651 | 0.011169122 |
| CYP2D7 | -1.091534403 | 0.011492695 |
| AL122125.1 | 1.604053216 | 0.011889815 |
| TREML3P | -6.801775382 | 0.011989225 |
| AC069079.1 | 1.795663368 | 0.012053149 |
| RAMP2-AS1 | 2.404947204 | 0.012064116 |
| AC021088.1 | 2.282604699 | 0.012177674 |
| AC008750.1 | -3.337606531 | 0.012283188 |
| CXXC5-AS1 | -2.172416975 | 0.012614639 |
| AL583722.4 | -3.508768801 | 0.012734944 |
| AC103996.3 | 3.952913533 | 0.012807463 |
| AP000763.3 | -2.11346283 | 0.012853431 |
| AC007342.7 | 1.597469839 | 0.012899028 |
| AL021937.3 | 1.616470511 | 0.012957064 |
| PRO1804 | 1.343677472 | 0.013224568 |
| AC012085.2 | 3.485836794 | 0.013274238 |
| AC009133.4 | -1.437725786 | 0.013330936 |
| CSPG4P10 | 1.792569871 | 0.013405658 |
| AC025165.1 | 1.43911329 | 0.013503066 |
| AL356273.3 | -3.213859849 | 0.013553836 |
| AL162424.1 | -1.805294255 | 0.013879993 |
| IGHG3 | 2.059478533 | 0.013924727 |
| AC083837.2 | -1.774372614 | 0.014036371 |
| AF127936.1 | 2.036328295 | 0.014054895 |
| AC104116.1 | -1.412442356 | 0.014201424 |
| AC109347.2 | -1.643916985 | 0.014361884 |
| AC120498.8 | 3.78283499 | 0.014383785 |
| AC090517.5 | 1.023720021 | 0.014457782 |
| AC015912.1 | -1.732049691 | 0.0149634 |
| AC063944.1 | 3.266490362 | 0.014992877 |
| AP001615.1 | -1.200696066 | 0.015023107 |
| C10orf25 | 1.000773098 | 0.015039585 |
| MRPS18CP6 | 1.676395929 | 0.015226874 |
| AC099489.2 | -5.022781262 | 0.015627225 |
| AC114488.2 | 1.493334825 | 0.01569628 |
| LINC00677 | -3.537237996 | 0.015755697 |
| AC011247.1 | 2.693682425 | 0.015999782 |
| LINC02137 | 5.792679602 | 0.016020337 |
| ITGA6-AS1 | -1.431241446 | 0.016024328 |
| AC090510.2 | 1.736394088 | 0.016174877 |
| AC090409.1 | 1.332405558 | 0.016234151 |
| GAS1RR | 2.691029212 | 0.016664878 |
| CHMP1B-AS1 | -1.810127179 | 0.016737359 |
| OCLNP1 | 2.965321679 | 0.016751585 |
| LINC00484 | 3.847430572 | 0.01676318 |
| AL050404.1 | 3.053450785 | 0.016793703 |
| GBA3 | -1.28580109 | 0.016954577 |
| FLG-AS1 | 1.391341423 | 0.017224114 |
| CR392039.4 | -3.747653636 | 0.017286542 |
| ELF3-AS1 | -1.102089101 | 0.01730574 |
| AL391822.1 | 2.869639417 | 0.01735251 |
| AC025031.3 | -2.531246036 | 0.017466074 |
| MEG8 | 1.05354417 | 0.017512018 |
| AL157714.2 | 2.892279065 | 0.01753727 |
| AC004803.1 | 1.380512515 | 0.017547248 |
| AC026367.3 | 1.903343576 | 0.017585676 |
| AL391069.2 | -1.989694503 | 0.017607545 |
| LINC02541 | -4.405482942 | 0.01764248 |
| AP000525.1 | -3.072163971 | 0.017994713 |
| AL512288.1 | -1.166090467 | 0.01849239 |
| AC069528.2 | -2.650290848 | 0.018514107 |
| AL078621.3 | 1.248759148 | 0.018809612 |
| AC092802.1 | 1.32000259 | 0.018851206 |
| FRG1JP | 1.632436659 | 0.019138542 |
| AL121603.2 | -1.439586684 | 0.019259761 |
| AC004817.3 | -3.783552382 | 0.019383191 |
| LINC01088 | 3.511376105 | 0.019552584 |
| ZNF460-AS1 | 1.311602927 | 0.019588978 |
| AL359715.1 | 1.612927506 | 0.019624037 |
| AP001533.1 | 2.107891245 | 0.019629702 |
| LINC01839 | 2.080987051 | 0.019718095 |
| AC079753.1 | -4.201404269 | 0.019831473 |
| RPL39P3 | -1.085992194 | 0.0199781 |
| RHOT1P1 | -2.366234588 | 0.020069046 |
| AL353763.1 | 1.386566751 | 0.02008149 |
| LINC01303 | -3.593561107 | 0.020218226 |
| LINC01678 | -3.89547178 | 0.020221193 |
| CATIP-AS1 | -2.245278296 | 0.020461793 |
| ROCK1P1 | 1.733012283 | 0.020552895 |
| RPL23AP35 | 2.076546267 | 0.021084944 |
| AP005329.2 | 3.336901847 | 0.021385117 |
| HLA-DQB1-AS1 | -2.968852792 | 0.021647729 |
| AC005264.1 | -3.395725971 | 0.021911605 |
| AC007637.1 | 1.48069511 | 0.022188598 |
| AL121890.2 | 2.311209664 | 0.022294863 |
| AC040934.1 | 1.042444228 | 0.022454111 |
| AC073046.1 | -1.528925453 | 0.022584257 |
| AC078909.1 | 2.917830969 | 0.022626902 |
| AP002840.2 | 2.161335059 | 0.022725404 |
| AL136090.1 | 1.673239555 | 0.02284844 |
| AC007787.2 | 2.121693081 | 0.022871805 |
| LINC00884 | -2.530940723 | 0.022889134 |
| AC011676.2 | -2.317776166 | 0.023031307 |
| RPL7L1P9 | 1.877597261 | 0.023366409 |
| DBH-AS1 | 1.332326551 | 0.023658044 |
| LINC01579 | 3.333894651 | 0.023857757 |
| AC105914.2 | 3.653096997 | 0.023899879 |
| AC007541.1 | 1.672007439 | 0.024181286 |
| LINC02154 | -5.87975357 | 0.024384536 |
| AC019254.1 | -3.356882696 | 0.02447909 |
| LINC01506 | -3.64663354 | 0.024536109 |
| DUXAP8 | -2.553247382 | 0.024676154 |
| AP001412.1 | 1.593564244 | 0.024898813 |
| NHEG1 | 5.06402714 | 0.024954346 |
| C9orf106 | 1.516592589 | 0.025098225 |
| LINC00337 | -1.011880627 | 0.025114862 |
| AL683842.1 | -2.349156762 | 0.025247094 |
| AC073109.1 | 1.774482804 | 0.025455462 |
| AC003965.2 | -2.990426755 | 0.025752083 |
| AC009652.1 | 4.041552565 | 0.025889063 |
| HMGN2P15 | 2.310899777 | 0.025921678 |
| FAM66D | 1.373394588 | 0.026040589 |
| AC011472.1 | 2.141141185 | 0.026136012 |
| AL132642.1 | 2.389737606 | 0.026351896 |
| AL590006.1 | 1.880561594 | 0.026410382 |
| AC135279.3 | -2.565676123 | 0.026440107 |
| ARHGEF7-IT1 | 2.147099143 | 0.026506279 |
| AC147651.3 | -3.204615869 | 0.026682103 |
| AC084290.1 | -2.987266038 | 0.026893716 |
| LINC00899 | 1.471904558 | 0.027034916 |
| CSF2RBP1 | -6.358695708 | 0.027057414 |
| AL118508.3 | -5.923474463 | 0.027069824 |
| AC078909.2 | 3.326577832 | 0.027169341 |
| AC012100.2 | -1.414500689 | 0.027374296 |
| AC244034.1 | -3.651847842 | 0.027455327 |
| AL008635.1 | -2.680603543 | 0.027504693 |
| AC067945.1 | -2.666389058 | 0.027756484 |
| AP001922.6 | 2.940958309 | 0.027863946 |
| AL157871.4 | -2.669642763 | 0.02790473 |
| LINC02470 | -6.720331401 | 0.027917925 |
| MTND3P12 | -4.755667731 | 0.028105889 |
| LINC01270 | -1.964786459 | 0.028228036 |
| AC037459.2 | 1.008736983 | 0.028303607 |
| AC245060.5 | 1.535794134 | 0.028343822 |
| AC243562.2 | 1.425824527 | 0.02852371 |
| MFAP1P1 | 1.523645759 | 0.028588406 |
| LINC01215 | -2.760224868 | 0.028647809 |
| AC083949.1 | -1.8174868 | 0.028685727 |
| LINC01237 | 1.670838974 | 0.02868601 |
| MIR4697HG | 2.538679912 | 0.028815333 |
| FLJ22447 | -2.693770621 | 0.028839265 |
| AC007728.2 | -2.61758897 | 0.028993969 |
| AC124014.1 | -3.241447734 | 0.029125658 |
| AC009145.2 | 1.550495996 | 0.029325618 |
| SUGT1P4-STRA6LP | 1.478666507 | 0.029740404 |
| TMC3-AS1 | 1.740102858 | 0.029818597 |
| AC129492.6 | 5.18147209 | 0.029883601 |
| BX890604.1 | 1.879981426 | 0.030008224 |
| AL035425.1 | 2.990433996 | 0.030008641 |
| BMS1P10 | 1.232350774 | 0.03007954 |
| ADAMTS9-AS2 | 1.8357749 | 0.030091111 |
| RPL36AP33 | 1.449110381 | 0.03010981 |
| AL512274.1 | -1.258967059 | 0.030294827 |
| AC010536.2 | 1.48377843 | 0.030380531 |
| ZNF295-AS1 | -1.148797426 | 0.030393259 |
| DISC1FP1 | 4.007680976 | 0.030601422 |
| AC091057.1 | -1.2357486 | 0.030672552 |
| AL445253.1 | -1.077095585 | 0.030675945 |
| AL138895.1 | 1.903510936 | 0.03088271 |
| AC004492.1 | 1.082424781 | 0.030997958 |
| AC073352.2 | -3.440739971 | 0.031243026 |
| AL359715.2 | 1.583117829 | 0.031531168 |
| HLA-DPA3 | -5.997425836 | 0.031934732 |
| AC027796.1 | 1.733068147 | 0.031937757 |
| AC074050.4 | -1.556011904 | 0.032000043 |
| AC005548.1 | 2.736024841 | 0.032312637 |
| AL512303.1 | 1.759457343 | 0.032399576 |
| AC021766.1 | 1.292226526 | 0.032621398 |
| AC006449.2 | -1.252030678 | 0.032669028 |
| MTND5P26 | 1.340041337 | 0.032689526 |
| IL10RB-DT | -1.922125678 | 0.032888413 |
| IER3-AS1 | -2.933094741 | 0.033281849 |
| AC083837.1 | -2.20394337 | 0.033388213 |
| AP001324.3 | 4.485398651 | 0.033466277 |
| SPATA20P1 | -5.553273094 | 0.03349349 |
| AC005332.2 | 1.138608786 | 0.033535972 |
| AC106739.1 | -2.543965154 | 0.03359785 |
| AC013643.3 | 1.667467917 | 0.033709268 |
| EIF5A2P1 | 1.118253336 | 0.033717041 |
| AC011899.2 | -2.841496195 | 0.033724266 |
| C1GALT1P2 | -5.023524079 | 0.033818607 |
| AP001610.1 | -2.204499848 | 0.033855784 |
| AC016251.1 | 1.356414785 | 0.03410165 |
| AL451069.1 | 2.596920211 | 0.034106027 |
| IGHG1 | 2.511706624 | 0.034455491 |
| AC018761.3 | -1.811302577 | 0.034600687 |
| KDELC1P1 | 1.000934636 | 0.03481882 |
| AC007390.2 | 1.129033133 | 0.03482557 |
| AP000866.5 | -1.25010329 | 0.035087196 |
| AC073878.1 | 3.280196095 | 0.03509422 |
| AC084398.2 | -3.318688712 | 0.035174114 |
| AC245884.11 | -3.592280821 | 0.035220931 |
| AC011472.2 | -1.961189076 | 0.035411119 |
| MKRN5P | 1.189734048 | 0.035591869 |
| AC006033.2 | -2.068102831 | 0.035716401 |
| AL583856.2 | 1.853440386 | 0.035985157 |
| PDCD6IPP1 | 2.881503526 | 0.036184276 |
| DOCK9-DT | 1.462761659 | 0.036200062 |
| SLC26A4-AS1 | 2.501403119 | 0.036261421 |
| AC004490.1 | -1.173197323 | 0.03641665 |
| TRGV8 | -2.914093476 | 0.036476941 |
| RPS3AP37 | 1.801372925 | 0.036663717 |
| AC016708.1 | 1.639817956 | 0.037113222 |
| RPL14P1 | 3.276488702 | 0.037282634 |
| LINC01876 | 2.07322538 | 0.037292442 |
| AF064858.2 | -1.602358995 | 0.037400868 |
| ARHGAP5-AS1 | 1.231702343 | 0.037502373 |
| AC105942.1 | 1.593460656 | 0.037550013 |
| AC008514.1 | 1.233320575 | 0.037878538 |
| AC024337.2 | 1.681161603 | 0.03803297 |
| LINC02126 | 1.940914998 | 0.038143519 |
| OR7E140P | -3.27602547 | 0.038302212 |
| RARA-AS1 | -1.890762993 | 0.038304376 |
| SUGT1P1 | 1.303537359 | 0.03835256 |
| U62631.1 | -2.431044696 | 0.038517623 |
| AL359740.1 | 1.445483825 | 0.038674063 |
| AL136131.3 | -1.903663536 | 0.038689155 |
| TVP23CP2 | 1.193569502 | 0.038711741 |
| AC025280.2 | 4.261165454 | 0.039096279 |
| AC027335.2 | 1.862087965 | 0.039354566 |
| AC066613.1 | -3.474913881 | 0.039431143 |
| AC253576.2 | 2.333286887 | 0.039608027 |
| AL513217.1 | 2.073356234 | 0.03963639 |
| AL031600.3 | -1.315260407 | 0.039636863 |
| AC007262.2 | 2.041941566 | 0.039702508 |
| MACROD2-IT1 | 2.265190089 | 0.039745841 |
| LINC00506 | 1.526440122 | 0.040167596 |
| AP003068.3 | -1.476009553 | 0.040304233 |
| AC007032.1 | -3.446410396 | 0.040331988 |
| SSXP10 | 2.637335151 | 0.040410515 |
| DNAJC27-AS1 | 1.478560569 | 0.040770812 |
| AC009495.1 | -1.487589669 | 0.040821 |
| LINC02596 | -4.284406366 | 0.04094429 |
| LINC01136 | -3.116263695 | 0.04096168 |
| AC105074.1 | 1.13047639 | 0.041165657 |
| VIM-AS1 | -1.121028026 | 0.041466051 |
| AC008870.3 | -2.552186132 | 0.041523458 |
| AC010761.5 | -1.330055927 | 0.041598284 |
| KRTAP5-AS1 | -1.013573588 | 0.041818761 |
| AP002812.3 | 1.421717551 | 0.041911375 |
| LRRFIP1P1 | 1.020905265 | 0.041992792 |
| AC097460.3 | 1.675598793 | 0.042143546 |
| AC003973.3 | 1.359898836 | 0.042532009 |
| GTF2IP7 | 3.025539837 | 0.04261935 |
| C3orf86 | -2.629133673 | 0.04263567 |
| AC008105.2 | -2.044776237 | 0.042722306 |
| AC011676.5 | -1.730355588 | 0.042723655 |
| AC011365.1 | 2.041536316 | 0.042744227 |
| BX322234.1 | -1.312565933 | 0.042751108 |
| AC098869.2 | -2.113732055 | 0.043019082 |
| AC004988.1 | -4.919589193 | 0.04306904 |
| RPS10P2 | 2.705805002 | 0.043533115 |
| AC013643.2 | 1.584598593 | 0.043746603 |
| AL122035.1 | 1.036905616 | 0.04379469 |
| ANKRD20A18P | 1.399728786 | 0.04401251 |
| LINC00886 | 1.725487758 | 0.044117543 |
| AL356599.1 | 1.018933196 | 0.044240106 |
| AC079414.3 | 1.349643396 | 0.04441901 |
| AL355483.1 | -2.138509607 | 0.044431262 |
| ATE1-AS1 | 2.192641222 | 0.044671062 |
| AC008892.1 | -3.803297751 | 0.044877163 |
| AL035461.2 | -1.265019188 | 0.044925758 |
| AC108734.3 | 2.838950987 | 0.045159874 |
| AC007681.1 | 1.227343938 | 0.045212276 |
| LINC00664 | -2.608414549 | 0.045237144 |
| KCNJ2-AS1 | -2.312760124 | 0.0452593 |
| AL359715.3 | 1.482558104 | 0.045304176 |
| AL096701.3 | 1.182347703 | 0.045317876 |
| PABPC1P4 | 1.784885182 | 0.045495536 |
| AC018809.2 | 1.056980636 | 0.045621892 |
| AL121899.1 | -2.278647793 | 0.0456821 |
| AC130456.3 | -3.488292204 | 0.045910414 |
| AL627309.7 | -3.002966734 | 0.045962037 |
| FAM213AP2 | 1.529266245 | 0.046092849 |
| AC026124.1 | 1.604065515 | 0.046282443 |
| ST20-AS1 | -1.082049384 | 0.04637705 |
| AL135910.1 | 2.046281672 | 0.046467364 |
| AC024909.2 | -1.433212965 | 0.046487721 |
| AC096708.3 | 1.349185398 | 0.046696543 |
| AP003071.3 | 1.677444689 | 0.046732506 |
| LINC01016 | 3.813509772 | 0.046811989 |
| AC073288.2 | -1.291220883 | 0.047021668 |
| AC008393.1 | 1.332979173 | 0.047028368 |
| AC004982.1 | 1.49839708 | 0.047334575 |
| TAS2R63P | 1.646909515 | 0.047573601 |
| IGLV3-1 | 5.761533629 | 0.047723429 |
| AC114728.1 | 1.53013581 | 0.047732721 |
| AC010306.1 | 1.334049499 | 0.048409568 |
| AC027288.3 | 1.08020818 | 0.048988694 |
| AP000857.1 | 2.03646521 | 0.049067898 |
| LINC01798 | 1.145584998 | 0.049235755 |
| AC023813.1 | -5.23720782 | 0.049278774 |
| AC116903.1 | -2.021311811 | 0.049325025 |
| AC074050.3 | -1.538997246 | 0.049454884 |
| AC092053.3 | -2.393197331 | 0.049538245 |
| AC010185.1 | 1.546387318 | 0.049729825 |
| AP001453.2 | -1.182995988 | 0.049986843 |
| AC012368.2 | -2.113018224 | 0.049993174 |
