# Supplementary material for: Identification and validation of long non-coding RNA associated ceRNAs in intrauterine adhesion
Source: Bioengineered. 2021 Dec 30;13(1):1039–48. doi: 10.1080/21655979.2021.2017578 (PMC8805920; doi:10.1080/21655979.2021.2017578)
Supplement: Supplemental Material [file KBIE_A_2017578_SM8799.zip › supplementary/Supplementary Table 3 ceRNA.docx]

**Supplementary Table 3. The integrated lncRNA-miRNA-mRNA interaction**

| **lncRNA** | **miRNA** | **mRNA** |
| --- | --- | --- |
| AC095050.1 | hsa-miR-25-3p | INMT |
| AC095050.1 | hsa-miR-25-3p | REEP1 |
| AC095050.1 | hsa-miR-25-3p | CCDC80 |
| AC095050.1 | hsa-miR-25-3p | HOXA13 |
| AC095050.1 | hsa-miR-25-3p | HLF |
| AC095050.1 | hsa-miR-25-3p | NRK |
| AC095050.1 | hsa-miR-25-3p | PTGIS |
| AC095050.1 | hsa-miR-25-3p | ATP1B2 |
| AC095050.1 | hsa-miR-25-3p | MATN2 |
| AC095050.1 | hsa-miR-25-3p | PI15 |
| AC095050.1 | hsa-miR-25-3p | PDE8B |
| AC095050.1 | hsa-miR-25-3p | OPRK1 |
| AC095050.1 | hsa-miR-25-3p | KCNE4 |
| AC095050.1 | hsa-miR-25-3p | RAB3C |
| AC095050.1 | hsa-miR-25-3p | ACTC1 |
| AC095050.1 | hsa-miR-25-3p | EMCN |
| AC095050.1 | hsa-miR-25-3p | FAT3 |
| AC095050.1 | hsa-miR-25-3p | PRRT2 |
| AC095050.1 | hsa-miR-25-3p | KCNK3 |
| AC095050.1 | hsa-miR-25-3p | SLC38A11 |
| AC095050.1 | hsa-miR-25-3p | GPR37 |
| AC095050.1 | hsa-miR-25-3p | CPLX1 |
| AC095050.1 | hsa-miR-25-3p | GPR22 |
| AC095050.1 | hsa-miR-25-3p | ADH1B |
| AC095050.1 | hsa-miR-25-3p | TUB |
| AC095050.1 | hsa-miR-25-3p | MSRB3 |
| AC095050.1 | hsa-miR-25-3p | LPP |
| AC095050.1 | hsa-miR-25-3p | CLU |
| AC095050.1 | hsa-miR-25-3p | PLAG1 |
| AC095050.1 | hsa-miR-25-3p | LURAP1L |
| AC095050.1 | hsa-miR-25-3p | KIAA1549L |
| AC095050.1 | hsa-miR-25-3p | C11orf87 |
| AC095050.1 | hsa-miR-25-3p | TNFAIP8L3 |
| AC095050.1 | hsa-miR-25-3p | BTNL9 |
| AC095050.1 | hsa-miR-25-3p | COL4A6 |
| AC095050.1 | hsa-miR-25-3p | TSPAN18 |
| AC095050.1 | hsa-miR-25-3p | SLC24A2 |
| AC095050.1 | hsa-miR-25-3p | NR3C2 |
| AC095050.1 | hsa-miR-25-3p | CAMK2A |
| AC095050.1 | hsa-miR-25-3p | DCX |
| AC095050.1 | hsa-miR-25-3p | CACNA1H |
| AC095050.1 | hsa-miR-25-3p | PHACTR3 |
| AC095050.1 | hsa-miR-25-3p | NFIX |
| AC095050.1 | hsa-miR-25-3p | MYLK |
| AC095050.1 | hsa-miR-25-3p | ZNF521 |
| AC095050.1 | hsa-miR-25-3p | CNR1 |
| AC095050.1 | hsa-miR-25-3p | GPR161 |
| AC095050.1 | hsa-miR-25-3p | RBM20 |
| AC095050.1 | hsa-miR-25-3p | BEND6 |
| AC095050.1 | hsa-miR-25-3p | PTGFR |
| AC095050.1 | hsa-miR-25-3p | LRRC1 |
| AC095050.1 | hsa-miR-25-3p | PTGER3 |
| AC095050.1 | hsa-miR-25-3p | SORBS1 |
| AC095050.1 | hsa-miR-25-3p | KCNB1 |
| AC095050.1 | hsa-miR-25-3p | RAB40A |
| AC095050.1 | hsa-miR-25-3p | JPH2 |
| AC095050.1 | hsa-miR-25-3p | TRPM3 |
| AC095050.1 | hsa-miR-25-3p | PTCHD1 |
| AC095050.1 | hsa-miR-25-3p | BNC2 |
| AC095050.1 | hsa-miR-25-3p | LGI2 |
| AC095050.1 | hsa-miR-25-3p | RYR3 |
| AC095050.1 | hsa-miR-25-3p | CDON |
| AC095050.1 | hsa-miR-25-3p | NT5DC3 |
| AC095050.1 | hsa-miR-25-3p | KALRN |
| AC095050.1 | hsa-miR-25-3p | SPOCK1 |
| AC095050.1 | hsa-miR-25-3p | ST8SIA1 |
| AC095050.1 | hsa-miR-25-3p | ZNF208 |
| AC095050.1 | hsa-miR-25-3p | SRL |
| AC095050.1 | hsa-miR-25-3p | ACTG2 |
| AC095050.1 | hsa-miR-25-3p | ADAMTSL5 |
| AC095050.1 | hsa-miR-25-3p | HMCN2 |
| AC095050.1 | hsa-miR-25-3p | SYNPO2 |
| AC095050.1 | hsa-miR-25-3p | GFRA1 |
| AC095050.1 | hsa-miR-25-3p | TCEA3 |
| AC095050.1 | hsa-miR-25-3p | KLF8 |
| AC095050.1 | hsa-miR-25-3p | ABI3BP |
| AC095050.1 | hsa-miR-25-3p | ST6GALNAC5 |
| AC095050.1 | hsa-miR-25-3p | PCOLCE2 |
| AC095050.1 | hsa-miR-25-3p | SLC15A2 |
| AC095050.1 | hsa-miR-25-3p | MEGF10 |
| AC095050.1 | hsa-miR-25-3p | TMEM200B |
| AC095050.1 | hsa-miR-25-3p | RANBP17 |
| AC095050.1 | hsa-miR-25-3p | NAALAD2 |
| AC095050.1 | hsa-miR-25-3p | CES4A |
| AC095050.1 | hsa-miR-25-3p | AOC3 |
| LINC00632 | hsa-miR-93-5p | TLL1 |
| LINC00632 | hsa-miR-93-5p | CCDC80 |
| LINC00632 | hsa-miR-93-5p | HOXA13 |
| LINC00632 | hsa-miR-93-5p | HLF |
| LINC00632 | hsa-miR-93-5p | IGFBP5 |
| LINC00632 | hsa-miR-93-5p | PTGIS |
| LINC00632 | hsa-miR-93-5p | CNN1 |
| LINC00632 | hsa-miR-93-5p | TEX15 |
| LINC00632 | hsa-miR-93-5p | PI15 |
| LINC00632 | hsa-miR-93-5p | ASPA |
| LINC00632 | hsa-miR-93-5p | ASXL3 |
| LINC00632 | hsa-miR-93-5p | OSR1 |
| LINC00632 | hsa-miR-93-5p | TRPC1 |
| LINC00632 | hsa-miR-93-5p | SLC2A12 |
| LINC00632 | hsa-miR-93-5p | TMEM47 |
| LINC00632 | hsa-miR-93-5p | KCNE4 |
| LINC00632 | hsa-miR-93-5p | PDLIM3 |
| LINC00632 | hsa-miR-93-5p | UNC5D |
| LINC00632 | hsa-miR-93-5p | FBXO27 |
| LINC00632 | hsa-miR-93-5p | TCF23 |
| LINC00632 | hsa-miR-93-5p | EMCN |
| LINC00632 | hsa-miR-93-5p | HPGD |
| LINC00632 | hsa-miR-93-5p | NPY1R |
| LINC00632 | hsa-miR-93-5p | FAT3 |
| LINC00632 | hsa-miR-93-5p | TMEM132B |
| LINC00632 | hsa-miR-93-5p | GPR37 |
| LINC00632 | hsa-miR-93-5p | GPR22 |
| LINC00632 | hsa-miR-93-5p | ADH1B |
| LINC00632 | hsa-miR-93-5p | MSRB3 |
| LINC00632 | hsa-miR-93-5p | LPP |
| LINC00632 | hsa-miR-93-5p | BVES |
| LINC00632 | hsa-miR-93-5p | PLAG1 |
| LINC00632 | hsa-miR-93-5p | SLC2A4 |
| LINC00632 | hsa-miR-93-5p | FIBIN |
| LINC00632 | hsa-miR-93-5p | KIAA1549L |
| LINC00632 | hsa-miR-93-5p | C3orf80 |
| LINC00632 | hsa-miR-93-5p | C11orf87 |
| LINC00632 | hsa-miR-93-5p | TNFAIP8L3 |
| LINC00632 | hsa-miR-93-5p | ST6GALNAC3 |
| LINC00632 | hsa-miR-93-5p | C3orf70 |
| LINC00632 | hsa-miR-93-5p | SLC24A2 |
| LINC00632 | hsa-miR-93-5p | PRELP |
| LINC00632 | hsa-miR-93-5p | ATP2B2 |
| LINC00632 | hsa-miR-93-5p | SMOC2 |
| LINC00632 | hsa-miR-93-5p | MYOM1 |
| LINC00632 | hsa-miR-93-5p | ADAM33 |
| LINC00632 | hsa-miR-93-5p | DCX |
| LINC00632 | hsa-miR-93-5p | ANKRD36 |
| LINC00632 | hsa-miR-93-5p | ZNF483 |
| LINC00632 | hsa-miR-93-5p | MFAP5 |
| LINC00632 | hsa-miR-93-5p | MAP2 |
| LINC00632 | hsa-miR-93-5p | FBXL22 |
| LINC00632 | hsa-miR-93-5p | MYLK |
| LINC00632 | hsa-miR-93-5p | CNR1 |
| LINC00632 | hsa-miR-93-5p | DNM3 |
| LINC00632 | hsa-miR-93-5p | GPR161 |
| LINC00632 | hsa-miR-93-5p | ITGA10 |
| LINC00632 | hsa-miR-93-5p | RBM20 |
| LINC00632 | hsa-miR-93-5p | SYPL2 |
| LINC00632 | hsa-miR-93-5p | CDR1 |
| LINC00632 | hsa-miR-93-5p | PTGER3 |
| LINC00632 | hsa-miR-93-5p | SLC25A27 |
| LINC00632 | hsa-miR-93-5p | KCNB1 |
| LINC00632 | hsa-miR-93-5p | PAK3 |
| LINC00632 | hsa-miR-93-5p | IRS4 |
| LINC00632 | hsa-miR-93-5p | SLC6A9 |
| LINC00632 | hsa-miR-93-5p | WISP2 |
| LINC00632 | hsa-miR-93-5p | PRKG1 |
| LINC00632 | hsa-miR-93-5p | TRPM3 |
| LINC00632 | hsa-miR-93-5p | MAMDC2 |
| LINC00632 | hsa-miR-93-5p | PKHD1L1 |
| LINC00632 | hsa-miR-93-5p | PTCHD1 |
| LINC00632 | hsa-miR-93-5p | DCLK1 |
| LINC00632 | hsa-miR-93-5p | BNC2 |
| LINC00632 | hsa-miR-93-5p | SMOC1 |
| LINC00632 | hsa-miR-93-5p | CYS1 |
| LINC00632 | hsa-miR-93-5p | LGI2 |
| LINC00632 | hsa-miR-93-5p | SEMA5A |
| LINC00632 | hsa-miR-93-5p | SLC22A3 |
| LINC00632 | hsa-miR-93-5p | NT5DC3 |
| LINC00632 | hsa-miR-93-5p | ANKRD39 |
| LINC00632 | hsa-miR-93-5p | SPOCK1 |
| LINC00632 | hsa-miR-93-5p | FAM13A |
| LINC00632 | hsa-miR-93-5p | MAPK10 |
| LINC00632 | hsa-miR-93-5p | CDKL1 |
| LINC00632 | hsa-miR-93-5p | KCNG1 |
| LINC00632 | hsa-miR-93-5p | ST8SIA1 |
| LINC00632 | hsa-miR-93-5p | PGM5 |
| LINC00632 | hsa-miR-93-5p | RIC3 |
| LINC00632 | hsa-miR-93-5p | DGKB |
| LINC00632 | hsa-miR-93-5p | ADAMTSL5 |
| LINC00632 | hsa-miR-93-5p | MRVI1 |
| LINC00632 | hsa-miR-93-5p | SYNPO2 |
| LINC00632 | hsa-miR-93-5p | GFRA1 |
| LINC00632 | hsa-miR-93-5p | SHISA6 |
| LINC00632 | hsa-miR-93-5p | KLF8 |
| LINC00632 | hsa-miR-93-5p | FSBP |
| LINC00632 | hsa-miR-93-5p | SLC15A2 |
| LINC00632 | hsa-miR-93-5p | TMEM158 |
| LINC00632 | hsa-miR-93-5p | SORCS2 |
| LINC00632 | hsa-miR-93-5p | YPEL4 |
| LINC00632 | hsa-miR-93-5p | SYBU |
| LINC00632 | hsa-miR-93-5p | NAALAD2 |
| LINC00632 | hsa-miR-93-5p | PIANP |
| LINC00632 | hsa-miR-93-5p | FXYD6 |
| LINC00632 | hsa-miR-93-5p | FHL5 |
| LINC00632 | hsa-miR-93-5p | HSD17B6 |
| LINC00632 | hsa-miR-93-5p | EML5 |
| LINC00632 | hsa-miR-93-5p | CCDC68 |
| LINC00632 | hsa-miR-93-5p | PPP1R12B |
| MBNL1-AS1 | hsa-miR-93-5p | TLL1 |
| MBNL1-AS1 | hsa-miR-93-5p | CCDC80 |
| MBNL1-AS1 | hsa-miR-93-5p | HOXA13 |
| MBNL1-AS1 | hsa-miR-93-5p | HLF |
| MBNL1-AS1 | hsa-miR-93-5p | IGFBP5 |
| MBNL1-AS1 | hsa-miR-93-5p | PTGIS |
| MBNL1-AS1 | hsa-miR-93-5p | CNN1 |
| MBNL1-AS1 | hsa-miR-93-5p | TEX15 |
| MBNL1-AS1 | hsa-miR-93-5p | PI15 |
| MBNL1-AS1 | hsa-miR-93-5p | ASPA |
| MBNL1-AS1 | hsa-miR-93-5p | ASXL3 |
| MBNL1-AS1 | hsa-miR-93-5p | OSR1 |
| MBNL1-AS1 | hsa-miR-93-5p | TRPC1 |
| MBNL1-AS1 | hsa-miR-93-5p | SLC2A12 |
| MBNL1-AS1 | hsa-miR-93-5p | TMEM47 |
| MBNL1-AS1 | hsa-miR-93-5p | KCNE4 |
| MBNL1-AS1 | hsa-miR-93-5p | PDLIM3 |
| MBNL1-AS1 | hsa-miR-93-5p | UNC5D |
| MBNL1-AS1 | hsa-miR-93-5p | FBXO27 |
| MBNL1-AS1 | hsa-miR-93-5p | TCF23 |
| MBNL1-AS1 | hsa-miR-93-5p | EMCN |
| MBNL1-AS1 | hsa-miR-93-5p | HPGD |
| MBNL1-AS1 | hsa-miR-93-5p | NPY1R |
| MBNL1-AS1 | hsa-miR-93-5p | FAT3 |
| MBNL1-AS1 | hsa-miR-93-5p | TMEM132B |
| MBNL1-AS1 | hsa-miR-93-5p | GPR37 |
| MBNL1-AS1 | hsa-miR-93-5p | GPR22 |
| MBNL1-AS1 | hsa-miR-93-5p | ADH1B |
| MBNL1-AS1 | hsa-miR-93-5p | MSRB3 |
| MBNL1-AS1 | hsa-miR-93-5p | LPP |
| MBNL1-AS1 | hsa-miR-93-5p | BVES |
| MBNL1-AS1 | hsa-miR-93-5p | PLAG1 |
| MBNL1-AS1 | hsa-miR-93-5p | SLC2A4 |
| MBNL1-AS1 | hsa-miR-93-5p | FIBIN |
| MBNL1-AS1 | hsa-miR-93-5p | KIAA1549L |
| MBNL1-AS1 | hsa-miR-93-5p | C3orf80 |
| MBNL1-AS1 | hsa-miR-93-5p | C11orf87 |
| MBNL1-AS1 | hsa-miR-93-5p | TNFAIP8L3 |
| MBNL1-AS1 | hsa-miR-93-5p | ST6GALNAC3 |
| MBNL1-AS1 | hsa-miR-93-5p | C3orf70 |
| MBNL1-AS1 | hsa-miR-93-5p | SLC24A2 |
| MBNL1-AS1 | hsa-miR-93-5p | PRELP |
| MBNL1-AS1 | hsa-miR-93-5p | ATP2B2 |
| MBNL1-AS1 | hsa-miR-93-5p | SMOC2 |
| MBNL1-AS1 | hsa-miR-93-5p | MYOM1 |
| MBNL1-AS1 | hsa-miR-93-5p | ADAM33 |
| MBNL1-AS1 | hsa-miR-93-5p | DCX |
| MBNL1-AS1 | hsa-miR-93-5p | ANKRD36 |
| MBNL1-AS1 | hsa-miR-93-5p | ZNF483 |
| MBNL1-AS1 | hsa-miR-93-5p | MFAP5 |
| MBNL1-AS1 | hsa-miR-93-5p | MAP2 |
| MBNL1-AS1 | hsa-miR-93-5p | FBXL22 |
| MBNL1-AS1 | hsa-miR-93-5p | MYLK |
| MBNL1-AS1 | hsa-miR-93-5p | CNR1 |
| MBNL1-AS1 | hsa-miR-93-5p | DNM3 |
| MBNL1-AS1 | hsa-miR-93-5p | GPR161 |
| MBNL1-AS1 | hsa-miR-93-5p | ITGA10 |
| MBNL1-AS1 | hsa-miR-93-5p | RBM20 |
| MBNL1-AS1 | hsa-miR-93-5p | SYPL2 |
| MBNL1-AS1 | hsa-miR-93-5p | CDR1 |
| MBNL1-AS1 | hsa-miR-93-5p | PTGER3 |
| MBNL1-AS1 | hsa-miR-93-5p | SLC25A27 |
| MBNL1-AS1 | hsa-miR-93-5p | KCNB1 |
| MBNL1-AS1 | hsa-miR-93-5p | PAK3 |
| MBNL1-AS1 | hsa-miR-93-5p | IRS4 |
| MBNL1-AS1 | hsa-miR-93-5p | SLC6A9 |
| MBNL1-AS1 | hsa-miR-93-5p | WISP2 |
| MBNL1-AS1 | hsa-miR-93-5p | PRKG1 |
| MBNL1-AS1 | hsa-miR-93-5p | TRPM3 |
| MBNL1-AS1 | hsa-miR-93-5p | MAMDC2 |
| MBNL1-AS1 | hsa-miR-93-5p | PKHD1L1 |
| MBNL1-AS1 | hsa-miR-93-5p | PTCHD1 |
| MBNL1-AS1 | hsa-miR-93-5p | DCLK1 |
| MBNL1-AS1 | hsa-miR-93-5p | BNC2 |
| MBNL1-AS1 | hsa-miR-93-5p | SMOC1 |
| MBNL1-AS1 | hsa-miR-93-5p | CYS1 |
| MBNL1-AS1 | hsa-miR-93-5p | LGI2 |
| MBNL1-AS1 | hsa-miR-93-5p | SEMA5A |
| MBNL1-AS1 | hsa-miR-93-5p | SLC22A3 |
| MBNL1-AS1 | hsa-miR-93-5p | NT5DC3 |
| MBNL1-AS1 | hsa-miR-93-5p | ANKRD39 |
| MBNL1-AS1 | hsa-miR-93-5p | SPOCK1 |
| MBNL1-AS1 | hsa-miR-93-5p | FAM13A |
| MBNL1-AS1 | hsa-miR-93-5p | MAPK10 |
| MBNL1-AS1 | hsa-miR-93-5p | CDKL1 |
| MBNL1-AS1 | hsa-miR-93-5p | KCNG1 |
| MBNL1-AS1 | hsa-miR-93-5p | ST8SIA1 |
| MBNL1-AS1 | hsa-miR-93-5p | PGM5 |
| MBNL1-AS1 | hsa-miR-93-5p | RIC3 |
| MBNL1-AS1 | hsa-miR-93-5p | DGKB |
| MBNL1-AS1 | hsa-miR-93-5p | ADAMTSL5 |
| MBNL1-AS1 | hsa-miR-93-5p | MRVI1 |
| MBNL1-AS1 | hsa-miR-93-5p | SYNPO2 |
| MBNL1-AS1 | hsa-miR-93-5p | GFRA1 |
| MBNL1-AS1 | hsa-miR-93-5p | SHISA6 |
| MBNL1-AS1 | hsa-miR-93-5p | KLF8 |
| MBNL1-AS1 | hsa-miR-93-5p | FSBP |
| MBNL1-AS1 | hsa-miR-93-5p | SLC15A2 |
| MBNL1-AS1 | hsa-miR-93-5p | TMEM158 |
| MBNL1-AS1 | hsa-miR-93-5p | SORCS2 |
| MBNL1-AS1 | hsa-miR-93-5p | YPEL4 |
| MBNL1-AS1 | hsa-miR-93-5p | SYBU |
| MBNL1-AS1 | hsa-miR-93-5p | NAALAD2 |
| MBNL1-AS1 | hsa-miR-93-5p | PIANP |
| MBNL1-AS1 | hsa-miR-93-5p | FXYD6 |
| MBNL1-AS1 | hsa-miR-93-5p | FHL5 |
| MBNL1-AS1 | hsa-miR-93-5p | HSD17B6 |
| MBNL1-AS1 | hsa-miR-93-5p | EML5 |
| MBNL1-AS1 | hsa-miR-93-5p | CCDC68 |
| MBNL1-AS1 | hsa-miR-93-5p | PPP1R12B |
| AF001548.3 | hsa-miR-93-5p | TLL1 |
| AF001548.3 | hsa-miR-93-5p | CCDC80 |
| AF001548.3 | hsa-miR-93-5p | HOXA13 |
| AF001548.3 | hsa-miR-93-5p | HLF |
| AF001548.3 | hsa-miR-93-5p | IGFBP5 |
| AF001548.3 | hsa-miR-93-5p | PTGIS |
| AF001548.3 | hsa-miR-93-5p | CNN1 |
| AF001548.3 | hsa-miR-93-5p | TEX15 |
| AF001548.3 | hsa-miR-93-5p | PI15 |
| AF001548.3 | hsa-miR-93-5p | ASPA |
| AF001548.3 | hsa-miR-93-5p | ASXL3 |
| AF001548.3 | hsa-miR-93-5p | OSR1 |
| AF001548.3 | hsa-miR-93-5p | TRPC1 |
| AF001548.3 | hsa-miR-93-5p | SLC2A12 |
| AF001548.3 | hsa-miR-93-5p | TMEM47 |
| AF001548.3 | hsa-miR-93-5p | KCNE4 |
| AF001548.3 | hsa-miR-93-5p | PDLIM3 |
| AF001548.3 | hsa-miR-93-5p | UNC5D |
| AF001548.3 | hsa-miR-93-5p | FBXO27 |
| AF001548.3 | hsa-miR-93-5p | TCF23 |
| AF001548.3 | hsa-miR-93-5p | EMCN |
| AF001548.3 | hsa-miR-93-5p | HPGD |
| AF001548.3 | hsa-miR-93-5p | NPY1R |
| AF001548.3 | hsa-miR-93-5p | FAT3 |
| AF001548.3 | hsa-miR-93-5p | TMEM132B |
| AF001548.3 | hsa-miR-93-5p | GPR37 |
| AF001548.3 | hsa-miR-93-5p | GPR22 |
| AF001548.3 | hsa-miR-93-5p | ADH1B |
| AF001548.3 | hsa-miR-93-5p | MSRB3 |
| AF001548.3 | hsa-miR-93-5p | LPP |
| AF001548.3 | hsa-miR-93-5p | BVES |
| AF001548.3 | hsa-miR-93-5p | PLAG1 |
| AF001548.3 | hsa-miR-93-5p | SLC2A4 |
| AF001548.3 | hsa-miR-93-5p | FIBIN |
| AF001548.3 | hsa-miR-93-5p | KIAA1549L |
| AF001548.3 | hsa-miR-93-5p | C3orf80 |
| AF001548.3 | hsa-miR-93-5p | C11orf87 |
| AF001548.3 | hsa-miR-93-5p | TNFAIP8L3 |
| AF001548.3 | hsa-miR-93-5p | ST6GALNAC3 |
| AF001548.3 | hsa-miR-93-5p | C3orf70 |
| AF001548.3 | hsa-miR-93-5p | SLC24A2 |
| AF001548.3 | hsa-miR-93-5p | PRELP |
| AF001548.3 | hsa-miR-93-5p | ATP2B2 |
| AF001548.3 | hsa-miR-93-5p | SMOC2 |
| AF001548.3 | hsa-miR-93-5p | MYOM1 |
| AF001548.3 | hsa-miR-93-5p | ADAM33 |
| AF001548.3 | hsa-miR-93-5p | DCX |
| AF001548.3 | hsa-miR-93-5p | ANKRD36 |
| AF001548.3 | hsa-miR-93-5p | ZNF483 |
| AF001548.3 | hsa-miR-93-5p | MFAP5 |
| AF001548.3 | hsa-miR-93-5p | MAP2 |
| AF001548.3 | hsa-miR-93-5p | FBXL22 |
| AF001548.3 | hsa-miR-93-5p | MYLK |
| AF001548.3 | hsa-miR-93-5p | CNR1 |
| AF001548.3 | hsa-miR-93-5p | DNM3 |
| AF001548.3 | hsa-miR-93-5p | GPR161 |
| AF001548.3 | hsa-miR-93-5p | ITGA10 |
| AF001548.3 | hsa-miR-93-5p | RBM20 |
| AF001548.3 | hsa-miR-93-5p | SYPL2 |
| AF001548.3 | hsa-miR-93-5p | CDR1 |
| AF001548.3 | hsa-miR-93-5p | PTGER3 |
| AF001548.3 | hsa-miR-93-5p | SLC25A27 |
| AF001548.3 | hsa-miR-93-5p | KCNB1 |
| AF001548.3 | hsa-miR-93-5p | PAK3 |
| AF001548.3 | hsa-miR-93-5p | IRS4 |
| AF001548.3 | hsa-miR-93-5p | SLC6A9 |
| AF001548.3 | hsa-miR-93-5p | WISP2 |
| AF001548.3 | hsa-miR-93-5p | PRKG1 |
| AF001548.3 | hsa-miR-93-5p | TRPM3 |
| AF001548.3 | hsa-miR-93-5p | MAMDC2 |
| AF001548.3 | hsa-miR-93-5p | PKHD1L1 |
| AF001548.3 | hsa-miR-93-5p | PTCHD1 |
| AF001548.3 | hsa-miR-93-5p | DCLK1 |
| AF001548.3 | hsa-miR-93-5p | BNC2 |
| AF001548.3 | hsa-miR-93-5p | SMOC1 |
| AF001548.3 | hsa-miR-93-5p | CYS1 |
| AF001548.3 | hsa-miR-93-5p | LGI2 |
| AF001548.3 | hsa-miR-93-5p | SEMA5A |
| AF001548.3 | hsa-miR-93-5p | SLC22A3 |
| AF001548.3 | hsa-miR-93-5p | NT5DC3 |
| AF001548.3 | hsa-miR-93-5p | ANKRD39 |
| AF001548.3 | hsa-miR-93-5p | SPOCK1 |
| AF001548.3 | hsa-miR-93-5p | FAM13A |
| AF001548.3 | hsa-miR-93-5p | MAPK10 |
| AF001548.3 | hsa-miR-93-5p | CDKL1 |
| AF001548.3 | hsa-miR-93-5p | KCNG1 |
| AF001548.3 | hsa-miR-93-5p | ST8SIA1 |
| AF001548.3 | hsa-miR-93-5p | PGM5 |
| AF001548.3 | hsa-miR-93-5p | RIC3 |
| AF001548.3 | hsa-miR-93-5p | DGKB |
| AF001548.3 | hsa-miR-93-5p | ADAMTSL5 |
| AF001548.3 | hsa-miR-93-5p | MRVI1 |
| AF001548.3 | hsa-miR-93-5p | SYNPO2 |
| AF001548.3 | hsa-miR-93-5p | GFRA1 |
| AF001548.3 | hsa-miR-93-5p | SHISA6 |
| AF001548.3 | hsa-miR-93-5p | KLF8 |
| AF001548.3 | hsa-miR-93-5p | FSBP |
| AF001548.3 | hsa-miR-93-5p | SLC15A2 |
| AF001548.3 | hsa-miR-93-5p | TMEM158 |
| AF001548.3 | hsa-miR-93-5p | SORCS2 |
| AF001548.3 | hsa-miR-93-5p | YPEL4 |
| AF001548.3 | hsa-miR-93-5p | SYBU |
| AF001548.3 | hsa-miR-93-5p | NAALAD2 |
| AF001548.3 | hsa-miR-93-5p | PIANP |
| AF001548.3 | hsa-miR-93-5p | FXYD6 |
| AF001548.3 | hsa-miR-93-5p | FHL5 |
| AF001548.3 | hsa-miR-93-5p | HSD17B6 |
| AF001548.3 | hsa-miR-93-5p | EML5 |
| AF001548.3 | hsa-miR-93-5p | CCDC68 |
| AF001548.3 | hsa-miR-93-5p | PPP1R12B |
| LINC01482 | hsa-miR-92b-3p | INMT |
| LINC01482 | hsa-miR-92b-3p | REEP1 |
| LINC01482 | hsa-miR-92b-3p | CCDC80 |
| LINC01482 | hsa-miR-92b-3p | HOXA13 |
| LINC01482 | hsa-miR-92b-3p | HLF |
| LINC01482 | hsa-miR-92b-3p | NRK |
| LINC01482 | hsa-miR-92b-3p | PTGIS |
| LINC01482 | hsa-miR-92b-3p | ATP1B2 |
| LINC01482 | hsa-miR-92b-3p | MATN2 |
| LINC01482 | hsa-miR-92b-3p | PI15 |
| LINC01482 | hsa-miR-92b-3p | PDE8B |
| LINC01482 | hsa-miR-92b-3p | OPRK1 |
| LINC01482 | hsa-miR-92b-3p | KCNE4 |
| LINC01482 | hsa-miR-92b-3p | RAB3C |
| LINC01482 | hsa-miR-92b-3p | ACTC1 |
| LINC01482 | hsa-miR-92b-3p | EMCN |
| LINC01482 | hsa-miR-92b-3p | FAT3 |
| LINC01482 | hsa-miR-92b-3p | PRRT2 |
| LINC01482 | hsa-miR-92b-3p | KCNK3 |
| LINC01482 | hsa-miR-92b-3p | SLC38A11 |
| LINC01482 | hsa-miR-92b-3p | GPR37 |
| LINC01482 | hsa-miR-92b-3p | CPLX1 |
| LINC01482 | hsa-miR-92b-3p | GPR22 |
| LINC01482 | hsa-miR-92b-3p | ADH1B |
| LINC01482 | hsa-miR-92b-3p | TUB |
| LINC01482 | hsa-miR-92b-3p | MSRB3 |
| LINC01482 | hsa-miR-92b-3p | LPP |
| LINC01482 | hsa-miR-92b-3p | CLU |
| LINC01482 | hsa-miR-92b-3p | PLAG1 |
| LINC01482 | hsa-miR-92b-3p | LURAP1L |
| LINC01482 | hsa-miR-92b-3p | KIAA1549L |
| LINC01482 | hsa-miR-92b-3p | C11orf87 |
| LINC01482 | hsa-miR-92b-3p | TNFAIP8L3 |
| LINC01482 | hsa-miR-92b-3p | BTNL9 |
| LINC01482 | hsa-miR-92b-3p | COL4A6 |
| LINC01482 | hsa-miR-92b-3p | TSPAN18 |
| LINC01482 | hsa-miR-92b-3p | SLC24A2 |
| LINC01482 | hsa-miR-92b-3p | NR3C2 |
| LINC01482 | hsa-miR-92b-3p | CAMK2A |
| LINC01482 | hsa-miR-92b-3p | DCX |
| LINC01482 | hsa-miR-92b-3p | CACNA1H |
| LINC01482 | hsa-miR-92b-3p | PHACTR3 |
| LINC01482 | hsa-miR-92b-3p | NFIX |
| LINC01482 | hsa-miR-92b-3p | MYLK |
| LINC01482 | hsa-miR-92b-3p | ZNF521 |
| LINC01482 | hsa-miR-92b-3p | CNR1 |
| LINC01482 | hsa-miR-92b-3p | GPR161 |
| LINC01482 | hsa-miR-92b-3p | RBM20 |
| LINC01482 | hsa-miR-92b-3p | BEND6 |
| LINC01482 | hsa-miR-92b-3p | PTGFR |
| LINC01482 | hsa-miR-92b-3p | LRRC1 |
| LINC01482 | hsa-miR-92b-3p | PTGER3 |
| LINC01482 | hsa-miR-92b-3p | SORBS1 |
| LINC01482 | hsa-miR-92b-3p | KCNB1 |
| LINC01482 | hsa-miR-92b-3p | RAB40A |
| LINC01482 | hsa-miR-92b-3p | JPH2 |
| LINC01482 | hsa-miR-92b-3p | TRPM3 |
| LINC01482 | hsa-miR-92b-3p | PTCHD1 |
| LINC01482 | hsa-miR-92b-3p | BNC2 |
| LINC01482 | hsa-miR-92b-3p | LGI2 |
| LINC01482 | hsa-miR-92b-3p | RYR3 |
| LINC01482 | hsa-miR-92b-3p | CDON |
| LINC01482 | hsa-miR-92b-3p | NT5DC3 |
| LINC01482 | hsa-miR-92b-3p | KALRN |
| LINC01482 | hsa-miR-92b-3p | SPOCK1 |
| LINC01482 | hsa-miR-92b-3p | ST8SIA1 |
| LINC01482 | hsa-miR-92b-3p | ZNF208 |
| LINC01482 | hsa-miR-92b-3p | SRL |
| LINC01482 | hsa-miR-92b-3p | ACTG2 |
| LINC01482 | hsa-miR-92b-3p | ADAMTSL5 |
| LINC01482 | hsa-miR-92b-3p | HMCN2 |
| LINC01482 | hsa-miR-92b-3p | SYNPO2 |
| LINC01482 | hsa-miR-92b-3p | GFRA1 |
| LINC01482 | hsa-miR-92b-3p | TCEA3 |
| LINC01482 | hsa-miR-92b-3p | KLF8 |
| LINC01482 | hsa-miR-92b-3p | ABI3BP |
| LINC01482 | hsa-miR-92b-3p | ST6GALNAC5 |
| LINC01482 | hsa-miR-92b-3p | PCOLCE2 |
| LINC01482 | hsa-miR-92b-3p | SLC15A2 |
| LINC01482 | hsa-miR-92b-3p | MEGF10 |
| LINC01482 | hsa-miR-92b-3p | TMEM200B |
| LINC01482 | hsa-miR-92b-3p | RANBP17 |
| LINC01482 | hsa-miR-92b-3p | NAALAD2 |
| LINC01482 | hsa-miR-92b-3p | CES4A |
| LINC01482 | hsa-miR-92b-3p | AOC3 |
| LINC00632 | hsa-miR-199b-5p | INMT |
| LINC00632 | hsa-miR-199b-5p | CCDC80 |
| LINC00632 | hsa-miR-199b-5p | HOXA13 |
| LINC00632 | hsa-miR-199b-5p | HLF |
| LINC00632 | hsa-miR-199b-5p | IGFBP5 |
| LINC00632 | hsa-miR-199b-5p | RAB9B |
| LINC00632 | hsa-miR-199b-5p | PTGIS |
| LINC00632 | hsa-miR-199b-5p | LRRC17 |
| LINC00632 | hsa-miR-199b-5p | CNN1 |
| LINC00632 | hsa-miR-199b-5p | GATA5 |
| LINC00632 | hsa-miR-199b-5p | MATN2 |
| LINC00632 | hsa-miR-199b-5p | PI15 |
| LINC00632 | hsa-miR-199b-5p | ASPA |
| LINC00632 | hsa-miR-199b-5p | OPRK1 |
| LINC00632 | hsa-miR-199b-5p | OSR1 |
| LINC00632 | hsa-miR-199b-5p | SLC2A12 |
| LINC00632 | hsa-miR-199b-5p | TMEM47 |
| LINC00632 | hsa-miR-199b-5p | KCNE4 |
| LINC00632 | hsa-miR-199b-5p | RAB3C |
| LINC00632 | hsa-miR-199b-5p | PDLIM3 |
| LINC00632 | hsa-miR-199b-5p | TCF23 |
| LINC00632 | hsa-miR-199b-5p | FAT3 |
| LINC00632 | hsa-miR-199b-5p | TMEM132B |
| LINC00632 | hsa-miR-199b-5p | MYRIP |
| LINC00632 | hsa-miR-199b-5p | KCNK3 |
| LINC00632 | hsa-miR-199b-5p | GPR37 |
| LINC00632 | hsa-miR-199b-5p | TUB |
| LINC00632 | hsa-miR-199b-5p | C7 |
| LINC00632 | hsa-miR-199b-5p | GPR88 |
| LINC00632 | hsa-miR-199b-5p | CLU |
| LINC00632 | hsa-miR-199b-5p | NCAM1 |
| LINC00632 | hsa-miR-199b-5p | SLC2A4 |
| LINC00632 | hsa-miR-199b-5p | KIAA1549L |
| LINC00632 | hsa-miR-199b-5p | C11orf87 |
| LINC00632 | hsa-miR-199b-5p | ST6GALNAC3 |
| LINC00632 | hsa-miR-199b-5p | SYNGR1 |
| LINC00632 | hsa-miR-199b-5p | C3orf70 |
| LINC00632 | hsa-miR-199b-5p | TSPAN18 |
| LINC00632 | hsa-miR-199b-5p | SLC24A2 |
| LINC00632 | hsa-miR-199b-5p | ATP2B2 |
| LINC00632 | hsa-miR-199b-5p | RSPO3 |
| LINC00632 | hsa-miR-199b-5p | DCX |
| LINC00632 | hsa-miR-199b-5p | POTEF |
| LINC00632 | hsa-miR-199b-5p | MAP2 |
| LINC00632 | hsa-miR-199b-5p | LMOD1 |
| LINC00632 | hsa-miR-199b-5p | GPR161 |
| LINC00632 | hsa-miR-199b-5p | ITGA10 |
| LINC00632 | hsa-miR-199b-5p | CDR1 |
| LINC00632 | hsa-miR-199b-5p | BEND6 |
| LINC00632 | hsa-miR-199b-5p | LRRC1 |
| LINC00632 | hsa-miR-199b-5p | KCNB1 |
| LINC00632 | hsa-miR-199b-5p | IRS4 |
| LINC00632 | hsa-miR-199b-5p | FABP3 |
| LINC00632 | hsa-miR-199b-5p | HIF3A |
| LINC00632 | hsa-miR-199b-5p | PKHD1L1 |
| LINC00632 | hsa-miR-199b-5p | PTCHD1 |
| LINC00632 | hsa-miR-199b-5p | DCLK1 |
| LINC00632 | hsa-miR-199b-5p | BNC2 |
| LINC00632 | hsa-miR-199b-5p | SMOC1 |
| LINC00632 | hsa-miR-199b-5p | LGI2 |
| LINC00632 | hsa-miR-199b-5p | SEMA5A |
| LINC00632 | hsa-miR-199b-5p | SLC22A3 |
| LINC00632 | hsa-miR-199b-5p | CDON |
| LINC00632 | hsa-miR-199b-5p | KALRN |
| LINC00632 | hsa-miR-199b-5p | SPOCK1 |
| LINC00632 | hsa-miR-199b-5p | MAPK10 |
| LINC00632 | hsa-miR-199b-5p | KCNG1 |
| LINC00632 | hsa-miR-199b-5p | ST8SIA1 |
| LINC00632 | hsa-miR-199b-5p | ZNF208 |
| LINC00632 | hsa-miR-199b-5p | MEIS2 |
| LINC00632 | hsa-miR-199b-5p | SRL |
| LINC00632 | hsa-miR-199b-5p | CRYBB3 |
| LINC00632 | hsa-miR-199b-5p | ADAMTSL5 |
| LINC00632 | hsa-miR-199b-5p | SYNPO2 |
| LINC00632 | hsa-miR-199b-5p | GFRA1 |
| LINC00632 | hsa-miR-199b-5p | MRGPRF |
| LINC00632 | hsa-miR-199b-5p | SHISA6 |
| LINC00632 | hsa-miR-199b-5p | FAM107A |
| LINC00632 | hsa-miR-199b-5p | KLF8 |
| LINC00632 | hsa-miR-199b-5p | ST6GALNAC5 |
| LINC00632 | hsa-miR-199b-5p | EPHA3 |
| LINC00632 | hsa-miR-199b-5p | DIO3 |
| LINC00632 | hsa-miR-199b-5p | PIANP |
| LINC00632 | hsa-miR-199b-5p | IGFBP6 |
| LINC00632 | hsa-miR-199b-5p | EML5 |
| LINC00632 | hsa-miR-199b-5p | DUOXA1 |
| LINC00632 | hsa-miR-199b-5p | PPP1R12B |
| AC092162.2 | hsa-miR-199b-5p | INMT |
| AC092162.2 | hsa-miR-199b-5p | CCDC80 |
| AC092162.2 | hsa-miR-199b-5p | HOXA13 |
| AC092162.2 | hsa-miR-199b-5p | HLF |
| AC092162.2 | hsa-miR-199b-5p | IGFBP5 |
| AC092162.2 | hsa-miR-199b-5p | RAB9B |
| AC092162.2 | hsa-miR-199b-5p | PTGIS |
| AC092162.2 | hsa-miR-199b-5p | LRRC17 |
| AC092162.2 | hsa-miR-199b-5p | CNN1 |
| AC092162.2 | hsa-miR-199b-5p | GATA5 |
| AC092162.2 | hsa-miR-199b-5p | MATN2 |
| AC092162.2 | hsa-miR-199b-5p | PI15 |
| AC092162.2 | hsa-miR-199b-5p | ASPA |
| AC092162.2 | hsa-miR-199b-5p | OPRK1 |
| AC092162.2 | hsa-miR-199b-5p | OSR1 |
| AC092162.2 | hsa-miR-199b-5p | SLC2A12 |
| AC092162.2 | hsa-miR-199b-5p | TMEM47 |
| AC092162.2 | hsa-miR-199b-5p | KCNE4 |
| AC092162.2 | hsa-miR-199b-5p | RAB3C |
| AC092162.2 | hsa-miR-199b-5p | PDLIM3 |
| AC092162.2 | hsa-miR-199b-5p | TCF23 |
| AC092162.2 | hsa-miR-199b-5p | FAT3 |
| AC092162.2 | hsa-miR-199b-5p | TMEM132B |
| AC092162.2 | hsa-miR-199b-5p | MYRIP |
| AC092162.2 | hsa-miR-199b-5p | KCNK3 |
| AC092162.2 | hsa-miR-199b-5p | GPR37 |
| AC092162.2 | hsa-miR-199b-5p | TUB |
| AC092162.2 | hsa-miR-199b-5p | C7 |
| AC092162.2 | hsa-miR-199b-5p | GPR88 |
| AC092162.2 | hsa-miR-199b-5p | CLU |
| AC092162.2 | hsa-miR-199b-5p | NCAM1 |
| AC092162.2 | hsa-miR-199b-5p | SLC2A4 |
| AC092162.2 | hsa-miR-199b-5p | KIAA1549L |
| AC092162.2 | hsa-miR-199b-5p | C11orf87 |
| AC092162.2 | hsa-miR-199b-5p | ST6GALNAC3 |
| AC092162.2 | hsa-miR-199b-5p | SYNGR1 |
| AC092162.2 | hsa-miR-199b-5p | C3orf70 |
| AC092162.2 | hsa-miR-199b-5p | TSPAN18 |
| AC092162.2 | hsa-miR-199b-5p | SLC24A2 |
| AC092162.2 | hsa-miR-199b-5p | ATP2B2 |
| AC092162.2 | hsa-miR-199b-5p | RSPO3 |
| AC092162.2 | hsa-miR-199b-5p | DCX |
| AC092162.2 | hsa-miR-199b-5p | POTEF |
| AC092162.2 | hsa-miR-199b-5p | MAP2 |
| AC092162.2 | hsa-miR-199b-5p | LMOD1 |
| AC092162.2 | hsa-miR-199b-5p | GPR161 |
| AC092162.2 | hsa-miR-199b-5p | ITGA10 |
| AC092162.2 | hsa-miR-199b-5p | CDR1 |
| AC092162.2 | hsa-miR-199b-5p | BEND6 |
| AC092162.2 | hsa-miR-199b-5p | LRRC1 |
| AC092162.2 | hsa-miR-199b-5p | KCNB1 |
| AC092162.2 | hsa-miR-199b-5p | IRS4 |
| AC092162.2 | hsa-miR-199b-5p | FABP3 |
| AC092162.2 | hsa-miR-199b-5p | HIF3A |
| AC092162.2 | hsa-miR-199b-5p | PKHD1L1 |
| AC092162.2 | hsa-miR-199b-5p | PTCHD1 |
| AC092162.2 | hsa-miR-199b-5p | DCLK1 |
| AC092162.2 | hsa-miR-199b-5p | BNC2 |
| AC092162.2 | hsa-miR-199b-5p | SMOC1 |
| AC092162.2 | hsa-miR-199b-5p | LGI2 |
| AC092162.2 | hsa-miR-199b-5p | SEMA5A |
| AC092162.2 | hsa-miR-199b-5p | SLC22A3 |
| AC092162.2 | hsa-miR-199b-5p | CDON |
| AC092162.2 | hsa-miR-199b-5p | KALRN |
| AC092162.2 | hsa-miR-199b-5p | SPOCK1 |
| AC092162.2 | hsa-miR-199b-5p | MAPK10 |
| AC092162.2 | hsa-miR-199b-5p | KCNG1 |
| AC092162.2 | hsa-miR-199b-5p | ST8SIA1 |
| AC092162.2 | hsa-miR-199b-5p | ZNF208 |
| AC092162.2 | hsa-miR-199b-5p | MEIS2 |
| AC092162.2 | hsa-miR-199b-5p | SRL |
| AC092162.2 | hsa-miR-199b-5p | CRYBB3 |
| AC092162.2 | hsa-miR-199b-5p | ADAMTSL5 |
| AC092162.2 | hsa-miR-199b-5p | SYNPO2 |
| AC092162.2 | hsa-miR-199b-5p | GFRA1 |
| AC092162.2 | hsa-miR-199b-5p | MRGPRF |
| AC092162.2 | hsa-miR-199b-5p | SHISA6 |
| AC092162.2 | hsa-miR-199b-5p | FAM107A |
| AC092162.2 | hsa-miR-199b-5p | KLF8 |
| AC092162.2 | hsa-miR-199b-5p | ST6GALNAC5 |
| AC092162.2 | hsa-miR-199b-5p | EPHA3 |
| AC092162.2 | hsa-miR-199b-5p | DIO3 |
| AC092162.2 | hsa-miR-199b-5p | PIANP |
| AC092162.2 | hsa-miR-199b-5p | IGFBP6 |
| AC092162.2 | hsa-miR-199b-5p | EML5 |
| AC092162.2 | hsa-miR-199b-5p | DUOXA1 |
| AC092162.2 | hsa-miR-199b-5p | PPP1R12B |
| LINC00632 | hsa-miR-425-5p | TLL1 |
| LINC00632 | hsa-miR-425-5p | REEP1 |
| LINC00632 | hsa-miR-425-5p | CCDC80 |
| LINC00632 | hsa-miR-425-5p | HLF |
| LINC00632 | hsa-miR-425-5p | GNAZ |
| LINC00632 | hsa-miR-425-5p | TEX15 |
| LINC00632 | hsa-miR-425-5p | HTR2B |
| LINC00632 | hsa-miR-425-5p | PI15 |
| LINC00632 | hsa-miR-425-5p | ASXL3 |
| LINC00632 | hsa-miR-425-5p | SLC2A12 |
| LINC00632 | hsa-miR-425-5p | RAB3C |
| LINC00632 | hsa-miR-425-5p | PDLIM3 |
| LINC00632 | hsa-miR-425-5p | UNC5D |
| LINC00632 | hsa-miR-425-5p | HPGD |
| LINC00632 | hsa-miR-425-5p | NPY1R |
| LINC00632 | hsa-miR-425-5p | TMEM132B |
| LINC00632 | hsa-miR-425-5p | MYRIP |
| LINC00632 | hsa-miR-425-5p | GPR37 |
| LINC00632 | hsa-miR-425-5p | ADH1B |
| LINC00632 | hsa-miR-425-5p | LPP |
| LINC00632 | hsa-miR-425-5p | BVES |
| LINC00632 | hsa-miR-425-5p | GPR88 |
| LINC00632 | hsa-miR-425-5p | CLU |
| LINC00632 | hsa-miR-425-5p | NCAM1 |
| LINC00632 | hsa-miR-425-5p | FIBIN |
| LINC00632 | hsa-miR-425-5p | SYT9 |
| LINC00632 | hsa-miR-425-5p | KIAA1549L |
| LINC00632 | hsa-miR-425-5p | C3orf80 |
| LINC00632 | hsa-miR-425-5p | C11orf87 |
| LINC00632 | hsa-miR-425-5p | SYNGR1 |
| LINC00632 | hsa-miR-425-5p | WSCD2 |
| LINC00632 | hsa-miR-425-5p | C3orf70 |
| LINC00632 | hsa-miR-425-5p | SLC24A2 |
| LINC00632 | hsa-miR-425-5p | NR3C2 |
| LINC00632 | hsa-miR-425-5p | DCX |
| LINC00632 | hsa-miR-425-5p | PLN |
| LINC00632 | hsa-miR-425-5p | PHACTR3 |
| LINC00632 | hsa-miR-425-5p | CNR1 |
| LINC00632 | hsa-miR-425-5p | DNM3 |
| LINC00632 | hsa-miR-425-5p | GPR161 |
| LINC00632 | hsa-miR-425-5p | PBX1 |
| LINC00632 | hsa-miR-425-5p | LDOC1 |
| LINC00632 | hsa-miR-425-5p | PTGFR |
| LINC00632 | hsa-miR-425-5p | LRRC1 |
| LINC00632 | hsa-miR-425-5p | PTGER3 |
| LINC00632 | hsa-miR-425-5p | SORBS1 |
| LINC00632 | hsa-miR-425-5p | KCNB1 |
| LINC00632 | hsa-miR-425-5p | PAK3 |
| LINC00632 | hsa-miR-425-5p | CHRDL1 |
| LINC00632 | hsa-miR-425-5p | IRS4 |
| LINC00632 | hsa-miR-425-5p | HYI |
| LINC00632 | hsa-miR-425-5p | RAB40A |
| LINC00632 | hsa-miR-425-5p | PRKG1 |
| LINC00632 | hsa-miR-425-5p | DMD |
| LINC00632 | hsa-miR-425-5p | BNC2 |
| LINC00632 | hsa-miR-425-5p | SEMA5A |
| LINC00632 | hsa-miR-425-5p | SLC22A3 |
| LINC00632 | hsa-miR-425-5p | CDON |
| LINC00632 | hsa-miR-425-5p | NT5DC3 |
| LINC00632 | hsa-miR-425-5p | PRIMA1 |
| LINC00632 | hsa-miR-425-5p | ST8SIA1 |
| LINC00632 | hsa-miR-425-5p | RIC3 |
| LINC00632 | hsa-miR-425-5p | MEIS2 |
| LINC00632 | hsa-miR-425-5p | SRL |
| LINC00632 | hsa-miR-425-5p | MRVI1 |
| LINC00632 | hsa-miR-425-5p | HMCN2 |
| LINC00632 | hsa-miR-425-5p | GFRA1 |
| LINC00632 | hsa-miR-425-5p | SHISA6 |
| LINC00632 | hsa-miR-425-5p | TCEA3 |
| LINC00632 | hsa-miR-425-5p | MUC15 |
| LINC00632 | hsa-miR-425-5p | ST6GALNAC5 |
| LINC00632 | hsa-miR-425-5p | SLIT3 |
| LINC00632 | hsa-miR-425-5p | NAALAD2 |
| LINC00632 | hsa-miR-425-5p | TRERF1 |
| LINC00632 | hsa-miR-425-5p | ADCYAP1 |
| LINC00632 | hsa-miR-199a-5p | INMT |
| LINC00632 | hsa-miR-199a-5p | CCDC80 |
| LINC00632 | hsa-miR-199a-5p | HOXA13 |
| LINC00632 | hsa-miR-199a-5p | HLF |
| LINC00632 | hsa-miR-199a-5p | IGFBP5 |
| LINC00632 | hsa-miR-199a-5p | RAB9B |
| LINC00632 | hsa-miR-199a-5p | PTGIS |
| LINC00632 | hsa-miR-199a-5p | LRRC17 |
| LINC00632 | hsa-miR-199a-5p | CNN1 |
| LINC00632 | hsa-miR-199a-5p | GATA5 |
| LINC00632 | hsa-miR-199a-5p | MATN2 |
| LINC00632 | hsa-miR-199a-5p | PI15 |
| LINC00632 | hsa-miR-199a-5p | ASPA |
| LINC00632 | hsa-miR-199a-5p | OPRK1 |
| LINC00632 | hsa-miR-199a-5p | OSR1 |
| LINC00632 | hsa-miR-199a-5p | SLC2A12 |
| LINC00632 | hsa-miR-199a-5p | TMEM47 |
| LINC00632 | hsa-miR-199a-5p | KCNE4 |
| LINC00632 | hsa-miR-199a-5p | RAB3C |
| LINC00632 | hsa-miR-199a-5p | PDLIM3 |
| LINC00632 | hsa-miR-199a-5p | TCF23 |
| LINC00632 | hsa-miR-199a-5p | FAT3 |
| LINC00632 | hsa-miR-199a-5p | TMEM132B |
| LINC00632 | hsa-miR-199a-5p | MYRIP |
| LINC00632 | hsa-miR-199a-5p | KCNK3 |
| LINC00632 | hsa-miR-199a-5p | GPR37 |
| LINC00632 | hsa-miR-199a-5p | TUB |
| LINC00632 | hsa-miR-199a-5p | C7 |
| LINC00632 | hsa-miR-199a-5p | GPR88 |
| LINC00632 | hsa-miR-199a-5p | CLU |
| LINC00632 | hsa-miR-199a-5p | NCAM1 |
| LINC00632 | hsa-miR-199a-5p | SLC2A4 |
| LINC00632 | hsa-miR-199a-5p | KIAA1549L |
| LINC00632 | hsa-miR-199a-5p | C11orf87 |
| LINC00632 | hsa-miR-199a-5p | ST6GALNAC3 |
| LINC00632 | hsa-miR-199a-5p | SYNGR1 |
| LINC00632 | hsa-miR-199a-5p | C3orf70 |
| LINC00632 | hsa-miR-199a-5p | TSPAN18 |
| LINC00632 | hsa-miR-199a-5p | SLC24A2 |
| LINC00632 | hsa-miR-199a-5p | ATP2B2 |
| LINC00632 | hsa-miR-199a-5p | RSPO3 |
| LINC00632 | hsa-miR-199a-5p | DCX |
| LINC00632 | hsa-miR-199a-5p | POTEF |
| LINC00632 | hsa-miR-199a-5p | MAP2 |
| LINC00632 | hsa-miR-199a-5p | LMOD1 |
| LINC00632 | hsa-miR-199a-5p | GPR161 |
| LINC00632 | hsa-miR-199a-5p | ITGA10 |
| LINC00632 | hsa-miR-199a-5p | CDR1 |
| LINC00632 | hsa-miR-199a-5p | BEND6 |
| LINC00632 | hsa-miR-199a-5p | LRRC1 |
| LINC00632 | hsa-miR-199a-5p | KCNB1 |
| LINC00632 | hsa-miR-199a-5p | IRS4 |
| LINC00632 | hsa-miR-199a-5p | FABP3 |
| LINC00632 | hsa-miR-199a-5p | HIF3A |
| LINC00632 | hsa-miR-199a-5p | PKHD1L1 |
| LINC00632 | hsa-miR-199a-5p | PTCHD1 |
| LINC00632 | hsa-miR-199a-5p | DCLK1 |
| LINC00632 | hsa-miR-199a-5p | BNC2 |
| LINC00632 | hsa-miR-199a-5p | SMOC1 |
| LINC00632 | hsa-miR-199a-5p | LGI2 |
| LINC00632 | hsa-miR-199a-5p | SEMA5A |
| LINC00632 | hsa-miR-199a-5p | SLC22A3 |
| LINC00632 | hsa-miR-199a-5p | CDON |
| LINC00632 | hsa-miR-199a-5p | KALRN |
| LINC00632 | hsa-miR-199a-5p | SPOCK1 |
| LINC00632 | hsa-miR-199a-5p | MAPK10 |
| LINC00632 | hsa-miR-199a-5p | KCNG1 |
| LINC00632 | hsa-miR-199a-5p | ST8SIA1 |
| LINC00632 | hsa-miR-199a-5p | ZNF208 |
| LINC00632 | hsa-miR-199a-5p | MEIS2 |
| LINC00632 | hsa-miR-199a-5p | SRL |
| LINC00632 | hsa-miR-199a-5p | CRYBB3 |
| LINC00632 | hsa-miR-199a-5p | ADAMTSL5 |
| LINC00632 | hsa-miR-199a-5p | SYNPO2 |
| LINC00632 | hsa-miR-199a-5p | GFRA1 |
| LINC00632 | hsa-miR-199a-5p | MRGPRF |
| LINC00632 | hsa-miR-199a-5p | SHISA6 |
| LINC00632 | hsa-miR-199a-5p | FAM107A |
| LINC00632 | hsa-miR-199a-5p | KLF8 |
| LINC00632 | hsa-miR-199a-5p | ST6GALNAC5 |
| LINC00632 | hsa-miR-199a-5p | EPHA3 |
| LINC00632 | hsa-miR-199a-5p | DIO3 |
| LINC00632 | hsa-miR-199a-5p | PIANP |
| LINC00632 | hsa-miR-199a-5p | IGFBP6 |
| LINC00632 | hsa-miR-199a-5p | EML5 |
| LINC00632 | hsa-miR-199a-5p | DUOXA1 |
| LINC00632 | hsa-miR-199a-5p | PPP1R12B |
| AL031429.2 | hsa-miR-199a-5p | INMT |
| AL031429.2 | hsa-miR-199a-5p | CCDC80 |
| AL031429.2 | hsa-miR-199a-5p | HOXA13 |
| AL031429.2 | hsa-miR-199a-5p | HLF |
| AL031429.2 | hsa-miR-199a-5p | IGFBP5 |
| AL031429.2 | hsa-miR-199a-5p | RAB9B |
| AL031429.2 | hsa-miR-199a-5p | PTGIS |
| AL031429.2 | hsa-miR-199a-5p | LRRC17 |
| AL031429.2 | hsa-miR-199a-5p | CNN1 |
| AL031429.2 | hsa-miR-199a-5p | GATA5 |
| AL031429.2 | hsa-miR-199a-5p | MATN2 |
| AL031429.2 | hsa-miR-199a-5p | PI15 |
| AL031429.2 | hsa-miR-199a-5p | ASPA |
| AL031429.2 | hsa-miR-199a-5p | OPRK1 |
| AL031429.2 | hsa-miR-199a-5p | OSR1 |
| AL031429.2 | hsa-miR-199a-5p | SLC2A12 |
| AL031429.2 | hsa-miR-199a-5p | TMEM47 |
| AL031429.2 | hsa-miR-199a-5p | KCNE4 |
| AL031429.2 | hsa-miR-199a-5p | RAB3C |
| AL031429.2 | hsa-miR-199a-5p | PDLIM3 |
| AL031429.2 | hsa-miR-199a-5p | TCF23 |
| AL031429.2 | hsa-miR-199a-5p | FAT3 |
| AL031429.2 | hsa-miR-199a-5p | TMEM132B |
| AL031429.2 | hsa-miR-199a-5p | MYRIP |
| AL031429.2 | hsa-miR-199a-5p | KCNK3 |
| AL031429.2 | hsa-miR-199a-5p | GPR37 |
| AL031429.2 | hsa-miR-199a-5p | TUB |
| AL031429.2 | hsa-miR-199a-5p | C7 |
| AL031429.2 | hsa-miR-199a-5p | GPR88 |
| AL031429.2 | hsa-miR-199a-5p | CLU |
| AL031429.2 | hsa-miR-199a-5p | NCAM1 |
| AL031429.2 | hsa-miR-199a-5p | SLC2A4 |
| AL031429.2 | hsa-miR-199a-5p | KIAA1549L |
| AL031429.2 | hsa-miR-199a-5p | C11orf87 |
| AL031429.2 | hsa-miR-199a-5p | ST6GALNAC3 |
| AL031429.2 | hsa-miR-199a-5p | SYNGR1 |
| AL031429.2 | hsa-miR-199a-5p | C3orf70 |
| AL031429.2 | hsa-miR-199a-5p | TSPAN18 |
| AL031429.2 | hsa-miR-199a-5p | SLC24A2 |
| AL031429.2 | hsa-miR-199a-5p | ATP2B2 |
| AL031429.2 | hsa-miR-199a-5p | RSPO3 |
| AL031429.2 | hsa-miR-199a-5p | DCX |
| AL031429.2 | hsa-miR-199a-5p | POTEF |
| AL031429.2 | hsa-miR-199a-5p | MAP2 |
| AL031429.2 | hsa-miR-199a-5p | LMOD1 |
| AL031429.2 | hsa-miR-199a-5p | GPR161 |
| AL031429.2 | hsa-miR-199a-5p | ITGA10 |
| AL031429.2 | hsa-miR-199a-5p | CDR1 |
| AL031429.2 | hsa-miR-199a-5p | BEND6 |
| AL031429.2 | hsa-miR-199a-5p | LRRC1 |
| AL031429.2 | hsa-miR-199a-5p | KCNB1 |
| AL031429.2 | hsa-miR-199a-5p | IRS4 |
| AL031429.2 | hsa-miR-199a-5p | FABP3 |
| AL031429.2 | hsa-miR-199a-5p | HIF3A |
| AL031429.2 | hsa-miR-199a-5p | PKHD1L1 |
| AL031429.2 | hsa-miR-199a-5p | PTCHD1 |
| AL031429.2 | hsa-miR-199a-5p | DCLK1 |
| AL031429.2 | hsa-miR-199a-5p | BNC2 |
| AL031429.2 | hsa-miR-199a-5p | SMOC1 |
| AL031429.2 | hsa-miR-199a-5p | LGI2 |
| AL031429.2 | hsa-miR-199a-5p | SEMA5A |
| AL031429.2 | hsa-miR-199a-5p | SLC22A3 |
| AL031429.2 | hsa-miR-199a-5p | CDON |
| AL031429.2 | hsa-miR-199a-5p | KALRN |
| AL031429.2 | hsa-miR-199a-5p | SPOCK1 |
| AL031429.2 | hsa-miR-199a-5p | MAPK10 |
| AL031429.2 | hsa-miR-199a-5p | KCNG1 |
| AL031429.2 | hsa-miR-199a-5p | ST8SIA1 |
| AL031429.2 | hsa-miR-199a-5p | ZNF208 |
| AL031429.2 | hsa-miR-199a-5p | MEIS2 |
| AL031429.2 | hsa-miR-199a-5p | SRL |
| AL031429.2 | hsa-miR-199a-5p | CRYBB3 |
| AL031429.2 | hsa-miR-199a-5p | ADAMTSL5 |
| AL031429.2 | hsa-miR-199a-5p | SYNPO2 |
| AL031429.2 | hsa-miR-199a-5p | GFRA1 |
| AL031429.2 | hsa-miR-199a-5p | MRGPRF |
| AL031429.2 | hsa-miR-199a-5p | SHISA6 |
| AL031429.2 | hsa-miR-199a-5p | FAM107A |
| AL031429.2 | hsa-miR-199a-5p | KLF8 |
| AL031429.2 | hsa-miR-199a-5p | ST6GALNAC5 |
| AL031429.2 | hsa-miR-199a-5p | EPHA3 |
| AL031429.2 | hsa-miR-199a-5p | DIO3 |
| AL031429.2 | hsa-miR-199a-5p | PIANP |
| AL031429.2 | hsa-miR-199a-5p | IGFBP6 |
| AL031429.2 | hsa-miR-199a-5p | EML5 |
| AL031429.2 | hsa-miR-199a-5p | DUOXA1 |
| AL031429.2 | hsa-miR-199a-5p | PPP1R12B |
| AF001548.3 | hsa-miR-17-5p | TLL1 |
| AF001548.3 | hsa-miR-17-5p | CCDC80 |
| AF001548.3 | hsa-miR-17-5p | HOXA13 |
| AF001548.3 | hsa-miR-17-5p | HLF |
| AF001548.3 | hsa-miR-17-5p | IGFBP5 |
| AF001548.3 | hsa-miR-17-5p | PTGIS |
| AF001548.3 | hsa-miR-17-5p | CNN1 |
| AF001548.3 | hsa-miR-17-5p | TEX15 |
| AF001548.3 | hsa-miR-17-5p | PI15 |
| AF001548.3 | hsa-miR-17-5p | ASPA |
| AF001548.3 | hsa-miR-17-5p | ASXL3 |
| AF001548.3 | hsa-miR-17-5p | OSR1 |
| AF001548.3 | hsa-miR-17-5p | TRPC1 |
| AF001548.3 | hsa-miR-17-5p | SLC2A12 |
| AF001548.3 | hsa-miR-17-5p | TMEM47 |
| AF001548.3 | hsa-miR-17-5p | KCNE4 |
| AF001548.3 | hsa-miR-17-5p | PDLIM3 |
| AF001548.3 | hsa-miR-17-5p | UNC5D |
| AF001548.3 | hsa-miR-17-5p | FBXO27 |
| AF001548.3 | hsa-miR-17-5p | TCF23 |
| AF001548.3 | hsa-miR-17-5p | EMCN |
| AF001548.3 | hsa-miR-17-5p | HPGD |
| AF001548.3 | hsa-miR-17-5p | NPY1R |
| AF001548.3 | hsa-miR-17-5p | FAT3 |
| AF001548.3 | hsa-miR-17-5p | TMEM132B |
| AF001548.3 | hsa-miR-17-5p | GPR37 |
| AF001548.3 | hsa-miR-17-5p | GPR22 |
| AF001548.3 | hsa-miR-17-5p | ADH1B |
| AF001548.3 | hsa-miR-17-5p | MSRB3 |
| AF001548.3 | hsa-miR-17-5p | LPP |
| AF001548.3 | hsa-miR-17-5p | BVES |
| AF001548.3 | hsa-miR-17-5p | PLAG1 |
| AF001548.3 | hsa-miR-17-5p | SLC2A4 |
| AF001548.3 | hsa-miR-17-5p | FIBIN |
| AF001548.3 | hsa-miR-17-5p | KIAA1549L |
| AF001548.3 | hsa-miR-17-5p | C3orf80 |
| AF001548.3 | hsa-miR-17-5p | C11orf87 |
| AF001548.3 | hsa-miR-17-5p | TNFAIP8L3 |
| AF001548.3 | hsa-miR-17-5p | ST6GALNAC3 |
| AF001548.3 | hsa-miR-17-5p | C3orf70 |
| AF001548.3 | hsa-miR-17-5p | SLC24A2 |
| AF001548.3 | hsa-miR-17-5p | PRELP |
| AF001548.3 | hsa-miR-17-5p | ATP2B2 |
| AF001548.3 | hsa-miR-17-5p | SMOC2 |
| AF001548.3 | hsa-miR-17-5p | MYOM1 |
| AF001548.3 | hsa-miR-17-5p | ADAM33 |
| AF001548.3 | hsa-miR-17-5p | DCX |
| AF001548.3 | hsa-miR-17-5p | ANKRD36 |
| AF001548.3 | hsa-miR-17-5p | ZNF483 |
| AF001548.3 | hsa-miR-17-5p | MFAP5 |
| AF001548.3 | hsa-miR-17-5p | MAP2 |
| AF001548.3 | hsa-miR-17-5p | FBXL22 |
| AF001548.3 | hsa-miR-17-5p | MYLK |
| AF001548.3 | hsa-miR-17-5p | CNR1 |
| AF001548.3 | hsa-miR-17-5p | DNM3 |
| AF001548.3 | hsa-miR-17-5p | GPR161 |
| AF001548.3 | hsa-miR-17-5p | ITGA10 |
| AF001548.3 | hsa-miR-17-5p | RBM20 |
| AF001548.3 | hsa-miR-17-5p | SYPL2 |
| AF001548.3 | hsa-miR-17-5p | CDR1 |
| AF001548.3 | hsa-miR-17-5p | PTGER3 |
| AF001548.3 | hsa-miR-17-5p | SLC25A27 |
| AF001548.3 | hsa-miR-17-5p | KCNB1 |
| AF001548.3 | hsa-miR-17-5p | PAK3 |
| AF001548.3 | hsa-miR-17-5p | IRS4 |
| AF001548.3 | hsa-miR-17-5p | SLC6A9 |
| AF001548.3 | hsa-miR-17-5p | WISP2 |
| AF001548.3 | hsa-miR-17-5p | PRKG1 |
| AF001548.3 | hsa-miR-17-5p | TRPM3 |
| AF001548.3 | hsa-miR-17-5p | MAMDC2 |
| AF001548.3 | hsa-miR-17-5p | PKHD1L1 |
| AF001548.3 | hsa-miR-17-5p | PTCHD1 |
| AF001548.3 | hsa-miR-17-5p | DCLK1 |
| AF001548.3 | hsa-miR-17-5p | BNC2 |
| AF001548.3 | hsa-miR-17-5p | SMOC1 |
| AF001548.3 | hsa-miR-17-5p | CYS1 |
| AF001548.3 | hsa-miR-17-5p | LGI2 |
| AF001548.3 | hsa-miR-17-5p | SEMA5A |
| AF001548.3 | hsa-miR-17-5p | SLC22A3 |
| AF001548.3 | hsa-miR-17-5p | NT5DC3 |
| AF001548.3 | hsa-miR-17-5p | ANKRD39 |
| AF001548.3 | hsa-miR-17-5p | SPOCK1 |
| AF001548.3 | hsa-miR-17-5p | FAM13A |
| AF001548.3 | hsa-miR-17-5p | MAPK10 |
| AF001548.3 | hsa-miR-17-5p | CDKL1 |
| AF001548.3 | hsa-miR-17-5p | KCNG1 |
| AF001548.3 | hsa-miR-17-5p | ST8SIA1 |
| AF001548.3 | hsa-miR-17-5p | PGM5 |
| AF001548.3 | hsa-miR-17-5p | RIC3 |
| AF001548.3 | hsa-miR-17-5p | DGKB |
| AF001548.3 | hsa-miR-17-5p | ADAMTSL5 |
| AF001548.3 | hsa-miR-17-5p | MRVI1 |
| AF001548.3 | hsa-miR-17-5p | SYNPO2 |
| AF001548.3 | hsa-miR-17-5p | GFRA1 |
| AF001548.3 | hsa-miR-17-5p | SHISA6 |
| AF001548.3 | hsa-miR-17-5p | KLF8 |
| AF001548.3 | hsa-miR-17-5p | FSBP |
| AF001548.3 | hsa-miR-17-5p | SLC15A2 |
| AF001548.3 | hsa-miR-17-5p | TMEM158 |
| AF001548.3 | hsa-miR-17-5p | SORCS2 |
| AF001548.3 | hsa-miR-17-5p | YPEL4 |
| AF001548.3 | hsa-miR-17-5p | SYBU |
| AF001548.3 | hsa-miR-17-5p | NAALAD2 |
| AF001548.3 | hsa-miR-17-5p | PIANP |
| AF001548.3 | hsa-miR-17-5p | FXYD6 |
| AF001548.3 | hsa-miR-17-5p | FHL5 |
| AF001548.3 | hsa-miR-17-5p | HSD17B6 |
| AF001548.3 | hsa-miR-17-5p | EML5 |
| AF001548.3 | hsa-miR-17-5p | CCDC68 |
| AF001548.3 | hsa-miR-17-5p | PPP1R12B |
| AF001548.3 | hsa-miR-20b-5p | TLL1 |
| AF001548.3 | hsa-miR-20b-5p | CCDC80 |
| AF001548.3 | hsa-miR-20b-5p | HOXA13 |
| AF001548.3 | hsa-miR-20b-5p | HLF |
| AF001548.3 | hsa-miR-20b-5p | IGFBP5 |
| AF001548.3 | hsa-miR-20b-5p | PTGIS |
| AF001548.3 | hsa-miR-20b-5p | CNN1 |
| AF001548.3 | hsa-miR-20b-5p | TEX15 |
| AF001548.3 | hsa-miR-20b-5p | PI15 |
| AF001548.3 | hsa-miR-20b-5p | ASPA |
| AF001548.3 | hsa-miR-20b-5p | ASXL3 |
| AF001548.3 | hsa-miR-20b-5p | OSR1 |
| AF001548.3 | hsa-miR-20b-5p | TRPC1 |
| AF001548.3 | hsa-miR-20b-5p | SLC2A12 |
| AF001548.3 | hsa-miR-20b-5p | TMEM47 |
| AF001548.3 | hsa-miR-20b-5p | KCNE4 |
| AF001548.3 | hsa-miR-20b-5p | PDLIM3 |
| AF001548.3 | hsa-miR-20b-5p | UNC5D |
| AF001548.3 | hsa-miR-20b-5p | FBXO27 |
| AF001548.3 | hsa-miR-20b-5p | TCF23 |
| AF001548.3 | hsa-miR-20b-5p | EMCN |
| AF001548.3 | hsa-miR-20b-5p | HPGD |
| AF001548.3 | hsa-miR-20b-5p | NPY1R |
| AF001548.3 | hsa-miR-20b-5p | FAT3 |
| AF001548.3 | hsa-miR-20b-5p | TMEM132B |
| AF001548.3 | hsa-miR-20b-5p | GPR37 |
| AF001548.3 | hsa-miR-20b-5p | GPR22 |
| AF001548.3 | hsa-miR-20b-5p | ADH1B |
| AF001548.3 | hsa-miR-20b-5p | MSRB3 |
| AF001548.3 | hsa-miR-20b-5p | LPP |
| AF001548.3 | hsa-miR-20b-5p | BVES |
| AF001548.3 | hsa-miR-20b-5p | PLAG1 |
| AF001548.3 | hsa-miR-20b-5p | SLC2A4 |
| AF001548.3 | hsa-miR-20b-5p | FIBIN |
| AF001548.3 | hsa-miR-20b-5p | KIAA1549L |
| AF001548.3 | hsa-miR-20b-5p | C3orf80 |
| AF001548.3 | hsa-miR-20b-5p | C11orf87 |
| AF001548.3 | hsa-miR-20b-5p | TNFAIP8L3 |
| AF001548.3 | hsa-miR-20b-5p | ST6GALNAC3 |
| AF001548.3 | hsa-miR-20b-5p | C3orf70 |
| AF001548.3 | hsa-miR-20b-5p | SLC24A2 |
| AF001548.3 | hsa-miR-20b-5p | PRELP |
| AF001548.3 | hsa-miR-20b-5p | ATP2B2 |
| AF001548.3 | hsa-miR-20b-5p | SMOC2 |
| AF001548.3 | hsa-miR-20b-5p | MYOM1 |
| AF001548.3 | hsa-miR-20b-5p | ADAM33 |
| AF001548.3 | hsa-miR-20b-5p | DCX |
| AF001548.3 | hsa-miR-20b-5p | ANKRD36 |
| AF001548.3 | hsa-miR-20b-5p | ZNF483 |
| AF001548.3 | hsa-miR-20b-5p | MFAP5 |
| AF001548.3 | hsa-miR-20b-5p | MAP2 |
| AF001548.3 | hsa-miR-20b-5p | FBXL22 |
| AF001548.3 | hsa-miR-20b-5p | MYLK |
| AF001548.3 | hsa-miR-20b-5p | CNR1 |
| AF001548.3 | hsa-miR-20b-5p | DNM3 |
| AF001548.3 | hsa-miR-20b-5p | GPR161 |
| AF001548.3 | hsa-miR-20b-5p | ITGA10 |
| AF001548.3 | hsa-miR-20b-5p | RBM20 |
| AF001548.3 | hsa-miR-20b-5p | SYPL2 |
| AF001548.3 | hsa-miR-20b-5p | CDR1 |
| AF001548.3 | hsa-miR-20b-5p | PTGER3 |
| AF001548.3 | hsa-miR-20b-5p | SLC25A27 |
| AF001548.3 | hsa-miR-20b-5p | KCNB1 |
| AF001548.3 | hsa-miR-20b-5p | PAK3 |
| AF001548.3 | hsa-miR-20b-5p | IRS4 |
| AF001548.3 | hsa-miR-20b-5p | SLC6A9 |
| AF001548.3 | hsa-miR-20b-5p | WISP2 |
| AF001548.3 | hsa-miR-20b-5p | PRKG1 |
| AF001548.3 | hsa-miR-20b-5p | TRPM3 |
| AF001548.3 | hsa-miR-20b-5p | MAMDC2 |
| AF001548.3 | hsa-miR-20b-5p | PKHD1L1 |
| AF001548.3 | hsa-miR-20b-5p | PTCHD1 |
| AF001548.3 | hsa-miR-20b-5p | DCLK1 |
| AF001548.3 | hsa-miR-20b-5p | BNC2 |
| AF001548.3 | hsa-miR-20b-5p | SMOC1 |
| AF001548.3 | hsa-miR-20b-5p | CYS1 |
| AF001548.3 | hsa-miR-20b-5p | LGI2 |
| AF001548.3 | hsa-miR-20b-5p | SEMA5A |
| AF001548.3 | hsa-miR-20b-5p | SLC22A3 |
| AF001548.3 | hsa-miR-20b-5p | NT5DC3 |
| AF001548.3 | hsa-miR-20b-5p | ANKRD39 |
| AF001548.3 | hsa-miR-20b-5p | SPOCK1 |
| AF001548.3 | hsa-miR-20b-5p | FAM13A |
| AF001548.3 | hsa-miR-20b-5p | MAPK10 |
| AF001548.3 | hsa-miR-20b-5p | CDKL1 |
| AF001548.3 | hsa-miR-20b-5p | KCNG1 |
| AF001548.3 | hsa-miR-20b-5p | ST8SIA1 |
| AF001548.3 | hsa-miR-20b-5p | PGM5 |
| AF001548.3 | hsa-miR-20b-5p | RIC3 |
| AF001548.3 | hsa-miR-20b-5p | DGKB |
| AF001548.3 | hsa-miR-20b-5p | ADAMTSL5 |
| AF001548.3 | hsa-miR-20b-5p | MRVI1 |
| AF001548.3 | hsa-miR-20b-5p | SYNPO2 |
| AF001548.3 | hsa-miR-20b-5p | GFRA1 |
| AF001548.3 | hsa-miR-20b-5p | SHISA6 |
| AF001548.3 | hsa-miR-20b-5p | KLF8 |
| AF001548.3 | hsa-miR-20b-5p | FSBP |
| AF001548.3 | hsa-miR-20b-5p | SLC15A2 |
| AF001548.3 | hsa-miR-20b-5p | TMEM158 |
| AF001548.3 | hsa-miR-20b-5p | SORCS2 |
| AF001548.3 | hsa-miR-20b-5p | YPEL4 |
| AF001548.3 | hsa-miR-20b-5p | SYBU |
| AF001548.3 | hsa-miR-20b-5p | NAALAD2 |
| AF001548.3 | hsa-miR-20b-5p | PIANP |
| AF001548.3 | hsa-miR-20b-5p | FXYD6 |
| AF001548.3 | hsa-miR-20b-5p | FHL5 |
| AF001548.3 | hsa-miR-20b-5p | HSD17B6 |
| AF001548.3 | hsa-miR-20b-5p | EML5 |
| AF001548.3 | hsa-miR-20b-5p | CCDC68 |
| AF001548.3 | hsa-miR-20b-5p | PPP1R12B |
| AC024901.1 | hsa-miR-181b-5p | TLL1 |
| AC024901.1 | hsa-miR-181b-5p | REEP1 |
| AC024901.1 | hsa-miR-181b-5p | ADAM11 |
| AC024901.1 | hsa-miR-181b-5p | CCDC80 |
| AC024901.1 | hsa-miR-181b-5p | ACTA2 |
| AC024901.1 | hsa-miR-181b-5p | HLF |
| AC024901.1 | hsa-miR-181b-5p | SLC5A9 |
| AC024901.1 | hsa-miR-181b-5p | NRK |
| AC024901.1 | hsa-miR-181b-5p | PTGIS |
| AC024901.1 | hsa-miR-181b-5p | SCGB1D2 |
| AC024901.1 | hsa-miR-181b-5p | ATP1B2 |
| AC024901.1 | hsa-miR-181b-5p | ASPA |
| AC024901.1 | hsa-miR-181b-5p | BCHE |
| AC024901.1 | hsa-miR-181b-5p | OPRK1 |
| AC024901.1 | hsa-miR-181b-5p | ASXL3 |
| AC024901.1 | hsa-miR-181b-5p | TRPC1 |
| AC024901.1 | hsa-miR-181b-5p | TMEM47 |
| AC024901.1 | hsa-miR-181b-5p | RAB3C |
| AC024901.1 | hsa-miR-181b-5p | PDLIM3 |
| AC024901.1 | hsa-miR-181b-5p | UNC5D |
| AC024901.1 | hsa-miR-181b-5p | ACTC1 |
| AC024901.1 | hsa-miR-181b-5p | HFM1 |
| AC024901.1 | hsa-miR-181b-5p | COL6A3 |
| AC024901.1 | hsa-miR-181b-5p | TCF23 |
| AC024901.1 | hsa-miR-181b-5p | FAT3 |
| AC024901.1 | hsa-miR-181b-5p | TMEM132B |
| AC024901.1 | hsa-miR-181b-5p | HSPB3 |
| AC024901.1 | hsa-miR-181b-5p | VAT1L |
| AC024901.1 | hsa-miR-181b-5p | SLC38A11 |
| AC024901.1 | hsa-miR-181b-5p | GPR37 |
| AC024901.1 | hsa-miR-181b-5p | GPR22 |
| AC024901.1 | hsa-miR-181b-5p | ADH1B |
| AC024901.1 | hsa-miR-181b-5p | TUB |
| AC024901.1 | hsa-miR-181b-5p | MSRB3 |
| AC024901.1 | hsa-miR-181b-5p | GAL3ST3 |
| AC024901.1 | hsa-miR-181b-5p | LPP |
| AC024901.1 | hsa-miR-181b-5p | C7 |
| AC024901.1 | hsa-miR-181b-5p | BVES |
| AC024901.1 | hsa-miR-181b-5p | GPR88 |
| AC024901.1 | hsa-miR-181b-5p | PLAG1 |
| AC024901.1 | hsa-miR-181b-5p | KIAA1549L |
| AC024901.1 | hsa-miR-181b-5p | DMRTA1 |
| AC024901.1 | hsa-miR-181b-5p | C3orf80 |
| AC024901.1 | hsa-miR-181b-5p | C11orf87 |
| AC024901.1 | hsa-miR-181b-5p | GRID1 |
| AC024901.1 | hsa-miR-181b-5p | ST6GALNAC3 |
| AC024901.1 | hsa-miR-181b-5p | WSCD2 |
| AC024901.1 | hsa-miR-181b-5p | CADPS2 |
| AC024901.1 | hsa-miR-181b-5p | TSPAN18 |
| AC024901.1 | hsa-miR-181b-5p | NR3C2 |
| AC024901.1 | hsa-miR-181b-5p | ATP2B2 |
| AC024901.1 | hsa-miR-181b-5p | JPH4 |
| AC024901.1 | hsa-miR-181b-5p | PLN |
| AC024901.1 | hsa-miR-181b-5p | ZNF483 |
| AC024901.1 | hsa-miR-181b-5p | ATL1 |
| AC024901.1 | hsa-miR-181b-5p | MAP2 |
| AC024901.1 | hsa-miR-181b-5p | CNR1 |
| AC024901.1 | hsa-miR-181b-5p | DNM3 |
| AC024901.1 | hsa-miR-181b-5p | PBX1 |
| AC024901.1 | hsa-miR-181b-5p | SYPL2 |
| AC024901.1 | hsa-miR-181b-5p | PTGER3 |
| AC024901.1 | hsa-miR-181b-5p | SLC25A27 |
| AC024901.1 | hsa-miR-181b-5p | KCNB1 |
| AC024901.1 | hsa-miR-181b-5p | IRS4 |
| AC024901.1 | hsa-miR-181b-5p | RAB40A |
| AC024901.1 | hsa-miR-181b-5p | PRKG1 |
| AC024901.1 | hsa-miR-181b-5p | TRPM3 |
| AC024901.1 | hsa-miR-181b-5p | MAMDC2 |
| AC024901.1 | hsa-miR-181b-5p | PTCHD1 |
| AC024901.1 | hsa-miR-181b-5p | DCLK1 |
| AC024901.1 | hsa-miR-181b-5p | BNC2 |
| AC024901.1 | hsa-miR-181b-5p | IGF2 |
| AC024901.1 | hsa-miR-181b-5p | LGI2 |
| AC024901.1 | hsa-miR-181b-5p | RYR3 |
| AC024901.1 | hsa-miR-181b-5p | SLC22A3 |
| AC024901.1 | hsa-miR-181b-5p | CDON |
| AC024901.1 | hsa-miR-181b-5p | NT5DC3 |
| AC024901.1 | hsa-miR-181b-5p | PRIMA1 |
| AC024901.1 | hsa-miR-181b-5p | SPOCK1 |
| AC024901.1 | hsa-miR-181b-5p | FAM13A |
| AC024901.1 | hsa-miR-181b-5p | MAPK10 |
| AC024901.1 | hsa-miR-181b-5p | KCNG1 |
| AC024901.1 | hsa-miR-181b-5p | ST8SIA1 |
| AC024901.1 | hsa-miR-181b-5p | RIC3 |
| AC024901.1 | hsa-miR-181b-5p | MEIS2 |
| AC024901.1 | hsa-miR-181b-5p | DGKB |
| AC024901.1 | hsa-miR-181b-5p | SYNC |
| AC024901.1 | hsa-miR-181b-5p | ITGA11 |
| AC024901.1 | hsa-miR-181b-5p | SYNPO2 |
| AC024901.1 | hsa-miR-181b-5p | GFRA1 |
| AC024901.1 | hsa-miR-181b-5p | SHISA6 |
| AC024901.1 | hsa-miR-181b-5p | FAM107A |
| AC024901.1 | hsa-miR-181b-5p | KLF8 |
| AC024901.1 | hsa-miR-181b-5p | ABI3BP |
| AC024901.1 | hsa-miR-181b-5p | ST6GALNAC5 |
| AC024901.1 | hsa-miR-181b-5p | FSBP |
| AC024901.1 | hsa-miR-181b-5p | EPHA3 |
| AC024901.1 | hsa-miR-181b-5p | STMN2 |
| AC024901.1 | hsa-miR-181b-5p | SLIT3 |
| AC024901.1 | hsa-miR-181b-5p | ANGPT1 |
| AC024901.1 | hsa-miR-181b-5p | RANBP17 |
| AC024901.1 | hsa-miR-181b-5p | NAALAD2 |
| AC024901.1 | hsa-miR-181b-5p | TRERF1 |
| AC024901.1 | hsa-miR-181b-5p | CDH13 |
| AC024901.1 | hsa-miR-181b-5p | PPP1R12B |
| LINC00632 | hsa-miR-127-3p | ASPA |
| LINC00632 | hsa-miR-127-3p | CAMK2A |
| LINC00632 | hsa-miR-127-3p | ATP2B2 |
| LINC00632 | hsa-miR-127-3p | CREB3L1 |
| AC068733.3 | hsa-miR-127-3p | ASPA |
| AC068733.3 | hsa-miR-127-3p | CAMK2A |
| AC068733.3 | hsa-miR-127-3p | ATP2B2 |
| AC068733.3 | hsa-miR-127-3p | CREB3L1 |
| AC098679.2 | hsa-miR-127-3p | ASPA |
| AC098679.2 | hsa-miR-127-3p | CAMK2A |
| AC098679.2 | hsa-miR-127-3p | ATP2B2 |
| AC098679.2 | hsa-miR-127-3p | CREB3L1 |
| AP003355.2 | hsa-miR-449b-5p | INMT |
| AP003355.2 | hsa-miR-449b-5p | FSTL3 |
| AP003355.2 | hsa-miR-449b-5p | ADAM11 |
| AP003355.2 | hsa-miR-449b-5p | HOXA13 |
| AP003355.2 | hsa-miR-449b-5p | HLF |
| AP003355.2 | hsa-miR-449b-5p | IGFBP5 |
| AP003355.2 | hsa-miR-449b-5p | NRK |
| AP003355.2 | hsa-miR-449b-5p | PTGIS |
| AP003355.2 | hsa-miR-449b-5p | NRN1 |
| AP003355.2 | hsa-miR-449b-5p | GNAZ |
| AP003355.2 | hsa-miR-449b-5p | GATA5 |
| AP003355.2 | hsa-miR-449b-5p | ABCC9 |
| AP003355.2 | hsa-miR-449b-5p | ASXL3 |
| AP003355.2 | hsa-miR-449b-5p | MYL9 |
| AP003355.2 | hsa-miR-449b-5p | PDLIM3 |
| AP003355.2 | hsa-miR-449b-5p | UNC5D |
| AP003355.2 | hsa-miR-449b-5p | LHCGR |
| AP003355.2 | hsa-miR-449b-5p | FAT3 |
| AP003355.2 | hsa-miR-449b-5p | PRRT2 |
| AP003355.2 | hsa-miR-449b-5p | MYRIP |
| AP003355.2 | hsa-miR-449b-5p | KCNK3 |
| AP003355.2 | hsa-miR-449b-5p | GPR22 |
| AP003355.2 | hsa-miR-449b-5p | SPEG |
| AP003355.2 | hsa-miR-449b-5p | LPP |
| AP003355.2 | hsa-miR-449b-5p | PLAG1 |
| AP003355.2 | hsa-miR-449b-5p | SYT9 |
| AP003355.2 | hsa-miR-449b-5p | DUOX1 |
| AP003355.2 | hsa-miR-449b-5p | CAPN6 |
| AP003355.2 | hsa-miR-449b-5p | DMRTA1 |
| AP003355.2 | hsa-miR-449b-5p | GDNF |
| AP003355.2 | hsa-miR-449b-5p | GRID1 |
| AP003355.2 | hsa-miR-449b-5p | SYNGR1 |
| AP003355.2 | hsa-miR-449b-5p | BCAN |
| AP003355.2 | hsa-miR-449b-5p | KCNH2 |
| AP003355.2 | hsa-miR-449b-5p | WSCD2 |
| AP003355.2 | hsa-miR-449b-5p | C3orf70 |
| AP003355.2 | hsa-miR-449b-5p | TSPAN18 |
| AP003355.2 | hsa-miR-449b-5p | PRELP |
| AP003355.2 | hsa-miR-449b-5p | SNCG |
| AP003355.2 | hsa-miR-449b-5p | DCX |
| AP003355.2 | hsa-miR-449b-5p | ANKRD36 |
| AP003355.2 | hsa-miR-449b-5p | PLN |
| AP003355.2 | hsa-miR-449b-5p | MAP2 |
| AP003355.2 | hsa-miR-449b-5p | MYLK |
| AP003355.2 | hsa-miR-449b-5p | CNR1 |
| AP003355.2 | hsa-miR-449b-5p | LMOD1 |
| AP003355.2 | hsa-miR-449b-5p | DNM3 |
| AP003355.2 | hsa-miR-449b-5p | GPR161 |
| AP003355.2 | hsa-miR-449b-5p | ITGA10 |
| AP003355.2 | hsa-miR-449b-5p | CDR1 |
| AP003355.2 | hsa-miR-449b-5p | SORBS1 |
| AP003355.2 | hsa-miR-449b-5p | SLC25A27 |
| AP003355.2 | hsa-miR-449b-5p | PAK3 |
| AP003355.2 | hsa-miR-449b-5p | IRS4 |
| AP003355.2 | hsa-miR-449b-5p | WISP2 |
| AP003355.2 | hsa-miR-449b-5p | JPH2 |
| AP003355.2 | hsa-miR-449b-5p | NUDT13 |
| AP003355.2 | hsa-miR-449b-5p | RSPO1 |
| AP003355.2 | hsa-miR-449b-5p | FABP3 |
| AP003355.2 | hsa-miR-449b-5p | PRKG1 |
| AP003355.2 | hsa-miR-449b-5p | TRPM3 |
| AP003355.2 | hsa-miR-449b-5p | PKHD1L1 |
| AP003355.2 | hsa-miR-449b-5p | PTCHD1 |
| AP003355.2 | hsa-miR-449b-5p | ADRA1D |
| AP003355.2 | hsa-miR-449b-5p | DCLK1 |
| AP003355.2 | hsa-miR-449b-5p | BNC2 |
| AP003355.2 | hsa-miR-449b-5p | IGF2 |
| AP003355.2 | hsa-miR-449b-5p | CYS1 |
| AP003355.2 | hsa-miR-449b-5p | LGI2 |
| AP003355.2 | hsa-miR-449b-5p | SLC22A3 |
| AP003355.2 | hsa-miR-449b-5p | CDON |
| AP003355.2 | hsa-miR-449b-5p | MYL3 |
| AP003355.2 | hsa-miR-449b-5p | KCNG1 |
| AP003355.2 | hsa-miR-449b-5p | ST8SIA1 |
| AP003355.2 | hsa-miR-449b-5p | RIC3 |
| AP003355.2 | hsa-miR-449b-5p | SRL |
| AP003355.2 | hsa-miR-449b-5p | NFASC |
| AP003355.2 | hsa-miR-449b-5p | SYNC |
| AP003355.2 | hsa-miR-449b-5p | MRVI1 |
| AP003355.2 | hsa-miR-449b-5p | ITGA11 |
| AP003355.2 | hsa-miR-449b-5p | SEPT4 |
| AP003355.2 | hsa-miR-449b-5p | GFRA1 |
| AP003355.2 | hsa-miR-449b-5p | MRGPRF |
| AP003355.2 | hsa-miR-449b-5p | SHISA6 |
| AP003355.2 | hsa-miR-449b-5p | FAM107A |
| AP003355.2 | hsa-miR-449b-5p | KCNMA1 |
| AP003355.2 | hsa-miR-449b-5p | SLC15A2 |
| AP003355.2 | hsa-miR-449b-5p | SLIT3 |
| AP003355.2 | hsa-miR-449b-5p | TMEM200B |
| AP003355.2 | hsa-miR-449b-5p | CREB3L1 |
| AP003355.2 | hsa-miR-449b-5p | TAGLN |
| AP003355.2 | hsa-miR-449b-5p | CES4A |
| AP003355.2 | hsa-miR-449b-5p | FHL5 |
| AP003355.2 | hsa-miR-449b-5p | EML5 |
| AP003355.2 | hsa-miR-449b-5p | DUOXA1 |
| AP003355.2 | hsa-miR-449b-5p | CDH13 |
| AP003355.2 | hsa-miR-449b-5p | PPP1R12B |
| MBNL1-AS1 | hsa-miR-155-5p | TLL1 |
| MBNL1-AS1 | hsa-miR-155-5p | IGFBP5 |
| MBNL1-AS1 | hsa-miR-155-5p | NRK |
| MBNL1-AS1 | hsa-miR-155-5p | PTGIS |
| MBNL1-AS1 | hsa-miR-155-5p | COL21A1 |
| MBNL1-AS1 | hsa-miR-155-5p | HAPLN2 |
| MBNL1-AS1 | hsa-miR-155-5p | RERG |
| MBNL1-AS1 | hsa-miR-155-5p | ABCC9 |
| MBNL1-AS1 | hsa-miR-155-5p | NGEF |
| MBNL1-AS1 | hsa-miR-155-5p | OSR1 |
| MBNL1-AS1 | hsa-miR-155-5p | SLC2A12 |
| MBNL1-AS1 | hsa-miR-155-5p | TMEM47 |
| MBNL1-AS1 | hsa-miR-155-5p | KCNK13 |
| MBNL1-AS1 | hsa-miR-155-5p | RAB3C |
| MBNL1-AS1 | hsa-miR-155-5p | CPA3 |
| MBNL1-AS1 | hsa-miR-155-5p | EMCN |
| MBNL1-AS1 | hsa-miR-155-5p | MYRIP |
| MBNL1-AS1 | hsa-miR-155-5p | SLC38A11 |
| MBNL1-AS1 | hsa-miR-155-5p | GPR22 |
| MBNL1-AS1 | hsa-miR-155-5p | ADH1B |
| MBNL1-AS1 | hsa-miR-155-5p | SCG2 |
| MBNL1-AS1 | hsa-miR-155-5p | LPP |
| MBNL1-AS1 | hsa-miR-155-5p | C7 |
| MBNL1-AS1 | hsa-miR-155-5p | EBF1 |
| MBNL1-AS1 | hsa-miR-155-5p | PLAG1 |
| MBNL1-AS1 | hsa-miR-155-5p | LURAP1L |
| MBNL1-AS1 | hsa-miR-155-5p | KIAA1549L |
| MBNL1-AS1 | hsa-miR-155-5p | DMRTA1 |
| MBNL1-AS1 | hsa-miR-155-5p | C11orf87 |
| MBNL1-AS1 | hsa-miR-155-5p | SLC24A2 |
| MBNL1-AS1 | hsa-miR-155-5p | NR3C2 |
| MBNL1-AS1 | hsa-miR-155-5p | DCX |
| MBNL1-AS1 | hsa-miR-155-5p | PLN |
| MBNL1-AS1 | hsa-miR-155-5p | KLHDC1 |
| MBNL1-AS1 | hsa-miR-155-5p | NFIX |
| MBNL1-AS1 | hsa-miR-155-5p | MAP2 |
| MBNL1-AS1 | hsa-miR-155-5p | MYLK |
| MBNL1-AS1 | hsa-miR-155-5p | ITGA10 |
| MBNL1-AS1 | hsa-miR-155-5p | CDR1 |
| MBNL1-AS1 | hsa-miR-155-5p | KCNB1 |
| MBNL1-AS1 | hsa-miR-155-5p | PAK3 |
| MBNL1-AS1 | hsa-miR-155-5p | CHRDL1 |
| MBNL1-AS1 | hsa-miR-155-5p | HYI |
| MBNL1-AS1 | hsa-miR-155-5p | RAB40A |
| MBNL1-AS1 | hsa-miR-155-5p | NUDT13 |
| MBNL1-AS1 | hsa-miR-155-5p | PRKG1 |
| MBNL1-AS1 | hsa-miR-155-5p | GPR20 |
| MBNL1-AS1 | hsa-miR-155-5p | PTCHD1 |
| MBNL1-AS1 | hsa-miR-155-5p | DCLK1 |
| MBNL1-AS1 | hsa-miR-155-5p | BNC2 |
| MBNL1-AS1 | hsa-miR-155-5p | IGF2 |
| MBNL1-AS1 | hsa-miR-155-5p | LGI2 |
| MBNL1-AS1 | hsa-miR-155-5p | SEMA5A |
| MBNL1-AS1 | hsa-miR-155-5p | CDON |
| MBNL1-AS1 | hsa-miR-155-5p | SPOCK1 |
| MBNL1-AS1 | hsa-miR-155-5p | MAPK10 |
| MBNL1-AS1 | hsa-miR-155-5p | KCNG1 |
| MBNL1-AS1 | hsa-miR-155-5p | ST8SIA1 |
| MBNL1-AS1 | hsa-miR-155-5p | NFASC |
| MBNL1-AS1 | hsa-miR-155-5p | DGKB |
| MBNL1-AS1 | hsa-miR-155-5p | SCN7A |
| MBNL1-AS1 | hsa-miR-155-5p | HSPB7 |
| MBNL1-AS1 | hsa-miR-155-5p | GFRA1 |
| MBNL1-AS1 | hsa-miR-155-5p | KLF8 |
| MBNL1-AS1 | hsa-miR-155-5p | SLC15A2 |
| MBNL1-AS1 | hsa-miR-155-5p | EPHA3 |
| MBNL1-AS1 | hsa-miR-155-5p | STMN2 |
| MBNL1-AS1 | hsa-miR-155-5p | NAALAD2 |
| MBNL1-AS1 | hsa-miR-155-5p | PHEX |
| MBNL1-AS1 | hsa-miR-155-5p | FHL5 |
| MBNL1-AS1 | hsa-miR-155-5p | ANKS1B |
| MBNL1-AS1 | hsa-miR-155-5p | EML5 |
| MBNL1-AS1 | hsa-miR-155-5p | DET1 |
| MBNL1-AS1 | hsa-miR-155-5p | PPP1R12B |
| MBNL1-AS1 | hsa-miR-503-5p | TLL1 |
| MBNL1-AS1 | hsa-miR-503-5p | ADAM11 |
| MBNL1-AS1 | hsa-miR-503-5p | CCDC80 |
| MBNL1-AS1 | hsa-miR-503-5p | RAB9B |
| MBNL1-AS1 | hsa-miR-503-5p | NRN1 |
| MBNL1-AS1 | hsa-miR-503-5p | ATP1B2 |
| MBNL1-AS1 | hsa-miR-503-5p | CASQ2 |
| MBNL1-AS1 | hsa-miR-503-5p | TPM1 |
| MBNL1-AS1 | hsa-miR-503-5p | TMEM47 |
| MBNL1-AS1 | hsa-miR-503-5p | PDLIM3 |
| MBNL1-AS1 | hsa-miR-503-5p | UNC5D |
| MBNL1-AS1 | hsa-miR-503-5p | ACTC1 |
| MBNL1-AS1 | hsa-miR-503-5p | TMEM132B |
| MBNL1-AS1 | hsa-miR-503-5p | MYRIP |
| MBNL1-AS1 | hsa-miR-503-5p | TUB |
| MBNL1-AS1 | hsa-miR-503-5p | LPP |
| MBNL1-AS1 | hsa-miR-503-5p | BVES |
| MBNL1-AS1 | hsa-miR-503-5p | GPR88 |
| MBNL1-AS1 | hsa-miR-503-5p | IL20RA |
| MBNL1-AS1 | hsa-miR-503-5p | PLAG1 |
| MBNL1-AS1 | hsa-miR-503-5p | SLC2A4 |
| MBNL1-AS1 | hsa-miR-503-5p | LURAP1L |
| MBNL1-AS1 | hsa-miR-503-5p | CAPN6 |
| MBNL1-AS1 | hsa-miR-503-5p | ST6GALNAC3 |
| MBNL1-AS1 | hsa-miR-503-5p | KCNH2 |
| MBNL1-AS1 | hsa-miR-503-5p | C3orf70 |
| MBNL1-AS1 | hsa-miR-503-5p | TSPAN18 |
| MBNL1-AS1 | hsa-miR-503-5p | SLC24A2 |
| MBNL1-AS1 | hsa-miR-503-5p | PRELP |
| MBNL1-AS1 | hsa-miR-503-5p | RSPO3 |
| MBNL1-AS1 | hsa-miR-503-5p | MYLK |
| MBNL1-AS1 | hsa-miR-503-5p | RBM20 |
| MBNL1-AS1 | hsa-miR-503-5p | PTGFR |
| MBNL1-AS1 | hsa-miR-503-5p | SORBS1 |
| MBNL1-AS1 | hsa-miR-503-5p | TRPM3 |
| MBNL1-AS1 | hsa-miR-503-5p | MAMDC2 |
| MBNL1-AS1 | hsa-miR-503-5p | DMD |
| MBNL1-AS1 | hsa-miR-503-5p | PTCHD1 |
| MBNL1-AS1 | hsa-miR-503-5p | BNC2 |
| MBNL1-AS1 | hsa-miR-503-5p | IGF2 |
| MBNL1-AS1 | hsa-miR-503-5p | CYS1 |
| MBNL1-AS1 | hsa-miR-503-5p | LGI2 |
| MBNL1-AS1 | hsa-miR-503-5p | CDON |
| MBNL1-AS1 | hsa-miR-503-5p | NT5DC3 |
| MBNL1-AS1 | hsa-miR-503-5p | KALRN |
| MBNL1-AS1 | hsa-miR-503-5p | MYL3 |
| MBNL1-AS1 | hsa-miR-503-5p | MTMR11 |
| MBNL1-AS1 | hsa-miR-503-5p | ADAMTSL5 |
| MBNL1-AS1 | hsa-miR-503-5p | SYNPO2 |
| MBNL1-AS1 | hsa-miR-503-5p | OSR2 |
| MBNL1-AS1 | hsa-miR-503-5p | SHISA6 |
| MBNL1-AS1 | hsa-miR-503-5p | MYH11 |
| MBNL1-AS1 | hsa-miR-503-5p | KLF8 |
| MBNL1-AS1 | hsa-miR-503-5p | SLIT3 |
| MBNL1-AS1 | hsa-miR-503-5p | NAALAD2 |
| MBNL1-AS1 | hsa-miR-503-5p | PHEX |
| MBNL1-AS1 | hsa-miR-503-5p | TRERF1 |
| MBNL1-AS1 | hsa-miR-503-5p | LDB3 |
| MBNL1-AS1 | hsa-miR-503-5p | EML5 |
| MBNL1-AS1 | hsa-miR-503-5p | NDUFA4L2 |
| MBNL1-AS1 | hsa-miR-503-5p | RBFOX3 |
| MBNL1-AS1 | hsa-miR-503-5p | CCDC68 |
| MBNL1-AS1 | hsa-miR-503-5p | PPP1R12B |
| ADIRF-AS1 | hsa-miR-503-5p | TLL1 |
| ADIRF-AS1 | hsa-miR-503-5p | ADAM11 |
| ADIRF-AS1 | hsa-miR-503-5p | CCDC80 |
| ADIRF-AS1 | hsa-miR-503-5p | RAB9B |
| ADIRF-AS1 | hsa-miR-503-5p | NRN1 |
| ADIRF-AS1 | hsa-miR-503-5p | ATP1B2 |
| ADIRF-AS1 | hsa-miR-503-5p | CASQ2 |
| ADIRF-AS1 | hsa-miR-503-5p | TPM1 |
| ADIRF-AS1 | hsa-miR-503-5p | TMEM47 |
| ADIRF-AS1 | hsa-miR-503-5p | PDLIM3 |
| ADIRF-AS1 | hsa-miR-503-5p | UNC5D |
| ADIRF-AS1 | hsa-miR-503-5p | ACTC1 |
| ADIRF-AS1 | hsa-miR-503-5p | TMEM132B |
| ADIRF-AS1 | hsa-miR-503-5p | MYRIP |
| ADIRF-AS1 | hsa-miR-503-5p | TUB |
| ADIRF-AS1 | hsa-miR-503-5p | LPP |
| ADIRF-AS1 | hsa-miR-503-5p | BVES |
| ADIRF-AS1 | hsa-miR-503-5p | GPR88 |
| ADIRF-AS1 | hsa-miR-503-5p | IL20RA |
| ADIRF-AS1 | hsa-miR-503-5p | PLAG1 |
| ADIRF-AS1 | hsa-miR-503-5p | SLC2A4 |
| ADIRF-AS1 | hsa-miR-503-5p | LURAP1L |
| ADIRF-AS1 | hsa-miR-503-5p | CAPN6 |
| ADIRF-AS1 | hsa-miR-503-5p | ST6GALNAC3 |
| ADIRF-AS1 | hsa-miR-503-5p | KCNH2 |
| ADIRF-AS1 | hsa-miR-503-5p | C3orf70 |
| ADIRF-AS1 | hsa-miR-503-5p | TSPAN18 |
| ADIRF-AS1 | hsa-miR-503-5p | SLC24A2 |
| ADIRF-AS1 | hsa-miR-503-5p | PRELP |
| ADIRF-AS1 | hsa-miR-503-5p | RSPO3 |
| ADIRF-AS1 | hsa-miR-503-5p | MYLK |
| ADIRF-AS1 | hsa-miR-503-5p | RBM20 |
| ADIRF-AS1 | hsa-miR-503-5p | PTGFR |
| ADIRF-AS1 | hsa-miR-503-5p | SORBS1 |
| ADIRF-AS1 | hsa-miR-503-5p | TRPM3 |
| ADIRF-AS1 | hsa-miR-503-5p | MAMDC2 |
| ADIRF-AS1 | hsa-miR-503-5p | DMD |
| ADIRF-AS1 | hsa-miR-503-5p | PTCHD1 |
| ADIRF-AS1 | hsa-miR-503-5p | BNC2 |
| ADIRF-AS1 | hsa-miR-503-5p | IGF2 |
| ADIRF-AS1 | hsa-miR-503-5p | CYS1 |
| ADIRF-AS1 | hsa-miR-503-5p | LGI2 |
| ADIRF-AS1 | hsa-miR-503-5p | CDON |
| ADIRF-AS1 | hsa-miR-503-5p | NT5DC3 |
| ADIRF-AS1 | hsa-miR-503-5p | KALRN |
| ADIRF-AS1 | hsa-miR-503-5p | MYL3 |
| ADIRF-AS1 | hsa-miR-503-5p | MTMR11 |
| ADIRF-AS1 | hsa-miR-503-5p | ADAMTSL5 |
| ADIRF-AS1 | hsa-miR-503-5p | SYNPO2 |
| ADIRF-AS1 | hsa-miR-503-5p | OSR2 |
| ADIRF-AS1 | hsa-miR-503-5p | SHISA6 |
| ADIRF-AS1 | hsa-miR-503-5p | MYH11 |
| ADIRF-AS1 | hsa-miR-503-5p | KLF8 |
| ADIRF-AS1 | hsa-miR-503-5p | SLIT3 |
| ADIRF-AS1 | hsa-miR-503-5p | NAALAD2 |
| ADIRF-AS1 | hsa-miR-503-5p | PHEX |
| ADIRF-AS1 | hsa-miR-503-5p | TRERF1 |
| ADIRF-AS1 | hsa-miR-503-5p | LDB3 |
| ADIRF-AS1 | hsa-miR-503-5p | EML5 |
| ADIRF-AS1 | hsa-miR-503-5p | NDUFA4L2 |
| ADIRF-AS1 | hsa-miR-503-5p | RBFOX3 |
| ADIRF-AS1 | hsa-miR-503-5p | CCDC68 |
| ADIRF-AS1 | hsa-miR-503-5p | PPP1R12B |
| AC024901.1 | hsa-miR-503-5p | TLL1 |
| AC024901.1 | hsa-miR-503-5p | ADAM11 |
| AC024901.1 | hsa-miR-503-5p | CCDC80 |
| AC024901.1 | hsa-miR-503-5p | RAB9B |
| AC024901.1 | hsa-miR-503-5p | NRN1 |
| AC024901.1 | hsa-miR-503-5p | ATP1B2 |
| AC024901.1 | hsa-miR-503-5p | CASQ2 |
| AC024901.1 | hsa-miR-503-5p | TPM1 |
| AC024901.1 | hsa-miR-503-5p | TMEM47 |
| AC024901.1 | hsa-miR-503-5p | PDLIM3 |
| AC024901.1 | hsa-miR-503-5p | UNC5D |
| AC024901.1 | hsa-miR-503-5p | ACTC1 |
| AC024901.1 | hsa-miR-503-5p | TMEM132B |
| AC024901.1 | hsa-miR-503-5p | MYRIP |
| AC024901.1 | hsa-miR-503-5p | TUB |
| AC024901.1 | hsa-miR-503-5p | LPP |
| AC024901.1 | hsa-miR-503-5p | BVES |
| AC024901.1 | hsa-miR-503-5p | GPR88 |
| AC024901.1 | hsa-miR-503-5p | IL20RA |
| AC024901.1 | hsa-miR-503-5p | PLAG1 |
| AC024901.1 | hsa-miR-503-5p | SLC2A4 |
| AC024901.1 | hsa-miR-503-5p | LURAP1L |
| AC024901.1 | hsa-miR-503-5p | CAPN6 |
| AC024901.1 | hsa-miR-503-5p | ST6GALNAC3 |
| AC024901.1 | hsa-miR-503-5p | KCNH2 |
| AC024901.1 | hsa-miR-503-5p | C3orf70 |
| AC024901.1 | hsa-miR-503-5p | TSPAN18 |
| AC024901.1 | hsa-miR-503-5p | SLC24A2 |
| AC024901.1 | hsa-miR-503-5p | PRELP |
| AC024901.1 | hsa-miR-503-5p | RSPO3 |
| AC024901.1 | hsa-miR-503-5p | MYLK |
| AC024901.1 | hsa-miR-503-5p | RBM20 |
| AC024901.1 | hsa-miR-503-5p | PTGFR |
| AC024901.1 | hsa-miR-503-5p | SORBS1 |
| AC024901.1 | hsa-miR-503-5p | TRPM3 |
| AC024901.1 | hsa-miR-503-5p | MAMDC2 |
| AC024901.1 | hsa-miR-503-5p | DMD |
| AC024901.1 | hsa-miR-503-5p | PTCHD1 |
| AC024901.1 | hsa-miR-503-5p | BNC2 |
| AC024901.1 | hsa-miR-503-5p | IGF2 |
| AC024901.1 | hsa-miR-503-5p | CYS1 |
| AC024901.1 | hsa-miR-503-5p | LGI2 |
| AC024901.1 | hsa-miR-503-5p | CDON |
| AC024901.1 | hsa-miR-503-5p | NT5DC3 |
| AC024901.1 | hsa-miR-503-5p | KALRN |
| AC024901.1 | hsa-miR-503-5p | MYL3 |
| AC024901.1 | hsa-miR-503-5p | MTMR11 |
| AC024901.1 | hsa-miR-503-5p | ADAMTSL5 |
| AC024901.1 | hsa-miR-503-5p | SYNPO2 |
| AC024901.1 | hsa-miR-503-5p | OSR2 |
| AC024901.1 | hsa-miR-503-5p | SHISA6 |
| AC024901.1 | hsa-miR-503-5p | MYH11 |
| AC024901.1 | hsa-miR-503-5p | KLF8 |
| AC024901.1 | hsa-miR-503-5p | SLIT3 |
| AC024901.1 | hsa-miR-503-5p | NAALAD2 |
| AC024901.1 | hsa-miR-503-5p | PHEX |
| AC024901.1 | hsa-miR-503-5p | TRERF1 |
| AC024901.1 | hsa-miR-503-5p | LDB3 |
| AC024901.1 | hsa-miR-503-5p | EML5 |
| AC024901.1 | hsa-miR-503-5p | NDUFA4L2 |
| AC024901.1 | hsa-miR-503-5p | RBFOX3 |
| AC024901.1 | hsa-miR-503-5p | CCDC68 |
| AC024901.1 | hsa-miR-503-5p | PPP1R12B |
| ACTA2-AS1 | hsa-miR-187-3p | NGEF |
| ACTA2-AS1 | hsa-miR-187-3p | UNC5D |
| ACTA2-AS1 | hsa-miR-187-3p | THNSL2 |
| ACTA2-AS1 | hsa-miR-187-3p | FLNC |
| ACTA2-AS1 | hsa-miR-187-3p | ATP2B2 |
| ACTA2-AS1 | hsa-miR-187-3p | CNR1 |
| ACTA2-AS1 | hsa-miR-187-3p | TRPM3 |
| ACTA2-AS1 | hsa-miR-187-3p | BNC2 |
| ACTA2-AS1 | hsa-miR-187-3p | SEMA5A |
| ACTA2-AS1 | hsa-miR-187-3p | MYH11 |
| ACTA2-AS1 | hsa-miR-187-3p | SLC15A2 |
| ACTA2-AS1 | hsa-miR-187-3p | MEGF10 |
| HRAT92 | hsa-miR-187-3p | NGEF |
| HRAT92 | hsa-miR-187-3p | UNC5D |
| HRAT92 | hsa-miR-187-3p | THNSL2 |
| HRAT92 | hsa-miR-187-3p | FLNC |
| HRAT92 | hsa-miR-187-3p | ATP2B2 |
| HRAT92 | hsa-miR-187-3p | CNR1 |
| HRAT92 | hsa-miR-187-3p | TRPM3 |
| HRAT92 | hsa-miR-187-3p | BNC2 |
| HRAT92 | hsa-miR-187-3p | SEMA5A |
| HRAT92 | hsa-miR-187-3p | MYH11 |
| HRAT92 | hsa-miR-187-3p | SLC15A2 |
| HRAT92 | hsa-miR-187-3p | MEGF10 |
| MIR1-1HG-AS1 | hsa-miR-107 | REEP1 |
| MIR1-1HG-AS1 | hsa-miR-107 | ADAM11 |
| MIR1-1HG-AS1 | hsa-miR-107 | CCDC80 |
| MIR1-1HG-AS1 | hsa-miR-107 | HOXA13 |
| MIR1-1HG-AS1 | hsa-miR-107 | HLF |
| MIR1-1HG-AS1 | hsa-miR-107 | RAB9B |
| MIR1-1HG-AS1 | hsa-miR-107 | NRN1 |
| MIR1-1HG-AS1 | hsa-miR-107 | ATP1B2 |
| MIR1-1HG-AS1 | hsa-miR-107 | ASPA |
| MIR1-1HG-AS1 | hsa-miR-107 | PDE8B |
| MIR1-1HG-AS1 | hsa-miR-107 | TPM1 |
| MIR1-1HG-AS1 | hsa-miR-107 | PTH2R |
| MIR1-1HG-AS1 | hsa-miR-107 | TRPC1 |
| MIR1-1HG-AS1 | hsa-miR-107 | TMEM47 |
| MIR1-1HG-AS1 | hsa-miR-107 | MYL9 |
| MIR1-1HG-AS1 | hsa-miR-107 | RAB3C |
| MIR1-1HG-AS1 | hsa-miR-107 | ACTC1 |
| MIR1-1HG-AS1 | hsa-miR-107 | KCNIP3 |
| MIR1-1HG-AS1 | hsa-miR-107 | COL6A3 |
| MIR1-1HG-AS1 | hsa-miR-107 | NPY1R |
| MIR1-1HG-AS1 | hsa-miR-107 | FAT3 |
| MIR1-1HG-AS1 | hsa-miR-107 | TMEM132B |
| MIR1-1HG-AS1 | hsa-miR-107 | PRRT2 |
| MIR1-1HG-AS1 | hsa-miR-107 | HSPB3 |
| MIR1-1HG-AS1 | hsa-miR-107 | TUB |
| MIR1-1HG-AS1 | hsa-miR-107 | LPP |
| MIR1-1HG-AS1 | hsa-miR-107 | C7 |
| MIR1-1HG-AS1 | hsa-miR-107 | EBF1 |
| MIR1-1HG-AS1 | hsa-miR-107 | BVES |
| MIR1-1HG-AS1 | hsa-miR-107 | CLU |
| MIR1-1HG-AS1 | hsa-miR-107 | PLAG1 |
| MIR1-1HG-AS1 | hsa-miR-107 | SLC2A4 |
| MIR1-1HG-AS1 | hsa-miR-107 | SYT9 |
| MIR1-1HG-AS1 | hsa-miR-107 | DMRTA1 |
| MIR1-1HG-AS1 | hsa-miR-107 | C3orf80 |
| MIR1-1HG-AS1 | hsa-miR-107 | ST6GALNAC3 |
| MIR1-1HG-AS1 | hsa-miR-107 | SYNGR1 |
| MIR1-1HG-AS1 | hsa-miR-107 | TSPAN18 |
| MIR1-1HG-AS1 | hsa-miR-107 | PRELP |
| MIR1-1HG-AS1 | hsa-miR-107 | NR3C2 |
| MIR1-1HG-AS1 | hsa-miR-107 | CAMK2A |
| MIR1-1HG-AS1 | hsa-miR-107 | SNCG |
| MIR1-1HG-AS1 | hsa-miR-107 | MYOM1 |
| MIR1-1HG-AS1 | hsa-miR-107 | RSPO3 |
| MIR1-1HG-AS1 | hsa-miR-107 | DCX |
| MIR1-1HG-AS1 | hsa-miR-107 | ANKRD36 |
| MIR1-1HG-AS1 | hsa-miR-107 | MYLK |
| MIR1-1HG-AS1 | hsa-miR-107 | CNR1 |
| MIR1-1HG-AS1 | hsa-miR-107 | LMOD1 |
| MIR1-1HG-AS1 | hsa-miR-107 | DNM3 |
| MIR1-1HG-AS1 | hsa-miR-107 | ITGA10 |
| MIR1-1HG-AS1 | hsa-miR-107 | RBM20 |
| MIR1-1HG-AS1 | hsa-miR-107 | SYPL2 |
| MIR1-1HG-AS1 | hsa-miR-107 | LDOC1 |
| MIR1-1HG-AS1 | hsa-miR-107 | CDR1 |
| MIR1-1HG-AS1 | hsa-miR-107 | KCNB1 |
| MIR1-1HG-AS1 | hsa-miR-107 | PAK3 |
| MIR1-1HG-AS1 | hsa-miR-107 | CHRDL1 |
| MIR1-1HG-AS1 | hsa-miR-107 | RAB40A |
| MIR1-1HG-AS1 | hsa-miR-107 | JPH2 |
| MIR1-1HG-AS1 | hsa-miR-107 | DES |
| MIR1-1HG-AS1 | hsa-miR-107 | PRKG1 |
| MIR1-1HG-AS1 | hsa-miR-107 | DMD |
| MIR1-1HG-AS1 | hsa-miR-107 | PTCHD1 |
| MIR1-1HG-AS1 | hsa-miR-107 | BNC2 |
| MIR1-1HG-AS1 | hsa-miR-107 | SEMA5A |
| MIR1-1HG-AS1 | hsa-miR-107 | NT5DC3 |
| MIR1-1HG-AS1 | hsa-miR-107 | PRIMA1 |
| MIR1-1HG-AS1 | hsa-miR-107 | ANKRD39 |
| MIR1-1HG-AS1 | hsa-miR-107 | MAPK10 |
| MIR1-1HG-AS1 | hsa-miR-107 | RIC3 |
| MIR1-1HG-AS1 | hsa-miR-107 | NFASC |
| MIR1-1HG-AS1 | hsa-miR-107 | MTMR11 |
| MIR1-1HG-AS1 | hsa-miR-107 | SCN7A |
| MIR1-1HG-AS1 | hsa-miR-107 | MRVI1 |
| MIR1-1HG-AS1 | hsa-miR-107 | ITGA11 |
| MIR1-1HG-AS1 | hsa-miR-107 | OSR2 |
| MIR1-1HG-AS1 | hsa-miR-107 | MRGPRF |
| MIR1-1HG-AS1 | hsa-miR-107 | TCEA3 |
| MIR1-1HG-AS1 | hsa-miR-107 | MYH11 |
| MIR1-1HG-AS1 | hsa-miR-107 | KLF8 |
| MIR1-1HG-AS1 | hsa-miR-107 | ABI3BP |
| MIR1-1HG-AS1 | hsa-miR-107 | ST6GALNAC5 |
| MIR1-1HG-AS1 | hsa-miR-107 | EPHA3 |
| MIR1-1HG-AS1 | hsa-miR-107 | AQP1 |
| MIR1-1HG-AS1 | hsa-miR-107 | TMEM200B |
| MIR1-1HG-AS1 | hsa-miR-107 | PIANP |
| MIR1-1HG-AS1 | hsa-miR-107 | EML5 |
| MIR1-1HG-AS1 | hsa-miR-107 | AHNAK2 |
| MIR1-1HG-AS1 | hsa-miR-107 | CDH13 |
| MIR1-1HG-AS1 | hsa-miR-107 | ADCYAP1 |
| AC024901.1 | hsa-miR-181d-5p | TLL1 |
| AC024901.1 | hsa-miR-181d-5p | REEP1 |
| AC024901.1 | hsa-miR-181d-5p | ADAM11 |
| AC024901.1 | hsa-miR-181d-5p | CCDC80 |
| AC024901.1 | hsa-miR-181d-5p | ACTA2 |
| AC024901.1 | hsa-miR-181d-5p | HLF |
| AC024901.1 | hsa-miR-181d-5p | SLC5A9 |
| AC024901.1 | hsa-miR-181d-5p | NRK |
| AC024901.1 | hsa-miR-181d-5p | PTGIS |
| AC024901.1 | hsa-miR-181d-5p | SCGB1D2 |
| AC024901.1 | hsa-miR-181d-5p | ATP1B2 |
| AC024901.1 | hsa-miR-181d-5p | ASPA |
| AC024901.1 | hsa-miR-181d-5p | BCHE |
| AC024901.1 | hsa-miR-181d-5p | OPRK1 |
| AC024901.1 | hsa-miR-181d-5p | ASXL3 |
| AC024901.1 | hsa-miR-181d-5p | TRPC1 |
| AC024901.1 | hsa-miR-181d-5p | TMEM47 |
| AC024901.1 | hsa-miR-181d-5p | RAB3C |
| AC024901.1 | hsa-miR-181d-5p | PDLIM3 |
| AC024901.1 | hsa-miR-181d-5p | UNC5D |
| AC024901.1 | hsa-miR-181d-5p | ACTC1 |
| AC024901.1 | hsa-miR-181d-5p | HFM1 |
| AC024901.1 | hsa-miR-181d-5p | COL6A3 |
| AC024901.1 | hsa-miR-181d-5p | TCF23 |
| AC024901.1 | hsa-miR-181d-5p | FAT3 |
| AC024901.1 | hsa-miR-181d-5p | TMEM132B |
| AC024901.1 | hsa-miR-181d-5p | HSPB3 |
| AC024901.1 | hsa-miR-181d-5p | VAT1L |
| AC024901.1 | hsa-miR-181d-5p | SLC38A11 |
| AC024901.1 | hsa-miR-181d-5p | GPR37 |
| AC024901.1 | hsa-miR-181d-5p | GPR22 |
| AC024901.1 | hsa-miR-181d-5p | ADH1B |
| AC024901.1 | hsa-miR-181d-5p | TUB |
| AC024901.1 | hsa-miR-181d-5p | MSRB3 |
| AC024901.1 | hsa-miR-181d-5p | GAL3ST3 |
| AC024901.1 | hsa-miR-181d-5p | LPP |
| AC024901.1 | hsa-miR-181d-5p | C7 |
| AC024901.1 | hsa-miR-181d-5p | BVES |
| AC024901.1 | hsa-miR-181d-5p | GPR88 |
| AC024901.1 | hsa-miR-181d-5p | PLAG1 |
| AC024901.1 | hsa-miR-181d-5p | KIAA1549L |
| AC024901.1 | hsa-miR-181d-5p | DMRTA1 |
| AC024901.1 | hsa-miR-181d-5p | C3orf80 |
| AC024901.1 | hsa-miR-181d-5p | C11orf87 |
| AC024901.1 | hsa-miR-181d-5p | GRID1 |
| AC024901.1 | hsa-miR-181d-5p | ST6GALNAC3 |
| AC024901.1 | hsa-miR-181d-5p | WSCD2 |
| AC024901.1 | hsa-miR-181d-5p | CADPS2 |
| AC024901.1 | hsa-miR-181d-5p | TSPAN18 |
| AC024901.1 | hsa-miR-181d-5p | NR3C2 |
| AC024901.1 | hsa-miR-181d-5p | ATP2B2 |
| AC024901.1 | hsa-miR-181d-5p | JPH4 |
| AC024901.1 | hsa-miR-181d-5p | PLN |
| AC024901.1 | hsa-miR-181d-5p | ZNF483 |
| AC024901.1 | hsa-miR-181d-5p | ATL1 |
| AC024901.1 | hsa-miR-181d-5p | MAP2 |
| AC024901.1 | hsa-miR-181d-5p | CNR1 |
| AC024901.1 | hsa-miR-181d-5p | DNM3 |
| AC024901.1 | hsa-miR-181d-5p | PBX1 |
| AC024901.1 | hsa-miR-181d-5p | SYPL2 |
| AC024901.1 | hsa-miR-181d-5p | PTGER3 |
| AC024901.1 | hsa-miR-181d-5p | SLC25A27 |
| AC024901.1 | hsa-miR-181d-5p | KCNB1 |
| AC024901.1 | hsa-miR-181d-5p | IRS4 |
| AC024901.1 | hsa-miR-181d-5p | RAB40A |
| AC024901.1 | hsa-miR-181d-5p | PRKG1 |
| AC024901.1 | hsa-miR-181d-5p | TRPM3 |
| AC024901.1 | hsa-miR-181d-5p | MAMDC2 |
| AC024901.1 | hsa-miR-181d-5p | PTCHD1 |
| AC024901.1 | hsa-miR-181d-5p | DCLK1 |
| AC024901.1 | hsa-miR-181d-5p | BNC2 |
| AC024901.1 | hsa-miR-181d-5p | IGF2 |
| AC024901.1 | hsa-miR-181d-5p | LGI2 |
| AC024901.1 | hsa-miR-181d-5p | RYR3 |
| AC024901.1 | hsa-miR-181d-5p | SLC22A3 |
| AC024901.1 | hsa-miR-181d-5p | CDON |
| AC024901.1 | hsa-miR-181d-5p | NT5DC3 |
| AC024901.1 | hsa-miR-181d-5p | PRIMA1 |
| AC024901.1 | hsa-miR-181d-5p | SPOCK1 |
| AC024901.1 | hsa-miR-181d-5p | FAM13A |
| AC024901.1 | hsa-miR-181d-5p | MAPK10 |
| AC024901.1 | hsa-miR-181d-5p | KCNG1 |
| AC024901.1 | hsa-miR-181d-5p | ST8SIA1 |
| AC024901.1 | hsa-miR-181d-5p | RIC3 |
| AC024901.1 | hsa-miR-181d-5p | MEIS2 |
| AC024901.1 | hsa-miR-181d-5p | DGKB |
| AC024901.1 | hsa-miR-181d-5p | SYNC |
| AC024901.1 | hsa-miR-181d-5p | ITGA11 |
| AC024901.1 | hsa-miR-181d-5p | SYNPO2 |
| AC024901.1 | hsa-miR-181d-5p | GFRA1 |
| AC024901.1 | hsa-miR-181d-5p | SHISA6 |
| AC024901.1 | hsa-miR-181d-5p | FAM107A |
| AC024901.1 | hsa-miR-181d-5p | KLF8 |
| AC024901.1 | hsa-miR-181d-5p | ABI3BP |
| AC024901.1 | hsa-miR-181d-5p | ST6GALNAC5 |
| AC024901.1 | hsa-miR-181d-5p | FSBP |
| AC024901.1 | hsa-miR-181d-5p | EPHA3 |
| AC024901.1 | hsa-miR-181d-5p | STMN2 |
| AC024901.1 | hsa-miR-181d-5p | SLIT3 |
| AC024901.1 | hsa-miR-181d-5p | ANGPT1 |
| AC024901.1 | hsa-miR-181d-5p | RANBP17 |
| AC024901.1 | hsa-miR-181d-5p | NAALAD2 |
| AC024901.1 | hsa-miR-181d-5p | TRERF1 |
| AC024901.1 | hsa-miR-181d-5p | CDH13 |
| AC024901.1 | hsa-miR-181d-5p | PPP1R12B |
| MIR1-1HG-AS1 | hsa-miR-151a-5p | HLF |
| MIR1-1HG-AS1 | hsa-miR-151a-5p | ACTC1 |
| MIR1-1HG-AS1 | hsa-miR-151a-5p | LHCGR |
| MIR1-1HG-AS1 | hsa-miR-151a-5p | GPR37 |
| MIR1-1HG-AS1 | hsa-miR-151a-5p | CPLX1 |
| MIR1-1HG-AS1 | hsa-miR-151a-5p | SYNGR1 |
| MIR1-1HG-AS1 | hsa-miR-151a-5p | C3orf70 |
| MIR1-1HG-AS1 | hsa-miR-151a-5p | PRELP |
| MIR1-1HG-AS1 | hsa-miR-151a-5p | NFIX |
| MIR1-1HG-AS1 | hsa-miR-151a-5p | ITGA10 |
| MIR1-1HG-AS1 | hsa-miR-151a-5p | BEND6 |
| MIR1-1HG-AS1 | hsa-miR-151a-5p | ADIRF |
| MIR1-1HG-AS1 | hsa-miR-151a-5p | SLC6A9 |
| MIR1-1HG-AS1 | hsa-miR-151a-5p | PTCHD1 |
| MIR1-1HG-AS1 | hsa-miR-151a-5p | IGF2 |
| MIR1-1HG-AS1 | hsa-miR-151a-5p | CDON |
| MIR1-1HG-AS1 | hsa-miR-151a-5p | HSPB7 |
| MIR1-1HG-AS1 | hsa-miR-151a-5p | SEPT4 |
| MIR1-1HG-AS1 | hsa-miR-151a-5p | GFRA1 |
| MIR1-1HG-AS1 | hsa-miR-151a-5p | MRGPRF |
| MIR1-1HG-AS1 | hsa-miR-151a-5p | TCEA3 |
| MIR1-1HG-AS1 | hsa-miR-151a-5p | PPP1R12B |
| AC068733.3 | hsa-miR-151a-5p | HLF |
| AC068733.3 | hsa-miR-151a-5p | ACTC1 |
| AC068733.3 | hsa-miR-151a-5p | LHCGR |
| AC068733.3 | hsa-miR-151a-5p | GPR37 |
| AC068733.3 | hsa-miR-151a-5p | CPLX1 |
| AC068733.3 | hsa-miR-151a-5p | SYNGR1 |
| AC068733.3 | hsa-miR-151a-5p | C3orf70 |
| AC068733.3 | hsa-miR-151a-5p | PRELP |
| AC068733.3 | hsa-miR-151a-5p | NFIX |
| AC068733.3 | hsa-miR-151a-5p | ITGA10 |
| AC068733.3 | hsa-miR-151a-5p | BEND6 |
| AC068733.3 | hsa-miR-151a-5p | ADIRF |
| AC068733.3 | hsa-miR-151a-5p | SLC6A9 |
| AC068733.3 | hsa-miR-151a-5p | PTCHD1 |
| AC068733.3 | hsa-miR-151a-5p | IGF2 |
| AC068733.3 | hsa-miR-151a-5p | CDON |
| AC068733.3 | hsa-miR-151a-5p | HSPB7 |
| AC068733.3 | hsa-miR-151a-5p | SEPT4 |
| AC068733.3 | hsa-miR-151a-5p | GFRA1 |
| AC068733.3 | hsa-miR-151a-5p | MRGPRF |
| AC068733.3 | hsa-miR-151a-5p | TCEA3 |
| AC068733.3 | hsa-miR-151a-5p | PPP1R12B |
| PGM5-AS1 | hsa-miR-185-5p | REEP1 |
| PGM5-AS1 | hsa-miR-185-5p | REM1 |
| PGM5-AS1 | hsa-miR-185-5p | CCDC80 |
| PGM5-AS1 | hsa-miR-185-5p | HOXA13 |
| PGM5-AS1 | hsa-miR-185-5p | HLF |
| PGM5-AS1 | hsa-miR-185-5p | IGFBP5 |
| PGM5-AS1 | hsa-miR-185-5p | RAB9B |
| PGM5-AS1 | hsa-miR-185-5p | SCGB1D2 |
| PGM5-AS1 | hsa-miR-185-5p | GNAZ |
| PGM5-AS1 | hsa-miR-185-5p | MCHR1 |
| PGM5-AS1 | hsa-miR-185-5p | ATP1B2 |
| PGM5-AS1 | hsa-miR-185-5p | HAPLN2 |
| PGM5-AS1 | hsa-miR-185-5p | CASQ2 |
| PGM5-AS1 | hsa-miR-185-5p | ASPA |
| PGM5-AS1 | hsa-miR-185-5p | PDE8B |
| PGM5-AS1 | hsa-miR-185-5p | OSR1 |
| PGM5-AS1 | hsa-miR-185-5p | SLC2A12 |
| PGM5-AS1 | hsa-miR-185-5p | UNC5D |
| PGM5-AS1 | hsa-miR-185-5p | TCF23 |
| PGM5-AS1 | hsa-miR-185-5p | MASP1 |
| PGM5-AS1 | hsa-miR-185-5p | EMCN |
| PGM5-AS1 | hsa-miR-185-5p | TMEM132B |
| PGM5-AS1 | hsa-miR-185-5p | PRRT2 |
| PGM5-AS1 | hsa-miR-185-5p | VAT1L |
| PGM5-AS1 | hsa-miR-185-5p | KCNK3 |
| PGM5-AS1 | hsa-miR-185-5p | SLC38A11 |
| PGM5-AS1 | hsa-miR-185-5p | GPR37 |
| PGM5-AS1 | hsa-miR-185-5p | CPLX1 |
| PGM5-AS1 | hsa-miR-185-5p | GPR22 |
| PGM5-AS1 | hsa-miR-185-5p | TUB |
| PGM5-AS1 | hsa-miR-185-5p | MSRB3 |
| PGM5-AS1 | hsa-miR-185-5p | LPP |
| PGM5-AS1 | hsa-miR-185-5p | EBF1 |
| PGM5-AS1 | hsa-miR-185-5p | CLU |
| PGM5-AS1 | hsa-miR-185-5p | NCAM1 |
| PGM5-AS1 | hsa-miR-185-5p | FIBIN |
| PGM5-AS1 | hsa-miR-185-5p | ALOX12B |
| PGM5-AS1 | hsa-miR-185-5p | TNFAIP8L3 |
| PGM5-AS1 | hsa-miR-185-5p | GRID1 |
| PGM5-AS1 | hsa-miR-185-5p | PCP4 |
| PGM5-AS1 | hsa-miR-185-5p | SYNGR1 |
| PGM5-AS1 | hsa-miR-185-5p | WSCD2 |
| PGM5-AS1 | hsa-miR-185-5p | CADPS2 |
| PGM5-AS1 | hsa-miR-185-5p | C3orf70 |
| PGM5-AS1 | hsa-miR-185-5p | TSPAN18 |
| PGM5-AS1 | hsa-miR-185-5p | SLC24A2 |
| PGM5-AS1 | hsa-miR-185-5p | PRELP |
| PGM5-AS1 | hsa-miR-185-5p | CAMK2A |
| PGM5-AS1 | hsa-miR-185-5p | ATP2B2 |
| PGM5-AS1 | hsa-miR-185-5p | JPH4 |
| PGM5-AS1 | hsa-miR-185-5p | ADAM33 |
| PGM5-AS1 | hsa-miR-185-5p | DCX |
| PGM5-AS1 | hsa-miR-185-5p | PLN |
| PGM5-AS1 | hsa-miR-185-5p | KLHDC1 |
| PGM5-AS1 | hsa-miR-185-5p | NFIX |
| PGM5-AS1 | hsa-miR-185-5p | MAP2 |
| PGM5-AS1 | hsa-miR-185-5p | MYLK |
| PGM5-AS1 | hsa-miR-185-5p | CNR1 |
| PGM5-AS1 | hsa-miR-185-5p | LMOD1 |
| PGM5-AS1 | hsa-miR-185-5p | PBX1 |
| PGM5-AS1 | hsa-miR-185-5p | RBM20 |
| PGM5-AS1 | hsa-miR-185-5p | SYPL2 |
| PGM5-AS1 | hsa-miR-185-5p | CDR1 |
| PGM5-AS1 | hsa-miR-185-5p | PTGFR |
| PGM5-AS1 | hsa-miR-185-5p | KCNB1 |
| PGM5-AS1 | hsa-miR-185-5p | PAK3 |
| PGM5-AS1 | hsa-miR-185-5p | CHRDL1 |
| PGM5-AS1 | hsa-miR-185-5p | WISP2 |
| PGM5-AS1 | hsa-miR-185-5p | FABP3 |
| PGM5-AS1 | hsa-miR-185-5p | TRPM3 |
| PGM5-AS1 | hsa-miR-185-5p | HIF3A |
| PGM5-AS1 | hsa-miR-185-5p | GPR20 |
| PGM5-AS1 | hsa-miR-185-5p | PTCHD1 |
| PGM5-AS1 | hsa-miR-185-5p | BNC2 |
| PGM5-AS1 | hsa-miR-185-5p | SMOC1 |
| PGM5-AS1 | hsa-miR-185-5p | IGF2 |
| PGM5-AS1 | hsa-miR-185-5p | CYS1 |
| PGM5-AS1 | hsa-miR-185-5p | SEMA5A |
| PGM5-AS1 | hsa-miR-185-5p | SLC22A3 |
| PGM5-AS1 | hsa-miR-185-5p | CDON |
| PGM5-AS1 | hsa-miR-185-5p | SPOCK1 |
| PGM5-AS1 | hsa-miR-185-5p | MYL3 |
| PGM5-AS1 | hsa-miR-185-5p | RIC3 |
| PGM5-AS1 | hsa-miR-185-5p | DGKB |
| PGM5-AS1 | hsa-miR-185-5p | LIMS2 |
| PGM5-AS1 | hsa-miR-185-5p | SCN7A |
| PGM5-AS1 | hsa-miR-185-5p | HSPB7 |
| PGM5-AS1 | hsa-miR-185-5p | ADAMTSL5 |
| PGM5-AS1 | hsa-miR-185-5p | MRVI1 |
| PGM5-AS1 | hsa-miR-185-5p | OSR2 |
| PGM5-AS1 | hsa-miR-185-5p | GFRA1 |
| PGM5-AS1 | hsa-miR-185-5p | MRGPRF |
| PGM5-AS1 | hsa-miR-185-5p | SHISA6 |
| PGM5-AS1 | hsa-miR-185-5p | CMYA5 |
| PGM5-AS1 | hsa-miR-185-5p | TCEA3 |
| PGM5-AS1 | hsa-miR-185-5p | MUC15 |
| PGM5-AS1 | hsa-miR-185-5p | FAM107A |
| PGM5-AS1 | hsa-miR-185-5p | KLF8 |
| PGM5-AS1 | hsa-miR-185-5p | TCEAL4 |
| PGM5-AS1 | hsa-miR-185-5p | ST6GALNAC5 |
| PGM5-AS1 | hsa-miR-185-5p | LDB2 |
| PGM5-AS1 | hsa-miR-185-5p | SORCS2 |
| PGM5-AS1 | hsa-miR-185-5p | SLIT3 |
| PGM5-AS1 | hsa-miR-185-5p | TMEM200B |
| PGM5-AS1 | hsa-miR-185-5p | RANBP17 |
| PGM5-AS1 | hsa-miR-185-5p | PIANP |
| PGM5-AS1 | hsa-miR-185-5p | CES4A |
| PGM5-AS1 | hsa-miR-185-5p | FHL5 |
| PGM5-AS1 | hsa-miR-185-5p | TRERF1 |
| PGM5-AS1 | hsa-miR-185-5p | DUOXA1 |
| PGM5-AS1 | hsa-miR-185-5p | CDH13 |
| PGM5-AS1 | hsa-miR-185-5p | RBFOX3 |
| PGM5-AS1 | hsa-miR-185-5p | AOC3 |
| PGM5-AS1 | hsa-miR-185-5p | PPP1R12B |
| TRHDE-AS1 | hsa-miR-185-5p | REEP1 |
| TRHDE-AS1 | hsa-miR-185-5p | REM1 |
| TRHDE-AS1 | hsa-miR-185-5p | CCDC80 |
| TRHDE-AS1 | hsa-miR-185-5p | HOXA13 |
| TRHDE-AS1 | hsa-miR-185-5p | HLF |
| TRHDE-AS1 | hsa-miR-185-5p | IGFBP5 |
| TRHDE-AS1 | hsa-miR-185-5p | RAB9B |
| TRHDE-AS1 | hsa-miR-185-5p | SCGB1D2 |
| TRHDE-AS1 | hsa-miR-185-5p | GNAZ |
| TRHDE-AS1 | hsa-miR-185-5p | MCHR1 |
| TRHDE-AS1 | hsa-miR-185-5p | ATP1B2 |
| TRHDE-AS1 | hsa-miR-185-5p | HAPLN2 |
| TRHDE-AS1 | hsa-miR-185-5p | CASQ2 |
| TRHDE-AS1 | hsa-miR-185-5p | ASPA |
| TRHDE-AS1 | hsa-miR-185-5p | PDE8B |
| TRHDE-AS1 | hsa-miR-185-5p | OSR1 |
| TRHDE-AS1 | hsa-miR-185-5p | SLC2A12 |
| TRHDE-AS1 | hsa-miR-185-5p | UNC5D |
| TRHDE-AS1 | hsa-miR-185-5p | TCF23 |
| TRHDE-AS1 | hsa-miR-185-5p | MASP1 |
| TRHDE-AS1 | hsa-miR-185-5p | EMCN |
| TRHDE-AS1 | hsa-miR-185-5p | TMEM132B |
| TRHDE-AS1 | hsa-miR-185-5p | PRRT2 |
| TRHDE-AS1 | hsa-miR-185-5p | VAT1L |
| TRHDE-AS1 | hsa-miR-185-5p | KCNK3 |
| TRHDE-AS1 | hsa-miR-185-5p | SLC38A11 |
| TRHDE-AS1 | hsa-miR-185-5p | GPR37 |
| TRHDE-AS1 | hsa-miR-185-5p | CPLX1 |
| TRHDE-AS1 | hsa-miR-185-5p | GPR22 |
| TRHDE-AS1 | hsa-miR-185-5p | TUB |
| TRHDE-AS1 | hsa-miR-185-5p | MSRB3 |
| TRHDE-AS1 | hsa-miR-185-5p | LPP |
| TRHDE-AS1 | hsa-miR-185-5p | EBF1 |
| TRHDE-AS1 | hsa-miR-185-5p | CLU |
| TRHDE-AS1 | hsa-miR-185-5p | NCAM1 |
| TRHDE-AS1 | hsa-miR-185-5p | FIBIN |
| TRHDE-AS1 | hsa-miR-185-5p | ALOX12B |
| TRHDE-AS1 | hsa-miR-185-5p | TNFAIP8L3 |
| TRHDE-AS1 | hsa-miR-185-5p | GRID1 |
| TRHDE-AS1 | hsa-miR-185-5p | PCP4 |
| TRHDE-AS1 | hsa-miR-185-5p | SYNGR1 |
| TRHDE-AS1 | hsa-miR-185-5p | WSCD2 |
| TRHDE-AS1 | hsa-miR-185-5p | CADPS2 |
| TRHDE-AS1 | hsa-miR-185-5p | C3orf70 |
| TRHDE-AS1 | hsa-miR-185-5p | TSPAN18 |
| TRHDE-AS1 | hsa-miR-185-5p | SLC24A2 |
| TRHDE-AS1 | hsa-miR-185-5p | PRELP |
| TRHDE-AS1 | hsa-miR-185-5p | CAMK2A |
| TRHDE-AS1 | hsa-miR-185-5p | ATP2B2 |
| TRHDE-AS1 | hsa-miR-185-5p | JPH4 |
| TRHDE-AS1 | hsa-miR-185-5p | ADAM33 |
| TRHDE-AS1 | hsa-miR-185-5p | DCX |
| TRHDE-AS1 | hsa-miR-185-5p | PLN |
| TRHDE-AS1 | hsa-miR-185-5p | KLHDC1 |
| TRHDE-AS1 | hsa-miR-185-5p | NFIX |
| TRHDE-AS1 | hsa-miR-185-5p | MAP2 |
| TRHDE-AS1 | hsa-miR-185-5p | MYLK |
| TRHDE-AS1 | hsa-miR-185-5p | CNR1 |
| TRHDE-AS1 | hsa-miR-185-5p | LMOD1 |
| TRHDE-AS1 | hsa-miR-185-5p | PBX1 |
| TRHDE-AS1 | hsa-miR-185-5p | RBM20 |
| TRHDE-AS1 | hsa-miR-185-5p | SYPL2 |
| TRHDE-AS1 | hsa-miR-185-5p | CDR1 |
| TRHDE-AS1 | hsa-miR-185-5p | PTGFR |
| TRHDE-AS1 | hsa-miR-185-5p | KCNB1 |
| TRHDE-AS1 | hsa-miR-185-5p | PAK3 |
| TRHDE-AS1 | hsa-miR-185-5p | CHRDL1 |
| TRHDE-AS1 | hsa-miR-185-5p | WISP2 |
| TRHDE-AS1 | hsa-miR-185-5p | FABP3 |
| TRHDE-AS1 | hsa-miR-185-5p | TRPM3 |
| TRHDE-AS1 | hsa-miR-185-5p | HIF3A |
| TRHDE-AS1 | hsa-miR-185-5p | GPR20 |
| TRHDE-AS1 | hsa-miR-185-5p | PTCHD1 |
| TRHDE-AS1 | hsa-miR-185-5p | BNC2 |
| TRHDE-AS1 | hsa-miR-185-5p | SMOC1 |
| TRHDE-AS1 | hsa-miR-185-5p | IGF2 |
| TRHDE-AS1 | hsa-miR-185-5p | CYS1 |
| TRHDE-AS1 | hsa-miR-185-5p | SEMA5A |
| TRHDE-AS1 | hsa-miR-185-5p | SLC22A3 |
| TRHDE-AS1 | hsa-miR-185-5p | CDON |
| TRHDE-AS1 | hsa-miR-185-5p | SPOCK1 |
| TRHDE-AS1 | hsa-miR-185-5p | MYL3 |
| TRHDE-AS1 | hsa-miR-185-5p | RIC3 |
| TRHDE-AS1 | hsa-miR-185-5p | DGKB |
| TRHDE-AS1 | hsa-miR-185-5p | LIMS2 |
| TRHDE-AS1 | hsa-miR-185-5p | SCN7A |
| TRHDE-AS1 | hsa-miR-185-5p | HSPB7 |
| TRHDE-AS1 | hsa-miR-185-5p | ADAMTSL5 |
| TRHDE-AS1 | hsa-miR-185-5p | MRVI1 |
| TRHDE-AS1 | hsa-miR-185-5p | OSR2 |
| TRHDE-AS1 | hsa-miR-185-5p | GFRA1 |
| TRHDE-AS1 | hsa-miR-185-5p | MRGPRF |
| TRHDE-AS1 | hsa-miR-185-5p | SHISA6 |
| TRHDE-AS1 | hsa-miR-185-5p | CMYA5 |
| TRHDE-AS1 | hsa-miR-185-5p | TCEA3 |
| TRHDE-AS1 | hsa-miR-185-5p | MUC15 |
| TRHDE-AS1 | hsa-miR-185-5p | FAM107A |
| TRHDE-AS1 | hsa-miR-185-5p | KLF8 |
| TRHDE-AS1 | hsa-miR-185-5p | TCEAL4 |
| TRHDE-AS1 | hsa-miR-185-5p | ST6GALNAC5 |
| TRHDE-AS1 | hsa-miR-185-5p | LDB2 |
| TRHDE-AS1 | hsa-miR-185-5p | SORCS2 |
| TRHDE-AS1 | hsa-miR-185-5p | SLIT3 |
| TRHDE-AS1 | hsa-miR-185-5p | TMEM200B |
| TRHDE-AS1 | hsa-miR-185-5p | RANBP17 |
| TRHDE-AS1 | hsa-miR-185-5p | PIANP |
| TRHDE-AS1 | hsa-miR-185-5p | CES4A |
| TRHDE-AS1 | hsa-miR-185-5p | FHL5 |
| TRHDE-AS1 | hsa-miR-185-5p | TRERF1 |
| TRHDE-AS1 | hsa-miR-185-5p | DUOXA1 |
| TRHDE-AS1 | hsa-miR-185-5p | CDH13 |
| TRHDE-AS1 | hsa-miR-185-5p | RBFOX3 |
| TRHDE-AS1 | hsa-miR-185-5p | AOC3 |
| TRHDE-AS1 | hsa-miR-185-5p | PPP1R12B |
| IGKV3-20 | hsa-miR-185-5p | REEP1 |
| IGKV3-20 | hsa-miR-185-5p | REM1 |
| IGKV3-20 | hsa-miR-185-5p | CCDC80 |
| IGKV3-20 | hsa-miR-185-5p | HOXA13 |
| IGKV3-20 | hsa-miR-185-5p | HLF |
| IGKV3-20 | hsa-miR-185-5p | IGFBP5 |
| IGKV3-20 | hsa-miR-185-5p | RAB9B |
| IGKV3-20 | hsa-miR-185-5p | SCGB1D2 |
| IGKV3-20 | hsa-miR-185-5p | GNAZ |
| IGKV3-20 | hsa-miR-185-5p | MCHR1 |
| IGKV3-20 | hsa-miR-185-5p | ATP1B2 |
| IGKV3-20 | hsa-miR-185-5p | HAPLN2 |
| IGKV3-20 | hsa-miR-185-5p | CASQ2 |
| IGKV3-20 | hsa-miR-185-5p | ASPA |
| IGKV3-20 | hsa-miR-185-5p | PDE8B |
| IGKV3-20 | hsa-miR-185-5p | OSR1 |
| IGKV3-20 | hsa-miR-185-5p | SLC2A12 |
| IGKV3-20 | hsa-miR-185-5p | UNC5D |
| IGKV3-20 | hsa-miR-185-5p | TCF23 |
| IGKV3-20 | hsa-miR-185-5p | MASP1 |
| IGKV3-20 | hsa-miR-185-5p | EMCN |
| IGKV3-20 | hsa-miR-185-5p | TMEM132B |
| IGKV3-20 | hsa-miR-185-5p | PRRT2 |
| IGKV3-20 | hsa-miR-185-5p | VAT1L |
| IGKV3-20 | hsa-miR-185-5p | KCNK3 |
| IGKV3-20 | hsa-miR-185-5p | SLC38A11 |
| IGKV3-20 | hsa-miR-185-5p | GPR37 |
| IGKV3-20 | hsa-miR-185-5p | CPLX1 |
| IGKV3-20 | hsa-miR-185-5p | GPR22 |
| IGKV3-20 | hsa-miR-185-5p | TUB |
| IGKV3-20 | hsa-miR-185-5p | MSRB3 |
| IGKV3-20 | hsa-miR-185-5p | LPP |
| IGKV3-20 | hsa-miR-185-5p | EBF1 |
| IGKV3-20 | hsa-miR-185-5p | CLU |
| IGKV3-20 | hsa-miR-185-5p | NCAM1 |
| IGKV3-20 | hsa-miR-185-5p | FIBIN |
| IGKV3-20 | hsa-miR-185-5p | ALOX12B |
| IGKV3-20 | hsa-miR-185-5p | TNFAIP8L3 |
| IGKV3-20 | hsa-miR-185-5p | GRID1 |
| IGKV3-20 | hsa-miR-185-5p | PCP4 |
| IGKV3-20 | hsa-miR-185-5p | SYNGR1 |
| IGKV3-20 | hsa-miR-185-5p | WSCD2 |
| IGKV3-20 | hsa-miR-185-5p | CADPS2 |
| IGKV3-20 | hsa-miR-185-5p | C3orf70 |
| IGKV3-20 | hsa-miR-185-5p | TSPAN18 |
| IGKV3-20 | hsa-miR-185-5p | SLC24A2 |
| IGKV3-20 | hsa-miR-185-5p | PRELP |
| IGKV3-20 | hsa-miR-185-5p | CAMK2A |
| IGKV3-20 | hsa-miR-185-5p | ATP2B2 |
| IGKV3-20 | hsa-miR-185-5p | JPH4 |
| IGKV3-20 | hsa-miR-185-5p | ADAM33 |
| IGKV3-20 | hsa-miR-185-5p | DCX |
| IGKV3-20 | hsa-miR-185-5p | PLN |
| IGKV3-20 | hsa-miR-185-5p | KLHDC1 |
| IGKV3-20 | hsa-miR-185-5p | NFIX |
| IGKV3-20 | hsa-miR-185-5p | MAP2 |
| IGKV3-20 | hsa-miR-185-5p | MYLK |
| IGKV3-20 | hsa-miR-185-5p | CNR1 |
| IGKV3-20 | hsa-miR-185-5p | LMOD1 |
| IGKV3-20 | hsa-miR-185-5p | PBX1 |
| IGKV3-20 | hsa-miR-185-5p | RBM20 |
| IGKV3-20 | hsa-miR-185-5p | SYPL2 |
| IGKV3-20 | hsa-miR-185-5p | CDR1 |
| IGKV3-20 | hsa-miR-185-5p | PTGFR |
| IGKV3-20 | hsa-miR-185-5p | KCNB1 |
| IGKV3-20 | hsa-miR-185-5p | PAK3 |
| IGKV3-20 | hsa-miR-185-5p | CHRDL1 |
| IGKV3-20 | hsa-miR-185-5p | WISP2 |
| IGKV3-20 | hsa-miR-185-5p | FABP3 |
| IGKV3-20 | hsa-miR-185-5p | TRPM3 |
| IGKV3-20 | hsa-miR-185-5p | HIF3A |
| IGKV3-20 | hsa-miR-185-5p | GPR20 |
| IGKV3-20 | hsa-miR-185-5p | PTCHD1 |
| IGKV3-20 | hsa-miR-185-5p | BNC2 |
| IGKV3-20 | hsa-miR-185-5p | SMOC1 |
| IGKV3-20 | hsa-miR-185-5p | IGF2 |
| IGKV3-20 | hsa-miR-185-5p | CYS1 |
| IGKV3-20 | hsa-miR-185-5p | SEMA5A |
| IGKV3-20 | hsa-miR-185-5p | SLC22A3 |
| IGKV3-20 | hsa-miR-185-5p | CDON |
| IGKV3-20 | hsa-miR-185-5p | SPOCK1 |
| IGKV3-20 | hsa-miR-185-5p | MYL3 |
| IGKV3-20 | hsa-miR-185-5p | RIC3 |
| IGKV3-20 | hsa-miR-185-5p | DGKB |
| IGKV3-20 | hsa-miR-185-5p | LIMS2 |
| IGKV3-20 | hsa-miR-185-5p | SCN7A |
| IGKV3-20 | hsa-miR-185-5p | HSPB7 |
| IGKV3-20 | hsa-miR-185-5p | ADAMTSL5 |
| IGKV3-20 | hsa-miR-185-5p | MRVI1 |
| IGKV3-20 | hsa-miR-185-5p | OSR2 |
| IGKV3-20 | hsa-miR-185-5p | GFRA1 |
| IGKV3-20 | hsa-miR-185-5p | MRGPRF |
| IGKV3-20 | hsa-miR-185-5p | SHISA6 |
| IGKV3-20 | hsa-miR-185-5p | CMYA5 |
| IGKV3-20 | hsa-miR-185-5p | TCEA3 |
| IGKV3-20 | hsa-miR-185-5p | MUC15 |
| IGKV3-20 | hsa-miR-185-5p | FAM107A |
| IGKV3-20 | hsa-miR-185-5p | KLF8 |
| IGKV3-20 | hsa-miR-185-5p | TCEAL4 |
| IGKV3-20 | hsa-miR-185-5p | ST6GALNAC5 |
| IGKV3-20 | hsa-miR-185-5p | LDB2 |
| IGKV3-20 | hsa-miR-185-5p | SORCS2 |
| IGKV3-20 | hsa-miR-185-5p | SLIT3 |
| IGKV3-20 | hsa-miR-185-5p | TMEM200B |
| IGKV3-20 | hsa-miR-185-5p | RANBP17 |
| IGKV3-20 | hsa-miR-185-5p | PIANP |
| IGKV3-20 | hsa-miR-185-5p | CES4A |
| IGKV3-20 | hsa-miR-185-5p | FHL5 |
| IGKV3-20 | hsa-miR-185-5p | TRERF1 |
| IGKV3-20 | hsa-miR-185-5p | DUOXA1 |
| IGKV3-20 | hsa-miR-185-5p | CDH13 |
| IGKV3-20 | hsa-miR-185-5p | RBFOX3 |
| IGKV3-20 | hsa-miR-185-5p | AOC3 |
| IGKV3-20 | hsa-miR-185-5p | PPP1R12B |
| DIO3OS | hsa-miR-185-5p | REEP1 |
| DIO3OS | hsa-miR-185-5p | REM1 |
| DIO3OS | hsa-miR-185-5p | CCDC80 |
| DIO3OS | hsa-miR-185-5p | HOXA13 |
| DIO3OS | hsa-miR-185-5p | HLF |
| DIO3OS | hsa-miR-185-5p | IGFBP5 |
| DIO3OS | hsa-miR-185-5p | RAB9B |
| DIO3OS | hsa-miR-185-5p | SCGB1D2 |
| DIO3OS | hsa-miR-185-5p | GNAZ |
| DIO3OS | hsa-miR-185-5p | MCHR1 |
| DIO3OS | hsa-miR-185-5p | ATP1B2 |
| DIO3OS | hsa-miR-185-5p | HAPLN2 |
| DIO3OS | hsa-miR-185-5p | CASQ2 |
| DIO3OS | hsa-miR-185-5p | ASPA |
| DIO3OS | hsa-miR-185-5p | PDE8B |
| DIO3OS | hsa-miR-185-5p | OSR1 |
| DIO3OS | hsa-miR-185-5p | SLC2A12 |
| DIO3OS | hsa-miR-185-5p | UNC5D |
| DIO3OS | hsa-miR-185-5p | TCF23 |
| DIO3OS | hsa-miR-185-5p | MASP1 |
| DIO3OS | hsa-miR-185-5p | EMCN |
| DIO3OS | hsa-miR-185-5p | TMEM132B |
| DIO3OS | hsa-miR-185-5p | PRRT2 |
| DIO3OS | hsa-miR-185-5p | VAT1L |
| DIO3OS | hsa-miR-185-5p | KCNK3 |
| DIO3OS | hsa-miR-185-5p | SLC38A11 |
| DIO3OS | hsa-miR-185-5p | GPR37 |
| DIO3OS | hsa-miR-185-5p | CPLX1 |
| DIO3OS | hsa-miR-185-5p | GPR22 |
| DIO3OS | hsa-miR-185-5p | TUB |
| DIO3OS | hsa-miR-185-5p | MSRB3 |
| DIO3OS | hsa-miR-185-5p | LPP |
| DIO3OS | hsa-miR-185-5p | EBF1 |
| DIO3OS | hsa-miR-185-5p | CLU |
| DIO3OS | hsa-miR-185-5p | NCAM1 |
| DIO3OS | hsa-miR-185-5p | FIBIN |
| DIO3OS | hsa-miR-185-5p | ALOX12B |
| DIO3OS | hsa-miR-185-5p | TNFAIP8L3 |
| DIO3OS | hsa-miR-185-5p | GRID1 |
| DIO3OS | hsa-miR-185-5p | PCP4 |
| DIO3OS | hsa-miR-185-5p | SYNGR1 |
| DIO3OS | hsa-miR-185-5p | WSCD2 |
| DIO3OS | hsa-miR-185-5p | CADPS2 |
| DIO3OS | hsa-miR-185-5p | C3orf70 |
| DIO3OS | hsa-miR-185-5p | TSPAN18 |
| DIO3OS | hsa-miR-185-5p | SLC24A2 |
| DIO3OS | hsa-miR-185-5p | PRELP |
| DIO3OS | hsa-miR-185-5p | CAMK2A |
| DIO3OS | hsa-miR-185-5p | ATP2B2 |
| DIO3OS | hsa-miR-185-5p | JPH4 |
| DIO3OS | hsa-miR-185-5p | ADAM33 |
| DIO3OS | hsa-miR-185-5p | DCX |
| DIO3OS | hsa-miR-185-5p | PLN |
| DIO3OS | hsa-miR-185-5p | KLHDC1 |
| DIO3OS | hsa-miR-185-5p | NFIX |
| DIO3OS | hsa-miR-185-5p | MAP2 |
| DIO3OS | hsa-miR-185-5p | MYLK |
| DIO3OS | hsa-miR-185-5p | CNR1 |
| DIO3OS | hsa-miR-185-5p | LMOD1 |
| DIO3OS | hsa-miR-185-5p | PBX1 |
| DIO3OS | hsa-miR-185-5p | RBM20 |
| DIO3OS | hsa-miR-185-5p | SYPL2 |
| DIO3OS | hsa-miR-185-5p | CDR1 |
| DIO3OS | hsa-miR-185-5p | PTGFR |
| DIO3OS | hsa-miR-185-5p | KCNB1 |
| DIO3OS | hsa-miR-185-5p | PAK3 |
| DIO3OS | hsa-miR-185-5p | CHRDL1 |
| DIO3OS | hsa-miR-185-5p | WISP2 |
| DIO3OS | hsa-miR-185-5p | FABP3 |
| DIO3OS | hsa-miR-185-5p | TRPM3 |
| DIO3OS | hsa-miR-185-5p | HIF3A |
| DIO3OS | hsa-miR-185-5p | GPR20 |
| DIO3OS | hsa-miR-185-5p | PTCHD1 |
| DIO3OS | hsa-miR-185-5p | BNC2 |
| DIO3OS | hsa-miR-185-5p | SMOC1 |
| DIO3OS | hsa-miR-185-5p | IGF2 |
| DIO3OS | hsa-miR-185-5p | CYS1 |
| DIO3OS | hsa-miR-185-5p | SEMA5A |
| DIO3OS | hsa-miR-185-5p | SLC22A3 |
| DIO3OS | hsa-miR-185-5p | CDON |
| DIO3OS | hsa-miR-185-5p | SPOCK1 |
| DIO3OS | hsa-miR-185-5p | MYL3 |
| DIO3OS | hsa-miR-185-5p | RIC3 |
| DIO3OS | hsa-miR-185-5p | DGKB |
| DIO3OS | hsa-miR-185-5p | LIMS2 |
| DIO3OS | hsa-miR-185-5p | SCN7A |
| DIO3OS | hsa-miR-185-5p | HSPB7 |
| DIO3OS | hsa-miR-185-5p | ADAMTSL5 |
| DIO3OS | hsa-miR-185-5p | MRVI1 |
| DIO3OS | hsa-miR-185-5p | OSR2 |
| DIO3OS | hsa-miR-185-5p | GFRA1 |
| DIO3OS | hsa-miR-185-5p | MRGPRF |
| DIO3OS | hsa-miR-185-5p | SHISA6 |
| DIO3OS | hsa-miR-185-5p | CMYA5 |
| DIO3OS | hsa-miR-185-5p | TCEA3 |
| DIO3OS | hsa-miR-185-5p | MUC15 |
| DIO3OS | hsa-miR-185-5p | FAM107A |
| DIO3OS | hsa-miR-185-5p | KLF8 |
| DIO3OS | hsa-miR-185-5p | TCEAL4 |
| DIO3OS | hsa-miR-185-5p | ST6GALNAC5 |
| DIO3OS | hsa-miR-185-5p | LDB2 |
| DIO3OS | hsa-miR-185-5p | SORCS2 |
| DIO3OS | hsa-miR-185-5p | SLIT3 |
| DIO3OS | hsa-miR-185-5p | TMEM200B |
| DIO3OS | hsa-miR-185-5p | RANBP17 |
| DIO3OS | hsa-miR-185-5p | PIANP |
| DIO3OS | hsa-miR-185-5p | CES4A |
| DIO3OS | hsa-miR-185-5p | FHL5 |
| DIO3OS | hsa-miR-185-5p | TRERF1 |
| DIO3OS | hsa-miR-185-5p | DUOXA1 |
| DIO3OS | hsa-miR-185-5p | CDH13 |
| DIO3OS | hsa-miR-185-5p | RBFOX3 |
| DIO3OS | hsa-miR-185-5p | AOC3 |
| DIO3OS | hsa-miR-185-5p | PPP1R12B |
| AP003355.2 | hsa-miR-708-5p | ADAM11 |
| AP003355.2 | hsa-miR-708-5p | CCDC80 |
| AP003355.2 | hsa-miR-708-5p | HLF |
| AP003355.2 | hsa-miR-708-5p | CASQ2 |
| AP003355.2 | hsa-miR-708-5p | SGCA |
| AP003355.2 | hsa-miR-708-5p | ASPA |
| AP003355.2 | hsa-miR-708-5p | ASXL3 |
| AP003355.2 | hsa-miR-708-5p | SLC2A12 |
| AP003355.2 | hsa-miR-708-5p | KCNE4 |
| AP003355.2 | hsa-miR-708-5p | EMCN |
| AP003355.2 | hsa-miR-708-5p | TMEM132B |
| AP003355.2 | hsa-miR-708-5p | VAT1L |
| AP003355.2 | hsa-miR-708-5p | KCNK3 |
| AP003355.2 | hsa-miR-708-5p | SLC38A11 |
| AP003355.2 | hsa-miR-708-5p | CPLX1 |
| AP003355.2 | hsa-miR-708-5p | GPR22 |
| AP003355.2 | hsa-miR-708-5p | GPR88 |
| AP003355.2 | hsa-miR-708-5p | NCAM1 |
| AP003355.2 | hsa-miR-708-5p | PLAG1 |
| AP003355.2 | hsa-miR-708-5p | FIBIN |
| AP003355.2 | hsa-miR-708-5p | LURAP1L |
| AP003355.2 | hsa-miR-708-5p | KIAA1549L |
| AP003355.2 | hsa-miR-708-5p | CAPN6 |
| AP003355.2 | hsa-miR-708-5p | GDNF |
| AP003355.2 | hsa-miR-708-5p | SYNGR1 |
| AP003355.2 | hsa-miR-708-5p | KCNH2 |
| AP003355.2 | hsa-miR-708-5p | TSPAN18 |
| AP003355.2 | hsa-miR-708-5p | PRELP |
| AP003355.2 | hsa-miR-708-5p | NR3C2 |
| AP003355.2 | hsa-miR-708-5p | RSPO3 |
| AP003355.2 | hsa-miR-708-5p | DCX |
| AP003355.2 | hsa-miR-708-5p | ZNF483 |
| AP003355.2 | hsa-miR-708-5p | DNM3 |
| AP003355.2 | hsa-miR-708-5p | GPR161 |
| AP003355.2 | hsa-miR-708-5p | ITGA10 |
| AP003355.2 | hsa-miR-708-5p | RBM20 |
| AP003355.2 | hsa-miR-708-5p | LDOC1 |
| AP003355.2 | hsa-miR-708-5p | PTGER3 |
| AP003355.2 | hsa-miR-708-5p | APCDD1L |
| AP003355.2 | hsa-miR-708-5p | KCNB1 |
| AP003355.2 | hsa-miR-708-5p | CHRDL1 |
| AP003355.2 | hsa-miR-708-5p | PRKG1 |
| AP003355.2 | hsa-miR-708-5p | TRPM3 |
| AP003355.2 | hsa-miR-708-5p | HIF3A |
| AP003355.2 | hsa-miR-708-5p | CYS1 |
| AP003355.2 | hsa-miR-708-5p | LGI2 |
| AP003355.2 | hsa-miR-708-5p | SEMA5A |
| AP003355.2 | hsa-miR-708-5p | SLC22A3 |
| AP003355.2 | hsa-miR-708-5p | LGI4 |
| AP003355.2 | hsa-miR-708-5p | CDON |
| AP003355.2 | hsa-miR-708-5p | RIC3 |
| AP003355.2 | hsa-miR-708-5p | MEIS2 |
| AP003355.2 | hsa-miR-708-5p | NFASC |
| AP003355.2 | hsa-miR-708-5p | SCN7A |
| AP003355.2 | hsa-miR-708-5p | MRVI1 |
| AP003355.2 | hsa-miR-708-5p | SYNPO2 |
| AP003355.2 | hsa-miR-708-5p | GFRA1 |
| AP003355.2 | hsa-miR-708-5p | MRGPRF |
| AP003355.2 | hsa-miR-708-5p | SHISA6 |
| AP003355.2 | hsa-miR-708-5p | FAM107A |
| AP003355.2 | hsa-miR-708-5p | TCEAL4 |
| AP003355.2 | hsa-miR-708-5p | PCOLCE2 |
| AP003355.2 | hsa-miR-708-5p | AQP1 |
| AP003355.2 | hsa-miR-708-5p | TMEM200B |
| AP003355.2 | hsa-miR-708-5p | TAGLN |
| AP003355.2 | hsa-miR-708-5p | PIANP |
| AP003355.2 | hsa-miR-708-5p | FXYD6 |
| AP003355.2 | hsa-miR-708-5p | LDB3 |
| AP003355.2 | hsa-miR-708-5p | EML5 |
| AP003355.2 | hsa-miR-708-5p | AOC3 |
| AP003355.2 | hsa-miR-708-5p | PPP1R12B |
| ADIRF-AS1 | hsa-miR-339-5p | HOXA13 |
| ADIRF-AS1 | hsa-miR-339-5p | IGFBP5 |
| ADIRF-AS1 | hsa-miR-339-5p | PTGIS |
| ADIRF-AS1 | hsa-miR-339-5p | ATP1B2 |
| ADIRF-AS1 | hsa-miR-339-5p | GATA5 |
| ADIRF-AS1 | hsa-miR-339-5p | ITGA7 |
| ADIRF-AS1 | hsa-miR-339-5p | ABCC9 |
| ADIRF-AS1 | hsa-miR-339-5p | SLC2A12 |
| ADIRF-AS1 | hsa-miR-339-5p | MYL9 |
| ADIRF-AS1 | hsa-miR-339-5p | RAB3C |
| ADIRF-AS1 | hsa-miR-339-5p | FBXO27 |
| ADIRF-AS1 | hsa-miR-339-5p | TCF23 |
| ADIRF-AS1 | hsa-miR-339-5p | VAT1L |
| ADIRF-AS1 | hsa-miR-339-5p | KCNK3 |
| ADIRF-AS1 | hsa-miR-339-5p | SLC38A11 |
| ADIRF-AS1 | hsa-miR-339-5p | CPLX1 |
| ADIRF-AS1 | hsa-miR-339-5p | ADH1B |
| ADIRF-AS1 | hsa-miR-339-5p | TUB |
| ADIRF-AS1 | hsa-miR-339-5p | MSRB3 |
| ADIRF-AS1 | hsa-miR-339-5p | LPP |
| ADIRF-AS1 | hsa-miR-339-5p | BVES |
| ADIRF-AS1 | hsa-miR-339-5p | CLU |
| ADIRF-AS1 | hsa-miR-339-5p | SLC2A4 |
| ADIRF-AS1 | hsa-miR-339-5p | SYNGR1 |
| ADIRF-AS1 | hsa-miR-339-5p | WSCD2 |
| ADIRF-AS1 | hsa-miR-339-5p | C3orf70 |
| ADIRF-AS1 | hsa-miR-339-5p | TSPAN18 |
| ADIRF-AS1 | hsa-miR-339-5p | SLC24A2 |
| ADIRF-AS1 | hsa-miR-339-5p | PRELP |
| ADIRF-AS1 | hsa-miR-339-5p | ATP2B2 |
| ADIRF-AS1 | hsa-miR-339-5p | PLN |
| ADIRF-AS1 | hsa-miR-339-5p | LMOD1 |
| ADIRF-AS1 | hsa-miR-339-5p | GPR161 |
| ADIRF-AS1 | hsa-miR-339-5p | SYPL2 |
| ADIRF-AS1 | hsa-miR-339-5p | CDR1 |
| ADIRF-AS1 | hsa-miR-339-5p | BEND6 |
| ADIRF-AS1 | hsa-miR-339-5p | PTGER3 |
| ADIRF-AS1 | hsa-miR-339-5p | SORBS1 |
| ADIRF-AS1 | hsa-miR-339-5p | KCNB1 |
| ADIRF-AS1 | hsa-miR-339-5p | PAK3 |
| ADIRF-AS1 | hsa-miR-339-5p | CHRDL1 |
| ADIRF-AS1 | hsa-miR-339-5p | IRS4 |
| ADIRF-AS1 | hsa-miR-339-5p | RAB40A |
| ADIRF-AS1 | hsa-miR-339-5p | WISP2 |
| ADIRF-AS1 | hsa-miR-339-5p | RSPO1 |
| ADIRF-AS1 | hsa-miR-339-5p | GPR20 |
| ADIRF-AS1 | hsa-miR-339-5p | TPM2 |
| ADIRF-AS1 | hsa-miR-339-5p | PTCHD1 |
| ADIRF-AS1 | hsa-miR-339-5p | DCLK1 |
| ADIRF-AS1 | hsa-miR-339-5p | BNC2 |
| ADIRF-AS1 | hsa-miR-339-5p | SMOC1 |
| ADIRF-AS1 | hsa-miR-339-5p | SEMA5A |
| ADIRF-AS1 | hsa-miR-339-5p | LGI4 |
| ADIRF-AS1 | hsa-miR-339-5p | CDON |
| ADIRF-AS1 | hsa-miR-339-5p | PRIMA1 |
| ADIRF-AS1 | hsa-miR-339-5p | FHL1 |
| ADIRF-AS1 | hsa-miR-339-5p | MAPK10 |
| ADIRF-AS1 | hsa-miR-339-5p | CDKL1 |
| ADIRF-AS1 | hsa-miR-339-5p | ST8SIA1 |
| ADIRF-AS1 | hsa-miR-339-5p | NFASC |
| ADIRF-AS1 | hsa-miR-339-5p | ITGA11 |
| ADIRF-AS1 | hsa-miR-339-5p | HMCN2 |
| ADIRF-AS1 | hsa-miR-339-5p | SYNPO2 |
| ADIRF-AS1 | hsa-miR-339-5p | GFRA1 |
| ADIRF-AS1 | hsa-miR-339-5p | MRGPRF |
| ADIRF-AS1 | hsa-miR-339-5p | SHISA6 |
| ADIRF-AS1 | hsa-miR-339-5p | TCEA3 |
| ADIRF-AS1 | hsa-miR-339-5p | KLF8 |
| ADIRF-AS1 | hsa-miR-339-5p | SLC15A2 |
| ADIRF-AS1 | hsa-miR-339-5p | EPHA3 |
| ADIRF-AS1 | hsa-miR-339-5p | SORCS2 |
| ADIRF-AS1 | hsa-miR-339-5p | KCNN2 |
| ADIRF-AS1 | hsa-miR-339-5p | EML5 |
| ADIRF-AS1 | hsa-miR-339-5p | CCDC68 |
| ADIRF-AS1 | hsa-miR-339-5p | PPP1R12B |
| MIR1-1HG-AS1 | hsa-miR-339-5p | HOXA13 |
| MIR1-1HG-AS1 | hsa-miR-339-5p | IGFBP5 |
| MIR1-1HG-AS1 | hsa-miR-339-5p | PTGIS |
| MIR1-1HG-AS1 | hsa-miR-339-5p | ATP1B2 |
| MIR1-1HG-AS1 | hsa-miR-339-5p | GATA5 |
| MIR1-1HG-AS1 | hsa-miR-339-5p | ITGA7 |
| MIR1-1HG-AS1 | hsa-miR-339-5p | ABCC9 |
| MIR1-1HG-AS1 | hsa-miR-339-5p | SLC2A12 |
| MIR1-1HG-AS1 | hsa-miR-339-5p | MYL9 |
| MIR1-1HG-AS1 | hsa-miR-339-5p | RAB3C |
| MIR1-1HG-AS1 | hsa-miR-339-5p | FBXO27 |
| MIR1-1HG-AS1 | hsa-miR-339-5p | TCF23 |
| MIR1-1HG-AS1 | hsa-miR-339-5p | VAT1L |
| MIR1-1HG-AS1 | hsa-miR-339-5p | KCNK3 |
| MIR1-1HG-AS1 | hsa-miR-339-5p | SLC38A11 |
| MIR1-1HG-AS1 | hsa-miR-339-5p | CPLX1 |
| MIR1-1HG-AS1 | hsa-miR-339-5p | ADH1B |
| MIR1-1HG-AS1 | hsa-miR-339-5p | TUB |
| MIR1-1HG-AS1 | hsa-miR-339-5p | MSRB3 |
| MIR1-1HG-AS1 | hsa-miR-339-5p | LPP |
| MIR1-1HG-AS1 | hsa-miR-339-5p | BVES |
| MIR1-1HG-AS1 | hsa-miR-339-5p | CLU |
| MIR1-1HG-AS1 | hsa-miR-339-5p | SLC2A4 |
| MIR1-1HG-AS1 | hsa-miR-339-5p | SYNGR1 |
| MIR1-1HG-AS1 | hsa-miR-339-5p | WSCD2 |
| MIR1-1HG-AS1 | hsa-miR-339-5p | C3orf70 |
| MIR1-1HG-AS1 | hsa-miR-339-5p | TSPAN18 |
| MIR1-1HG-AS1 | hsa-miR-339-5p | SLC24A2 |
| MIR1-1HG-AS1 | hsa-miR-339-5p | PRELP |
| MIR1-1HG-AS1 | hsa-miR-339-5p | ATP2B2 |
| MIR1-1HG-AS1 | hsa-miR-339-5p | PLN |
| MIR1-1HG-AS1 | hsa-miR-339-5p | LMOD1 |
| MIR1-1HG-AS1 | hsa-miR-339-5p | GPR161 |
| MIR1-1HG-AS1 | hsa-miR-339-5p | SYPL2 |
| MIR1-1HG-AS1 | hsa-miR-339-5p | CDR1 |
| MIR1-1HG-AS1 | hsa-miR-339-5p | BEND6 |
| MIR1-1HG-AS1 | hsa-miR-339-5p | PTGER3 |
| MIR1-1HG-AS1 | hsa-miR-339-5p | SORBS1 |
| MIR1-1HG-AS1 | hsa-miR-339-5p | KCNB1 |
| MIR1-1HG-AS1 | hsa-miR-339-5p | PAK3 |
| MIR1-1HG-AS1 | hsa-miR-339-5p | CHRDL1 |
| MIR1-1HG-AS1 | hsa-miR-339-5p | IRS4 |
| MIR1-1HG-AS1 | hsa-miR-339-5p | RAB40A |
| MIR1-1HG-AS1 | hsa-miR-339-5p | WISP2 |
| MIR1-1HG-AS1 | hsa-miR-339-5p | RSPO1 |
| MIR1-1HG-AS1 | hsa-miR-339-5p | GPR20 |
| MIR1-1HG-AS1 | hsa-miR-339-5p | TPM2 |
| MIR1-1HG-AS1 | hsa-miR-339-5p | PTCHD1 |
| MIR1-1HG-AS1 | hsa-miR-339-5p | DCLK1 |
| MIR1-1HG-AS1 | hsa-miR-339-5p | BNC2 |
| MIR1-1HG-AS1 | hsa-miR-339-5p | SMOC1 |
| MIR1-1HG-AS1 | hsa-miR-339-5p | SEMA5A |
| MIR1-1HG-AS1 | hsa-miR-339-5p | LGI4 |
| MIR1-1HG-AS1 | hsa-miR-339-5p | CDON |
| MIR1-1HG-AS1 | hsa-miR-339-5p | PRIMA1 |
| MIR1-1HG-AS1 | hsa-miR-339-5p | FHL1 |
| MIR1-1HG-AS1 | hsa-miR-339-5p | MAPK10 |
| MIR1-1HG-AS1 | hsa-miR-339-5p | CDKL1 |
| MIR1-1HG-AS1 | hsa-miR-339-5p | ST8SIA1 |
| MIR1-1HG-AS1 | hsa-miR-339-5p | NFASC |
| MIR1-1HG-AS1 | hsa-miR-339-5p | ITGA11 |
| MIR1-1HG-AS1 | hsa-miR-339-5p | HMCN2 |
| MIR1-1HG-AS1 | hsa-miR-339-5p | SYNPO2 |
| MIR1-1HG-AS1 | hsa-miR-339-5p | GFRA1 |
| MIR1-1HG-AS1 | hsa-miR-339-5p | MRGPRF |
| MIR1-1HG-AS1 | hsa-miR-339-5p | SHISA6 |
| MIR1-1HG-AS1 | hsa-miR-339-5p | TCEA3 |
| MIR1-1HG-AS1 | hsa-miR-339-5p | KLF8 |
| MIR1-1HG-AS1 | hsa-miR-339-5p | SLC15A2 |
| MIR1-1HG-AS1 | hsa-miR-339-5p | EPHA3 |
| MIR1-1HG-AS1 | hsa-miR-339-5p | SORCS2 |
| MIR1-1HG-AS1 | hsa-miR-339-5p | KCNN2 |
| MIR1-1HG-AS1 | hsa-miR-339-5p | EML5 |
| MIR1-1HG-AS1 | hsa-miR-339-5p | CCDC68 |
| MIR1-1HG-AS1 | hsa-miR-339-5p | PPP1R12B |
| AC100803.2 | hsa-miR-339-5p | HOXA13 |
| AC100803.2 | hsa-miR-339-5p | IGFBP5 |
| AC100803.2 | hsa-miR-339-5p | PTGIS |
| AC100803.2 | hsa-miR-339-5p | ATP1B2 |
| AC100803.2 | hsa-miR-339-5p | GATA5 |
| AC100803.2 | hsa-miR-339-5p | ITGA7 |
| AC100803.2 | hsa-miR-339-5p | ABCC9 |
| AC100803.2 | hsa-miR-339-5p | SLC2A12 |
| AC100803.2 | hsa-miR-339-5p | MYL9 |
| AC100803.2 | hsa-miR-339-5p | RAB3C |
| AC100803.2 | hsa-miR-339-5p | FBXO27 |
| AC100803.2 | hsa-miR-339-5p | TCF23 |
| AC100803.2 | hsa-miR-339-5p | VAT1L |
| AC100803.2 | hsa-miR-339-5p | KCNK3 |
| AC100803.2 | hsa-miR-339-5p | SLC38A11 |
| AC100803.2 | hsa-miR-339-5p | CPLX1 |
| AC100803.2 | hsa-miR-339-5p | ADH1B |
| AC100803.2 | hsa-miR-339-5p | TUB |
| AC100803.2 | hsa-miR-339-5p | MSRB3 |
| AC100803.2 | hsa-miR-339-5p | LPP |
| AC100803.2 | hsa-miR-339-5p | BVES |
| AC100803.2 | hsa-miR-339-5p | CLU |
| AC100803.2 | hsa-miR-339-5p | SLC2A4 |
| AC100803.2 | hsa-miR-339-5p | SYNGR1 |
| AC100803.2 | hsa-miR-339-5p | WSCD2 |
| AC100803.2 | hsa-miR-339-5p | C3orf70 |
| AC100803.2 | hsa-miR-339-5p | TSPAN18 |
| AC100803.2 | hsa-miR-339-5p | SLC24A2 |
| AC100803.2 | hsa-miR-339-5p | PRELP |
| AC100803.2 | hsa-miR-339-5p | ATP2B2 |
| AC100803.2 | hsa-miR-339-5p | PLN |
| AC100803.2 | hsa-miR-339-5p | LMOD1 |
| AC100803.2 | hsa-miR-339-5p | GPR161 |
| AC100803.2 | hsa-miR-339-5p | SYPL2 |
| AC100803.2 | hsa-miR-339-5p | CDR1 |
| AC100803.2 | hsa-miR-339-5p | BEND6 |
| AC100803.2 | hsa-miR-339-5p | PTGER3 |
| AC100803.2 | hsa-miR-339-5p | SORBS1 |
| AC100803.2 | hsa-miR-339-5p | KCNB1 |
| AC100803.2 | hsa-miR-339-5p | PAK3 |
| AC100803.2 | hsa-miR-339-5p | CHRDL1 |
| AC100803.2 | hsa-miR-339-5p | IRS4 |
| AC100803.2 | hsa-miR-339-5p | RAB40A |
| AC100803.2 | hsa-miR-339-5p | WISP2 |
| AC100803.2 | hsa-miR-339-5p | RSPO1 |
| AC100803.2 | hsa-miR-339-5p | GPR20 |
| AC100803.2 | hsa-miR-339-5p | TPM2 |
| AC100803.2 | hsa-miR-339-5p | PTCHD1 |
| AC100803.2 | hsa-miR-339-5p | DCLK1 |
| AC100803.2 | hsa-miR-339-5p | BNC2 |
| AC100803.2 | hsa-miR-339-5p | SMOC1 |
| AC100803.2 | hsa-miR-339-5p | SEMA5A |
| AC100803.2 | hsa-miR-339-5p | LGI4 |
| AC100803.2 | hsa-miR-339-5p | CDON |
| AC100803.2 | hsa-miR-339-5p | PRIMA1 |
| AC100803.2 | hsa-miR-339-5p | FHL1 |
| AC100803.2 | hsa-miR-339-5p | MAPK10 |
| AC100803.2 | hsa-miR-339-5p | CDKL1 |
| AC100803.2 | hsa-miR-339-5p | ST8SIA1 |
| AC100803.2 | hsa-miR-339-5p | NFASC |
| AC100803.2 | hsa-miR-339-5p | ITGA11 |
| AC100803.2 | hsa-miR-339-5p | HMCN2 |
| AC100803.2 | hsa-miR-339-5p | SYNPO2 |
| AC100803.2 | hsa-miR-339-5p | GFRA1 |
| AC100803.2 | hsa-miR-339-5p | MRGPRF |
| AC100803.2 | hsa-miR-339-5p | SHISA6 |
| AC100803.2 | hsa-miR-339-5p | TCEA3 |
| AC100803.2 | hsa-miR-339-5p | KLF8 |
| AC100803.2 | hsa-miR-339-5p | SLC15A2 |
| AC100803.2 | hsa-miR-339-5p | EPHA3 |
| AC100803.2 | hsa-miR-339-5p | SORCS2 |
| AC100803.2 | hsa-miR-339-5p | KCNN2 |
| AC100803.2 | hsa-miR-339-5p | EML5 |
| AC100803.2 | hsa-miR-339-5p | CCDC68 |
| AC100803.2 | hsa-miR-339-5p | PPP1R12B |
| RSU1P3 | hsa-miR-339-5p | HOXA13 |
| RSU1P3 | hsa-miR-339-5p | IGFBP5 |
| RSU1P3 | hsa-miR-339-5p | PTGIS |
| RSU1P3 | hsa-miR-339-5p | ATP1B2 |
| RSU1P3 | hsa-miR-339-5p | GATA5 |
| RSU1P3 | hsa-miR-339-5p | ITGA7 |
| RSU1P3 | hsa-miR-339-5p | ABCC9 |
| RSU1P3 | hsa-miR-339-5p | SLC2A12 |
| RSU1P3 | hsa-miR-339-5p | MYL9 |
| RSU1P3 | hsa-miR-339-5p | RAB3C |
| RSU1P3 | hsa-miR-339-5p | FBXO27 |
| RSU1P3 | hsa-miR-339-5p | TCF23 |
| RSU1P3 | hsa-miR-339-5p | VAT1L |
| RSU1P3 | hsa-miR-339-5p | KCNK3 |
| RSU1P3 | hsa-miR-339-5p | SLC38A11 |
| RSU1P3 | hsa-miR-339-5p | CPLX1 |
| RSU1P3 | hsa-miR-339-5p | ADH1B |
| RSU1P3 | hsa-miR-339-5p | TUB |
| RSU1P3 | hsa-miR-339-5p | MSRB3 |
| RSU1P3 | hsa-miR-339-5p | LPP |
| RSU1P3 | hsa-miR-339-5p | BVES |
| RSU1P3 | hsa-miR-339-5p | CLU |
| RSU1P3 | hsa-miR-339-5p | SLC2A4 |
| RSU1P3 | hsa-miR-339-5p | SYNGR1 |
| RSU1P3 | hsa-miR-339-5p | WSCD2 |
| RSU1P3 | hsa-miR-339-5p | C3orf70 |
| RSU1P3 | hsa-miR-339-5p | TSPAN18 |
| RSU1P3 | hsa-miR-339-5p | SLC24A2 |
| RSU1P3 | hsa-miR-339-5p | PRELP |
| RSU1P3 | hsa-miR-339-5p | ATP2B2 |
| RSU1P3 | hsa-miR-339-5p | PLN |
| RSU1P3 | hsa-miR-339-5p | LMOD1 |
| RSU1P3 | hsa-miR-339-5p | GPR161 |
| RSU1P3 | hsa-miR-339-5p | SYPL2 |
| RSU1P3 | hsa-miR-339-5p | CDR1 |
| RSU1P3 | hsa-miR-339-5p | BEND6 |
| RSU1P3 | hsa-miR-339-5p | PTGER3 |
| RSU1P3 | hsa-miR-339-5p | SORBS1 |
| RSU1P3 | hsa-miR-339-5p | KCNB1 |
| RSU1P3 | hsa-miR-339-5p | PAK3 |
| RSU1P3 | hsa-miR-339-5p | CHRDL1 |
| RSU1P3 | hsa-miR-339-5p | IRS4 |
| RSU1P3 | hsa-miR-339-5p | RAB40A |
| RSU1P3 | hsa-miR-339-5p | WISP2 |
| RSU1P3 | hsa-miR-339-5p | RSPO1 |
| RSU1P3 | hsa-miR-339-5p | GPR20 |
| RSU1P3 | hsa-miR-339-5p | TPM2 |
| RSU1P3 | hsa-miR-339-5p | PTCHD1 |
| RSU1P3 | hsa-miR-339-5p | DCLK1 |
| RSU1P3 | hsa-miR-339-5p | BNC2 |
| RSU1P3 | hsa-miR-339-5p | SMOC1 |
| RSU1P3 | hsa-miR-339-5p | SEMA5A |
| RSU1P3 | hsa-miR-339-5p | LGI4 |
| RSU1P3 | hsa-miR-339-5p | CDON |
| RSU1P3 | hsa-miR-339-5p | PRIMA1 |
| RSU1P3 | hsa-miR-339-5p | FHL1 |
| RSU1P3 | hsa-miR-339-5p | MAPK10 |
| RSU1P3 | hsa-miR-339-5p | CDKL1 |
| RSU1P3 | hsa-miR-339-5p | ST8SIA1 |
| RSU1P3 | hsa-miR-339-5p | NFASC |
| RSU1P3 | hsa-miR-339-5p | ITGA11 |
| RSU1P3 | hsa-miR-339-5p | HMCN2 |
| RSU1P3 | hsa-miR-339-5p | SYNPO2 |
| RSU1P3 | hsa-miR-339-5p | GFRA1 |
| RSU1P3 | hsa-miR-339-5p | MRGPRF |
| RSU1P3 | hsa-miR-339-5p | SHISA6 |
| RSU1P3 | hsa-miR-339-5p | TCEA3 |
| RSU1P3 | hsa-miR-339-5p | KLF8 |
| RSU1P3 | hsa-miR-339-5p | SLC15A2 |
| RSU1P3 | hsa-miR-339-5p | EPHA3 |
| RSU1P3 | hsa-miR-339-5p | SORCS2 |
| RSU1P3 | hsa-miR-339-5p | KCNN2 |
| RSU1P3 | hsa-miR-339-5p | EML5 |
| RSU1P3 | hsa-miR-339-5p | CCDC68 |
| RSU1P3 | hsa-miR-339-5p | PPP1R12B |
| AC068733.3 | hsa-miR-339-5p | HOXA13 |
| AC068733.3 | hsa-miR-339-5p | IGFBP5 |
| AC068733.3 | hsa-miR-339-5p | PTGIS |
| AC068733.3 | hsa-miR-339-5p | ATP1B2 |
| AC068733.3 | hsa-miR-339-5p | GATA5 |
| AC068733.3 | hsa-miR-339-5p | ITGA7 |
| AC068733.3 | hsa-miR-339-5p | ABCC9 |
| AC068733.3 | hsa-miR-339-5p | SLC2A12 |
| AC068733.3 | hsa-miR-339-5p | MYL9 |
| AC068733.3 | hsa-miR-339-5p | RAB3C |
| AC068733.3 | hsa-miR-339-5p | FBXO27 |
| AC068733.3 | hsa-miR-339-5p | TCF23 |
| AC068733.3 | hsa-miR-339-5p | VAT1L |
| AC068733.3 | hsa-miR-339-5p | KCNK3 |
| AC068733.3 | hsa-miR-339-5p | SLC38A11 |
| AC068733.3 | hsa-miR-339-5p | CPLX1 |
| AC068733.3 | hsa-miR-339-5p | ADH1B |
| AC068733.3 | hsa-miR-339-5p | TUB |
| AC068733.3 | hsa-miR-339-5p | MSRB3 |
| AC068733.3 | hsa-miR-339-5p | LPP |
| AC068733.3 | hsa-miR-339-5p | BVES |
| AC068733.3 | hsa-miR-339-5p | CLU |
| AC068733.3 | hsa-miR-339-5p | SLC2A4 |
| AC068733.3 | hsa-miR-339-5p | SYNGR1 |
| AC068733.3 | hsa-miR-339-5p | WSCD2 |
| AC068733.3 | hsa-miR-339-5p | C3orf70 |
| AC068733.3 | hsa-miR-339-5p | TSPAN18 |
| AC068733.3 | hsa-miR-339-5p | SLC24A2 |
| AC068733.3 | hsa-miR-339-5p | PRELP |
| AC068733.3 | hsa-miR-339-5p | ATP2B2 |
| AC068733.3 | hsa-miR-339-5p | PLN |
| AC068733.3 | hsa-miR-339-5p | LMOD1 |
| AC068733.3 | hsa-miR-339-5p | GPR161 |
| AC068733.3 | hsa-miR-339-5p | SYPL2 |
| AC068733.3 | hsa-miR-339-5p | CDR1 |
| AC068733.3 | hsa-miR-339-5p | BEND6 |
| AC068733.3 | hsa-miR-339-5p | PTGER3 |
| AC068733.3 | hsa-miR-339-5p | SORBS1 |
| AC068733.3 | hsa-miR-339-5p | KCNB1 |
| AC068733.3 | hsa-miR-339-5p | PAK3 |
| AC068733.3 | hsa-miR-339-5p | CHRDL1 |
| AC068733.3 | hsa-miR-339-5p | IRS4 |
| AC068733.3 | hsa-miR-339-5p | RAB40A |
| AC068733.3 | hsa-miR-339-5p | WISP2 |
| AC068733.3 | hsa-miR-339-5p | RSPO1 |
| AC068733.3 | hsa-miR-339-5p | GPR20 |
| AC068733.3 | hsa-miR-339-5p | TPM2 |
| AC068733.3 | hsa-miR-339-5p | PTCHD1 |
| AC068733.3 | hsa-miR-339-5p | DCLK1 |
| AC068733.3 | hsa-miR-339-5p | BNC2 |
| AC068733.3 | hsa-miR-339-5p | SMOC1 |
| AC068733.3 | hsa-miR-339-5p | SEMA5A |
| AC068733.3 | hsa-miR-339-5p | LGI4 |
| AC068733.3 | hsa-miR-339-5p | CDON |
| AC068733.3 | hsa-miR-339-5p | PRIMA1 |
| AC068733.3 | hsa-miR-339-5p | FHL1 |
| AC068733.3 | hsa-miR-339-5p | MAPK10 |
| AC068733.3 | hsa-miR-339-5p | CDKL1 |
| AC068733.3 | hsa-miR-339-5p | ST8SIA1 |
| AC068733.3 | hsa-miR-339-5p | NFASC |
| AC068733.3 | hsa-miR-339-5p | ITGA11 |
| AC068733.3 | hsa-miR-339-5p | HMCN2 |
| AC068733.3 | hsa-miR-339-5p | SYNPO2 |
| AC068733.3 | hsa-miR-339-5p | GFRA1 |
| AC068733.3 | hsa-miR-339-5p | MRGPRF |
| AC068733.3 | hsa-miR-339-5p | SHISA6 |
| AC068733.3 | hsa-miR-339-5p | TCEA3 |
| AC068733.3 | hsa-miR-339-5p | KLF8 |
| AC068733.3 | hsa-miR-339-5p | SLC15A2 |
| AC068733.3 | hsa-miR-339-5p | EPHA3 |
| AC068733.3 | hsa-miR-339-5p | SORCS2 |
| AC068733.3 | hsa-miR-339-5p | KCNN2 |
| AC068733.3 | hsa-miR-339-5p | EML5 |
| AC068733.3 | hsa-miR-339-5p | CCDC68 |
| AC068733.3 | hsa-miR-339-5p | PPP1R12B |
| LINC01482 | hsa-miR-339-5p | HOXA13 |
| LINC01482 | hsa-miR-339-5p | IGFBP5 |
| LINC01482 | hsa-miR-339-5p | PTGIS |
| LINC01482 | hsa-miR-339-5p | ATP1B2 |
| LINC01482 | hsa-miR-339-5p | GATA5 |
| LINC01482 | hsa-miR-339-5p | ITGA7 |
| LINC01482 | hsa-miR-339-5p | ABCC9 |
| LINC01482 | hsa-miR-339-5p | SLC2A12 |
| LINC01482 | hsa-miR-339-5p | MYL9 |
| LINC01482 | hsa-miR-339-5p | RAB3C |
| LINC01482 | hsa-miR-339-5p | FBXO27 |
| LINC01482 | hsa-miR-339-5p | TCF23 |
| LINC01482 | hsa-miR-339-5p | VAT1L |
| LINC01482 | hsa-miR-339-5p | KCNK3 |
| LINC01482 | hsa-miR-339-5p | SLC38A11 |
| LINC01482 | hsa-miR-339-5p | CPLX1 |
| LINC01482 | hsa-miR-339-5p | ADH1B |
| LINC01482 | hsa-miR-339-5p | TUB |
| LINC01482 | hsa-miR-339-5p | MSRB3 |
| LINC01482 | hsa-miR-339-5p | LPP |
| LINC01482 | hsa-miR-339-5p | BVES |
| LINC01482 | hsa-miR-339-5p | CLU |
| LINC01482 | hsa-miR-339-5p | SLC2A4 |
| LINC01482 | hsa-miR-339-5p | SYNGR1 |
| LINC01482 | hsa-miR-339-5p | WSCD2 |
| LINC01482 | hsa-miR-339-5p | C3orf70 |
| LINC01482 | hsa-miR-339-5p | TSPAN18 |
| LINC01482 | hsa-miR-339-5p | SLC24A2 |
| LINC01482 | hsa-miR-339-5p | PRELP |
| LINC01482 | hsa-miR-339-5p | ATP2B2 |
| LINC01482 | hsa-miR-339-5p | PLN |
| LINC01482 | hsa-miR-339-5p | LMOD1 |
| LINC01482 | hsa-miR-339-5p | GPR161 |
| LINC01482 | hsa-miR-339-5p | SYPL2 |
| LINC01482 | hsa-miR-339-5p | CDR1 |
| LINC01482 | hsa-miR-339-5p | BEND6 |
| LINC01482 | hsa-miR-339-5p | PTGER3 |
| LINC01482 | hsa-miR-339-5p | SORBS1 |
| LINC01482 | hsa-miR-339-5p | KCNB1 |
| LINC01482 | hsa-miR-339-5p | PAK3 |
| LINC01482 | hsa-miR-339-5p | CHRDL1 |
| LINC01482 | hsa-miR-339-5p | IRS4 |
| LINC01482 | hsa-miR-339-5p | RAB40A |
| LINC01482 | hsa-miR-339-5p | WISP2 |
| LINC01482 | hsa-miR-339-5p | RSPO1 |
| LINC01482 | hsa-miR-339-5p | GPR20 |
| LINC01482 | hsa-miR-339-5p | TPM2 |
| LINC01482 | hsa-miR-339-5p | PTCHD1 |
| LINC01482 | hsa-miR-339-5p | DCLK1 |
| LINC01482 | hsa-miR-339-5p | BNC2 |
| LINC01482 | hsa-miR-339-5p | SMOC1 |
| LINC01482 | hsa-miR-339-5p | SEMA5A |
| LINC01482 | hsa-miR-339-5p | LGI4 |
| LINC01482 | hsa-miR-339-5p | CDON |
| LINC01482 | hsa-miR-339-5p | PRIMA1 |
| LINC01482 | hsa-miR-339-5p | FHL1 |
| LINC01482 | hsa-miR-339-5p | MAPK10 |
| LINC01482 | hsa-miR-339-5p | CDKL1 |
| LINC01482 | hsa-miR-339-5p | ST8SIA1 |
| LINC01482 | hsa-miR-339-5p | NFASC |
| LINC01482 | hsa-miR-339-5p | ITGA11 |
| LINC01482 | hsa-miR-339-5p | HMCN2 |
| LINC01482 | hsa-miR-339-5p | SYNPO2 |
| LINC01482 | hsa-miR-339-5p | GFRA1 |
| LINC01482 | hsa-miR-339-5p | MRGPRF |
| LINC01482 | hsa-miR-339-5p | SHISA6 |
| LINC01482 | hsa-miR-339-5p | TCEA3 |
| LINC01482 | hsa-miR-339-5p | KLF8 |
| LINC01482 | hsa-miR-339-5p | SLC15A2 |
| LINC01482 | hsa-miR-339-5p | EPHA3 |
| LINC01482 | hsa-miR-339-5p | SORCS2 |
| LINC01482 | hsa-miR-339-5p | KCNN2 |
| LINC01482 | hsa-miR-339-5p | EML5 |
| LINC01482 | hsa-miR-339-5p | CCDC68 |
| LINC01482 | hsa-miR-339-5p | PPP1R12B |
| AC098679.2 | hsa-miR-339-5p | HOXA13 |
| AC098679.2 | hsa-miR-339-5p | IGFBP5 |
| AC098679.2 | hsa-miR-339-5p | PTGIS |
| AC098679.2 | hsa-miR-339-5p | ATP1B2 |
| AC098679.2 | hsa-miR-339-5p | GATA5 |
| AC098679.2 | hsa-miR-339-5p | ITGA7 |
| AC098679.2 | hsa-miR-339-5p | ABCC9 |
| AC098679.2 | hsa-miR-339-5p | SLC2A12 |
| AC098679.2 | hsa-miR-339-5p | MYL9 |
| AC098679.2 | hsa-miR-339-5p | RAB3C |
| AC098679.2 | hsa-miR-339-5p | FBXO27 |
| AC098679.2 | hsa-miR-339-5p | TCF23 |
| AC098679.2 | hsa-miR-339-5p | VAT1L |
| AC098679.2 | hsa-miR-339-5p | KCNK3 |
| AC098679.2 | hsa-miR-339-5p | SLC38A11 |
| AC098679.2 | hsa-miR-339-5p | CPLX1 |
| AC098679.2 | hsa-miR-339-5p | ADH1B |
| AC098679.2 | hsa-miR-339-5p | TUB |
| AC098679.2 | hsa-miR-339-5p | MSRB3 |
| AC098679.2 | hsa-miR-339-5p | LPP |
| AC098679.2 | hsa-miR-339-5p | BVES |
| AC098679.2 | hsa-miR-339-5p | CLU |
| AC098679.2 | hsa-miR-339-5p | SLC2A4 |
| AC098679.2 | hsa-miR-339-5p | SYNGR1 |
| AC098679.2 | hsa-miR-339-5p | WSCD2 |
| AC098679.2 | hsa-miR-339-5p | C3orf70 |
| AC098679.2 | hsa-miR-339-5p | TSPAN18 |
| AC098679.2 | hsa-miR-339-5p | SLC24A2 |
| AC098679.2 | hsa-miR-339-5p | PRELP |
| AC098679.2 | hsa-miR-339-5p | ATP2B2 |
| AC098679.2 | hsa-miR-339-5p | PLN |
| AC098679.2 | hsa-miR-339-5p | LMOD1 |
| AC098679.2 | hsa-miR-339-5p | GPR161 |
| AC098679.2 | hsa-miR-339-5p | SYPL2 |
| AC098679.2 | hsa-miR-339-5p | CDR1 |
| AC098679.2 | hsa-miR-339-5p | BEND6 |
| AC098679.2 | hsa-miR-339-5p | PTGER3 |
| AC098679.2 | hsa-miR-339-5p | SORBS1 |
| AC098679.2 | hsa-miR-339-5p | KCNB1 |
| AC098679.2 | hsa-miR-339-5p | PAK3 |
| AC098679.2 | hsa-miR-339-5p | CHRDL1 |
| AC098679.2 | hsa-miR-339-5p | IRS4 |
| AC098679.2 | hsa-miR-339-5p | RAB40A |
| AC098679.2 | hsa-miR-339-5p | WISP2 |
| AC098679.2 | hsa-miR-339-5p | RSPO1 |
| AC098679.2 | hsa-miR-339-5p | GPR20 |
| AC098679.2 | hsa-miR-339-5p | TPM2 |
| AC098679.2 | hsa-miR-339-5p | PTCHD1 |
| AC098679.2 | hsa-miR-339-5p | DCLK1 |
| AC098679.2 | hsa-miR-339-5p | BNC2 |
| AC098679.2 | hsa-miR-339-5p | SMOC1 |
| AC098679.2 | hsa-miR-339-5p | SEMA5A |
| AC098679.2 | hsa-miR-339-5p | LGI4 |
| AC098679.2 | hsa-miR-339-5p | CDON |
| AC098679.2 | hsa-miR-339-5p | PRIMA1 |
| AC098679.2 | hsa-miR-339-5p | FHL1 |
| AC098679.2 | hsa-miR-339-5p | MAPK10 |
| AC098679.2 | hsa-miR-339-5p | CDKL1 |
| AC098679.2 | hsa-miR-339-5p | ST8SIA1 |
| AC098679.2 | hsa-miR-339-5p | NFASC |
| AC098679.2 | hsa-miR-339-5p | ITGA11 |
| AC098679.2 | hsa-miR-339-5p | HMCN2 |
| AC098679.2 | hsa-miR-339-5p | SYNPO2 |
| AC098679.2 | hsa-miR-339-5p | GFRA1 |
| AC098679.2 | hsa-miR-339-5p | MRGPRF |
| AC098679.2 | hsa-miR-339-5p | SHISA6 |
| AC098679.2 | hsa-miR-339-5p | TCEA3 |
| AC098679.2 | hsa-miR-339-5p | KLF8 |
| AC098679.2 | hsa-miR-339-5p | SLC15A2 |
| AC098679.2 | hsa-miR-339-5p | EPHA3 |
| AC098679.2 | hsa-miR-339-5p | SORCS2 |
| AC098679.2 | hsa-miR-339-5p | KCNN2 |
| AC098679.2 | hsa-miR-339-5p | EML5 |
| AC098679.2 | hsa-miR-339-5p | CCDC68 |
| AC098679.2 | hsa-miR-339-5p | PPP1R12B |
| GOLGA2P10 | hsa-miR-339-5p | HOXA13 |
| GOLGA2P10 | hsa-miR-339-5p | IGFBP5 |
| GOLGA2P10 | hsa-miR-339-5p | PTGIS |
| GOLGA2P10 | hsa-miR-339-5p | ATP1B2 |
| GOLGA2P10 | hsa-miR-339-5p | GATA5 |
| GOLGA2P10 | hsa-miR-339-5p | ITGA7 |
| GOLGA2P10 | hsa-miR-339-5p | ABCC9 |
| GOLGA2P10 | hsa-miR-339-5p | SLC2A12 |
| GOLGA2P10 | hsa-miR-339-5p | MYL9 |
| GOLGA2P10 | hsa-miR-339-5p | RAB3C |
| GOLGA2P10 | hsa-miR-339-5p | FBXO27 |
| GOLGA2P10 | hsa-miR-339-5p | TCF23 |
| GOLGA2P10 | hsa-miR-339-5p | VAT1L |
| GOLGA2P10 | hsa-miR-339-5p | KCNK3 |
| GOLGA2P10 | hsa-miR-339-5p | SLC38A11 |
| GOLGA2P10 | hsa-miR-339-5p | CPLX1 |
| GOLGA2P10 | hsa-miR-339-5p | ADH1B |
| GOLGA2P10 | hsa-miR-339-5p | TUB |
| GOLGA2P10 | hsa-miR-339-5p | MSRB3 |
| GOLGA2P10 | hsa-miR-339-5p | LPP |
| GOLGA2P10 | hsa-miR-339-5p | BVES |
| GOLGA2P10 | hsa-miR-339-5p | CLU |
| GOLGA2P10 | hsa-miR-339-5p | SLC2A4 |
| GOLGA2P10 | hsa-miR-339-5p | SYNGR1 |
| GOLGA2P10 | hsa-miR-339-5p | WSCD2 |
| GOLGA2P10 | hsa-miR-339-5p | C3orf70 |
| GOLGA2P10 | hsa-miR-339-5p | TSPAN18 |
| GOLGA2P10 | hsa-miR-339-5p | SLC24A2 |
| GOLGA2P10 | hsa-miR-339-5p | PRELP |
| GOLGA2P10 | hsa-miR-339-5p | ATP2B2 |
| GOLGA2P10 | hsa-miR-339-5p | PLN |
| GOLGA2P10 | hsa-miR-339-5p | LMOD1 |
| GOLGA2P10 | hsa-miR-339-5p | GPR161 |
| GOLGA2P10 | hsa-miR-339-5p | SYPL2 |
| GOLGA2P10 | hsa-miR-339-5p | CDR1 |
| GOLGA2P10 | hsa-miR-339-5p | BEND6 |
| GOLGA2P10 | hsa-miR-339-5p | PTGER3 |
| GOLGA2P10 | hsa-miR-339-5p | SORBS1 |
| GOLGA2P10 | hsa-miR-339-5p | KCNB1 |
| GOLGA2P10 | hsa-miR-339-5p | PAK3 |
| GOLGA2P10 | hsa-miR-339-5p | CHRDL1 |
| GOLGA2P10 | hsa-miR-339-5p | IRS4 |
| GOLGA2P10 | hsa-miR-339-5p | RAB40A |
| GOLGA2P10 | hsa-miR-339-5p | WISP2 |
| GOLGA2P10 | hsa-miR-339-5p | RSPO1 |
| GOLGA2P10 | hsa-miR-339-5p | GPR20 |
| GOLGA2P10 | hsa-miR-339-5p | TPM2 |
| GOLGA2P10 | hsa-miR-339-5p | PTCHD1 |
| GOLGA2P10 | hsa-miR-339-5p | DCLK1 |
| GOLGA2P10 | hsa-miR-339-5p | BNC2 |
| GOLGA2P10 | hsa-miR-339-5p | SMOC1 |
| GOLGA2P10 | hsa-miR-339-5p | SEMA5A |
| GOLGA2P10 | hsa-miR-339-5p | LGI4 |
| GOLGA2P10 | hsa-miR-339-5p | CDON |
| GOLGA2P10 | hsa-miR-339-5p | PRIMA1 |
| GOLGA2P10 | hsa-miR-339-5p | FHL1 |
| GOLGA2P10 | hsa-miR-339-5p | MAPK10 |
| GOLGA2P10 | hsa-miR-339-5p | CDKL1 |
| GOLGA2P10 | hsa-miR-339-5p | ST8SIA1 |
| GOLGA2P10 | hsa-miR-339-5p | NFASC |
| GOLGA2P10 | hsa-miR-339-5p | ITGA11 |
| GOLGA2P10 | hsa-miR-339-5p | HMCN2 |
| GOLGA2P10 | hsa-miR-339-5p | SYNPO2 |
| GOLGA2P10 | hsa-miR-339-5p | GFRA1 |
| GOLGA2P10 | hsa-miR-339-5p | MRGPRF |
| GOLGA2P10 | hsa-miR-339-5p | SHISA6 |
| GOLGA2P10 | hsa-miR-339-5p | TCEA3 |
| GOLGA2P10 | hsa-miR-339-5p | KLF8 |
| GOLGA2P10 | hsa-miR-339-5p | SLC15A2 |
| GOLGA2P10 | hsa-miR-339-5p | EPHA3 |
| GOLGA2P10 | hsa-miR-339-5p | SORCS2 |
| GOLGA2P10 | hsa-miR-339-5p | KCNN2 |
| GOLGA2P10 | hsa-miR-339-5p | EML5 |
| GOLGA2P10 | hsa-miR-339-5p | CCDC68 |
| GOLGA2P10 | hsa-miR-339-5p | PPP1R12B |
| AP001107.8 | hsa-miR-339-5p | HOXA13 |
| AP001107.8 | hsa-miR-339-5p | IGFBP5 |
| AP001107.8 | hsa-miR-339-5p | PTGIS |
| AP001107.8 | hsa-miR-339-5p | ATP1B2 |
| AP001107.8 | hsa-miR-339-5p | GATA5 |
| AP001107.8 | hsa-miR-339-5p | ITGA7 |
| AP001107.8 | hsa-miR-339-5p | ABCC9 |
| AP001107.8 | hsa-miR-339-5p | SLC2A12 |
| AP001107.8 | hsa-miR-339-5p | MYL9 |
| AP001107.8 | hsa-miR-339-5p | RAB3C |
| AP001107.8 | hsa-miR-339-5p | FBXO27 |
| AP001107.8 | hsa-miR-339-5p | TCF23 |
| AP001107.8 | hsa-miR-339-5p | VAT1L |
| AP001107.8 | hsa-miR-339-5p | KCNK3 |
| AP001107.8 | hsa-miR-339-5p | SLC38A11 |
| AP001107.8 | hsa-miR-339-5p | CPLX1 |
| AP001107.8 | hsa-miR-339-5p | ADH1B |
| AP001107.8 | hsa-miR-339-5p | TUB |
| AP001107.8 | hsa-miR-339-5p | MSRB3 |
| AP001107.8 | hsa-miR-339-5p | LPP |
| AP001107.8 | hsa-miR-339-5p | BVES |
| AP001107.8 | hsa-miR-339-5p | CLU |
| AP001107.8 | hsa-miR-339-5p | SLC2A4 |
| AP001107.8 | hsa-miR-339-5p | SYNGR1 |
| AP001107.8 | hsa-miR-339-5p | WSCD2 |
| AP001107.8 | hsa-miR-339-5p | C3orf70 |
| AP001107.8 | hsa-miR-339-5p | TSPAN18 |
| AP001107.8 | hsa-miR-339-5p | SLC24A2 |
| AP001107.8 | hsa-miR-339-5p | PRELP |
| AP001107.8 | hsa-miR-339-5p | ATP2B2 |
| AP001107.8 | hsa-miR-339-5p | PLN |
| AP001107.8 | hsa-miR-339-5p | LMOD1 |
| AP001107.8 | hsa-miR-339-5p | GPR161 |
| AP001107.8 | hsa-miR-339-5p | SYPL2 |
| AP001107.8 | hsa-miR-339-5p | CDR1 |
| AP001107.8 | hsa-miR-339-5p | BEND6 |
| AP001107.8 | hsa-miR-339-5p | PTGER3 |
| AP001107.8 | hsa-miR-339-5p | SORBS1 |
| AP001107.8 | hsa-miR-339-5p | KCNB1 |
| AP001107.8 | hsa-miR-339-5p | PAK3 |
| AP001107.8 | hsa-miR-339-5p | CHRDL1 |
| AP001107.8 | hsa-miR-339-5p | IRS4 |
| AP001107.8 | hsa-miR-339-5p | RAB40A |
| AP001107.8 | hsa-miR-339-5p | WISP2 |
| AP001107.8 | hsa-miR-339-5p | RSPO1 |
| AP001107.8 | hsa-miR-339-5p | GPR20 |
| AP001107.8 | hsa-miR-339-5p | TPM2 |
| AP001107.8 | hsa-miR-339-5p | PTCHD1 |
| AP001107.8 | hsa-miR-339-5p | DCLK1 |
| AP001107.8 | hsa-miR-339-5p | BNC2 |
| AP001107.8 | hsa-miR-339-5p | SMOC1 |
| AP001107.8 | hsa-miR-339-5p | SEMA5A |
| AP001107.8 | hsa-miR-339-5p | LGI4 |
| AP001107.8 | hsa-miR-339-5p | CDON |
| AP001107.8 | hsa-miR-339-5p | PRIMA1 |
| AP001107.8 | hsa-miR-339-5p | FHL1 |
| AP001107.8 | hsa-miR-339-5p | MAPK10 |
| AP001107.8 | hsa-miR-339-5p | CDKL1 |
| AP001107.8 | hsa-miR-339-5p | ST8SIA1 |
| AP001107.8 | hsa-miR-339-5p | NFASC |
| AP001107.8 | hsa-miR-339-5p | ITGA11 |
| AP001107.8 | hsa-miR-339-5p | HMCN2 |
| AP001107.8 | hsa-miR-339-5p | SYNPO2 |
| AP001107.8 | hsa-miR-339-5p | GFRA1 |
| AP001107.8 | hsa-miR-339-5p | MRGPRF |
| AP001107.8 | hsa-miR-339-5p | SHISA6 |
| AP001107.8 | hsa-miR-339-5p | TCEA3 |
| AP001107.8 | hsa-miR-339-5p | KLF8 |
| AP001107.8 | hsa-miR-339-5p | SLC15A2 |
| AP001107.8 | hsa-miR-339-5p | EPHA3 |
| AP001107.8 | hsa-miR-339-5p | SORCS2 |
| AP001107.8 | hsa-miR-339-5p | KCNN2 |
| AP001107.8 | hsa-miR-339-5p | EML5 |
| AP001107.8 | hsa-miR-339-5p | CCDC68 |
| AP001107.8 | hsa-miR-339-5p | PPP1R12B |
| AC024901.1 | hsa-miR-339-5p | HOXA13 |
| AC024901.1 | hsa-miR-339-5p | IGFBP5 |
| AC024901.1 | hsa-miR-339-5p | PTGIS |
| AC024901.1 | hsa-miR-339-5p | ATP1B2 |
| AC024901.1 | hsa-miR-339-5p | GATA5 |
| AC024901.1 | hsa-miR-339-5p | ITGA7 |
| AC024901.1 | hsa-miR-339-5p | ABCC9 |
| AC024901.1 | hsa-miR-339-5p | SLC2A12 |
| AC024901.1 | hsa-miR-339-5p | MYL9 |
| AC024901.1 | hsa-miR-339-5p | RAB3C |
| AC024901.1 | hsa-miR-339-5p | FBXO27 |
| AC024901.1 | hsa-miR-339-5p | TCF23 |
| AC024901.1 | hsa-miR-339-5p | VAT1L |
| AC024901.1 | hsa-miR-339-5p | KCNK3 |
| AC024901.1 | hsa-miR-339-5p | SLC38A11 |
| AC024901.1 | hsa-miR-339-5p | CPLX1 |
| AC024901.1 | hsa-miR-339-5p | ADH1B |
| AC024901.1 | hsa-miR-339-5p | TUB |
| AC024901.1 | hsa-miR-339-5p | MSRB3 |
| AC024901.1 | hsa-miR-339-5p | LPP |
| AC024901.1 | hsa-miR-339-5p | BVES |
| AC024901.1 | hsa-miR-339-5p | CLU |
| AC024901.1 | hsa-miR-339-5p | SLC2A4 |
| AC024901.1 | hsa-miR-339-5p | SYNGR1 |
| AC024901.1 | hsa-miR-339-5p | WSCD2 |
| AC024901.1 | hsa-miR-339-5p | C3orf70 |
| AC024901.1 | hsa-miR-339-5p | TSPAN18 |
| AC024901.1 | hsa-miR-339-5p | SLC24A2 |
| AC024901.1 | hsa-miR-339-5p | PRELP |
| AC024901.1 | hsa-miR-339-5p | ATP2B2 |
| AC024901.1 | hsa-miR-339-5p | PLN |
| AC024901.1 | hsa-miR-339-5p | LMOD1 |
| AC024901.1 | hsa-miR-339-5p | GPR161 |
| AC024901.1 | hsa-miR-339-5p | SYPL2 |
| AC024901.1 | hsa-miR-339-5p | CDR1 |
| AC024901.1 | hsa-miR-339-5p | BEND6 |
| AC024901.1 | hsa-miR-339-5p | PTGER3 |
| AC024901.1 | hsa-miR-339-5p | SORBS1 |
| AC024901.1 | hsa-miR-339-5p | KCNB1 |
| AC024901.1 | hsa-miR-339-5p | PAK3 |
| AC024901.1 | hsa-miR-339-5p | CHRDL1 |
| AC024901.1 | hsa-miR-339-5p | IRS4 |
| AC024901.1 | hsa-miR-339-5p | RAB40A |
| AC024901.1 | hsa-miR-339-5p | WISP2 |
| AC024901.1 | hsa-miR-339-5p | RSPO1 |
| AC024901.1 | hsa-miR-339-5p | GPR20 |
| AC024901.1 | hsa-miR-339-5p | TPM2 |
| AC024901.1 | hsa-miR-339-5p | PTCHD1 |
| AC024901.1 | hsa-miR-339-5p | DCLK1 |
| AC024901.1 | hsa-miR-339-5p | BNC2 |
| AC024901.1 | hsa-miR-339-5p | SMOC1 |
| AC024901.1 | hsa-miR-339-5p | SEMA5A |
| AC024901.1 | hsa-miR-339-5p | LGI4 |
| AC024901.1 | hsa-miR-339-5p | CDON |
| AC024901.1 | hsa-miR-339-5p | PRIMA1 |
| AC024901.1 | hsa-miR-339-5p | FHL1 |
| AC024901.1 | hsa-miR-339-5p | MAPK10 |
| AC024901.1 | hsa-miR-339-5p | CDKL1 |
| AC024901.1 | hsa-miR-339-5p | ST8SIA1 |
| AC024901.1 | hsa-miR-339-5p | NFASC |
| AC024901.1 | hsa-miR-339-5p | ITGA11 |
| AC024901.1 | hsa-miR-339-5p | HMCN2 |
| AC024901.1 | hsa-miR-339-5p | SYNPO2 |
| AC024901.1 | hsa-miR-339-5p | GFRA1 |
| AC024901.1 | hsa-miR-339-5p | MRGPRF |
| AC024901.1 | hsa-miR-339-5p | SHISA6 |
| AC024901.1 | hsa-miR-339-5p | TCEA3 |
| AC024901.1 | hsa-miR-339-5p | KLF8 |
| AC024901.1 | hsa-miR-339-5p | SLC15A2 |
| AC024901.1 | hsa-miR-339-5p | EPHA3 |
| AC024901.1 | hsa-miR-339-5p | SORCS2 |
| AC024901.1 | hsa-miR-339-5p | KCNN2 |
| AC024901.1 | hsa-miR-339-5p | EML5 |
| AC024901.1 | hsa-miR-339-5p | CCDC68 |
| AC024901.1 | hsa-miR-339-5p | PPP1R12B |
| AP003355.2 | hsa-miR-339-5p | HOXA13 |
| AP003355.2 | hsa-miR-339-5p | IGFBP5 |
| AP003355.2 | hsa-miR-339-5p | PTGIS |
| AP003355.2 | hsa-miR-339-5p | ATP1B2 |
| AP003355.2 | hsa-miR-339-5p | GATA5 |
| AP003355.2 | hsa-miR-339-5p | ITGA7 |
| AP003355.2 | hsa-miR-339-5p | ABCC9 |
| AP003355.2 | hsa-miR-339-5p | SLC2A12 |
| AP003355.2 | hsa-miR-339-5p | MYL9 |
| AP003355.2 | hsa-miR-339-5p | RAB3C |
| AP003355.2 | hsa-miR-339-5p | FBXO27 |
| AP003355.2 | hsa-miR-339-5p | TCF23 |
| AP003355.2 | hsa-miR-339-5p | VAT1L |
| AP003355.2 | hsa-miR-339-5p | KCNK3 |
| AP003355.2 | hsa-miR-339-5p | SLC38A11 |
| AP003355.2 | hsa-miR-339-5p | CPLX1 |
| AP003355.2 | hsa-miR-339-5p | ADH1B |
| AP003355.2 | hsa-miR-339-5p | TUB |
| AP003355.2 | hsa-miR-339-5p | MSRB3 |
| AP003355.2 | hsa-miR-339-5p | LPP |
| AP003355.2 | hsa-miR-339-5p | BVES |
| AP003355.2 | hsa-miR-339-5p | CLU |
| AP003355.2 | hsa-miR-339-5p | SLC2A4 |
| AP003355.2 | hsa-miR-339-5p | SYNGR1 |
| AP003355.2 | hsa-miR-339-5p | WSCD2 |
| AP003355.2 | hsa-miR-339-5p | C3orf70 |
| AP003355.2 | hsa-miR-339-5p | TSPAN18 |
| AP003355.2 | hsa-miR-339-5p | SLC24A2 |
| AP003355.2 | hsa-miR-339-5p | PRELP |
| AP003355.2 | hsa-miR-339-5p | ATP2B2 |
| AP003355.2 | hsa-miR-339-5p | PLN |
| AP003355.2 | hsa-miR-339-5p | LMOD1 |
| AP003355.2 | hsa-miR-339-5p | GPR161 |
| AP003355.2 | hsa-miR-339-5p | SYPL2 |
| AP003355.2 | hsa-miR-339-5p | CDR1 |
| AP003355.2 | hsa-miR-339-5p | BEND6 |
| AP003355.2 | hsa-miR-339-5p | PTGER3 |
| AP003355.2 | hsa-miR-339-5p | SORBS1 |
| AP003355.2 | hsa-miR-339-5p | KCNB1 |
| AP003355.2 | hsa-miR-339-5p | PAK3 |
| AP003355.2 | hsa-miR-339-5p | CHRDL1 |
| AP003355.2 | hsa-miR-339-5p | IRS4 |
| AP003355.2 | hsa-miR-339-5p | RAB40A |
| AP003355.2 | hsa-miR-339-5p | WISP2 |
| AP003355.2 | hsa-miR-339-5p | RSPO1 |
| AP003355.2 | hsa-miR-339-5p | GPR20 |
| AP003355.2 | hsa-miR-339-5p | TPM2 |
| AP003355.2 | hsa-miR-339-5p | PTCHD1 |
| AP003355.2 | hsa-miR-339-5p | DCLK1 |
| AP003355.2 | hsa-miR-339-5p | BNC2 |
| AP003355.2 | hsa-miR-339-5p | SMOC1 |
| AP003355.2 | hsa-miR-339-5p | SEMA5A |
| AP003355.2 | hsa-miR-339-5p | LGI4 |
| AP003355.2 | hsa-miR-339-5p | CDON |
| AP003355.2 | hsa-miR-339-5p | PRIMA1 |
| AP003355.2 | hsa-miR-339-5p | FHL1 |
| AP003355.2 | hsa-miR-339-5p | MAPK10 |
| AP003355.2 | hsa-miR-339-5p | CDKL1 |
| AP003355.2 | hsa-miR-339-5p | ST8SIA1 |
| AP003355.2 | hsa-miR-339-5p | NFASC |
| AP003355.2 | hsa-miR-339-5p | ITGA11 |
| AP003355.2 | hsa-miR-339-5p | HMCN2 |
| AP003355.2 | hsa-miR-339-5p | SYNPO2 |
| AP003355.2 | hsa-miR-339-5p | GFRA1 |
| AP003355.2 | hsa-miR-339-5p | MRGPRF |
| AP003355.2 | hsa-miR-339-5p | SHISA6 |
| AP003355.2 | hsa-miR-339-5p | TCEA3 |
| AP003355.2 | hsa-miR-339-5p | KLF8 |
| AP003355.2 | hsa-miR-339-5p | SLC15A2 |
| AP003355.2 | hsa-miR-339-5p | EPHA3 |
| AP003355.2 | hsa-miR-339-5p | SORCS2 |
| AP003355.2 | hsa-miR-339-5p | KCNN2 |
| AP003355.2 | hsa-miR-339-5p | EML5 |
| AP003355.2 | hsa-miR-339-5p | CCDC68 |
| AP003355.2 | hsa-miR-339-5p | PPP1R12B |
| DIO3OS | hsa-miR-339-5p | HOXA13 |
| DIO3OS | hsa-miR-339-5p | IGFBP5 |
| DIO3OS | hsa-miR-339-5p | PTGIS |
| DIO3OS | hsa-miR-339-5p | ATP1B2 |
| DIO3OS | hsa-miR-339-5p | GATA5 |
| DIO3OS | hsa-miR-339-5p | ITGA7 |
| DIO3OS | hsa-miR-339-5p | ABCC9 |
| DIO3OS | hsa-miR-339-5p | SLC2A12 |
| DIO3OS | hsa-miR-339-5p | MYL9 |
| DIO3OS | hsa-miR-339-5p | RAB3C |
| DIO3OS | hsa-miR-339-5p | FBXO27 |
| DIO3OS | hsa-miR-339-5p | TCF23 |
| DIO3OS | hsa-miR-339-5p | VAT1L |
| DIO3OS | hsa-miR-339-5p | KCNK3 |
| DIO3OS | hsa-miR-339-5p | SLC38A11 |
| DIO3OS | hsa-miR-339-5p | CPLX1 |
| DIO3OS | hsa-miR-339-5p | ADH1B |
| DIO3OS | hsa-miR-339-5p | TUB |
| DIO3OS | hsa-miR-339-5p | MSRB3 |
| DIO3OS | hsa-miR-339-5p | LPP |
| DIO3OS | hsa-miR-339-5p | BVES |
| DIO3OS | hsa-miR-339-5p | CLU |
| DIO3OS | hsa-miR-339-5p | SLC2A4 |
| DIO3OS | hsa-miR-339-5p | SYNGR1 |
| DIO3OS | hsa-miR-339-5p | WSCD2 |
| DIO3OS | hsa-miR-339-5p | C3orf70 |
| DIO3OS | hsa-miR-339-5p | TSPAN18 |
| DIO3OS | hsa-miR-339-5p | SLC24A2 |
| DIO3OS | hsa-miR-339-5p | PRELP |
| DIO3OS | hsa-miR-339-5p | ATP2B2 |
| DIO3OS | hsa-miR-339-5p | PLN |
| DIO3OS | hsa-miR-339-5p | LMOD1 |
| DIO3OS | hsa-miR-339-5p | GPR161 |
| DIO3OS | hsa-miR-339-5p | SYPL2 |
| DIO3OS | hsa-miR-339-5p | CDR1 |
| DIO3OS | hsa-miR-339-5p | BEND6 |
| DIO3OS | hsa-miR-339-5p | PTGER3 |
| DIO3OS | hsa-miR-339-5p | SORBS1 |
| DIO3OS | hsa-miR-339-5p | KCNB1 |
| DIO3OS | hsa-miR-339-5p | PAK3 |
| DIO3OS | hsa-miR-339-5p | CHRDL1 |
| DIO3OS | hsa-miR-339-5p | IRS4 |
| DIO3OS | hsa-miR-339-5p | RAB40A |
| DIO3OS | hsa-miR-339-5p | WISP2 |
| DIO3OS | hsa-miR-339-5p | RSPO1 |
| DIO3OS | hsa-miR-339-5p | GPR20 |
| DIO3OS | hsa-miR-339-5p | TPM2 |
| DIO3OS | hsa-miR-339-5p | PTCHD1 |
| DIO3OS | hsa-miR-339-5p | DCLK1 |
| DIO3OS | hsa-miR-339-5p | BNC2 |
| DIO3OS | hsa-miR-339-5p | SMOC1 |
| DIO3OS | hsa-miR-339-5p | SEMA5A |
| DIO3OS | hsa-miR-339-5p | LGI4 |
| DIO3OS | hsa-miR-339-5p | CDON |
| DIO3OS | hsa-miR-339-5p | PRIMA1 |
| DIO3OS | hsa-miR-339-5p | FHL1 |
| DIO3OS | hsa-miR-339-5p | MAPK10 |
| DIO3OS | hsa-miR-339-5p | CDKL1 |
| DIO3OS | hsa-miR-339-5p | ST8SIA1 |
| DIO3OS | hsa-miR-339-5p | NFASC |
| DIO3OS | hsa-miR-339-5p | ITGA11 |
| DIO3OS | hsa-miR-339-5p | HMCN2 |
| DIO3OS | hsa-miR-339-5p | SYNPO2 |
| DIO3OS | hsa-miR-339-5p | GFRA1 |
| DIO3OS | hsa-miR-339-5p | MRGPRF |
| DIO3OS | hsa-miR-339-5p | SHISA6 |
| DIO3OS | hsa-miR-339-5p | TCEA3 |
| DIO3OS | hsa-miR-339-5p | KLF8 |
| DIO3OS | hsa-miR-339-5p | SLC15A2 |
| DIO3OS | hsa-miR-339-5p | EPHA3 |
| DIO3OS | hsa-miR-339-5p | SORCS2 |
| DIO3OS | hsa-miR-339-5p | KCNN2 |
| DIO3OS | hsa-miR-339-5p | EML5 |
| DIO3OS | hsa-miR-339-5p | CCDC68 |
| DIO3OS | hsa-miR-339-5p | PPP1R12B |
| HRAT92 | hsa-miR-339-5p | HOXA13 |
| HRAT92 | hsa-miR-339-5p | IGFBP5 |
| HRAT92 | hsa-miR-339-5p | PTGIS |
| HRAT92 | hsa-miR-339-5p | ATP1B2 |
| HRAT92 | hsa-miR-339-5p | GATA5 |
| HRAT92 | hsa-miR-339-5p | ITGA7 |
| HRAT92 | hsa-miR-339-5p | ABCC9 |
| HRAT92 | hsa-miR-339-5p | SLC2A12 |
| HRAT92 | hsa-miR-339-5p | MYL9 |
| HRAT92 | hsa-miR-339-5p | RAB3C |
| HRAT92 | hsa-miR-339-5p | FBXO27 |
| HRAT92 | hsa-miR-339-5p | TCF23 |
| HRAT92 | hsa-miR-339-5p | VAT1L |
| HRAT92 | hsa-miR-339-5p | KCNK3 |
| HRAT92 | hsa-miR-339-5p | SLC38A11 |
| HRAT92 | hsa-miR-339-5p | CPLX1 |
| HRAT92 | hsa-miR-339-5p | ADH1B |
| HRAT92 | hsa-miR-339-5p | TUB |
| HRAT92 | hsa-miR-339-5p | MSRB3 |
| HRAT92 | hsa-miR-339-5p | LPP |
| HRAT92 | hsa-miR-339-5p | BVES |
| HRAT92 | hsa-miR-339-5p | CLU |
| HRAT92 | hsa-miR-339-5p | SLC2A4 |
| HRAT92 | hsa-miR-339-5p | SYNGR1 |
| HRAT92 | hsa-miR-339-5p | WSCD2 |
| HRAT92 | hsa-miR-339-5p | C3orf70 |
| HRAT92 | hsa-miR-339-5p | TSPAN18 |
| HRAT92 | hsa-miR-339-5p | SLC24A2 |
| HRAT92 | hsa-miR-339-5p | PRELP |
| HRAT92 | hsa-miR-339-5p | ATP2B2 |
| HRAT92 | hsa-miR-339-5p | PLN |
| HRAT92 | hsa-miR-339-5p | LMOD1 |
| HRAT92 | hsa-miR-339-5p | GPR161 |
| HRAT92 | hsa-miR-339-5p | SYPL2 |
| HRAT92 | hsa-miR-339-5p | CDR1 |
| HRAT92 | hsa-miR-339-5p | BEND6 |
| HRAT92 | hsa-miR-339-5p | PTGER3 |
| HRAT92 | hsa-miR-339-5p | SORBS1 |
| HRAT92 | hsa-miR-339-5p | KCNB1 |
| HRAT92 | hsa-miR-339-5p | PAK3 |
| HRAT92 | hsa-miR-339-5p | CHRDL1 |
| HRAT92 | hsa-miR-339-5p | IRS4 |
| HRAT92 | hsa-miR-339-5p | RAB40A |
| HRAT92 | hsa-miR-339-5p | WISP2 |
| HRAT92 | hsa-miR-339-5p | RSPO1 |
| HRAT92 | hsa-miR-339-5p | GPR20 |
| HRAT92 | hsa-miR-339-5p | TPM2 |
| HRAT92 | hsa-miR-339-5p | PTCHD1 |
| HRAT92 | hsa-miR-339-5p | DCLK1 |
| HRAT92 | hsa-miR-339-5p | BNC2 |
| HRAT92 | hsa-miR-339-5p | SMOC1 |
| HRAT92 | hsa-miR-339-5p | SEMA5A |
| HRAT92 | hsa-miR-339-5p | LGI4 |
| HRAT92 | hsa-miR-339-5p | CDON |
| HRAT92 | hsa-miR-339-5p | PRIMA1 |
| HRAT92 | hsa-miR-339-5p | FHL1 |
| HRAT92 | hsa-miR-339-5p | MAPK10 |
| HRAT92 | hsa-miR-339-5p | CDKL1 |
| HRAT92 | hsa-miR-339-5p | ST8SIA1 |
| HRAT92 | hsa-miR-339-5p | NFASC |
| HRAT92 | hsa-miR-339-5p | ITGA11 |
| HRAT92 | hsa-miR-339-5p | HMCN2 |
| HRAT92 | hsa-miR-339-5p | SYNPO2 |
| HRAT92 | hsa-miR-339-5p | GFRA1 |
| HRAT92 | hsa-miR-339-5p | MRGPRF |
| HRAT92 | hsa-miR-339-5p | SHISA6 |
| HRAT92 | hsa-miR-339-5p | TCEA3 |
| HRAT92 | hsa-miR-339-5p | KLF8 |
| HRAT92 | hsa-miR-339-5p | SLC15A2 |
| HRAT92 | hsa-miR-339-5p | EPHA3 |
| HRAT92 | hsa-miR-339-5p | SORCS2 |
| HRAT92 | hsa-miR-339-5p | KCNN2 |
| HRAT92 | hsa-miR-339-5p | EML5 |
| HRAT92 | hsa-miR-339-5p | CCDC68 |
| HRAT92 | hsa-miR-339-5p | PPP1R12B |
| MBNL1-AS1 | hsa-miR-149-5p | INMT |
| MBNL1-AS1 | hsa-miR-149-5p | FSTL3 |
| MBNL1-AS1 | hsa-miR-149-5p | ADAM11 |
| MBNL1-AS1 | hsa-miR-149-5p | CCDC80 |
| MBNL1-AS1 | hsa-miR-149-5p | IGFBP5 |
| MBNL1-AS1 | hsa-miR-149-5p | RAB9B |
| MBNL1-AS1 | hsa-miR-149-5p | PTGIS |
| MBNL1-AS1 | hsa-miR-149-5p | GATA5 |
| MBNL1-AS1 | hsa-miR-149-5p | POPDC3 |
| MBNL1-AS1 | hsa-miR-149-5p | RERG |
| MBNL1-AS1 | hsa-miR-149-5p | PDE8B |
| MBNL1-AS1 | hsa-miR-149-5p | PTH2R |
| MBNL1-AS1 | hsa-miR-149-5p | SLC2A12 |
| MBNL1-AS1 | hsa-miR-149-5p | RAB3C |
| MBNL1-AS1 | hsa-miR-149-5p | UNC5D |
| MBNL1-AS1 | hsa-miR-149-5p | TCF23 |
| MBNL1-AS1 | hsa-miR-149-5p | MASP1 |
| MBNL1-AS1 | hsa-miR-149-5p | TMEM132B |
| MBNL1-AS1 | hsa-miR-149-5p | MSRB3 |
| MBNL1-AS1 | hsa-miR-149-5p | GAL3ST3 |
| MBNL1-AS1 | hsa-miR-149-5p | SPEG |
| MBNL1-AS1 | hsa-miR-149-5p | LPP |
| MBNL1-AS1 | hsa-miR-149-5p | C7 |
| MBNL1-AS1 | hsa-miR-149-5p | GPR88 |
| MBNL1-AS1 | hsa-miR-149-5p | CLU |
| MBNL1-AS1 | hsa-miR-149-5p | PLAG1 |
| MBNL1-AS1 | hsa-miR-149-5p | SLC2A4 |
| MBNL1-AS1 | hsa-miR-149-5p | FIBIN |
| MBNL1-AS1 | hsa-miR-149-5p | SYT9 |
| MBNL1-AS1 | hsa-miR-149-5p | CAPN6 |
| MBNL1-AS1 | hsa-miR-149-5p | C11orf87 |
| MBNL1-AS1 | hsa-miR-149-5p | TNFAIP8L3 |
| MBNL1-AS1 | hsa-miR-149-5p | BTNL9 |
| MBNL1-AS1 | hsa-miR-149-5p | GRID1 |
| MBNL1-AS1 | hsa-miR-149-5p | ST6GALNAC3 |
| MBNL1-AS1 | hsa-miR-149-5p | SYNGR1 |
| MBNL1-AS1 | hsa-miR-149-5p | COL4A6 |
| MBNL1-AS1 | hsa-miR-149-5p | TSPAN18 |
| MBNL1-AS1 | hsa-miR-149-5p | SLC24A2 |
| MBNL1-AS1 | hsa-miR-149-5p | PRELP |
| MBNL1-AS1 | hsa-miR-149-5p | THNSL2 |
| MBNL1-AS1 | hsa-miR-149-5p | ATP2B2 |
| MBNL1-AS1 | hsa-miR-149-5p | MYOM1 |
| MBNL1-AS1 | hsa-miR-149-5p | ADAM33 |
| MBNL1-AS1 | hsa-miR-149-5p | DCX |
| MBNL1-AS1 | hsa-miR-149-5p | PLN |
| MBNL1-AS1 | hsa-miR-149-5p | ZNF483 |
| MBNL1-AS1 | hsa-miR-149-5p | NFIX |
| MBNL1-AS1 | hsa-miR-149-5p | MYLK |
| MBNL1-AS1 | hsa-miR-149-5p | LMOD1 |
| MBNL1-AS1 | hsa-miR-149-5p | DNM3 |
| MBNL1-AS1 | hsa-miR-149-5p | GPR161 |
| MBNL1-AS1 | hsa-miR-149-5p | SORBS1 |
| MBNL1-AS1 | hsa-miR-149-5p | SLC25A27 |
| MBNL1-AS1 | hsa-miR-149-5p | KCNB1 |
| MBNL1-AS1 | hsa-miR-149-5p | PAK3 |
| MBNL1-AS1 | hsa-miR-149-5p | WISP2 |
| MBNL1-AS1 | hsa-miR-149-5p | JPH2 |
| MBNL1-AS1 | hsa-miR-149-5p | RSPO1 |
| MBNL1-AS1 | hsa-miR-149-5p | PRKG1 |
| MBNL1-AS1 | hsa-miR-149-5p | GPR20 |
| MBNL1-AS1 | hsa-miR-149-5p | PKHD1L1 |
| MBNL1-AS1 | hsa-miR-149-5p | PTCHD1 |
| MBNL1-AS1 | hsa-miR-149-5p | DCLK1 |
| MBNL1-AS1 | hsa-miR-149-5p | BNC2 |
| MBNL1-AS1 | hsa-miR-149-5p | IGF2 |
| MBNL1-AS1 | hsa-miR-149-5p | CYS1 |
| MBNL1-AS1 | hsa-miR-149-5p | LGI2 |
| MBNL1-AS1 | hsa-miR-149-5p | SEMA5A |
| MBNL1-AS1 | hsa-miR-149-5p | CDON |
| MBNL1-AS1 | hsa-miR-149-5p | PRIMA1 |
| MBNL1-AS1 | hsa-miR-149-5p | MAPK10 |
| MBNL1-AS1 | hsa-miR-149-5p | KCNG1 |
| MBNL1-AS1 | hsa-miR-149-5p | ZNF208 |
| MBNL1-AS1 | hsa-miR-149-5p | MEIS2 |
| MBNL1-AS1 | hsa-miR-149-5p | NFASC |
| MBNL1-AS1 | hsa-miR-149-5p | DGKB |
| MBNL1-AS1 | hsa-miR-149-5p | SYNC |
| MBNL1-AS1 | hsa-miR-149-5p | LIMS2 |
| MBNL1-AS1 | hsa-miR-149-5p | MRVI1 |
| MBNL1-AS1 | hsa-miR-149-5p | ITGA11 |
| MBNL1-AS1 | hsa-miR-149-5p | GFRA1 |
| MBNL1-AS1 | hsa-miR-149-5p | MRGPRF |
| MBNL1-AS1 | hsa-miR-149-5p | SHISA6 |
| MBNL1-AS1 | hsa-miR-149-5p | TCEA3 |
| MBNL1-AS1 | hsa-miR-149-5p | MYH11 |
| MBNL1-AS1 | hsa-miR-149-5p | MUC15 |
| MBNL1-AS1 | hsa-miR-149-5p | FAM107A |
| MBNL1-AS1 | hsa-miR-149-5p | TCEAL4 |
| MBNL1-AS1 | hsa-miR-149-5p | SLC15A2 |
| MBNL1-AS1 | hsa-miR-149-5p | SORCS2 |
| MBNL1-AS1 | hsa-miR-149-5p | SLIT3 |
| MBNL1-AS1 | hsa-miR-149-5p | ANGPT1 |
| MBNL1-AS1 | hsa-miR-149-5p | NAALAD2 |
| MBNL1-AS1 | hsa-miR-149-5p | PIANP |
| MBNL1-AS1 | hsa-miR-149-5p | PLXNB3 |
| MBNL1-AS1 | hsa-miR-149-5p | FHL5 |
| MBNL1-AS1 | hsa-miR-149-5p | LDB3 |
| MBNL1-AS1 | hsa-miR-149-5p | NDUFA4L2 |
| MBNL1-AS1 | hsa-miR-149-5p | AHNAK2 |
| MBNL1-AS1 | hsa-miR-149-5p | DET1 |
| MBNL1-AS1 | hsa-miR-149-5p | RBFOX3 |
| MBNL1-AS1 | hsa-miR-149-5p | CCDC68 |
| MBNL1-AS1 | hsa-miR-149-5p | AOC3 |
| MBNL1-AS1 | hsa-miR-149-5p | PPP1R12B |
| ADIRF-AS1 | hsa-miR-149-5p | INMT |
| ADIRF-AS1 | hsa-miR-149-5p | FSTL3 |
| ADIRF-AS1 | hsa-miR-149-5p | ADAM11 |
| ADIRF-AS1 | hsa-miR-149-5p | CCDC80 |
| ADIRF-AS1 | hsa-miR-149-5p | IGFBP5 |
| ADIRF-AS1 | hsa-miR-149-5p | RAB9B |
| ADIRF-AS1 | hsa-miR-149-5p | PTGIS |
| ADIRF-AS1 | hsa-miR-149-5p | GATA5 |
| ADIRF-AS1 | hsa-miR-149-5p | POPDC3 |
| ADIRF-AS1 | hsa-miR-149-5p | RERG |
| ADIRF-AS1 | hsa-miR-149-5p | PDE8B |
| ADIRF-AS1 | hsa-miR-149-5p | PTH2R |
| ADIRF-AS1 | hsa-miR-149-5p | SLC2A12 |
| ADIRF-AS1 | hsa-miR-149-5p | RAB3C |
| ADIRF-AS1 | hsa-miR-149-5p | UNC5D |
| ADIRF-AS1 | hsa-miR-149-5p | TCF23 |
| ADIRF-AS1 | hsa-miR-149-5p | MASP1 |
| ADIRF-AS1 | hsa-miR-149-5p | TMEM132B |
| ADIRF-AS1 | hsa-miR-149-5p | MSRB3 |
| ADIRF-AS1 | hsa-miR-149-5p | GAL3ST3 |
| ADIRF-AS1 | hsa-miR-149-5p | SPEG |
| ADIRF-AS1 | hsa-miR-149-5p | LPP |
| ADIRF-AS1 | hsa-miR-149-5p | C7 |
| ADIRF-AS1 | hsa-miR-149-5p | GPR88 |
| ADIRF-AS1 | hsa-miR-149-5p | CLU |
| ADIRF-AS1 | hsa-miR-149-5p | PLAG1 |
| ADIRF-AS1 | hsa-miR-149-5p | SLC2A4 |
| ADIRF-AS1 | hsa-miR-149-5p | FIBIN |
| ADIRF-AS1 | hsa-miR-149-5p | SYT9 |
| ADIRF-AS1 | hsa-miR-149-5p | CAPN6 |
| ADIRF-AS1 | hsa-miR-149-5p | C11orf87 |
| ADIRF-AS1 | hsa-miR-149-5p | TNFAIP8L3 |
| ADIRF-AS1 | hsa-miR-149-5p | BTNL9 |
| ADIRF-AS1 | hsa-miR-149-5p | GRID1 |
| ADIRF-AS1 | hsa-miR-149-5p | ST6GALNAC3 |
| ADIRF-AS1 | hsa-miR-149-5p | SYNGR1 |
| ADIRF-AS1 | hsa-miR-149-5p | COL4A6 |
| ADIRF-AS1 | hsa-miR-149-5p | TSPAN18 |
| ADIRF-AS1 | hsa-miR-149-5p | SLC24A2 |
| ADIRF-AS1 | hsa-miR-149-5p | PRELP |
| ADIRF-AS1 | hsa-miR-149-5p | THNSL2 |
| ADIRF-AS1 | hsa-miR-149-5p | ATP2B2 |
| ADIRF-AS1 | hsa-miR-149-5p | MYOM1 |
| ADIRF-AS1 | hsa-miR-149-5p | ADAM33 |
| ADIRF-AS1 | hsa-miR-149-5p | DCX |
| ADIRF-AS1 | hsa-miR-149-5p | PLN |
| ADIRF-AS1 | hsa-miR-149-5p | ZNF483 |
| ADIRF-AS1 | hsa-miR-149-5p | NFIX |
| ADIRF-AS1 | hsa-miR-149-5p | MYLK |
| ADIRF-AS1 | hsa-miR-149-5p | LMOD1 |
| ADIRF-AS1 | hsa-miR-149-5p | DNM3 |
| ADIRF-AS1 | hsa-miR-149-5p | GPR161 |
| ADIRF-AS1 | hsa-miR-149-5p | SORBS1 |
| ADIRF-AS1 | hsa-miR-149-5p | SLC25A27 |
| ADIRF-AS1 | hsa-miR-149-5p | KCNB1 |
| ADIRF-AS1 | hsa-miR-149-5p | PAK3 |
| ADIRF-AS1 | hsa-miR-149-5p | WISP2 |
| ADIRF-AS1 | hsa-miR-149-5p | JPH2 |
| ADIRF-AS1 | hsa-miR-149-5p | RSPO1 |
| ADIRF-AS1 | hsa-miR-149-5p | PRKG1 |
| ADIRF-AS1 | hsa-miR-149-5p | GPR20 |
| ADIRF-AS1 | hsa-miR-149-5p | PKHD1L1 |
| ADIRF-AS1 | hsa-miR-149-5p | PTCHD1 |
| ADIRF-AS1 | hsa-miR-149-5p | DCLK1 |
| ADIRF-AS1 | hsa-miR-149-5p | BNC2 |
| ADIRF-AS1 | hsa-miR-149-5p | IGF2 |
| ADIRF-AS1 | hsa-miR-149-5p | CYS1 |
| ADIRF-AS1 | hsa-miR-149-5p | LGI2 |
| ADIRF-AS1 | hsa-miR-149-5p | SEMA5A |
| ADIRF-AS1 | hsa-miR-149-5p | CDON |
| ADIRF-AS1 | hsa-miR-149-5p | PRIMA1 |
| ADIRF-AS1 | hsa-miR-149-5p | MAPK10 |
| ADIRF-AS1 | hsa-miR-149-5p | KCNG1 |
| ADIRF-AS1 | hsa-miR-149-5p | ZNF208 |
| ADIRF-AS1 | hsa-miR-149-5p | MEIS2 |
| ADIRF-AS1 | hsa-miR-149-5p | NFASC |
| ADIRF-AS1 | hsa-miR-149-5p | DGKB |
| ADIRF-AS1 | hsa-miR-149-5p | SYNC |
| ADIRF-AS1 | hsa-miR-149-5p | LIMS2 |
| ADIRF-AS1 | hsa-miR-149-5p | MRVI1 |
| ADIRF-AS1 | hsa-miR-149-5p | ITGA11 |
| ADIRF-AS1 | hsa-miR-149-5p | GFRA1 |
| ADIRF-AS1 | hsa-miR-149-5p | MRGPRF |
| ADIRF-AS1 | hsa-miR-149-5p | SHISA6 |
| ADIRF-AS1 | hsa-miR-149-5p | TCEA3 |
| ADIRF-AS1 | hsa-miR-149-5p | MYH11 |
| ADIRF-AS1 | hsa-miR-149-5p | MUC15 |
| ADIRF-AS1 | hsa-miR-149-5p | FAM107A |
| ADIRF-AS1 | hsa-miR-149-5p | TCEAL4 |
| ADIRF-AS1 | hsa-miR-149-5p | SLC15A2 |
| ADIRF-AS1 | hsa-miR-149-5p | SORCS2 |
| ADIRF-AS1 | hsa-miR-149-5p | SLIT3 |
| ADIRF-AS1 | hsa-miR-149-5p | ANGPT1 |
| ADIRF-AS1 | hsa-miR-149-5p | NAALAD2 |
| ADIRF-AS1 | hsa-miR-149-5p | PIANP |
| ADIRF-AS1 | hsa-miR-149-5p | PLXNB3 |
| ADIRF-AS1 | hsa-miR-149-5p | FHL5 |
| ADIRF-AS1 | hsa-miR-149-5p | LDB3 |
| ADIRF-AS1 | hsa-miR-149-5p | NDUFA4L2 |
| ADIRF-AS1 | hsa-miR-149-5p | AHNAK2 |
| ADIRF-AS1 | hsa-miR-149-5p | DET1 |
| ADIRF-AS1 | hsa-miR-149-5p | RBFOX3 |
| ADIRF-AS1 | hsa-miR-149-5p | CCDC68 |
| ADIRF-AS1 | hsa-miR-149-5p | AOC3 |
| ADIRF-AS1 | hsa-miR-149-5p | PPP1R12B |
| MIR1-1HG-AS1 | hsa-miR-149-5p | INMT |
| MIR1-1HG-AS1 | hsa-miR-149-5p | FSTL3 |
| MIR1-1HG-AS1 | hsa-miR-149-5p | ADAM11 |
| MIR1-1HG-AS1 | hsa-miR-149-5p | CCDC80 |
| MIR1-1HG-AS1 | hsa-miR-149-5p | IGFBP5 |
| MIR1-1HG-AS1 | hsa-miR-149-5p | RAB9B |
| MIR1-1HG-AS1 | hsa-miR-149-5p | PTGIS |
| MIR1-1HG-AS1 | hsa-miR-149-5p | GATA5 |
| MIR1-1HG-AS1 | hsa-miR-149-5p | POPDC3 |
| MIR1-1HG-AS1 | hsa-miR-149-5p | RERG |
| MIR1-1HG-AS1 | hsa-miR-149-5p | PDE8B |
| MIR1-1HG-AS1 | hsa-miR-149-5p | PTH2R |
| MIR1-1HG-AS1 | hsa-miR-149-5p | SLC2A12 |
| MIR1-1HG-AS1 | hsa-miR-149-5p | RAB3C |
| MIR1-1HG-AS1 | hsa-miR-149-5p | UNC5D |
| MIR1-1HG-AS1 | hsa-miR-149-5p | TCF23 |
| MIR1-1HG-AS1 | hsa-miR-149-5p | MASP1 |
| MIR1-1HG-AS1 | hsa-miR-149-5p | TMEM132B |
| MIR1-1HG-AS1 | hsa-miR-149-5p | MSRB3 |
| MIR1-1HG-AS1 | hsa-miR-149-5p | GAL3ST3 |
| MIR1-1HG-AS1 | hsa-miR-149-5p | SPEG |
| MIR1-1HG-AS1 | hsa-miR-149-5p | LPP |
| MIR1-1HG-AS1 | hsa-miR-149-5p | C7 |
| MIR1-1HG-AS1 | hsa-miR-149-5p | GPR88 |
| MIR1-1HG-AS1 | hsa-miR-149-5p | CLU |
| MIR1-1HG-AS1 | hsa-miR-149-5p | PLAG1 |
| MIR1-1HG-AS1 | hsa-miR-149-5p | SLC2A4 |
| MIR1-1HG-AS1 | hsa-miR-149-5p | FIBIN |
| MIR1-1HG-AS1 | hsa-miR-149-5p | SYT9 |
| MIR1-1HG-AS1 | hsa-miR-149-5p | CAPN6 |
| MIR1-1HG-AS1 | hsa-miR-149-5p | C11orf87 |
| MIR1-1HG-AS1 | hsa-miR-149-5p | TNFAIP8L3 |
| MIR1-1HG-AS1 | hsa-miR-149-5p | BTNL9 |
| MIR1-1HG-AS1 | hsa-miR-149-5p | GRID1 |
| MIR1-1HG-AS1 | hsa-miR-149-5p | ST6GALNAC3 |
| MIR1-1HG-AS1 | hsa-miR-149-5p | SYNGR1 |
| MIR1-1HG-AS1 | hsa-miR-149-5p | COL4A6 |
| MIR1-1HG-AS1 | hsa-miR-149-5p | TSPAN18 |
| MIR1-1HG-AS1 | hsa-miR-149-5p | SLC24A2 |
| MIR1-1HG-AS1 | hsa-miR-149-5p | PRELP |
| MIR1-1HG-AS1 | hsa-miR-149-5p | THNSL2 |
| MIR1-1HG-AS1 | hsa-miR-149-5p | ATP2B2 |
| MIR1-1HG-AS1 | hsa-miR-149-5p | MYOM1 |
| MIR1-1HG-AS1 | hsa-miR-149-5p | ADAM33 |
| MIR1-1HG-AS1 | hsa-miR-149-5p | DCX |
| MIR1-1HG-AS1 | hsa-miR-149-5p | PLN |
| MIR1-1HG-AS1 | hsa-miR-149-5p | ZNF483 |
| MIR1-1HG-AS1 | hsa-miR-149-5p | NFIX |
| MIR1-1HG-AS1 | hsa-miR-149-5p | MYLK |
| MIR1-1HG-AS1 | hsa-miR-149-5p | LMOD1 |
| MIR1-1HG-AS1 | hsa-miR-149-5p | DNM3 |
| MIR1-1HG-AS1 | hsa-miR-149-5p | GPR161 |
| MIR1-1HG-AS1 | hsa-miR-149-5p | SORBS1 |
| MIR1-1HG-AS1 | hsa-miR-149-5p | SLC25A27 |
| MIR1-1HG-AS1 | hsa-miR-149-5p | KCNB1 |
| MIR1-1HG-AS1 | hsa-miR-149-5p | PAK3 |
| MIR1-1HG-AS1 | hsa-miR-149-5p | WISP2 |
| MIR1-1HG-AS1 | hsa-miR-149-5p | JPH2 |
| MIR1-1HG-AS1 | hsa-miR-149-5p | RSPO1 |
| MIR1-1HG-AS1 | hsa-miR-149-5p | PRKG1 |
| MIR1-1HG-AS1 | hsa-miR-149-5p | GPR20 |
| MIR1-1HG-AS1 | hsa-miR-149-5p | PKHD1L1 |
| MIR1-1HG-AS1 | hsa-miR-149-5p | PTCHD1 |
| MIR1-1HG-AS1 | hsa-miR-149-5p | DCLK1 |
| MIR1-1HG-AS1 | hsa-miR-149-5p | BNC2 |
| MIR1-1HG-AS1 | hsa-miR-149-5p | IGF2 |
| MIR1-1HG-AS1 | hsa-miR-149-5p | CYS1 |
| MIR1-1HG-AS1 | hsa-miR-149-5p | LGI2 |
| MIR1-1HG-AS1 | hsa-miR-149-5p | SEMA5A |
| MIR1-1HG-AS1 | hsa-miR-149-5p | CDON |
| MIR1-1HG-AS1 | hsa-miR-149-5p | PRIMA1 |
| MIR1-1HG-AS1 | hsa-miR-149-5p | MAPK10 |
| MIR1-1HG-AS1 | hsa-miR-149-5p | KCNG1 |
| MIR1-1HG-AS1 | hsa-miR-149-5p | ZNF208 |
| MIR1-1HG-AS1 | hsa-miR-149-5p | MEIS2 |
| MIR1-1HG-AS1 | hsa-miR-149-5p | NFASC |
| MIR1-1HG-AS1 | hsa-miR-149-5p | DGKB |
| MIR1-1HG-AS1 | hsa-miR-149-5p | SYNC |
| MIR1-1HG-AS1 | hsa-miR-149-5p | LIMS2 |
| MIR1-1HG-AS1 | hsa-miR-149-5p | MRVI1 |
| MIR1-1HG-AS1 | hsa-miR-149-5p | ITGA11 |
| MIR1-1HG-AS1 | hsa-miR-149-5p | GFRA1 |
| MIR1-1HG-AS1 | hsa-miR-149-5p | MRGPRF |
| MIR1-1HG-AS1 | hsa-miR-149-5p | SHISA6 |
| MIR1-1HG-AS1 | hsa-miR-149-5p | TCEA3 |
| MIR1-1HG-AS1 | hsa-miR-149-5p | MYH11 |
| MIR1-1HG-AS1 | hsa-miR-149-5p | MUC15 |
| MIR1-1HG-AS1 | hsa-miR-149-5p | FAM107A |
| MIR1-1HG-AS1 | hsa-miR-149-5p | TCEAL4 |
| MIR1-1HG-AS1 | hsa-miR-149-5p | SLC15A2 |
| MIR1-1HG-AS1 | hsa-miR-149-5p | SORCS2 |
| MIR1-1HG-AS1 | hsa-miR-149-5p | SLIT3 |
| MIR1-1HG-AS1 | hsa-miR-149-5p | ANGPT1 |
| MIR1-1HG-AS1 | hsa-miR-149-5p | NAALAD2 |
| MIR1-1HG-AS1 | hsa-miR-149-5p | PIANP |
| MIR1-1HG-AS1 | hsa-miR-149-5p | PLXNB3 |
| MIR1-1HG-AS1 | hsa-miR-149-5p | FHL5 |
| MIR1-1HG-AS1 | hsa-miR-149-5p | LDB3 |
| MIR1-1HG-AS1 | hsa-miR-149-5p | NDUFA4L2 |
| MIR1-1HG-AS1 | hsa-miR-149-5p | AHNAK2 |
| MIR1-1HG-AS1 | hsa-miR-149-5p | DET1 |
| MIR1-1HG-AS1 | hsa-miR-149-5p | RBFOX3 |
| MIR1-1HG-AS1 | hsa-miR-149-5p | CCDC68 |
| MIR1-1HG-AS1 | hsa-miR-149-5p | AOC3 |
| MIR1-1HG-AS1 | hsa-miR-149-5p | PPP1R12B |
| AC100803.2 | hsa-miR-149-5p | INMT |
| AC100803.2 | hsa-miR-149-5p | FSTL3 |
| AC100803.2 | hsa-miR-149-5p | ADAM11 |
| AC100803.2 | hsa-miR-149-5p | CCDC80 |
| AC100803.2 | hsa-miR-149-5p | IGFBP5 |
| AC100803.2 | hsa-miR-149-5p | RAB9B |
| AC100803.2 | hsa-miR-149-5p | PTGIS |
| AC100803.2 | hsa-miR-149-5p | GATA5 |
| AC100803.2 | hsa-miR-149-5p | POPDC3 |
| AC100803.2 | hsa-miR-149-5p | RERG |
| AC100803.2 | hsa-miR-149-5p | PDE8B |
| AC100803.2 | hsa-miR-149-5p | PTH2R |
| AC100803.2 | hsa-miR-149-5p | SLC2A12 |
| AC100803.2 | hsa-miR-149-5p | RAB3C |
| AC100803.2 | hsa-miR-149-5p | UNC5D |
| AC100803.2 | hsa-miR-149-5p | TCF23 |
| AC100803.2 | hsa-miR-149-5p | MASP1 |
| AC100803.2 | hsa-miR-149-5p | TMEM132B |
| AC100803.2 | hsa-miR-149-5p | MSRB3 |
| AC100803.2 | hsa-miR-149-5p | GAL3ST3 |
| AC100803.2 | hsa-miR-149-5p | SPEG |
| AC100803.2 | hsa-miR-149-5p | LPP |
| AC100803.2 | hsa-miR-149-5p | C7 |
| AC100803.2 | hsa-miR-149-5p | GPR88 |
| AC100803.2 | hsa-miR-149-5p | CLU |
| AC100803.2 | hsa-miR-149-5p | PLAG1 |
| AC100803.2 | hsa-miR-149-5p | SLC2A4 |
| AC100803.2 | hsa-miR-149-5p | FIBIN |
| AC100803.2 | hsa-miR-149-5p | SYT9 |
| AC100803.2 | hsa-miR-149-5p | CAPN6 |
| AC100803.2 | hsa-miR-149-5p | C11orf87 |
| AC100803.2 | hsa-miR-149-5p | TNFAIP8L3 |
| AC100803.2 | hsa-miR-149-5p | BTNL9 |
| AC100803.2 | hsa-miR-149-5p | GRID1 |
| AC100803.2 | hsa-miR-149-5p | ST6GALNAC3 |
| AC100803.2 | hsa-miR-149-5p | SYNGR1 |
| AC100803.2 | hsa-miR-149-5p | COL4A6 |
| AC100803.2 | hsa-miR-149-5p | TSPAN18 |
| AC100803.2 | hsa-miR-149-5p | SLC24A2 |
| AC100803.2 | hsa-miR-149-5p | PRELP |
| AC100803.2 | hsa-miR-149-5p | THNSL2 |
| AC100803.2 | hsa-miR-149-5p | ATP2B2 |
| AC100803.2 | hsa-miR-149-5p | MYOM1 |
| AC100803.2 | hsa-miR-149-5p | ADAM33 |
| AC100803.2 | hsa-miR-149-5p | DCX |
| AC100803.2 | hsa-miR-149-5p | PLN |
| AC100803.2 | hsa-miR-149-5p | ZNF483 |
| AC100803.2 | hsa-miR-149-5p | NFIX |
| AC100803.2 | hsa-miR-149-5p | MYLK |
| AC100803.2 | hsa-miR-149-5p | LMOD1 |
| AC100803.2 | hsa-miR-149-5p | DNM3 |
| AC100803.2 | hsa-miR-149-5p | GPR161 |
| AC100803.2 | hsa-miR-149-5p | SORBS1 |
| AC100803.2 | hsa-miR-149-5p | SLC25A27 |
| AC100803.2 | hsa-miR-149-5p | KCNB1 |
| AC100803.2 | hsa-miR-149-5p | PAK3 |
| AC100803.2 | hsa-miR-149-5p | WISP2 |
| AC100803.2 | hsa-miR-149-5p | JPH2 |
| AC100803.2 | hsa-miR-149-5p | RSPO1 |
| AC100803.2 | hsa-miR-149-5p | PRKG1 |
| AC100803.2 | hsa-miR-149-5p | GPR20 |
| AC100803.2 | hsa-miR-149-5p | PKHD1L1 |
| AC100803.2 | hsa-miR-149-5p | PTCHD1 |
| AC100803.2 | hsa-miR-149-5p | DCLK1 |
| AC100803.2 | hsa-miR-149-5p | BNC2 |
| AC100803.2 | hsa-miR-149-5p | IGF2 |
| AC100803.2 | hsa-miR-149-5p | CYS1 |
| AC100803.2 | hsa-miR-149-5p | LGI2 |
| AC100803.2 | hsa-miR-149-5p | SEMA5A |
| AC100803.2 | hsa-miR-149-5p | CDON |
| AC100803.2 | hsa-miR-149-5p | PRIMA1 |
| AC100803.2 | hsa-miR-149-5p | MAPK10 |
| AC100803.2 | hsa-miR-149-5p | KCNG1 |
| AC100803.2 | hsa-miR-149-5p | ZNF208 |
| AC100803.2 | hsa-miR-149-5p | MEIS2 |
| AC100803.2 | hsa-miR-149-5p | NFASC |
| AC100803.2 | hsa-miR-149-5p | DGKB |
| AC100803.2 | hsa-miR-149-5p | SYNC |
| AC100803.2 | hsa-miR-149-5p | LIMS2 |
| AC100803.2 | hsa-miR-149-5p | MRVI1 |
| AC100803.2 | hsa-miR-149-5p | ITGA11 |
| AC100803.2 | hsa-miR-149-5p | GFRA1 |
| AC100803.2 | hsa-miR-149-5p | MRGPRF |
| AC100803.2 | hsa-miR-149-5p | SHISA6 |
| AC100803.2 | hsa-miR-149-5p | TCEA3 |
| AC100803.2 | hsa-miR-149-5p | MYH11 |
| AC100803.2 | hsa-miR-149-5p | MUC15 |
| AC100803.2 | hsa-miR-149-5p | FAM107A |
| AC100803.2 | hsa-miR-149-5p | TCEAL4 |
| AC100803.2 | hsa-miR-149-5p | SLC15A2 |
| AC100803.2 | hsa-miR-149-5p | SORCS2 |
| AC100803.2 | hsa-miR-149-5p | SLIT3 |
| AC100803.2 | hsa-miR-149-5p | ANGPT1 |
| AC100803.2 | hsa-miR-149-5p | NAALAD2 |
| AC100803.2 | hsa-miR-149-5p | PIANP |
| AC100803.2 | hsa-miR-149-5p | PLXNB3 |
| AC100803.2 | hsa-miR-149-5p | FHL5 |
| AC100803.2 | hsa-miR-149-5p | LDB3 |
| AC100803.2 | hsa-miR-149-5p | NDUFA4L2 |
| AC100803.2 | hsa-miR-149-5p | AHNAK2 |
| AC100803.2 | hsa-miR-149-5p | DET1 |
| AC100803.2 | hsa-miR-149-5p | RBFOX3 |
| AC100803.2 | hsa-miR-149-5p | CCDC68 |
| AC100803.2 | hsa-miR-149-5p | AOC3 |
| AC100803.2 | hsa-miR-149-5p | PPP1R12B |
| AC098679.2 | hsa-miR-149-5p | INMT |
| AC098679.2 | hsa-miR-149-5p | FSTL3 |
| AC098679.2 | hsa-miR-149-5p | ADAM11 |
| AC098679.2 | hsa-miR-149-5p | CCDC80 |
| AC098679.2 | hsa-miR-149-5p | IGFBP5 |
| AC098679.2 | hsa-miR-149-5p | RAB9B |
| AC098679.2 | hsa-miR-149-5p | PTGIS |
| AC098679.2 | hsa-miR-149-5p | GATA5 |
| AC098679.2 | hsa-miR-149-5p | POPDC3 |
| AC098679.2 | hsa-miR-149-5p | RERG |
| AC098679.2 | hsa-miR-149-5p | PDE8B |
| AC098679.2 | hsa-miR-149-5p | PTH2R |
| AC098679.2 | hsa-miR-149-5p | SLC2A12 |
| AC098679.2 | hsa-miR-149-5p | RAB3C |
| AC098679.2 | hsa-miR-149-5p | UNC5D |
| AC098679.2 | hsa-miR-149-5p | TCF23 |
| AC098679.2 | hsa-miR-149-5p | MASP1 |
| AC098679.2 | hsa-miR-149-5p | TMEM132B |
| AC098679.2 | hsa-miR-149-5p | MSRB3 |
| AC098679.2 | hsa-miR-149-5p | GAL3ST3 |
| AC098679.2 | hsa-miR-149-5p | SPEG |
| AC098679.2 | hsa-miR-149-5p | LPP |
| AC098679.2 | hsa-miR-149-5p | C7 |
| AC098679.2 | hsa-miR-149-5p | GPR88 |
| AC098679.2 | hsa-miR-149-5p | CLU |
| AC098679.2 | hsa-miR-149-5p | PLAG1 |
| AC098679.2 | hsa-miR-149-5p | SLC2A4 |
| AC098679.2 | hsa-miR-149-5p | FIBIN |
| AC098679.2 | hsa-miR-149-5p | SYT9 |
| AC098679.2 | hsa-miR-149-5p | CAPN6 |
| AC098679.2 | hsa-miR-149-5p | C11orf87 |
| AC098679.2 | hsa-miR-149-5p | TNFAIP8L3 |
| AC098679.2 | hsa-miR-149-5p | BTNL9 |
| AC098679.2 | hsa-miR-149-5p | GRID1 |
| AC098679.2 | hsa-miR-149-5p | ST6GALNAC3 |
| AC098679.2 | hsa-miR-149-5p | SYNGR1 |
| AC098679.2 | hsa-miR-149-5p | COL4A6 |
| AC098679.2 | hsa-miR-149-5p | TSPAN18 |
| AC098679.2 | hsa-miR-149-5p | SLC24A2 |
| AC098679.2 | hsa-miR-149-5p | PRELP |
| AC098679.2 | hsa-miR-149-5p | THNSL2 |
| AC098679.2 | hsa-miR-149-5p | ATP2B2 |
| AC098679.2 | hsa-miR-149-5p | MYOM1 |
| AC098679.2 | hsa-miR-149-5p | ADAM33 |
| AC098679.2 | hsa-miR-149-5p | DCX |
| AC098679.2 | hsa-miR-149-5p | PLN |
| AC098679.2 | hsa-miR-149-5p | ZNF483 |
| AC098679.2 | hsa-miR-149-5p | NFIX |
| AC098679.2 | hsa-miR-149-5p | MYLK |
| AC098679.2 | hsa-miR-149-5p | LMOD1 |
| AC098679.2 | hsa-miR-149-5p | DNM3 |
| AC098679.2 | hsa-miR-149-5p | GPR161 |
| AC098679.2 | hsa-miR-149-5p | SORBS1 |
| AC098679.2 | hsa-miR-149-5p | SLC25A27 |
| AC098679.2 | hsa-miR-149-5p | KCNB1 |
| AC098679.2 | hsa-miR-149-5p | PAK3 |
| AC098679.2 | hsa-miR-149-5p | WISP2 |
| AC098679.2 | hsa-miR-149-5p | JPH2 |
| AC098679.2 | hsa-miR-149-5p | RSPO1 |
| AC098679.2 | hsa-miR-149-5p | PRKG1 |
| AC098679.2 | hsa-miR-149-5p | GPR20 |
| AC098679.2 | hsa-miR-149-5p | PKHD1L1 |
| AC098679.2 | hsa-miR-149-5p | PTCHD1 |
| AC098679.2 | hsa-miR-149-5p | DCLK1 |
| AC098679.2 | hsa-miR-149-5p | BNC2 |
| AC098679.2 | hsa-miR-149-5p | IGF2 |
| AC098679.2 | hsa-miR-149-5p | CYS1 |
| AC098679.2 | hsa-miR-149-5p | LGI2 |
| AC098679.2 | hsa-miR-149-5p | SEMA5A |
| AC098679.2 | hsa-miR-149-5p | CDON |
| AC098679.2 | hsa-miR-149-5p | PRIMA1 |
| AC098679.2 | hsa-miR-149-5p | MAPK10 |
| AC098679.2 | hsa-miR-149-5p | KCNG1 |
| AC098679.2 | hsa-miR-149-5p | ZNF208 |
| AC098679.2 | hsa-miR-149-5p | MEIS2 |
| AC098679.2 | hsa-miR-149-5p | NFASC |
| AC098679.2 | hsa-miR-149-5p | DGKB |
| AC098679.2 | hsa-miR-149-5p | SYNC |
| AC098679.2 | hsa-miR-149-5p | LIMS2 |
| AC098679.2 | hsa-miR-149-5p | MRVI1 |
| AC098679.2 | hsa-miR-149-5p | ITGA11 |
| AC098679.2 | hsa-miR-149-5p | GFRA1 |
| AC098679.2 | hsa-miR-149-5p | MRGPRF |
| AC098679.2 | hsa-miR-149-5p | SHISA6 |
| AC098679.2 | hsa-miR-149-5p | TCEA3 |
| AC098679.2 | hsa-miR-149-5p | MYH11 |
| AC098679.2 | hsa-miR-149-5p | MUC15 |
| AC098679.2 | hsa-miR-149-5p | FAM107A |
| AC098679.2 | hsa-miR-149-5p | TCEAL4 |
| AC098679.2 | hsa-miR-149-5p | SLC15A2 |
| AC098679.2 | hsa-miR-149-5p | SORCS2 |
| AC098679.2 | hsa-miR-149-5p | SLIT3 |
| AC098679.2 | hsa-miR-149-5p | ANGPT1 |
| AC098679.2 | hsa-miR-149-5p | NAALAD2 |
| AC098679.2 | hsa-miR-149-5p | PIANP |
| AC098679.2 | hsa-miR-149-5p | PLXNB3 |
| AC098679.2 | hsa-miR-149-5p | FHL5 |
| AC098679.2 | hsa-miR-149-5p | LDB3 |
| AC098679.2 | hsa-miR-149-5p | NDUFA4L2 |
| AC098679.2 | hsa-miR-149-5p | AHNAK2 |
| AC098679.2 | hsa-miR-149-5p | DET1 |
| AC098679.2 | hsa-miR-149-5p | RBFOX3 |
| AC098679.2 | hsa-miR-149-5p | CCDC68 |
| AC098679.2 | hsa-miR-149-5p | AOC3 |
| AC098679.2 | hsa-miR-149-5p | PPP1R12B |
| GOLGA2P10 | hsa-miR-149-5p | INMT |
| GOLGA2P10 | hsa-miR-149-5p | FSTL3 |
| GOLGA2P10 | hsa-miR-149-5p | ADAM11 |
| GOLGA2P10 | hsa-miR-149-5p | CCDC80 |
| GOLGA2P10 | hsa-miR-149-5p | IGFBP5 |
| GOLGA2P10 | hsa-miR-149-5p | RAB9B |
| GOLGA2P10 | hsa-miR-149-5p | PTGIS |
| GOLGA2P10 | hsa-miR-149-5p | GATA5 |
| GOLGA2P10 | hsa-miR-149-5p | POPDC3 |
| GOLGA2P10 | hsa-miR-149-5p | RERG |
| GOLGA2P10 | hsa-miR-149-5p | PDE8B |
| GOLGA2P10 | hsa-miR-149-5p | PTH2R |
| GOLGA2P10 | hsa-miR-149-5p | SLC2A12 |
| GOLGA2P10 | hsa-miR-149-5p | RAB3C |
| GOLGA2P10 | hsa-miR-149-5p | UNC5D |
| GOLGA2P10 | hsa-miR-149-5p | TCF23 |
| GOLGA2P10 | hsa-miR-149-5p | MASP1 |
| GOLGA2P10 | hsa-miR-149-5p | TMEM132B |
| GOLGA2P10 | hsa-miR-149-5p | MSRB3 |
| GOLGA2P10 | hsa-miR-149-5p | GAL3ST3 |
| GOLGA2P10 | hsa-miR-149-5p | SPEG |
| GOLGA2P10 | hsa-miR-149-5p | LPP |
| GOLGA2P10 | hsa-miR-149-5p | C7 |
| GOLGA2P10 | hsa-miR-149-5p | GPR88 |
| GOLGA2P10 | hsa-miR-149-5p | CLU |
| GOLGA2P10 | hsa-miR-149-5p | PLAG1 |
| GOLGA2P10 | hsa-miR-149-5p | SLC2A4 |
| GOLGA2P10 | hsa-miR-149-5p | FIBIN |
| GOLGA2P10 | hsa-miR-149-5p | SYT9 |
| GOLGA2P10 | hsa-miR-149-5p | CAPN6 |
| GOLGA2P10 | hsa-miR-149-5p | C11orf87 |
| GOLGA2P10 | hsa-miR-149-5p | TNFAIP8L3 |
| GOLGA2P10 | hsa-miR-149-5p | BTNL9 |
| GOLGA2P10 | hsa-miR-149-5p | GRID1 |
| GOLGA2P10 | hsa-miR-149-5p | ST6GALNAC3 |
| GOLGA2P10 | hsa-miR-149-5p | SYNGR1 |
| GOLGA2P10 | hsa-miR-149-5p | COL4A6 |
| GOLGA2P10 | hsa-miR-149-5p | TSPAN18 |
| GOLGA2P10 | hsa-miR-149-5p | SLC24A2 |
| GOLGA2P10 | hsa-miR-149-5p | PRELP |
| GOLGA2P10 | hsa-miR-149-5p | THNSL2 |
| GOLGA2P10 | hsa-miR-149-5p | ATP2B2 |
| GOLGA2P10 | hsa-miR-149-5p | MYOM1 |
| GOLGA2P10 | hsa-miR-149-5p | ADAM33 |
| GOLGA2P10 | hsa-miR-149-5p | DCX |
| GOLGA2P10 | hsa-miR-149-5p | PLN |
| GOLGA2P10 | hsa-miR-149-5p | ZNF483 |
| GOLGA2P10 | hsa-miR-149-5p | NFIX |
| GOLGA2P10 | hsa-miR-149-5p | MYLK |
| GOLGA2P10 | hsa-miR-149-5p | LMOD1 |
| GOLGA2P10 | hsa-miR-149-5p | DNM3 |
| GOLGA2P10 | hsa-miR-149-5p | GPR161 |
| GOLGA2P10 | hsa-miR-149-5p | SORBS1 |
| GOLGA2P10 | hsa-miR-149-5p | SLC25A27 |
| GOLGA2P10 | hsa-miR-149-5p | KCNB1 |
| GOLGA2P10 | hsa-miR-149-5p | PAK3 |
| GOLGA2P10 | hsa-miR-149-5p | WISP2 |
| GOLGA2P10 | hsa-miR-149-5p | JPH2 |
| GOLGA2P10 | hsa-miR-149-5p | RSPO1 |
| GOLGA2P10 | hsa-miR-149-5p | PRKG1 |
| GOLGA2P10 | hsa-miR-149-5p | GPR20 |
| GOLGA2P10 | hsa-miR-149-5p | PKHD1L1 |
| GOLGA2P10 | hsa-miR-149-5p | PTCHD1 |
| GOLGA2P10 | hsa-miR-149-5p | DCLK1 |
| GOLGA2P10 | hsa-miR-149-5p | BNC2 |
| GOLGA2P10 | hsa-miR-149-5p | IGF2 |
| GOLGA2P10 | hsa-miR-149-5p | CYS1 |
| GOLGA2P10 | hsa-miR-149-5p | LGI2 |
| GOLGA2P10 | hsa-miR-149-5p | SEMA5A |
| GOLGA2P10 | hsa-miR-149-5p | CDON |
| GOLGA2P10 | hsa-miR-149-5p | PRIMA1 |
| GOLGA2P10 | hsa-miR-149-5p | MAPK10 |
| GOLGA2P10 | hsa-miR-149-5p | KCNG1 |
| GOLGA2P10 | hsa-miR-149-5p | ZNF208 |
| GOLGA2P10 | hsa-miR-149-5p | MEIS2 |
| GOLGA2P10 | hsa-miR-149-5p | NFASC |
| GOLGA2P10 | hsa-miR-149-5p | DGKB |
| GOLGA2P10 | hsa-miR-149-5p | SYNC |
| GOLGA2P10 | hsa-miR-149-5p | LIMS2 |
| GOLGA2P10 | hsa-miR-149-5p | MRVI1 |
| GOLGA2P10 | hsa-miR-149-5p | ITGA11 |
| GOLGA2P10 | hsa-miR-149-5p | GFRA1 |
| GOLGA2P10 | hsa-miR-149-5p | MRGPRF |
| GOLGA2P10 | hsa-miR-149-5p | SHISA6 |
| GOLGA2P10 | hsa-miR-149-5p | TCEA3 |
| GOLGA2P10 | hsa-miR-149-5p | MYH11 |
| GOLGA2P10 | hsa-miR-149-5p | MUC15 |
| GOLGA2P10 | hsa-miR-149-5p | FAM107A |
| GOLGA2P10 | hsa-miR-149-5p | TCEAL4 |
| GOLGA2P10 | hsa-miR-149-5p | SLC15A2 |
| GOLGA2P10 | hsa-miR-149-5p | SORCS2 |
| GOLGA2P10 | hsa-miR-149-5p | SLIT3 |
| GOLGA2P10 | hsa-miR-149-5p | ANGPT1 |
| GOLGA2P10 | hsa-miR-149-5p | NAALAD2 |
| GOLGA2P10 | hsa-miR-149-5p | PIANP |
| GOLGA2P10 | hsa-miR-149-5p | PLXNB3 |
| GOLGA2P10 | hsa-miR-149-5p | FHL5 |
| GOLGA2P10 | hsa-miR-149-5p | LDB3 |
| GOLGA2P10 | hsa-miR-149-5p | NDUFA4L2 |
| GOLGA2P10 | hsa-miR-149-5p | AHNAK2 |
| GOLGA2P10 | hsa-miR-149-5p | DET1 |
| GOLGA2P10 | hsa-miR-149-5p | RBFOX3 |
| GOLGA2P10 | hsa-miR-149-5p | CCDC68 |
| GOLGA2P10 | hsa-miR-149-5p | AOC3 |
| GOLGA2P10 | hsa-miR-149-5p | PPP1R12B |
| Z73965.1 | hsa-miR-149-5p | INMT |
| Z73965.1 | hsa-miR-149-5p | FSTL3 |
| Z73965.1 | hsa-miR-149-5p | ADAM11 |
| Z73965.1 | hsa-miR-149-5p | CCDC80 |
| Z73965.1 | hsa-miR-149-5p | IGFBP5 |
| Z73965.1 | hsa-miR-149-5p | RAB9B |
| Z73965.1 | hsa-miR-149-5p | PTGIS |
| Z73965.1 | hsa-miR-149-5p | GATA5 |
| Z73965.1 | hsa-miR-149-5p | POPDC3 |
| Z73965.1 | hsa-miR-149-5p | RERG |
| Z73965.1 | hsa-miR-149-5p | PDE8B |
| Z73965.1 | hsa-miR-149-5p | PTH2R |
| Z73965.1 | hsa-miR-149-5p | SLC2A12 |
| Z73965.1 | hsa-miR-149-5p | RAB3C |
| Z73965.1 | hsa-miR-149-5p | UNC5D |
| Z73965.1 | hsa-miR-149-5p | TCF23 |
| Z73965.1 | hsa-miR-149-5p | MASP1 |
| Z73965.1 | hsa-miR-149-5p | TMEM132B |
| Z73965.1 | hsa-miR-149-5p | MSRB3 |
| Z73965.1 | hsa-miR-149-5p | GAL3ST3 |
| Z73965.1 | hsa-miR-149-5p | SPEG |
| Z73965.1 | hsa-miR-149-5p | LPP |
| Z73965.1 | hsa-miR-149-5p | C7 |
| Z73965.1 | hsa-miR-149-5p | GPR88 |
| Z73965.1 | hsa-miR-149-5p | CLU |
| Z73965.1 | hsa-miR-149-5p | PLAG1 |
| Z73965.1 | hsa-miR-149-5p | SLC2A4 |
| Z73965.1 | hsa-miR-149-5p | FIBIN |
| Z73965.1 | hsa-miR-149-5p | SYT9 |
| Z73965.1 | hsa-miR-149-5p | CAPN6 |
| Z73965.1 | hsa-miR-149-5p | C11orf87 |
| Z73965.1 | hsa-miR-149-5p | TNFAIP8L3 |
| Z73965.1 | hsa-miR-149-5p | BTNL9 |
| Z73965.1 | hsa-miR-149-5p | GRID1 |
| Z73965.1 | hsa-miR-149-5p | ST6GALNAC3 |
| Z73965.1 | hsa-miR-149-5p | SYNGR1 |
| Z73965.1 | hsa-miR-149-5p | COL4A6 |
| Z73965.1 | hsa-miR-149-5p | TSPAN18 |
| Z73965.1 | hsa-miR-149-5p | SLC24A2 |
| Z73965.1 | hsa-miR-149-5p | PRELP |
| Z73965.1 | hsa-miR-149-5p | THNSL2 |
| Z73965.1 | hsa-miR-149-5p | ATP2B2 |
| Z73965.1 | hsa-miR-149-5p | MYOM1 |
| Z73965.1 | hsa-miR-149-5p | ADAM33 |
| Z73965.1 | hsa-miR-149-5p | DCX |
| Z73965.1 | hsa-miR-149-5p | PLN |
| Z73965.1 | hsa-miR-149-5p | ZNF483 |
| Z73965.1 | hsa-miR-149-5p | NFIX |
| Z73965.1 | hsa-miR-149-5p | MYLK |
| Z73965.1 | hsa-miR-149-5p | LMOD1 |
| Z73965.1 | hsa-miR-149-5p | DNM3 |
| Z73965.1 | hsa-miR-149-5p | GPR161 |
| Z73965.1 | hsa-miR-149-5p | SORBS1 |
| Z73965.1 | hsa-miR-149-5p | SLC25A27 |
| Z73965.1 | hsa-miR-149-5p | KCNB1 |
| Z73965.1 | hsa-miR-149-5p | PAK3 |
| Z73965.1 | hsa-miR-149-5p | WISP2 |
| Z73965.1 | hsa-miR-149-5p | JPH2 |
| Z73965.1 | hsa-miR-149-5p | RSPO1 |
| Z73965.1 | hsa-miR-149-5p | PRKG1 |
| Z73965.1 | hsa-miR-149-5p | GPR20 |
| Z73965.1 | hsa-miR-149-5p | PKHD1L1 |
| Z73965.1 | hsa-miR-149-5p | PTCHD1 |
| Z73965.1 | hsa-miR-149-5p | DCLK1 |
| Z73965.1 | hsa-miR-149-5p | BNC2 |
| Z73965.1 | hsa-miR-149-5p | IGF2 |
| Z73965.1 | hsa-miR-149-5p | CYS1 |
| Z73965.1 | hsa-miR-149-5p | LGI2 |
| Z73965.1 | hsa-miR-149-5p | SEMA5A |
| Z73965.1 | hsa-miR-149-5p | CDON |
| Z73965.1 | hsa-miR-149-5p | PRIMA1 |
| Z73965.1 | hsa-miR-149-5p | MAPK10 |
| Z73965.1 | hsa-miR-149-5p | KCNG1 |
| Z73965.1 | hsa-miR-149-5p | ZNF208 |
| Z73965.1 | hsa-miR-149-5p | MEIS2 |
| Z73965.1 | hsa-miR-149-5p | NFASC |
| Z73965.1 | hsa-miR-149-5p | DGKB |
| Z73965.1 | hsa-miR-149-5p | SYNC |
| Z73965.1 | hsa-miR-149-5p | LIMS2 |
| Z73965.1 | hsa-miR-149-5p | MRVI1 |
| Z73965.1 | hsa-miR-149-5p | ITGA11 |
| Z73965.1 | hsa-miR-149-5p | GFRA1 |
| Z73965.1 | hsa-miR-149-5p | MRGPRF |
| Z73965.1 | hsa-miR-149-5p | SHISA6 |
| Z73965.1 | hsa-miR-149-5p | TCEA3 |
| Z73965.1 | hsa-miR-149-5p | MYH11 |
| Z73965.1 | hsa-miR-149-5p | MUC15 |
| Z73965.1 | hsa-miR-149-5p | FAM107A |
| Z73965.1 | hsa-miR-149-5p | TCEAL4 |
| Z73965.1 | hsa-miR-149-5p | SLC15A2 |
| Z73965.1 | hsa-miR-149-5p | SORCS2 |
| Z73965.1 | hsa-miR-149-5p | SLIT3 |
| Z73965.1 | hsa-miR-149-5p | ANGPT1 |
| Z73965.1 | hsa-miR-149-5p | NAALAD2 |
| Z73965.1 | hsa-miR-149-5p | PIANP |
| Z73965.1 | hsa-miR-149-5p | PLXNB3 |
| Z73965.1 | hsa-miR-149-5p | FHL5 |
| Z73965.1 | hsa-miR-149-5p | LDB3 |
| Z73965.1 | hsa-miR-149-5p | NDUFA4L2 |
| Z73965.1 | hsa-miR-149-5p | AHNAK2 |
| Z73965.1 | hsa-miR-149-5p | DET1 |
| Z73965.1 | hsa-miR-149-5p | RBFOX3 |
| Z73965.1 | hsa-miR-149-5p | CCDC68 |
| Z73965.1 | hsa-miR-149-5p | AOC3 |
| Z73965.1 | hsa-miR-149-5p | PPP1R12B |
| HRAT92 | hsa-miR-149-5p | INMT |
| HRAT92 | hsa-miR-149-5p | FSTL3 |
| HRAT92 | hsa-miR-149-5p | ADAM11 |
| HRAT92 | hsa-miR-149-5p | CCDC80 |
| HRAT92 | hsa-miR-149-5p | IGFBP5 |
| HRAT92 | hsa-miR-149-5p | RAB9B |
| HRAT92 | hsa-miR-149-5p | PTGIS |
| HRAT92 | hsa-miR-149-5p | GATA5 |
| HRAT92 | hsa-miR-149-5p | POPDC3 |
| HRAT92 | hsa-miR-149-5p | RERG |
| HRAT92 | hsa-miR-149-5p | PDE8B |
| HRAT92 | hsa-miR-149-5p | PTH2R |
| HRAT92 | hsa-miR-149-5p | SLC2A12 |
| HRAT92 | hsa-miR-149-5p | RAB3C |
| HRAT92 | hsa-miR-149-5p | UNC5D |
| HRAT92 | hsa-miR-149-5p | TCF23 |
| HRAT92 | hsa-miR-149-5p | MASP1 |
| HRAT92 | hsa-miR-149-5p | TMEM132B |
| HRAT92 | hsa-miR-149-5p | MSRB3 |
| HRAT92 | hsa-miR-149-5p | GAL3ST3 |
| HRAT92 | hsa-miR-149-5p | SPEG |
| HRAT92 | hsa-miR-149-5p | LPP |
| HRAT92 | hsa-miR-149-5p | C7 |
| HRAT92 | hsa-miR-149-5p | GPR88 |
| HRAT92 | hsa-miR-149-5p | CLU |
| HRAT92 | hsa-miR-149-5p | PLAG1 |
| HRAT92 | hsa-miR-149-5p | SLC2A4 |
| HRAT92 | hsa-miR-149-5p | FIBIN |
| HRAT92 | hsa-miR-149-5p | SYT9 |
| HRAT92 | hsa-miR-149-5p | CAPN6 |
| HRAT92 | hsa-miR-149-5p | C11orf87 |
| HRAT92 | hsa-miR-149-5p | TNFAIP8L3 |
| HRAT92 | hsa-miR-149-5p | BTNL9 |
| HRAT92 | hsa-miR-149-5p | GRID1 |
| HRAT92 | hsa-miR-149-5p | ST6GALNAC3 |
| HRAT92 | hsa-miR-149-5p | SYNGR1 |
| HRAT92 | hsa-miR-149-5p | COL4A6 |
| HRAT92 | hsa-miR-149-5p | TSPAN18 |
| HRAT92 | hsa-miR-149-5p | SLC24A2 |
| HRAT92 | hsa-miR-149-5p | PRELP |
| HRAT92 | hsa-miR-149-5p | THNSL2 |
| HRAT92 | hsa-miR-149-5p | ATP2B2 |
| HRAT92 | hsa-miR-149-5p | MYOM1 |
| HRAT92 | hsa-miR-149-5p | ADAM33 |
| HRAT92 | hsa-miR-149-5p | DCX |
| HRAT92 | hsa-miR-149-5p | PLN |
| HRAT92 | hsa-miR-149-5p | ZNF483 |
| HRAT92 | hsa-miR-149-5p | NFIX |
| HRAT92 | hsa-miR-149-5p | MYLK |
| HRAT92 | hsa-miR-149-5p | LMOD1 |
| HRAT92 | hsa-miR-149-5p | DNM3 |
| HRAT92 | hsa-miR-149-5p | GPR161 |
| HRAT92 | hsa-miR-149-5p | SORBS1 |
| HRAT92 | hsa-miR-149-5p | SLC25A27 |
| HRAT92 | hsa-miR-149-5p | KCNB1 |
| HRAT92 | hsa-miR-149-5p | PAK3 |
| HRAT92 | hsa-miR-149-5p | WISP2 |
| HRAT92 | hsa-miR-149-5p | JPH2 |
| HRAT92 | hsa-miR-149-5p | RSPO1 |
| HRAT92 | hsa-miR-149-5p | PRKG1 |
| HRAT92 | hsa-miR-149-5p | GPR20 |
| HRAT92 | hsa-miR-149-5p | PKHD1L1 |
| HRAT92 | hsa-miR-149-5p | PTCHD1 |
| HRAT92 | hsa-miR-149-5p | DCLK1 |
| HRAT92 | hsa-miR-149-5p | BNC2 |
| HRAT92 | hsa-miR-149-5p | IGF2 |
| HRAT92 | hsa-miR-149-5p | CYS1 |
| HRAT92 | hsa-miR-149-5p | LGI2 |
| HRAT92 | hsa-miR-149-5p | SEMA5A |
| HRAT92 | hsa-miR-149-5p | CDON |
| HRAT92 | hsa-miR-149-5p | PRIMA1 |
| HRAT92 | hsa-miR-149-5p | MAPK10 |
| HRAT92 | hsa-miR-149-5p | KCNG1 |
| HRAT92 | hsa-miR-149-5p | ZNF208 |
| HRAT92 | hsa-miR-149-5p | MEIS2 |
| HRAT92 | hsa-miR-149-5p | NFASC |
| HRAT92 | hsa-miR-149-5p | DGKB |
| HRAT92 | hsa-miR-149-5p | SYNC |
| HRAT92 | hsa-miR-149-5p | LIMS2 |
| HRAT92 | hsa-miR-149-5p | MRVI1 |
| HRAT92 | hsa-miR-149-5p | ITGA11 |
| HRAT92 | hsa-miR-149-5p | GFRA1 |
| HRAT92 | hsa-miR-149-5p | MRGPRF |
| HRAT92 | hsa-miR-149-5p | SHISA6 |
| HRAT92 | hsa-miR-149-5p | TCEA3 |
| HRAT92 | hsa-miR-149-5p | MYH11 |
| HRAT92 | hsa-miR-149-5p | MUC15 |
| HRAT92 | hsa-miR-149-5p | FAM107A |
| HRAT92 | hsa-miR-149-5p | TCEAL4 |
| HRAT92 | hsa-miR-149-5p | SLC15A2 |
| HRAT92 | hsa-miR-149-5p | SORCS2 |
| HRAT92 | hsa-miR-149-5p | SLIT3 |
| HRAT92 | hsa-miR-149-5p | ANGPT1 |
| HRAT92 | hsa-miR-149-5p | NAALAD2 |
| HRAT92 | hsa-miR-149-5p | PIANP |
| HRAT92 | hsa-miR-149-5p | PLXNB3 |
| HRAT92 | hsa-miR-149-5p | FHL5 |
| HRAT92 | hsa-miR-149-5p | LDB3 |
| HRAT92 | hsa-miR-149-5p | NDUFA4L2 |
| HRAT92 | hsa-miR-149-5p | AHNAK2 |
| HRAT92 | hsa-miR-149-5p | DET1 |
| HRAT92 | hsa-miR-149-5p | RBFOX3 |
| HRAT92 | hsa-miR-149-5p | CCDC68 |
| HRAT92 | hsa-miR-149-5p | AOC3 |
| HRAT92 | hsa-miR-149-5p | PPP1R12B |
| AC098679.2 | hsa-miR-652-3p | NRK |
| AC098679.2 | hsa-miR-652-3p | GNAZ |
| AC098679.2 | hsa-miR-652-3p | PI15 |
| AC098679.2 | hsa-miR-652-3p | PLAG1 |
| AC098679.2 | hsa-miR-652-3p | DMRTA1 |
| AC098679.2 | hsa-miR-652-3p | PDLIM7 |
| AC098679.2 | hsa-miR-652-3p | MFAP5 |
| AC098679.2 | hsa-miR-652-3p | CNR1 |
| AC098679.2 | hsa-miR-652-3p | GPR161 |
| AC098679.2 | hsa-miR-652-3p | PTGFR |
| AC098679.2 | hsa-miR-652-3p | LRRC1 |
| AC098679.2 | hsa-miR-652-3p | KCNB1 |
| AC098679.2 | hsa-miR-652-3p | ADIRF |
| AC098679.2 | hsa-miR-652-3p | PTCHD1 |
| AC098679.2 | hsa-miR-652-3p | KCNG1 |
| AC098679.2 | hsa-miR-652-3p | NFASC |
| ADIRF-AS1 | hsa-miR-874-3p | FSTL3 |
| ADIRF-AS1 | hsa-miR-874-3p | ADAM11 |
| ADIRF-AS1 | hsa-miR-874-3p | HLF |
| ADIRF-AS1 | hsa-miR-874-3p | IGFBP5 |
| ADIRF-AS1 | hsa-miR-874-3p | MCHR1 |
| ADIRF-AS1 | hsa-miR-874-3p | GATA5 |
| ADIRF-AS1 | hsa-miR-874-3p | TEX15 |
| ADIRF-AS1 | hsa-miR-874-3p | CASQ2 |
| ADIRF-AS1 | hsa-miR-874-3p | ASPA |
| ADIRF-AS1 | hsa-miR-874-3p | SLC2A12 |
| ADIRF-AS1 | hsa-miR-874-3p | MYL9 |
| ADIRF-AS1 | hsa-miR-874-3p | KCNE4 |
| ADIRF-AS1 | hsa-miR-874-3p | TCF23 |
| ADIRF-AS1 | hsa-miR-874-3p | HPGD |
| ADIRF-AS1 | hsa-miR-874-3p | TMEM132B |
| ADIRF-AS1 | hsa-miR-874-3p | HSPB3 |
| ADIRF-AS1 | hsa-miR-874-3p | KCNK3 |
| ADIRF-AS1 | hsa-miR-874-3p | GPR37 |
| ADIRF-AS1 | hsa-miR-874-3p | CPLX1 |
| ADIRF-AS1 | hsa-miR-874-3p | TUB |
| ADIRF-AS1 | hsa-miR-874-3p | MSRB3 |
| ADIRF-AS1 | hsa-miR-874-3p | SPEG |
| ADIRF-AS1 | hsa-miR-874-3p | C7 |
| ADIRF-AS1 | hsa-miR-874-3p | PLAG1 |
| ADIRF-AS1 | hsa-miR-874-3p | SLC2A4 |
| ADIRF-AS1 | hsa-miR-874-3p | FIBIN |
| ADIRF-AS1 | hsa-miR-874-3p | C11orf87 |
| ADIRF-AS1 | hsa-miR-874-3p | TNFAIP8L3 |
| ADIRF-AS1 | hsa-miR-874-3p | SYNGR1 |
| ADIRF-AS1 | hsa-miR-874-3p | WSCD2 |
| ADIRF-AS1 | hsa-miR-874-3p | C3orf70 |
| ADIRF-AS1 | hsa-miR-874-3p | TSPAN18 |
| ADIRF-AS1 | hsa-miR-874-3p | SLC24A2 |
| ADIRF-AS1 | hsa-miR-874-3p | CAMK2A |
| ADIRF-AS1 | hsa-miR-874-3p | SMOC2 |
| ADIRF-AS1 | hsa-miR-874-3p | JPH4 |
| ADIRF-AS1 | hsa-miR-874-3p | DCX |
| ADIRF-AS1 | hsa-miR-874-3p | PLN |
| ADIRF-AS1 | hsa-miR-874-3p | NFIX |
| ADIRF-AS1 | hsa-miR-874-3p | MYLK |
| ADIRF-AS1 | hsa-miR-874-3p | GPR161 |
| ADIRF-AS1 | hsa-miR-874-3p | PBX1 |
| ADIRF-AS1 | hsa-miR-874-3p | RBM20 |
| ADIRF-AS1 | hsa-miR-874-3p | SYPL2 |
| ADIRF-AS1 | hsa-miR-874-3p | BEND6 |
| ADIRF-AS1 | hsa-miR-874-3p | KCNB1 |
| ADIRF-AS1 | hsa-miR-874-3p | PAK3 |
| ADIRF-AS1 | hsa-miR-874-3p | SLC6A9 |
| ADIRF-AS1 | hsa-miR-874-3p | JPH2 |
| ADIRF-AS1 | hsa-miR-874-3p | NUDT13 |
| ADIRF-AS1 | hsa-miR-874-3p | RSPO1 |
| ADIRF-AS1 | hsa-miR-874-3p | FABP3 |
| ADIRF-AS1 | hsa-miR-874-3p | DES |
| ADIRF-AS1 | hsa-miR-874-3p | CCDC3 |
| ADIRF-AS1 | hsa-miR-874-3p | PTCHD1 |
| ADIRF-AS1 | hsa-miR-874-3p | ADRA1D |
| ADIRF-AS1 | hsa-miR-874-3p | DCLK1 |
| ADIRF-AS1 | hsa-miR-874-3p | CYS1 |
| ADIRF-AS1 | hsa-miR-874-3p | LGI2 |
| ADIRF-AS1 | hsa-miR-874-3p | NT5DC3 |
| ADIRF-AS1 | hsa-miR-874-3p | PRIMA1 |
| ADIRF-AS1 | hsa-miR-874-3p | CDKL1 |
| ADIRF-AS1 | hsa-miR-874-3p | ST8SIA1 |
| ADIRF-AS1 | hsa-miR-874-3p | RIC3 |
| ADIRF-AS1 | hsa-miR-874-3p | SRL |
| ADIRF-AS1 | hsa-miR-874-3p | NFASC |
| ADIRF-AS1 | hsa-miR-874-3p | CRYBB3 |
| ADIRF-AS1 | hsa-miR-874-3p | MTMR11 |
| ADIRF-AS1 | hsa-miR-874-3p | LIMS2 |
| ADIRF-AS1 | hsa-miR-874-3p | HSPB7 |
| ADIRF-AS1 | hsa-miR-874-3p | MRVI1 |
| ADIRF-AS1 | hsa-miR-874-3p | SEPT4 |
| ADIRF-AS1 | hsa-miR-874-3p | HMCN2 |
| ADIRF-AS1 | hsa-miR-874-3p | SYNPO2 |
| ADIRF-AS1 | hsa-miR-874-3p | GFRA1 |
| ADIRF-AS1 | hsa-miR-874-3p | MRGPRF |
| ADIRF-AS1 | hsa-miR-874-3p | SHISA6 |
| ADIRF-AS1 | hsa-miR-874-3p | FAM107A |
| ADIRF-AS1 | hsa-miR-874-3p | LDB2 |
| ADIRF-AS1 | hsa-miR-874-3p | SORCS2 |
| ADIRF-AS1 | hsa-miR-874-3p | STMN2 |
| ADIRF-AS1 | hsa-miR-874-3p | SLIT3 |
| ADIRF-AS1 | hsa-miR-874-3p | YPEL4 |
| ADIRF-AS1 | hsa-miR-874-3p | PIANP |
| ADIRF-AS1 | hsa-miR-874-3p | FHL5 |
| ADIRF-AS1 | hsa-miR-874-3p | TRERF1 |
| ADIRF-AS1 | hsa-miR-874-3p | LDB3 |
| ADIRF-AS1 | hsa-miR-874-3p | ADCYAP1 |
| ADIRF-AS1 | hsa-miR-874-3p | RBFOX3 |
| ADIRF-AS1 | hsa-miR-874-3p | PPP1R12B |
| MIR1-1HG-AS1 | hsa-miR-874-3p | FSTL3 |
| MIR1-1HG-AS1 | hsa-miR-874-3p | ADAM11 |
| MIR1-1HG-AS1 | hsa-miR-874-3p | HLF |
| MIR1-1HG-AS1 | hsa-miR-874-3p | IGFBP5 |
| MIR1-1HG-AS1 | hsa-miR-874-3p | MCHR1 |
| MIR1-1HG-AS1 | hsa-miR-874-3p | GATA5 |
| MIR1-1HG-AS1 | hsa-miR-874-3p | TEX15 |
| MIR1-1HG-AS1 | hsa-miR-874-3p | CASQ2 |
| MIR1-1HG-AS1 | hsa-miR-874-3p | ASPA |
| MIR1-1HG-AS1 | hsa-miR-874-3p | SLC2A12 |
| MIR1-1HG-AS1 | hsa-miR-874-3p | MYL9 |
| MIR1-1HG-AS1 | hsa-miR-874-3p | KCNE4 |
| MIR1-1HG-AS1 | hsa-miR-874-3p | TCF23 |
| MIR1-1HG-AS1 | hsa-miR-874-3p | HPGD |
| MIR1-1HG-AS1 | hsa-miR-874-3p | TMEM132B |
| MIR1-1HG-AS1 | hsa-miR-874-3p | HSPB3 |
| MIR1-1HG-AS1 | hsa-miR-874-3p | KCNK3 |
| MIR1-1HG-AS1 | hsa-miR-874-3p | GPR37 |
| MIR1-1HG-AS1 | hsa-miR-874-3p | CPLX1 |
| MIR1-1HG-AS1 | hsa-miR-874-3p | TUB |
| MIR1-1HG-AS1 | hsa-miR-874-3p | MSRB3 |
| MIR1-1HG-AS1 | hsa-miR-874-3p | SPEG |
| MIR1-1HG-AS1 | hsa-miR-874-3p | C7 |
| MIR1-1HG-AS1 | hsa-miR-874-3p | PLAG1 |
| MIR1-1HG-AS1 | hsa-miR-874-3p | SLC2A4 |
| MIR1-1HG-AS1 | hsa-miR-874-3p | FIBIN |
| MIR1-1HG-AS1 | hsa-miR-874-3p | C11orf87 |
| MIR1-1HG-AS1 | hsa-miR-874-3p | TNFAIP8L3 |
| MIR1-1HG-AS1 | hsa-miR-874-3p | SYNGR1 |
| MIR1-1HG-AS1 | hsa-miR-874-3p | WSCD2 |
| MIR1-1HG-AS1 | hsa-miR-874-3p | C3orf70 |
| MIR1-1HG-AS1 | hsa-miR-874-3p | TSPAN18 |
| MIR1-1HG-AS1 | hsa-miR-874-3p | SLC24A2 |
| MIR1-1HG-AS1 | hsa-miR-874-3p | CAMK2A |
| MIR1-1HG-AS1 | hsa-miR-874-3p | SMOC2 |
| MIR1-1HG-AS1 | hsa-miR-874-3p | JPH4 |
| MIR1-1HG-AS1 | hsa-miR-874-3p | DCX |
| MIR1-1HG-AS1 | hsa-miR-874-3p | PLN |
| MIR1-1HG-AS1 | hsa-miR-874-3p | NFIX |
| MIR1-1HG-AS1 | hsa-miR-874-3p | MYLK |
| MIR1-1HG-AS1 | hsa-miR-874-3p | GPR161 |
| MIR1-1HG-AS1 | hsa-miR-874-3p | PBX1 |
| MIR1-1HG-AS1 | hsa-miR-874-3p | RBM20 |
| MIR1-1HG-AS1 | hsa-miR-874-3p | SYPL2 |
| MIR1-1HG-AS1 | hsa-miR-874-3p | BEND6 |
| MIR1-1HG-AS1 | hsa-miR-874-3p | KCNB1 |
| MIR1-1HG-AS1 | hsa-miR-874-3p | PAK3 |
| MIR1-1HG-AS1 | hsa-miR-874-3p | SLC6A9 |
| MIR1-1HG-AS1 | hsa-miR-874-3p | JPH2 |
| MIR1-1HG-AS1 | hsa-miR-874-3p | NUDT13 |
| MIR1-1HG-AS1 | hsa-miR-874-3p | RSPO1 |
| MIR1-1HG-AS1 | hsa-miR-874-3p | FABP3 |
| MIR1-1HG-AS1 | hsa-miR-874-3p | DES |
| MIR1-1HG-AS1 | hsa-miR-874-3p | CCDC3 |
| MIR1-1HG-AS1 | hsa-miR-874-3p | PTCHD1 |
| MIR1-1HG-AS1 | hsa-miR-874-3p | ADRA1D |
| MIR1-1HG-AS1 | hsa-miR-874-3p | DCLK1 |
| MIR1-1HG-AS1 | hsa-miR-874-3p | CYS1 |
| MIR1-1HG-AS1 | hsa-miR-874-3p | LGI2 |
| MIR1-1HG-AS1 | hsa-miR-874-3p | NT5DC3 |
| MIR1-1HG-AS1 | hsa-miR-874-3p | PRIMA1 |
| MIR1-1HG-AS1 | hsa-miR-874-3p | CDKL1 |
| MIR1-1HG-AS1 | hsa-miR-874-3p | ST8SIA1 |
| MIR1-1HG-AS1 | hsa-miR-874-3p | RIC3 |
| MIR1-1HG-AS1 | hsa-miR-874-3p | SRL |
| MIR1-1HG-AS1 | hsa-miR-874-3p | NFASC |
| MIR1-1HG-AS1 | hsa-miR-874-3p | CRYBB3 |
| MIR1-1HG-AS1 | hsa-miR-874-3p | MTMR11 |
| MIR1-1HG-AS1 | hsa-miR-874-3p | LIMS2 |
| MIR1-1HG-AS1 | hsa-miR-874-3p | HSPB7 |
| MIR1-1HG-AS1 | hsa-miR-874-3p | MRVI1 |
| MIR1-1HG-AS1 | hsa-miR-874-3p | SEPT4 |
| MIR1-1HG-AS1 | hsa-miR-874-3p | HMCN2 |
| MIR1-1HG-AS1 | hsa-miR-874-3p | SYNPO2 |
| MIR1-1HG-AS1 | hsa-miR-874-3p | GFRA1 |
| MIR1-1HG-AS1 | hsa-miR-874-3p | MRGPRF |
| MIR1-1HG-AS1 | hsa-miR-874-3p | SHISA6 |
| MIR1-1HG-AS1 | hsa-miR-874-3p | FAM107A |
| MIR1-1HG-AS1 | hsa-miR-874-3p | LDB2 |
| MIR1-1HG-AS1 | hsa-miR-874-3p | SORCS2 |
| MIR1-1HG-AS1 | hsa-miR-874-3p | STMN2 |
| MIR1-1HG-AS1 | hsa-miR-874-3p | SLIT3 |
| MIR1-1HG-AS1 | hsa-miR-874-3p | YPEL4 |
| MIR1-1HG-AS1 | hsa-miR-874-3p | PIANP |
| MIR1-1HG-AS1 | hsa-miR-874-3p | FHL5 |
| MIR1-1HG-AS1 | hsa-miR-874-3p | TRERF1 |
| MIR1-1HG-AS1 | hsa-miR-874-3p | LDB3 |
| MIR1-1HG-AS1 | hsa-miR-874-3p | ADCYAP1 |
| MIR1-1HG-AS1 | hsa-miR-874-3p | RBFOX3 |
| MIR1-1HG-AS1 | hsa-miR-874-3p | PPP1R12B |
| AC100803.2 | hsa-miR-874-3p | FSTL3 |
| AC100803.2 | hsa-miR-874-3p | ADAM11 |
| AC100803.2 | hsa-miR-874-3p | HLF |
| AC100803.2 | hsa-miR-874-3p | IGFBP5 |
| AC100803.2 | hsa-miR-874-3p | MCHR1 |
| AC100803.2 | hsa-miR-874-3p | GATA5 |
| AC100803.2 | hsa-miR-874-3p | TEX15 |
| AC100803.2 | hsa-miR-874-3p | CASQ2 |
| AC100803.2 | hsa-miR-874-3p | ASPA |
| AC100803.2 | hsa-miR-874-3p | SLC2A12 |
| AC100803.2 | hsa-miR-874-3p | MYL9 |
| AC100803.2 | hsa-miR-874-3p | KCNE4 |
| AC100803.2 | hsa-miR-874-3p | TCF23 |
| AC100803.2 | hsa-miR-874-3p | HPGD |
| AC100803.2 | hsa-miR-874-3p | TMEM132B |
| AC100803.2 | hsa-miR-874-3p | HSPB3 |
| AC100803.2 | hsa-miR-874-3p | KCNK3 |
| AC100803.2 | hsa-miR-874-3p | GPR37 |
| AC100803.2 | hsa-miR-874-3p | CPLX1 |
| AC100803.2 | hsa-miR-874-3p | TUB |
| AC100803.2 | hsa-miR-874-3p | MSRB3 |
| AC100803.2 | hsa-miR-874-3p | SPEG |
| AC100803.2 | hsa-miR-874-3p | C7 |
| AC100803.2 | hsa-miR-874-3p | PLAG1 |
| AC100803.2 | hsa-miR-874-3p | SLC2A4 |
| AC100803.2 | hsa-miR-874-3p | FIBIN |
| AC100803.2 | hsa-miR-874-3p | C11orf87 |
| AC100803.2 | hsa-miR-874-3p | TNFAIP8L3 |
| AC100803.2 | hsa-miR-874-3p | SYNGR1 |
| AC100803.2 | hsa-miR-874-3p | WSCD2 |
| AC100803.2 | hsa-miR-874-3p | C3orf70 |
| AC100803.2 | hsa-miR-874-3p | TSPAN18 |
| AC100803.2 | hsa-miR-874-3p | SLC24A2 |
| AC100803.2 | hsa-miR-874-3p | CAMK2A |
| AC100803.2 | hsa-miR-874-3p | SMOC2 |
| AC100803.2 | hsa-miR-874-3p | JPH4 |
| AC100803.2 | hsa-miR-874-3p | DCX |
| AC100803.2 | hsa-miR-874-3p | PLN |
| AC100803.2 | hsa-miR-874-3p | NFIX |
| AC100803.2 | hsa-miR-874-3p | MYLK |
| AC100803.2 | hsa-miR-874-3p | GPR161 |
| AC100803.2 | hsa-miR-874-3p | PBX1 |
| AC100803.2 | hsa-miR-874-3p | RBM20 |
| AC100803.2 | hsa-miR-874-3p | SYPL2 |
| AC100803.2 | hsa-miR-874-3p | BEND6 |
| AC100803.2 | hsa-miR-874-3p | KCNB1 |
| AC100803.2 | hsa-miR-874-3p | PAK3 |
| AC100803.2 | hsa-miR-874-3p | SLC6A9 |
| AC100803.2 | hsa-miR-874-3p | JPH2 |
| AC100803.2 | hsa-miR-874-3p | NUDT13 |
| AC100803.2 | hsa-miR-874-3p | RSPO1 |
| AC100803.2 | hsa-miR-874-3p | FABP3 |
| AC100803.2 | hsa-miR-874-3p | DES |
| AC100803.2 | hsa-miR-874-3p | CCDC3 |
| AC100803.2 | hsa-miR-874-3p | PTCHD1 |
| AC100803.2 | hsa-miR-874-3p | ADRA1D |
| AC100803.2 | hsa-miR-874-3p | DCLK1 |
| AC100803.2 | hsa-miR-874-3p | CYS1 |
| AC100803.2 | hsa-miR-874-3p | LGI2 |
| AC100803.2 | hsa-miR-874-3p | NT5DC3 |
| AC100803.2 | hsa-miR-874-3p | PRIMA1 |
| AC100803.2 | hsa-miR-874-3p | CDKL1 |
| AC100803.2 | hsa-miR-874-3p | ST8SIA1 |
| AC100803.2 | hsa-miR-874-3p | RIC3 |
| AC100803.2 | hsa-miR-874-3p | SRL |
| AC100803.2 | hsa-miR-874-3p | NFASC |
| AC100803.2 | hsa-miR-874-3p | CRYBB3 |
| AC100803.2 | hsa-miR-874-3p | MTMR11 |
| AC100803.2 | hsa-miR-874-3p | LIMS2 |
| AC100803.2 | hsa-miR-874-3p | HSPB7 |
| AC100803.2 | hsa-miR-874-3p | MRVI1 |
| AC100803.2 | hsa-miR-874-3p | SEPT4 |
| AC100803.2 | hsa-miR-874-3p | HMCN2 |
| AC100803.2 | hsa-miR-874-3p | SYNPO2 |
| AC100803.2 | hsa-miR-874-3p | GFRA1 |
| AC100803.2 | hsa-miR-874-3p | MRGPRF |
| AC100803.2 | hsa-miR-874-3p | SHISA6 |
| AC100803.2 | hsa-miR-874-3p | FAM107A |
| AC100803.2 | hsa-miR-874-3p | LDB2 |
| AC100803.2 | hsa-miR-874-3p | SORCS2 |
| AC100803.2 | hsa-miR-874-3p | STMN2 |
| AC100803.2 | hsa-miR-874-3p | SLIT3 |
| AC100803.2 | hsa-miR-874-3p | YPEL4 |
| AC100803.2 | hsa-miR-874-3p | PIANP |
| AC100803.2 | hsa-miR-874-3p | FHL5 |
| AC100803.2 | hsa-miR-874-3p | TRERF1 |
| AC100803.2 | hsa-miR-874-3p | LDB3 |
| AC100803.2 | hsa-miR-874-3p | ADCYAP1 |
| AC100803.2 | hsa-miR-874-3p | RBFOX3 |
| AC100803.2 | hsa-miR-874-3p | PPP1R12B |
| MEF2C-AS1 | hsa-miR-874-3p | FSTL3 |
| MEF2C-AS1 | hsa-miR-874-3p | ADAM11 |
| MEF2C-AS1 | hsa-miR-874-3p | HLF |
| MEF2C-AS1 | hsa-miR-874-3p | IGFBP5 |
| MEF2C-AS1 | hsa-miR-874-3p | MCHR1 |
| MEF2C-AS1 | hsa-miR-874-3p | GATA5 |
| MEF2C-AS1 | hsa-miR-874-3p | TEX15 |
| MEF2C-AS1 | hsa-miR-874-3p | CASQ2 |
| MEF2C-AS1 | hsa-miR-874-3p | ASPA |
| MEF2C-AS1 | hsa-miR-874-3p | SLC2A12 |
| MEF2C-AS1 | hsa-miR-874-3p | MYL9 |
| MEF2C-AS1 | hsa-miR-874-3p | KCNE4 |
| MEF2C-AS1 | hsa-miR-874-3p | TCF23 |
| MEF2C-AS1 | hsa-miR-874-3p | HPGD |
| MEF2C-AS1 | hsa-miR-874-3p | TMEM132B |
| MEF2C-AS1 | hsa-miR-874-3p | HSPB3 |
| MEF2C-AS1 | hsa-miR-874-3p | KCNK3 |
| MEF2C-AS1 | hsa-miR-874-3p | GPR37 |
| MEF2C-AS1 | hsa-miR-874-3p | CPLX1 |
| MEF2C-AS1 | hsa-miR-874-3p | TUB |
| MEF2C-AS1 | hsa-miR-874-3p | MSRB3 |
| MEF2C-AS1 | hsa-miR-874-3p | SPEG |
| MEF2C-AS1 | hsa-miR-874-3p | C7 |
| MEF2C-AS1 | hsa-miR-874-3p | PLAG1 |
| MEF2C-AS1 | hsa-miR-874-3p | SLC2A4 |
| MEF2C-AS1 | hsa-miR-874-3p | FIBIN |
| MEF2C-AS1 | hsa-miR-874-3p | C11orf87 |
| MEF2C-AS1 | hsa-miR-874-3p | TNFAIP8L3 |
| MEF2C-AS1 | hsa-miR-874-3p | SYNGR1 |
| MEF2C-AS1 | hsa-miR-874-3p | WSCD2 |
| MEF2C-AS1 | hsa-miR-874-3p | C3orf70 |
| MEF2C-AS1 | hsa-miR-874-3p | TSPAN18 |
| MEF2C-AS1 | hsa-miR-874-3p | SLC24A2 |
| MEF2C-AS1 | hsa-miR-874-3p | CAMK2A |
| MEF2C-AS1 | hsa-miR-874-3p | SMOC2 |
| MEF2C-AS1 | hsa-miR-874-3p | JPH4 |
| MEF2C-AS1 | hsa-miR-874-3p | DCX |
| MEF2C-AS1 | hsa-miR-874-3p | PLN |
| MEF2C-AS1 | hsa-miR-874-3p | NFIX |
| MEF2C-AS1 | hsa-miR-874-3p | MYLK |
| MEF2C-AS1 | hsa-miR-874-3p | GPR161 |
| MEF2C-AS1 | hsa-miR-874-3p | PBX1 |
| MEF2C-AS1 | hsa-miR-874-3p | RBM20 |
| MEF2C-AS1 | hsa-miR-874-3p | SYPL2 |
| MEF2C-AS1 | hsa-miR-874-3p | BEND6 |
| MEF2C-AS1 | hsa-miR-874-3p | KCNB1 |
| MEF2C-AS1 | hsa-miR-874-3p | PAK3 |
| MEF2C-AS1 | hsa-miR-874-3p | SLC6A9 |
| MEF2C-AS1 | hsa-miR-874-3p | JPH2 |
| MEF2C-AS1 | hsa-miR-874-3p | NUDT13 |
| MEF2C-AS1 | hsa-miR-874-3p | RSPO1 |
| MEF2C-AS1 | hsa-miR-874-3p | FABP3 |
| MEF2C-AS1 | hsa-miR-874-3p | DES |
| MEF2C-AS1 | hsa-miR-874-3p | CCDC3 |
| MEF2C-AS1 | hsa-miR-874-3p | PTCHD1 |
| MEF2C-AS1 | hsa-miR-874-3p | ADRA1D |
| MEF2C-AS1 | hsa-miR-874-3p | DCLK1 |
| MEF2C-AS1 | hsa-miR-874-3p | CYS1 |
| MEF2C-AS1 | hsa-miR-874-3p | LGI2 |
| MEF2C-AS1 | hsa-miR-874-3p | NT5DC3 |
| MEF2C-AS1 | hsa-miR-874-3p | PRIMA1 |
| MEF2C-AS1 | hsa-miR-874-3p | CDKL1 |
| MEF2C-AS1 | hsa-miR-874-3p | ST8SIA1 |
| MEF2C-AS1 | hsa-miR-874-3p | RIC3 |
| MEF2C-AS1 | hsa-miR-874-3p | SRL |
| MEF2C-AS1 | hsa-miR-874-3p | NFASC |
| MEF2C-AS1 | hsa-miR-874-3p | CRYBB3 |
| MEF2C-AS1 | hsa-miR-874-3p | MTMR11 |
| MEF2C-AS1 | hsa-miR-874-3p | LIMS2 |
| MEF2C-AS1 | hsa-miR-874-3p | HSPB7 |
| MEF2C-AS1 | hsa-miR-874-3p | MRVI1 |
| MEF2C-AS1 | hsa-miR-874-3p | SEPT4 |
| MEF2C-AS1 | hsa-miR-874-3p | HMCN2 |
| MEF2C-AS1 | hsa-miR-874-3p | SYNPO2 |
| MEF2C-AS1 | hsa-miR-874-3p | GFRA1 |
| MEF2C-AS1 | hsa-miR-874-3p | MRGPRF |
| MEF2C-AS1 | hsa-miR-874-3p | SHISA6 |
| MEF2C-AS1 | hsa-miR-874-3p | FAM107A |
| MEF2C-AS1 | hsa-miR-874-3p | LDB2 |
| MEF2C-AS1 | hsa-miR-874-3p | SORCS2 |
| MEF2C-AS1 | hsa-miR-874-3p | STMN2 |
| MEF2C-AS1 | hsa-miR-874-3p | SLIT3 |
| MEF2C-AS1 | hsa-miR-874-3p | YPEL4 |
| MEF2C-AS1 | hsa-miR-874-3p | PIANP |
| MEF2C-AS1 | hsa-miR-874-3p | FHL5 |
| MEF2C-AS1 | hsa-miR-874-3p | TRERF1 |
| MEF2C-AS1 | hsa-miR-874-3p | LDB3 |
| MEF2C-AS1 | hsa-miR-874-3p | ADCYAP1 |
| MEF2C-AS1 | hsa-miR-874-3p | RBFOX3 |
| MEF2C-AS1 | hsa-miR-874-3p | PPP1R12B |
| AP001107.8 | hsa-miR-874-3p | FSTL3 |
| AP001107.8 | hsa-miR-874-3p | ADAM11 |
| AP001107.8 | hsa-miR-874-3p | HLF |
| AP001107.8 | hsa-miR-874-3p | IGFBP5 |
| AP001107.8 | hsa-miR-874-3p | MCHR1 |
| AP001107.8 | hsa-miR-874-3p | GATA5 |
| AP001107.8 | hsa-miR-874-3p | TEX15 |
| AP001107.8 | hsa-miR-874-3p | CASQ2 |
| AP001107.8 | hsa-miR-874-3p | ASPA |
| AP001107.8 | hsa-miR-874-3p | SLC2A12 |
| AP001107.8 | hsa-miR-874-3p | MYL9 |
| AP001107.8 | hsa-miR-874-3p | KCNE4 |
| AP001107.8 | hsa-miR-874-3p | TCF23 |
| AP001107.8 | hsa-miR-874-3p | HPGD |
| AP001107.8 | hsa-miR-874-3p | TMEM132B |
| AP001107.8 | hsa-miR-874-3p | HSPB3 |
| AP001107.8 | hsa-miR-874-3p | KCNK3 |
| AP001107.8 | hsa-miR-874-3p | GPR37 |
| AP001107.8 | hsa-miR-874-3p | CPLX1 |
| AP001107.8 | hsa-miR-874-3p | TUB |
| AP001107.8 | hsa-miR-874-3p | MSRB3 |
| AP001107.8 | hsa-miR-874-3p | SPEG |
| AP001107.8 | hsa-miR-874-3p | C7 |
| AP001107.8 | hsa-miR-874-3p | PLAG1 |
| AP001107.8 | hsa-miR-874-3p | SLC2A4 |
| AP001107.8 | hsa-miR-874-3p | FIBIN |
| AP001107.8 | hsa-miR-874-3p | C11orf87 |
| AP001107.8 | hsa-miR-874-3p | TNFAIP8L3 |
| AP001107.8 | hsa-miR-874-3p | SYNGR1 |
| AP001107.8 | hsa-miR-874-3p | WSCD2 |
| AP001107.8 | hsa-miR-874-3p | C3orf70 |
| AP001107.8 | hsa-miR-874-3p | TSPAN18 |
| AP001107.8 | hsa-miR-874-3p | SLC24A2 |
| AP001107.8 | hsa-miR-874-3p | CAMK2A |
| AP001107.8 | hsa-miR-874-3p | SMOC2 |
| AP001107.8 | hsa-miR-874-3p | JPH4 |
| AP001107.8 | hsa-miR-874-3p | DCX |
| AP001107.8 | hsa-miR-874-3p | PLN |
| AP001107.8 | hsa-miR-874-3p | NFIX |
| AP001107.8 | hsa-miR-874-3p | MYLK |
| AP001107.8 | hsa-miR-874-3p | GPR161 |
| AP001107.8 | hsa-miR-874-3p | PBX1 |
| AP001107.8 | hsa-miR-874-3p | RBM20 |
| AP001107.8 | hsa-miR-874-3p | SYPL2 |
| AP001107.8 | hsa-miR-874-3p | BEND6 |
| AP001107.8 | hsa-miR-874-3p | KCNB1 |
| AP001107.8 | hsa-miR-874-3p | PAK3 |
| AP001107.8 | hsa-miR-874-3p | SLC6A9 |
| AP001107.8 | hsa-miR-874-3p | JPH2 |
| AP001107.8 | hsa-miR-874-3p | NUDT13 |
| AP001107.8 | hsa-miR-874-3p | RSPO1 |
| AP001107.8 | hsa-miR-874-3p | FABP3 |
| AP001107.8 | hsa-miR-874-3p | DES |
| AP001107.8 | hsa-miR-874-3p | CCDC3 |
| AP001107.8 | hsa-miR-874-3p | PTCHD1 |
| AP001107.8 | hsa-miR-874-3p | ADRA1D |
| AP001107.8 | hsa-miR-874-3p | DCLK1 |
| AP001107.8 | hsa-miR-874-3p | CYS1 |
| AP001107.8 | hsa-miR-874-3p | LGI2 |
| AP001107.8 | hsa-miR-874-3p | NT5DC3 |
| AP001107.8 | hsa-miR-874-3p | PRIMA1 |
| AP001107.8 | hsa-miR-874-3p | CDKL1 |
| AP001107.8 | hsa-miR-874-3p | ST8SIA1 |
| AP001107.8 | hsa-miR-874-3p | RIC3 |
| AP001107.8 | hsa-miR-874-3p | SRL |
| AP001107.8 | hsa-miR-874-3p | NFASC |
| AP001107.8 | hsa-miR-874-3p | CRYBB3 |
| AP001107.8 | hsa-miR-874-3p | MTMR11 |
| AP001107.8 | hsa-miR-874-3p | LIMS2 |
| AP001107.8 | hsa-miR-874-3p | HSPB7 |
| AP001107.8 | hsa-miR-874-3p | MRVI1 |
| AP001107.8 | hsa-miR-874-3p | SEPT4 |
| AP001107.8 | hsa-miR-874-3p | HMCN2 |
| AP001107.8 | hsa-miR-874-3p | SYNPO2 |
| AP001107.8 | hsa-miR-874-3p | GFRA1 |
| AP001107.8 | hsa-miR-874-3p | MRGPRF |
| AP001107.8 | hsa-miR-874-3p | SHISA6 |
| AP001107.8 | hsa-miR-874-3p | FAM107A |
| AP001107.8 | hsa-miR-874-3p | LDB2 |
| AP001107.8 | hsa-miR-874-3p | SORCS2 |
| AP001107.8 | hsa-miR-874-3p | STMN2 |
| AP001107.8 | hsa-miR-874-3p | SLIT3 |
| AP001107.8 | hsa-miR-874-3p | YPEL4 |
| AP001107.8 | hsa-miR-874-3p | PIANP |
| AP001107.8 | hsa-miR-874-3p | FHL5 |
| AP001107.8 | hsa-miR-874-3p | TRERF1 |
| AP001107.8 | hsa-miR-874-3p | LDB3 |
| AP001107.8 | hsa-miR-874-3p | ADCYAP1 |
| AP001107.8 | hsa-miR-874-3p | RBFOX3 |
| AP001107.8 | hsa-miR-874-3p | PPP1R12B |
| LINC00632 | hsa-miR-532-3p | RERGL |
| LINC00632 | hsa-miR-532-3p | RAB9B |
| LINC00632 | hsa-miR-532-3p | PTGIS |
| LINC00632 | hsa-miR-532-3p | CNN1 |
| LINC00632 | hsa-miR-532-3p | POPDC3 |
| LINC00632 | hsa-miR-532-3p | PI15 |
| LINC00632 | hsa-miR-532-3p | ABCC9 |
| LINC00632 | hsa-miR-532-3p | ASPA |
| LINC00632 | hsa-miR-532-3p | NGEF |
| LINC00632 | hsa-miR-532-3p | MYL9 |
| LINC00632 | hsa-miR-532-3p | KCNE4 |
| LINC00632 | hsa-miR-532-3p | PDLIM3 |
| LINC00632 | hsa-miR-532-3p | UNC5D |
| LINC00632 | hsa-miR-532-3p | FBXO27 |
| LINC00632 | hsa-miR-532-3p | KCNIP3 |
| LINC00632 | hsa-miR-532-3p | TCF23 |
| LINC00632 | hsa-miR-532-3p | MASP1 |
| LINC00632 | hsa-miR-532-3p | HPGD |
| LINC00632 | hsa-miR-532-3p | PRRT2 |
| LINC00632 | hsa-miR-532-3p | KCNAB1 |
| LINC00632 | hsa-miR-532-3p | KCNK3 |
| LINC00632 | hsa-miR-532-3p | SLC38A11 |
| LINC00632 | hsa-miR-532-3p | ADH1B |
| LINC00632 | hsa-miR-532-3p | TUB |
| LINC00632 | hsa-miR-532-3p | MSRB3 |
| LINC00632 | hsa-miR-532-3p | SPEG |
| LINC00632 | hsa-miR-532-3p | LPP |
| LINC00632 | hsa-miR-532-3p | C7 |
| LINC00632 | hsa-miR-532-3p | EBF1 |
| LINC00632 | hsa-miR-532-3p | BVES |
| LINC00632 | hsa-miR-532-3p | CLU |
| LINC00632 | hsa-miR-532-3p | PLAG1 |
| LINC00632 | hsa-miR-532-3p | SLC2A4 |
| LINC00632 | hsa-miR-532-3p | FIBIN |
| LINC00632 | hsa-miR-532-3p | DUOX1 |
| LINC00632 | hsa-miR-532-3p | CAPN6 |
| LINC00632 | hsa-miR-532-3p | DMRTA1 |
| LINC00632 | hsa-miR-532-3p | C3orf80 |
| LINC00632 | hsa-miR-532-3p | GDNF |
| LINC00632 | hsa-miR-532-3p | TNFAIP8L3 |
| LINC00632 | hsa-miR-532-3p | SYNGR1 |
| LINC00632 | hsa-miR-532-3p | WSCD2 |
| LINC00632 | hsa-miR-532-3p | C3orf70 |
| LINC00632 | hsa-miR-532-3p | TSPAN18 |
| LINC00632 | hsa-miR-532-3p | PRELP |
| LINC00632 | hsa-miR-532-3p | THNSL2 |
| LINC00632 | hsa-miR-532-3p | NR3C2 |
| LINC00632 | hsa-miR-532-3p | SMOC2 |
| LINC00632 | hsa-miR-532-3p | JPH4 |
| LINC00632 | hsa-miR-532-3p | DCX |
| LINC00632 | hsa-miR-532-3p | ANKRD36 |
| LINC00632 | hsa-miR-532-3p | CACNA1H |
| LINC00632 | hsa-miR-532-3p | NFIX |
| LINC00632 | hsa-miR-532-3p | CNR1 |
| LINC00632 | hsa-miR-532-3p | LMOD1 |
| LINC00632 | hsa-miR-532-3p | GPR161 |
| LINC00632 | hsa-miR-532-3p | PBX1 |
| LINC00632 | hsa-miR-532-3p | RBM20 |
| LINC00632 | hsa-miR-532-3p | CDR1 |
| LINC00632 | hsa-miR-532-3p | KCNB1 |
| LINC00632 | hsa-miR-532-3p | PAK3 |
| LINC00632 | hsa-miR-532-3p | CHRDL1 |
| LINC00632 | hsa-miR-532-3p | SLC6A9 |
| LINC00632 | hsa-miR-532-3p | HYI |
| LINC00632 | hsa-miR-532-3p | FOXO6 |
| LINC00632 | hsa-miR-532-3p | JPH2 |
| LINC00632 | hsa-miR-532-3p | RSPO1 |
| LINC00632 | hsa-miR-532-3p | FABP3 |
| LINC00632 | hsa-miR-532-3p | DES |
| LINC00632 | hsa-miR-532-3p | TRPM3 |
| LINC00632 | hsa-miR-532-3p | MAMDC2 |
| LINC00632 | hsa-miR-532-3p | PKHD1L1 |
| LINC00632 | hsa-miR-532-3p | DMD |
| LINC00632 | hsa-miR-532-3p | PTCHD1 |
| LINC00632 | hsa-miR-532-3p | BNC2 |
| LINC00632 | hsa-miR-532-3p | SMOC1 |
| LINC00632 | hsa-miR-532-3p | CYS1 |
| LINC00632 | hsa-miR-532-3p | SEMA5A |
| LINC00632 | hsa-miR-532-3p | RYR3 |
| LINC00632 | hsa-miR-532-3p | CDON |
| LINC00632 | hsa-miR-532-3p | SPOCK1 |
| LINC00632 | hsa-miR-532-3p | FAM13A |
| LINC00632 | hsa-miR-532-3p | ST8SIA1 |
| LINC00632 | hsa-miR-532-3p | ZNF208 |
| LINC00632 | hsa-miR-532-3p | NFASC |
| LINC00632 | hsa-miR-532-3p | DGKB |
| LINC00632 | hsa-miR-532-3p | LIMS2 |
| LINC00632 | hsa-miR-532-3p | HSPB7 |
| LINC00632 | hsa-miR-532-3p | MRVI1 |
| LINC00632 | hsa-miR-532-3p | ITGA11 |
| LINC00632 | hsa-miR-532-3p | HMCN2 |
| LINC00632 | hsa-miR-532-3p | SYNPO2 |
| LINC00632 | hsa-miR-532-3p | GFRA1 |
| LINC00632 | hsa-miR-532-3p | MRGPRF |
| LINC00632 | hsa-miR-532-3p | TCEA3 |
| LINC00632 | hsa-miR-532-3p | FAM107A |
| LINC00632 | hsa-miR-532-3p | KLF8 |
| LINC00632 | hsa-miR-532-3p | FSBP |
| LINC00632 | hsa-miR-532-3p | SLC15A2 |
| LINC00632 | hsa-miR-532-3p | SORCS2 |
| LINC00632 | hsa-miR-532-3p | TMEM200B |
| LINC00632 | hsa-miR-532-3p | YPEL4 |
| LINC00632 | hsa-miR-532-3p | NAALAD2 |
| LINC00632 | hsa-miR-532-3p | PIANP |
| LINC00632 | hsa-miR-532-3p | LDB3 |
| LINC00632 | hsa-miR-532-3p | DUOXA1 |
| LINC00632 | hsa-miR-532-3p | DET1 |
| LINC00632 | hsa-miR-532-3p | RBFOX3 |
| LINC00632 | hsa-miR-532-3p | CCDC68 |
| LINC00632 | hsa-miR-532-3p | PPP1R12B |
| ADIRF-AS1 | hsa-miR-532-3p | RERGL |
| ADIRF-AS1 | hsa-miR-532-3p | RAB9B |
| ADIRF-AS1 | hsa-miR-532-3p | PTGIS |
| ADIRF-AS1 | hsa-miR-532-3p | CNN1 |
| ADIRF-AS1 | hsa-miR-532-3p | POPDC3 |
| ADIRF-AS1 | hsa-miR-532-3p | PI15 |
| ADIRF-AS1 | hsa-miR-532-3p | ABCC9 |
| ADIRF-AS1 | hsa-miR-532-3p | ASPA |
| ADIRF-AS1 | hsa-miR-532-3p | NGEF |
| ADIRF-AS1 | hsa-miR-532-3p | MYL9 |
| ADIRF-AS1 | hsa-miR-532-3p | KCNE4 |
| ADIRF-AS1 | hsa-miR-532-3p | PDLIM3 |
| ADIRF-AS1 | hsa-miR-532-3p | UNC5D |
| ADIRF-AS1 | hsa-miR-532-3p | FBXO27 |
| ADIRF-AS1 | hsa-miR-532-3p | KCNIP3 |
| ADIRF-AS1 | hsa-miR-532-3p | TCF23 |
| ADIRF-AS1 | hsa-miR-532-3p | MASP1 |
| ADIRF-AS1 | hsa-miR-532-3p | HPGD |
| ADIRF-AS1 | hsa-miR-532-3p | PRRT2 |
| ADIRF-AS1 | hsa-miR-532-3p | KCNAB1 |
| ADIRF-AS1 | hsa-miR-532-3p | KCNK3 |
| ADIRF-AS1 | hsa-miR-532-3p | SLC38A11 |
| ADIRF-AS1 | hsa-miR-532-3p | ADH1B |
| ADIRF-AS1 | hsa-miR-532-3p | TUB |
| ADIRF-AS1 | hsa-miR-532-3p | MSRB3 |
| ADIRF-AS1 | hsa-miR-532-3p | SPEG |
| ADIRF-AS1 | hsa-miR-532-3p | LPP |
| ADIRF-AS1 | hsa-miR-532-3p | C7 |
| ADIRF-AS1 | hsa-miR-532-3p | EBF1 |
| ADIRF-AS1 | hsa-miR-532-3p | BVES |
| ADIRF-AS1 | hsa-miR-532-3p | CLU |
| ADIRF-AS1 | hsa-miR-532-3p | PLAG1 |
| ADIRF-AS1 | hsa-miR-532-3p | SLC2A4 |
| ADIRF-AS1 | hsa-miR-532-3p | FIBIN |
| ADIRF-AS1 | hsa-miR-532-3p | DUOX1 |
| ADIRF-AS1 | hsa-miR-532-3p | CAPN6 |
| ADIRF-AS1 | hsa-miR-532-3p | DMRTA1 |
| ADIRF-AS1 | hsa-miR-532-3p | C3orf80 |
| ADIRF-AS1 | hsa-miR-532-3p | GDNF |
| ADIRF-AS1 | hsa-miR-532-3p | TNFAIP8L3 |
| ADIRF-AS1 | hsa-miR-532-3p | SYNGR1 |
| ADIRF-AS1 | hsa-miR-532-3p | WSCD2 |
| ADIRF-AS1 | hsa-miR-532-3p | C3orf70 |
| ADIRF-AS1 | hsa-miR-532-3p | TSPAN18 |
| ADIRF-AS1 | hsa-miR-532-3p | PRELP |
| ADIRF-AS1 | hsa-miR-532-3p | THNSL2 |
| ADIRF-AS1 | hsa-miR-532-3p | NR3C2 |
| ADIRF-AS1 | hsa-miR-532-3p | SMOC2 |
| ADIRF-AS1 | hsa-miR-532-3p | JPH4 |
| ADIRF-AS1 | hsa-miR-532-3p | DCX |
| ADIRF-AS1 | hsa-miR-532-3p | ANKRD36 |
| ADIRF-AS1 | hsa-miR-532-3p | CACNA1H |
| ADIRF-AS1 | hsa-miR-532-3p | NFIX |
| ADIRF-AS1 | hsa-miR-532-3p | CNR1 |
| ADIRF-AS1 | hsa-miR-532-3p | LMOD1 |
| ADIRF-AS1 | hsa-miR-532-3p | GPR161 |
| ADIRF-AS1 | hsa-miR-532-3p | PBX1 |
| ADIRF-AS1 | hsa-miR-532-3p | RBM20 |
| ADIRF-AS1 | hsa-miR-532-3p | CDR1 |
| ADIRF-AS1 | hsa-miR-532-3p | KCNB1 |
| ADIRF-AS1 | hsa-miR-532-3p | PAK3 |
| ADIRF-AS1 | hsa-miR-532-3p | CHRDL1 |
| ADIRF-AS1 | hsa-miR-532-3p | SLC6A9 |
| ADIRF-AS1 | hsa-miR-532-3p | HYI |
| ADIRF-AS1 | hsa-miR-532-3p | FOXO6 |
| ADIRF-AS1 | hsa-miR-532-3p | JPH2 |
| ADIRF-AS1 | hsa-miR-532-3p | RSPO1 |
| ADIRF-AS1 | hsa-miR-532-3p | FABP3 |
| ADIRF-AS1 | hsa-miR-532-3p | DES |
| ADIRF-AS1 | hsa-miR-532-3p | TRPM3 |
| ADIRF-AS1 | hsa-miR-532-3p | MAMDC2 |
| ADIRF-AS1 | hsa-miR-532-3p | PKHD1L1 |
| ADIRF-AS1 | hsa-miR-532-3p | DMD |
| ADIRF-AS1 | hsa-miR-532-3p | PTCHD1 |
| ADIRF-AS1 | hsa-miR-532-3p | BNC2 |
| ADIRF-AS1 | hsa-miR-532-3p | SMOC1 |
| ADIRF-AS1 | hsa-miR-532-3p | CYS1 |
| ADIRF-AS1 | hsa-miR-532-3p | SEMA5A |
| ADIRF-AS1 | hsa-miR-532-3p | RYR3 |
| ADIRF-AS1 | hsa-miR-532-3p | CDON |
| ADIRF-AS1 | hsa-miR-532-3p | SPOCK1 |
| ADIRF-AS1 | hsa-miR-532-3p | FAM13A |
| ADIRF-AS1 | hsa-miR-532-3p | ST8SIA1 |
| ADIRF-AS1 | hsa-miR-532-3p | ZNF208 |
| ADIRF-AS1 | hsa-miR-532-3p | NFASC |
| ADIRF-AS1 | hsa-miR-532-3p | DGKB |
| ADIRF-AS1 | hsa-miR-532-3p | LIMS2 |
| ADIRF-AS1 | hsa-miR-532-3p | HSPB7 |
| ADIRF-AS1 | hsa-miR-532-3p | MRVI1 |
| ADIRF-AS1 | hsa-miR-532-3p | ITGA11 |
| ADIRF-AS1 | hsa-miR-532-3p | HMCN2 |
| ADIRF-AS1 | hsa-miR-532-3p | SYNPO2 |
| ADIRF-AS1 | hsa-miR-532-3p | GFRA1 |
| ADIRF-AS1 | hsa-miR-532-3p | MRGPRF |
| ADIRF-AS1 | hsa-miR-532-3p | TCEA3 |
| ADIRF-AS1 | hsa-miR-532-3p | FAM107A |
| ADIRF-AS1 | hsa-miR-532-3p | KLF8 |
| ADIRF-AS1 | hsa-miR-532-3p | FSBP |
| ADIRF-AS1 | hsa-miR-532-3p | SLC15A2 |
| ADIRF-AS1 | hsa-miR-532-3p | SORCS2 |
| ADIRF-AS1 | hsa-miR-532-3p | TMEM200B |
| ADIRF-AS1 | hsa-miR-532-3p | YPEL4 |
| ADIRF-AS1 | hsa-miR-532-3p | NAALAD2 |
| ADIRF-AS1 | hsa-miR-532-3p | PIANP |
| ADIRF-AS1 | hsa-miR-532-3p | LDB3 |
| ADIRF-AS1 | hsa-miR-532-3p | DUOXA1 |
| ADIRF-AS1 | hsa-miR-532-3p | DET1 |
| ADIRF-AS1 | hsa-miR-532-3p | RBFOX3 |
| ADIRF-AS1 | hsa-miR-532-3p | CCDC68 |
| ADIRF-AS1 | hsa-miR-532-3p | PPP1R12B |
| AC053503.6 | hsa-miR-532-3p | RERGL |
| AC053503.6 | hsa-miR-532-3p | RAB9B |
| AC053503.6 | hsa-miR-532-3p | PTGIS |
| AC053503.6 | hsa-miR-532-3p | CNN1 |
| AC053503.6 | hsa-miR-532-3p | POPDC3 |
| AC053503.6 | hsa-miR-532-3p | PI15 |
| AC053503.6 | hsa-miR-532-3p | ABCC9 |
| AC053503.6 | hsa-miR-532-3p | ASPA |
| AC053503.6 | hsa-miR-532-3p | NGEF |
| AC053503.6 | hsa-miR-532-3p | MYL9 |
| AC053503.6 | hsa-miR-532-3p | KCNE4 |
| AC053503.6 | hsa-miR-532-3p | PDLIM3 |
| AC053503.6 | hsa-miR-532-3p | UNC5D |
| AC053503.6 | hsa-miR-532-3p | FBXO27 |
| AC053503.6 | hsa-miR-532-3p | KCNIP3 |
| AC053503.6 | hsa-miR-532-3p | TCF23 |
| AC053503.6 | hsa-miR-532-3p | MASP1 |
| AC053503.6 | hsa-miR-532-3p | HPGD |
| AC053503.6 | hsa-miR-532-3p | PRRT2 |
| AC053503.6 | hsa-miR-532-3p | KCNAB1 |
| AC053503.6 | hsa-miR-532-3p | KCNK3 |
| AC053503.6 | hsa-miR-532-3p | SLC38A11 |
| AC053503.6 | hsa-miR-532-3p | ADH1B |
| AC053503.6 | hsa-miR-532-3p | TUB |
| AC053503.6 | hsa-miR-532-3p | MSRB3 |
| AC053503.6 | hsa-miR-532-3p | SPEG |
| AC053503.6 | hsa-miR-532-3p | LPP |
| AC053503.6 | hsa-miR-532-3p | C7 |
| AC053503.6 | hsa-miR-532-3p | EBF1 |
| AC053503.6 | hsa-miR-532-3p | BVES |
| AC053503.6 | hsa-miR-532-3p | CLU |
| AC053503.6 | hsa-miR-532-3p | PLAG1 |
| AC053503.6 | hsa-miR-532-3p | SLC2A4 |
| AC053503.6 | hsa-miR-532-3p | FIBIN |
| AC053503.6 | hsa-miR-532-3p | DUOX1 |
| AC053503.6 | hsa-miR-532-3p | CAPN6 |
| AC053503.6 | hsa-miR-532-3p | DMRTA1 |
| AC053503.6 | hsa-miR-532-3p | C3orf80 |
| AC053503.6 | hsa-miR-532-3p | GDNF |
| AC053503.6 | hsa-miR-532-3p | TNFAIP8L3 |
| AC053503.6 | hsa-miR-532-3p | SYNGR1 |
| AC053503.6 | hsa-miR-532-3p | WSCD2 |
| AC053503.6 | hsa-miR-532-3p | C3orf70 |
| AC053503.6 | hsa-miR-532-3p | TSPAN18 |
| AC053503.6 | hsa-miR-532-3p | PRELP |
| AC053503.6 | hsa-miR-532-3p | THNSL2 |
| AC053503.6 | hsa-miR-532-3p | NR3C2 |
| AC053503.6 | hsa-miR-532-3p | SMOC2 |
| AC053503.6 | hsa-miR-532-3p | JPH4 |
| AC053503.6 | hsa-miR-532-3p | DCX |
| AC053503.6 | hsa-miR-532-3p | ANKRD36 |
| AC053503.6 | hsa-miR-532-3p | CACNA1H |
| AC053503.6 | hsa-miR-532-3p | NFIX |
| AC053503.6 | hsa-miR-532-3p | CNR1 |
| AC053503.6 | hsa-miR-532-3p | LMOD1 |
| AC053503.6 | hsa-miR-532-3p | GPR161 |
| AC053503.6 | hsa-miR-532-3p | PBX1 |
| AC053503.6 | hsa-miR-532-3p | RBM20 |
| AC053503.6 | hsa-miR-532-3p | CDR1 |
| AC053503.6 | hsa-miR-532-3p | KCNB1 |
| AC053503.6 | hsa-miR-532-3p | PAK3 |
| AC053503.6 | hsa-miR-532-3p | CHRDL1 |
| AC053503.6 | hsa-miR-532-3p | SLC6A9 |
| AC053503.6 | hsa-miR-532-3p | HYI |
| AC053503.6 | hsa-miR-532-3p | FOXO6 |
| AC053503.6 | hsa-miR-532-3p | JPH2 |
| AC053503.6 | hsa-miR-532-3p | RSPO1 |
| AC053503.6 | hsa-miR-532-3p | FABP3 |
| AC053503.6 | hsa-miR-532-3p | DES |
| AC053503.6 | hsa-miR-532-3p | TRPM3 |
| AC053503.6 | hsa-miR-532-3p | MAMDC2 |
| AC053503.6 | hsa-miR-532-3p | PKHD1L1 |
| AC053503.6 | hsa-miR-532-3p | DMD |
| AC053503.6 | hsa-miR-532-3p | PTCHD1 |
| AC053503.6 | hsa-miR-532-3p | BNC2 |
| AC053503.6 | hsa-miR-532-3p | SMOC1 |
| AC053503.6 | hsa-miR-532-3p | CYS1 |
| AC053503.6 | hsa-miR-532-3p | SEMA5A |
| AC053503.6 | hsa-miR-532-3p | RYR3 |
| AC053503.6 | hsa-miR-532-3p | CDON |
| AC053503.6 | hsa-miR-532-3p | SPOCK1 |
| AC053503.6 | hsa-miR-532-3p | FAM13A |
| AC053503.6 | hsa-miR-532-3p | ST8SIA1 |
| AC053503.6 | hsa-miR-532-3p | ZNF208 |
| AC053503.6 | hsa-miR-532-3p | NFASC |
| AC053503.6 | hsa-miR-532-3p | DGKB |
| AC053503.6 | hsa-miR-532-3p | LIMS2 |
| AC053503.6 | hsa-miR-532-3p | HSPB7 |
| AC053503.6 | hsa-miR-532-3p | MRVI1 |
| AC053503.6 | hsa-miR-532-3p | ITGA11 |
| AC053503.6 | hsa-miR-532-3p | HMCN2 |
| AC053503.6 | hsa-miR-532-3p | SYNPO2 |
| AC053503.6 | hsa-miR-532-3p | GFRA1 |
| AC053503.6 | hsa-miR-532-3p | MRGPRF |
| AC053503.6 | hsa-miR-532-3p | TCEA3 |
| AC053503.6 | hsa-miR-532-3p | FAM107A |
| AC053503.6 | hsa-miR-532-3p | KLF8 |
| AC053503.6 | hsa-miR-532-3p | FSBP |
| AC053503.6 | hsa-miR-532-3p | SLC15A2 |
| AC053503.6 | hsa-miR-532-3p | SORCS2 |
| AC053503.6 | hsa-miR-532-3p | TMEM200B |
| AC053503.6 | hsa-miR-532-3p | YPEL4 |
| AC053503.6 | hsa-miR-532-3p | NAALAD2 |
| AC053503.6 | hsa-miR-532-3p | PIANP |
| AC053503.6 | hsa-miR-532-3p | LDB3 |
| AC053503.6 | hsa-miR-532-3p | DUOXA1 |
| AC053503.6 | hsa-miR-532-3p | DET1 |
| AC053503.6 | hsa-miR-532-3p | RBFOX3 |
| AC053503.6 | hsa-miR-532-3p | CCDC68 |
| AC053503.6 | hsa-miR-532-3p | PPP1R12B |
| LINC01482 | hsa-miR-532-3p | RERGL |
| LINC01482 | hsa-miR-532-3p | RAB9B |
| LINC01482 | hsa-miR-532-3p | PTGIS |
| LINC01482 | hsa-miR-532-3p | CNN1 |
| LINC01482 | hsa-miR-532-3p | POPDC3 |
| LINC01482 | hsa-miR-532-3p | PI15 |
| LINC01482 | hsa-miR-532-3p | ABCC9 |
| LINC01482 | hsa-miR-532-3p | ASPA |
| LINC01482 | hsa-miR-532-3p | NGEF |
| LINC01482 | hsa-miR-532-3p | MYL9 |
| LINC01482 | hsa-miR-532-3p | KCNE4 |
| LINC01482 | hsa-miR-532-3p | PDLIM3 |
| LINC01482 | hsa-miR-532-3p | UNC5D |
| LINC01482 | hsa-miR-532-3p | FBXO27 |
| LINC01482 | hsa-miR-532-3p | KCNIP3 |
| LINC01482 | hsa-miR-532-3p | TCF23 |
| LINC01482 | hsa-miR-532-3p | MASP1 |
| LINC01482 | hsa-miR-532-3p | HPGD |
| LINC01482 | hsa-miR-532-3p | PRRT2 |
| LINC01482 | hsa-miR-532-3p | KCNAB1 |
| LINC01482 | hsa-miR-532-3p | KCNK3 |
| LINC01482 | hsa-miR-532-3p | SLC38A11 |
| LINC01482 | hsa-miR-532-3p | ADH1B |
| LINC01482 | hsa-miR-532-3p | TUB |
| LINC01482 | hsa-miR-532-3p | MSRB3 |
| LINC01482 | hsa-miR-532-3p | SPEG |
| LINC01482 | hsa-miR-532-3p | LPP |
| LINC01482 | hsa-miR-532-3p | C7 |
| LINC01482 | hsa-miR-532-3p | EBF1 |
| LINC01482 | hsa-miR-532-3p | BVES |
| LINC01482 | hsa-miR-532-3p | CLU |
| LINC01482 | hsa-miR-532-3p | PLAG1 |
| LINC01482 | hsa-miR-532-3p | SLC2A4 |
| LINC01482 | hsa-miR-532-3p | FIBIN |
| LINC01482 | hsa-miR-532-3p | DUOX1 |
| LINC01482 | hsa-miR-532-3p | CAPN6 |
| LINC01482 | hsa-miR-532-3p | DMRTA1 |
| LINC01482 | hsa-miR-532-3p | C3orf80 |
| LINC01482 | hsa-miR-532-3p | GDNF |
| LINC01482 | hsa-miR-532-3p | TNFAIP8L3 |
| LINC01482 | hsa-miR-532-3p | SYNGR1 |
| LINC01482 | hsa-miR-532-3p | WSCD2 |
| LINC01482 | hsa-miR-532-3p | C3orf70 |
| LINC01482 | hsa-miR-532-3p | TSPAN18 |
| LINC01482 | hsa-miR-532-3p | PRELP |
| LINC01482 | hsa-miR-532-3p | THNSL2 |
| LINC01482 | hsa-miR-532-3p | NR3C2 |
| LINC01482 | hsa-miR-532-3p | SMOC2 |
| LINC01482 | hsa-miR-532-3p | JPH4 |
| LINC01482 | hsa-miR-532-3p | DCX |
| LINC01482 | hsa-miR-532-3p | ANKRD36 |
| LINC01482 | hsa-miR-532-3p | CACNA1H |
| LINC01482 | hsa-miR-532-3p | NFIX |
| LINC01482 | hsa-miR-532-3p | CNR1 |
| LINC01482 | hsa-miR-532-3p | LMOD1 |
| LINC01482 | hsa-miR-532-3p | GPR161 |
| LINC01482 | hsa-miR-532-3p | PBX1 |
| LINC01482 | hsa-miR-532-3p | RBM20 |
| LINC01482 | hsa-miR-532-3p | CDR1 |
| LINC01482 | hsa-miR-532-3p | KCNB1 |
| LINC01482 | hsa-miR-532-3p | PAK3 |
| LINC01482 | hsa-miR-532-3p | CHRDL1 |
| LINC01482 | hsa-miR-532-3p | SLC6A9 |
| LINC01482 | hsa-miR-532-3p | HYI |
| LINC01482 | hsa-miR-532-3p | FOXO6 |
| LINC01482 | hsa-miR-532-3p | JPH2 |
| LINC01482 | hsa-miR-532-3p | RSPO1 |
| LINC01482 | hsa-miR-532-3p | FABP3 |
| LINC01482 | hsa-miR-532-3p | DES |
| LINC01482 | hsa-miR-532-3p | TRPM3 |
| LINC01482 | hsa-miR-532-3p | MAMDC2 |
| LINC01482 | hsa-miR-532-3p | PKHD1L1 |
| LINC01482 | hsa-miR-532-3p | DMD |
| LINC01482 | hsa-miR-532-3p | PTCHD1 |
| LINC01482 | hsa-miR-532-3p | BNC2 |
| LINC01482 | hsa-miR-532-3p | SMOC1 |
| LINC01482 | hsa-miR-532-3p | CYS1 |
| LINC01482 | hsa-miR-532-3p | SEMA5A |
| LINC01482 | hsa-miR-532-3p | RYR3 |
| LINC01482 | hsa-miR-532-3p | CDON |
| LINC01482 | hsa-miR-532-3p | SPOCK1 |
| LINC01482 | hsa-miR-532-3p | FAM13A |
| LINC01482 | hsa-miR-532-3p | ST8SIA1 |
| LINC01482 | hsa-miR-532-3p | ZNF208 |
| LINC01482 | hsa-miR-532-3p | NFASC |
| LINC01482 | hsa-miR-532-3p | DGKB |
| LINC01482 | hsa-miR-532-3p | LIMS2 |
| LINC01482 | hsa-miR-532-3p | HSPB7 |
| LINC01482 | hsa-miR-532-3p | MRVI1 |
| LINC01482 | hsa-miR-532-3p | ITGA11 |
| LINC01482 | hsa-miR-532-3p | HMCN2 |
| LINC01482 | hsa-miR-532-3p | SYNPO2 |
| LINC01482 | hsa-miR-532-3p | GFRA1 |
| LINC01482 | hsa-miR-532-3p | MRGPRF |
| LINC01482 | hsa-miR-532-3p | TCEA3 |
| LINC01482 | hsa-miR-532-3p | FAM107A |
| LINC01482 | hsa-miR-532-3p | KLF8 |
| LINC01482 | hsa-miR-532-3p | FSBP |
| LINC01482 | hsa-miR-532-3p | SLC15A2 |
| LINC01482 | hsa-miR-532-3p | SORCS2 |
| LINC01482 | hsa-miR-532-3p | TMEM200B |
| LINC01482 | hsa-miR-532-3p | YPEL4 |
| LINC01482 | hsa-miR-532-3p | NAALAD2 |
| LINC01482 | hsa-miR-532-3p | PIANP |
| LINC01482 | hsa-miR-532-3p | LDB3 |
| LINC01482 | hsa-miR-532-3p | DUOXA1 |
| LINC01482 | hsa-miR-532-3p | DET1 |
| LINC01482 | hsa-miR-532-3p | RBFOX3 |
| LINC01482 | hsa-miR-532-3p | CCDC68 |
| LINC01482 | hsa-miR-532-3p | PPP1R12B |
| AP000344.1 | hsa-miR-532-3p | RERGL |
| AP000344.1 | hsa-miR-532-3p | RAB9B |
| AP000344.1 | hsa-miR-532-3p | PTGIS |
| AP000344.1 | hsa-miR-532-3p | CNN1 |
| AP000344.1 | hsa-miR-532-3p | POPDC3 |
| AP000344.1 | hsa-miR-532-3p | PI15 |
| AP000344.1 | hsa-miR-532-3p | ABCC9 |
| AP000344.1 | hsa-miR-532-3p | ASPA |
| AP000344.1 | hsa-miR-532-3p | NGEF |
| AP000344.1 | hsa-miR-532-3p | MYL9 |
| AP000344.1 | hsa-miR-532-3p | KCNE4 |
| AP000344.1 | hsa-miR-532-3p | PDLIM3 |
| AP000344.1 | hsa-miR-532-3p | UNC5D |
| AP000344.1 | hsa-miR-532-3p | FBXO27 |
| AP000344.1 | hsa-miR-532-3p | KCNIP3 |
| AP000344.1 | hsa-miR-532-3p | TCF23 |
| AP000344.1 | hsa-miR-532-3p | MASP1 |
| AP000344.1 | hsa-miR-532-3p | HPGD |
| AP000344.1 | hsa-miR-532-3p | PRRT2 |
| AP000344.1 | hsa-miR-532-3p | KCNAB1 |
| AP000344.1 | hsa-miR-532-3p | KCNK3 |
| AP000344.1 | hsa-miR-532-3p | SLC38A11 |
| AP000344.1 | hsa-miR-532-3p | ADH1B |
| AP000344.1 | hsa-miR-532-3p | TUB |
| AP000344.1 | hsa-miR-532-3p | MSRB3 |
| AP000344.1 | hsa-miR-532-3p | SPEG |
| AP000344.1 | hsa-miR-532-3p | LPP |
| AP000344.1 | hsa-miR-532-3p | C7 |
| AP000344.1 | hsa-miR-532-3p | EBF1 |
| AP000344.1 | hsa-miR-532-3p | BVES |
| AP000344.1 | hsa-miR-532-3p | CLU |
| AP000344.1 | hsa-miR-532-3p | PLAG1 |
| AP000344.1 | hsa-miR-532-3p | SLC2A4 |
| AP000344.1 | hsa-miR-532-3p | FIBIN |
| AP000344.1 | hsa-miR-532-3p | DUOX1 |
| AP000344.1 | hsa-miR-532-3p | CAPN6 |
| AP000344.1 | hsa-miR-532-3p | DMRTA1 |
| AP000344.1 | hsa-miR-532-3p | C3orf80 |
| AP000344.1 | hsa-miR-532-3p | GDNF |
| AP000344.1 | hsa-miR-532-3p | TNFAIP8L3 |
| AP000344.1 | hsa-miR-532-3p | SYNGR1 |
| AP000344.1 | hsa-miR-532-3p | WSCD2 |
| AP000344.1 | hsa-miR-532-3p | C3orf70 |
| AP000344.1 | hsa-miR-532-3p | TSPAN18 |
| AP000344.1 | hsa-miR-532-3p | PRELP |
| AP000344.1 | hsa-miR-532-3p | THNSL2 |
| AP000344.1 | hsa-miR-532-3p | NR3C2 |
| AP000344.1 | hsa-miR-532-3p | SMOC2 |
| AP000344.1 | hsa-miR-532-3p | JPH4 |
| AP000344.1 | hsa-miR-532-3p | DCX |
| AP000344.1 | hsa-miR-532-3p | ANKRD36 |
| AP000344.1 | hsa-miR-532-3p | CACNA1H |
| AP000344.1 | hsa-miR-532-3p | NFIX |
| AP000344.1 | hsa-miR-532-3p | CNR1 |
| AP000344.1 | hsa-miR-532-3p | LMOD1 |
| AP000344.1 | hsa-miR-532-3p | GPR161 |
| AP000344.1 | hsa-miR-532-3p | PBX1 |
| AP000344.1 | hsa-miR-532-3p | RBM20 |
| AP000344.1 | hsa-miR-532-3p | CDR1 |
| AP000344.1 | hsa-miR-532-3p | KCNB1 |
| AP000344.1 | hsa-miR-532-3p | PAK3 |
| AP000344.1 | hsa-miR-532-3p | CHRDL1 |
| AP000344.1 | hsa-miR-532-3p | SLC6A9 |
| AP000344.1 | hsa-miR-532-3p | HYI |
| AP000344.1 | hsa-miR-532-3p | FOXO6 |
| AP000344.1 | hsa-miR-532-3p | JPH2 |
| AP000344.1 | hsa-miR-532-3p | RSPO1 |
| AP000344.1 | hsa-miR-532-3p | FABP3 |
| AP000344.1 | hsa-miR-532-3p | DES |
| AP000344.1 | hsa-miR-532-3p | TRPM3 |
| AP000344.1 | hsa-miR-532-3p | MAMDC2 |
| AP000344.1 | hsa-miR-532-3p | PKHD1L1 |
| AP000344.1 | hsa-miR-532-3p | DMD |
| AP000344.1 | hsa-miR-532-3p | PTCHD1 |
| AP000344.1 | hsa-miR-532-3p | BNC2 |
| AP000344.1 | hsa-miR-532-3p | SMOC1 |
| AP000344.1 | hsa-miR-532-3p | CYS1 |
| AP000344.1 | hsa-miR-532-3p | SEMA5A |
| AP000344.1 | hsa-miR-532-3p | RYR3 |
| AP000344.1 | hsa-miR-532-3p | CDON |
| AP000344.1 | hsa-miR-532-3p | SPOCK1 |
| AP000344.1 | hsa-miR-532-3p | FAM13A |
| AP000344.1 | hsa-miR-532-3p | ST8SIA1 |
| AP000344.1 | hsa-miR-532-3p | ZNF208 |
| AP000344.1 | hsa-miR-532-3p | NFASC |
| AP000344.1 | hsa-miR-532-3p | DGKB |
| AP000344.1 | hsa-miR-532-3p | LIMS2 |
| AP000344.1 | hsa-miR-532-3p | HSPB7 |
| AP000344.1 | hsa-miR-532-3p | MRVI1 |
| AP000344.1 | hsa-miR-532-3p | ITGA11 |
| AP000344.1 | hsa-miR-532-3p | HMCN2 |
| AP000344.1 | hsa-miR-532-3p | SYNPO2 |
| AP000344.1 | hsa-miR-532-3p | GFRA1 |
| AP000344.1 | hsa-miR-532-3p | MRGPRF |
| AP000344.1 | hsa-miR-532-3p | TCEA3 |
| AP000344.1 | hsa-miR-532-3p | FAM107A |
| AP000344.1 | hsa-miR-532-3p | KLF8 |
| AP000344.1 | hsa-miR-532-3p | FSBP |
| AP000344.1 | hsa-miR-532-3p | SLC15A2 |
| AP000344.1 | hsa-miR-532-3p | SORCS2 |
| AP000344.1 | hsa-miR-532-3p | TMEM200B |
| AP000344.1 | hsa-miR-532-3p | YPEL4 |
| AP000344.1 | hsa-miR-532-3p | NAALAD2 |
| AP000344.1 | hsa-miR-532-3p | PIANP |
| AP000344.1 | hsa-miR-532-3p | LDB3 |
| AP000344.1 | hsa-miR-532-3p | DUOXA1 |
| AP000344.1 | hsa-miR-532-3p | DET1 |
| AP000344.1 | hsa-miR-532-3p | RBFOX3 |
| AP000344.1 | hsa-miR-532-3p | CCDC68 |
| AP000344.1 | hsa-miR-532-3p | PPP1R12B |
| GOLGA2P10 | hsa-miR-532-3p | RERGL |
| GOLGA2P10 | hsa-miR-532-3p | RAB9B |
| GOLGA2P10 | hsa-miR-532-3p | PTGIS |
| GOLGA2P10 | hsa-miR-532-3p | CNN1 |
| GOLGA2P10 | hsa-miR-532-3p | POPDC3 |
| GOLGA2P10 | hsa-miR-532-3p | PI15 |
| GOLGA2P10 | hsa-miR-532-3p | ABCC9 |
| GOLGA2P10 | hsa-miR-532-3p | ASPA |
| GOLGA2P10 | hsa-miR-532-3p | NGEF |
| GOLGA2P10 | hsa-miR-532-3p | MYL9 |
| GOLGA2P10 | hsa-miR-532-3p | KCNE4 |
| GOLGA2P10 | hsa-miR-532-3p | PDLIM3 |
| GOLGA2P10 | hsa-miR-532-3p | UNC5D |
| GOLGA2P10 | hsa-miR-532-3p | FBXO27 |
| GOLGA2P10 | hsa-miR-532-3p | KCNIP3 |
| GOLGA2P10 | hsa-miR-532-3p | TCF23 |
| GOLGA2P10 | hsa-miR-532-3p | MASP1 |
| GOLGA2P10 | hsa-miR-532-3p | HPGD |
| GOLGA2P10 | hsa-miR-532-3p | PRRT2 |
| GOLGA2P10 | hsa-miR-532-3p | KCNAB1 |
| GOLGA2P10 | hsa-miR-532-3p | KCNK3 |
| GOLGA2P10 | hsa-miR-532-3p | SLC38A11 |
| GOLGA2P10 | hsa-miR-532-3p | ADH1B |
| GOLGA2P10 | hsa-miR-532-3p | TUB |
| GOLGA2P10 | hsa-miR-532-3p | MSRB3 |
| GOLGA2P10 | hsa-miR-532-3p | SPEG |
| GOLGA2P10 | hsa-miR-532-3p | LPP |
| GOLGA2P10 | hsa-miR-532-3p | C7 |
| GOLGA2P10 | hsa-miR-532-3p | EBF1 |
| GOLGA2P10 | hsa-miR-532-3p | BVES |
| GOLGA2P10 | hsa-miR-532-3p | CLU |
| GOLGA2P10 | hsa-miR-532-3p | PLAG1 |
| GOLGA2P10 | hsa-miR-532-3p | SLC2A4 |
| GOLGA2P10 | hsa-miR-532-3p | FIBIN |
| GOLGA2P10 | hsa-miR-532-3p | DUOX1 |
| GOLGA2P10 | hsa-miR-532-3p | CAPN6 |
| GOLGA2P10 | hsa-miR-532-3p | DMRTA1 |
| GOLGA2P10 | hsa-miR-532-3p | C3orf80 |
| GOLGA2P10 | hsa-miR-532-3p | GDNF |
| GOLGA2P10 | hsa-miR-532-3p | TNFAIP8L3 |
| GOLGA2P10 | hsa-miR-532-3p | SYNGR1 |
| GOLGA2P10 | hsa-miR-532-3p | WSCD2 |
| GOLGA2P10 | hsa-miR-532-3p | C3orf70 |
| GOLGA2P10 | hsa-miR-532-3p | TSPAN18 |
| GOLGA2P10 | hsa-miR-532-3p | PRELP |
| GOLGA2P10 | hsa-miR-532-3p | THNSL2 |
| GOLGA2P10 | hsa-miR-532-3p | NR3C2 |
| GOLGA2P10 | hsa-miR-532-3p | SMOC2 |
| GOLGA2P10 | hsa-miR-532-3p | JPH4 |
| GOLGA2P10 | hsa-miR-532-3p | DCX |
| GOLGA2P10 | hsa-miR-532-3p | ANKRD36 |
| GOLGA2P10 | hsa-miR-532-3p | CACNA1H |
| GOLGA2P10 | hsa-miR-532-3p | NFIX |
| GOLGA2P10 | hsa-miR-532-3p | CNR1 |
| GOLGA2P10 | hsa-miR-532-3p | LMOD1 |
| GOLGA2P10 | hsa-miR-532-3p | GPR161 |
| GOLGA2P10 | hsa-miR-532-3p | PBX1 |
| GOLGA2P10 | hsa-miR-532-3p | RBM20 |
| GOLGA2P10 | hsa-miR-532-3p | CDR1 |
| GOLGA2P10 | hsa-miR-532-3p | KCNB1 |
| GOLGA2P10 | hsa-miR-532-3p | PAK3 |
| GOLGA2P10 | hsa-miR-532-3p | CHRDL1 |
| GOLGA2P10 | hsa-miR-532-3p | SLC6A9 |
| GOLGA2P10 | hsa-miR-532-3p | HYI |
| GOLGA2P10 | hsa-miR-532-3p | FOXO6 |
| GOLGA2P10 | hsa-miR-532-3p | JPH2 |
| GOLGA2P10 | hsa-miR-532-3p | RSPO1 |
| GOLGA2P10 | hsa-miR-532-3p | FABP3 |
| GOLGA2P10 | hsa-miR-532-3p | DES |
| GOLGA2P10 | hsa-miR-532-3p | TRPM3 |
| GOLGA2P10 | hsa-miR-532-3p | MAMDC2 |
| GOLGA2P10 | hsa-miR-532-3p | PKHD1L1 |
| GOLGA2P10 | hsa-miR-532-3p | DMD |
| GOLGA2P10 | hsa-miR-532-3p | PTCHD1 |
| GOLGA2P10 | hsa-miR-532-3p | BNC2 |
| GOLGA2P10 | hsa-miR-532-3p | SMOC1 |
| GOLGA2P10 | hsa-miR-532-3p | CYS1 |
| GOLGA2P10 | hsa-miR-532-3p | SEMA5A |
| GOLGA2P10 | hsa-miR-532-3p | RYR3 |
| GOLGA2P10 | hsa-miR-532-3p | CDON |
| GOLGA2P10 | hsa-miR-532-3p | SPOCK1 |
| GOLGA2P10 | hsa-miR-532-3p | FAM13A |
| GOLGA2P10 | hsa-miR-532-3p | ST8SIA1 |
| GOLGA2P10 | hsa-miR-532-3p | ZNF208 |
| GOLGA2P10 | hsa-miR-532-3p | NFASC |
| GOLGA2P10 | hsa-miR-532-3p | DGKB |
| GOLGA2P10 | hsa-miR-532-3p | LIMS2 |
| GOLGA2P10 | hsa-miR-532-3p | HSPB7 |
| GOLGA2P10 | hsa-miR-532-3p | MRVI1 |
| GOLGA2P10 | hsa-miR-532-3p | ITGA11 |
| GOLGA2P10 | hsa-miR-532-3p | HMCN2 |
| GOLGA2P10 | hsa-miR-532-3p | SYNPO2 |
| GOLGA2P10 | hsa-miR-532-3p | GFRA1 |
| GOLGA2P10 | hsa-miR-532-3p | MRGPRF |
| GOLGA2P10 | hsa-miR-532-3p | TCEA3 |
| GOLGA2P10 | hsa-miR-532-3p | FAM107A |
| GOLGA2P10 | hsa-miR-532-3p | KLF8 |
| GOLGA2P10 | hsa-miR-532-3p | FSBP |
| GOLGA2P10 | hsa-miR-532-3p | SLC15A2 |
| GOLGA2P10 | hsa-miR-532-3p | SORCS2 |
| GOLGA2P10 | hsa-miR-532-3p | TMEM200B |
| GOLGA2P10 | hsa-miR-532-3p | YPEL4 |
| GOLGA2P10 | hsa-miR-532-3p | NAALAD2 |
| GOLGA2P10 | hsa-miR-532-3p | PIANP |
| GOLGA2P10 | hsa-miR-532-3p | LDB3 |
| GOLGA2P10 | hsa-miR-532-3p | DUOXA1 |
| GOLGA2P10 | hsa-miR-532-3p | DET1 |
| GOLGA2P10 | hsa-miR-532-3p | RBFOX3 |
| GOLGA2P10 | hsa-miR-532-3p | CCDC68 |
| GOLGA2P10 | hsa-miR-532-3p | PPP1R12B |
| AP003355.2 | hsa-miR-532-3p | RERGL |
| AP003355.2 | hsa-miR-532-3p | RAB9B |
| AP003355.2 | hsa-miR-532-3p | PTGIS |
| AP003355.2 | hsa-miR-532-3p | CNN1 |
| AP003355.2 | hsa-miR-532-3p | POPDC3 |
| AP003355.2 | hsa-miR-532-3p | PI15 |
| AP003355.2 | hsa-miR-532-3p | ABCC9 |
| AP003355.2 | hsa-miR-532-3p | ASPA |
| AP003355.2 | hsa-miR-532-3p | NGEF |
| AP003355.2 | hsa-miR-532-3p | MYL9 |
| AP003355.2 | hsa-miR-532-3p | KCNE4 |
| AP003355.2 | hsa-miR-532-3p | PDLIM3 |
| AP003355.2 | hsa-miR-532-3p | UNC5D |
| AP003355.2 | hsa-miR-532-3p | FBXO27 |
| AP003355.2 | hsa-miR-532-3p | KCNIP3 |
| AP003355.2 | hsa-miR-532-3p | TCF23 |
| AP003355.2 | hsa-miR-532-3p | MASP1 |
| AP003355.2 | hsa-miR-532-3p | HPGD |
| AP003355.2 | hsa-miR-532-3p | PRRT2 |
| AP003355.2 | hsa-miR-532-3p | KCNAB1 |
| AP003355.2 | hsa-miR-532-3p | KCNK3 |
| AP003355.2 | hsa-miR-532-3p | SLC38A11 |
| AP003355.2 | hsa-miR-532-3p | ADH1B |
| AP003355.2 | hsa-miR-532-3p | TUB |
| AP003355.2 | hsa-miR-532-3p | MSRB3 |
| AP003355.2 | hsa-miR-532-3p | SPEG |
| AP003355.2 | hsa-miR-532-3p | LPP |
| AP003355.2 | hsa-miR-532-3p | C7 |
| AP003355.2 | hsa-miR-532-3p | EBF1 |
| AP003355.2 | hsa-miR-532-3p | BVES |
| AP003355.2 | hsa-miR-532-3p | CLU |
| AP003355.2 | hsa-miR-532-3p | PLAG1 |
| AP003355.2 | hsa-miR-532-3p | SLC2A4 |
| AP003355.2 | hsa-miR-532-3p | FIBIN |
| AP003355.2 | hsa-miR-532-3p | DUOX1 |
| AP003355.2 | hsa-miR-532-3p | CAPN6 |
| AP003355.2 | hsa-miR-532-3p | DMRTA1 |
| AP003355.2 | hsa-miR-532-3p | C3orf80 |
| AP003355.2 | hsa-miR-532-3p | GDNF |
| AP003355.2 | hsa-miR-532-3p | TNFAIP8L3 |
| AP003355.2 | hsa-miR-532-3p | SYNGR1 |
| AP003355.2 | hsa-miR-532-3p | WSCD2 |
| AP003355.2 | hsa-miR-532-3p | C3orf70 |
| AP003355.2 | hsa-miR-532-3p | TSPAN18 |
| AP003355.2 | hsa-miR-532-3p | PRELP |
| AP003355.2 | hsa-miR-532-3p | THNSL2 |
| AP003355.2 | hsa-miR-532-3p | NR3C2 |
| AP003355.2 | hsa-miR-532-3p | SMOC2 |
| AP003355.2 | hsa-miR-532-3p | JPH4 |
| AP003355.2 | hsa-miR-532-3p | DCX |
| AP003355.2 | hsa-miR-532-3p | ANKRD36 |
| AP003355.2 | hsa-miR-532-3p | CACNA1H |
| AP003355.2 | hsa-miR-532-3p | NFIX |
| AP003355.2 | hsa-miR-532-3p | CNR1 |
| AP003355.2 | hsa-miR-532-3p | LMOD1 |
| AP003355.2 | hsa-miR-532-3p | GPR161 |
| AP003355.2 | hsa-miR-532-3p | PBX1 |
| AP003355.2 | hsa-miR-532-3p | RBM20 |
| AP003355.2 | hsa-miR-532-3p | CDR1 |
| AP003355.2 | hsa-miR-532-3p | KCNB1 |
| AP003355.2 | hsa-miR-532-3p | PAK3 |
| AP003355.2 | hsa-miR-532-3p | CHRDL1 |
| AP003355.2 | hsa-miR-532-3p | SLC6A9 |
| AP003355.2 | hsa-miR-532-3p | HYI |
| AP003355.2 | hsa-miR-532-3p | FOXO6 |
| AP003355.2 | hsa-miR-532-3p | JPH2 |
| AP003355.2 | hsa-miR-532-3p | RSPO1 |
| AP003355.2 | hsa-miR-532-3p | FABP3 |
| AP003355.2 | hsa-miR-532-3p | DES |
| AP003355.2 | hsa-miR-532-3p | TRPM3 |
| AP003355.2 | hsa-miR-532-3p | MAMDC2 |
| AP003355.2 | hsa-miR-532-3p | PKHD1L1 |
| AP003355.2 | hsa-miR-532-3p | DMD |
| AP003355.2 | hsa-miR-532-3p | PTCHD1 |
| AP003355.2 | hsa-miR-532-3p | BNC2 |
| AP003355.2 | hsa-miR-532-3p | SMOC1 |
| AP003355.2 | hsa-miR-532-3p | CYS1 |
| AP003355.2 | hsa-miR-532-3p | SEMA5A |
| AP003355.2 | hsa-miR-532-3p | RYR3 |
| AP003355.2 | hsa-miR-532-3p | CDON |
| AP003355.2 | hsa-miR-532-3p | SPOCK1 |
| AP003355.2 | hsa-miR-532-3p | FAM13A |
| AP003355.2 | hsa-miR-532-3p | ST8SIA1 |
| AP003355.2 | hsa-miR-532-3p | ZNF208 |
| AP003355.2 | hsa-miR-532-3p | NFASC |
| AP003355.2 | hsa-miR-532-3p | DGKB |
| AP003355.2 | hsa-miR-532-3p | LIMS2 |
| AP003355.2 | hsa-miR-532-3p | HSPB7 |
| AP003355.2 | hsa-miR-532-3p | MRVI1 |
| AP003355.2 | hsa-miR-532-3p | ITGA11 |
| AP003355.2 | hsa-miR-532-3p | HMCN2 |
| AP003355.2 | hsa-miR-532-3p | SYNPO2 |
| AP003355.2 | hsa-miR-532-3p | GFRA1 |
| AP003355.2 | hsa-miR-532-3p | MRGPRF |
| AP003355.2 | hsa-miR-532-3p | TCEA3 |
| AP003355.2 | hsa-miR-532-3p | FAM107A |
| AP003355.2 | hsa-miR-532-3p | KLF8 |
| AP003355.2 | hsa-miR-532-3p | FSBP |
| AP003355.2 | hsa-miR-532-3p | SLC15A2 |
| AP003355.2 | hsa-miR-532-3p | SORCS2 |
| AP003355.2 | hsa-miR-532-3p | TMEM200B |
| AP003355.2 | hsa-miR-532-3p | YPEL4 |
| AP003355.2 | hsa-miR-532-3p | NAALAD2 |
| AP003355.2 | hsa-miR-532-3p | PIANP |
| AP003355.2 | hsa-miR-532-3p | LDB3 |
| AP003355.2 | hsa-miR-532-3p | DUOXA1 |
| AP003355.2 | hsa-miR-532-3p | DET1 |
| AP003355.2 | hsa-miR-532-3p | RBFOX3 |
| AP003355.2 | hsa-miR-532-3p | CCDC68 |
| AP003355.2 | hsa-miR-532-3p | PPP1R12B |
| DIO3OS | hsa-miR-532-3p | RERGL |
| DIO3OS | hsa-miR-532-3p | RAB9B |
| DIO3OS | hsa-miR-532-3p | PTGIS |
| DIO3OS | hsa-miR-532-3p | CNN1 |
| DIO3OS | hsa-miR-532-3p | POPDC3 |
| DIO3OS | hsa-miR-532-3p | PI15 |
| DIO3OS | hsa-miR-532-3p | ABCC9 |
| DIO3OS | hsa-miR-532-3p | ASPA |
| DIO3OS | hsa-miR-532-3p | NGEF |
| DIO3OS | hsa-miR-532-3p | MYL9 |
| DIO3OS | hsa-miR-532-3p | KCNE4 |
| DIO3OS | hsa-miR-532-3p | PDLIM3 |
| DIO3OS | hsa-miR-532-3p | UNC5D |
| DIO3OS | hsa-miR-532-3p | FBXO27 |
| DIO3OS | hsa-miR-532-3p | KCNIP3 |
| DIO3OS | hsa-miR-532-3p | TCF23 |
| DIO3OS | hsa-miR-532-3p | MASP1 |
| DIO3OS | hsa-miR-532-3p | HPGD |
| DIO3OS | hsa-miR-532-3p | PRRT2 |
| DIO3OS | hsa-miR-532-3p | KCNAB1 |
| DIO3OS | hsa-miR-532-3p | KCNK3 |
| DIO3OS | hsa-miR-532-3p | SLC38A11 |
| DIO3OS | hsa-miR-532-3p | ADH1B |
| DIO3OS | hsa-miR-532-3p | TUB |
| DIO3OS | hsa-miR-532-3p | MSRB3 |
| DIO3OS | hsa-miR-532-3p | SPEG |
| DIO3OS | hsa-miR-532-3p | LPP |
| DIO3OS | hsa-miR-532-3p | C7 |
| DIO3OS | hsa-miR-532-3p | EBF1 |
| DIO3OS | hsa-miR-532-3p | BVES |
| DIO3OS | hsa-miR-532-3p | CLU |
| DIO3OS | hsa-miR-532-3p | PLAG1 |
| DIO3OS | hsa-miR-532-3p | SLC2A4 |
| DIO3OS | hsa-miR-532-3p | FIBIN |
| DIO3OS | hsa-miR-532-3p | DUOX1 |
| DIO3OS | hsa-miR-532-3p | CAPN6 |
| DIO3OS | hsa-miR-532-3p | DMRTA1 |
| DIO3OS | hsa-miR-532-3p | C3orf80 |
| DIO3OS | hsa-miR-532-3p | GDNF |
| DIO3OS | hsa-miR-532-3p | TNFAIP8L3 |
| DIO3OS | hsa-miR-532-3p | SYNGR1 |
| DIO3OS | hsa-miR-532-3p | WSCD2 |
| DIO3OS | hsa-miR-532-3p | C3orf70 |
| DIO3OS | hsa-miR-532-3p | TSPAN18 |
| DIO3OS | hsa-miR-532-3p | PRELP |
| DIO3OS | hsa-miR-532-3p | THNSL2 |
| DIO3OS | hsa-miR-532-3p | NR3C2 |
| DIO3OS | hsa-miR-532-3p | SMOC2 |
| DIO3OS | hsa-miR-532-3p | JPH4 |
| DIO3OS | hsa-miR-532-3p | DCX |
| DIO3OS | hsa-miR-532-3p | ANKRD36 |
| DIO3OS | hsa-miR-532-3p | CACNA1H |
| DIO3OS | hsa-miR-532-3p | NFIX |
| DIO3OS | hsa-miR-532-3p | CNR1 |
| DIO3OS | hsa-miR-532-3p | LMOD1 |
| DIO3OS | hsa-miR-532-3p | GPR161 |
| DIO3OS | hsa-miR-532-3p | PBX1 |
| DIO3OS | hsa-miR-532-3p | RBM20 |
| DIO3OS | hsa-miR-532-3p | CDR1 |
| DIO3OS | hsa-miR-532-3p | KCNB1 |
| DIO3OS | hsa-miR-532-3p | PAK3 |
| DIO3OS | hsa-miR-532-3p | CHRDL1 |
| DIO3OS | hsa-miR-532-3p | SLC6A9 |
| DIO3OS | hsa-miR-532-3p | HYI |
| DIO3OS | hsa-miR-532-3p | FOXO6 |
| DIO3OS | hsa-miR-532-3p | JPH2 |
| DIO3OS | hsa-miR-532-3p | RSPO1 |
| DIO3OS | hsa-miR-532-3p | FABP3 |
| DIO3OS | hsa-miR-532-3p | DES |
| DIO3OS | hsa-miR-532-3p | TRPM3 |
| DIO3OS | hsa-miR-532-3p | MAMDC2 |
| DIO3OS | hsa-miR-532-3p | PKHD1L1 |
| DIO3OS | hsa-miR-532-3p | DMD |
| DIO3OS | hsa-miR-532-3p | PTCHD1 |
| DIO3OS | hsa-miR-532-3p | BNC2 |
| DIO3OS | hsa-miR-532-3p | SMOC1 |
| DIO3OS | hsa-miR-532-3p | CYS1 |
| DIO3OS | hsa-miR-532-3p | SEMA5A |
| DIO3OS | hsa-miR-532-3p | RYR3 |
| DIO3OS | hsa-miR-532-3p | CDON |
| DIO3OS | hsa-miR-532-3p | SPOCK1 |
| DIO3OS | hsa-miR-532-3p | FAM13A |
| DIO3OS | hsa-miR-532-3p | ST8SIA1 |
| DIO3OS | hsa-miR-532-3p | ZNF208 |
| DIO3OS | hsa-miR-532-3p | NFASC |
| DIO3OS | hsa-miR-532-3p | DGKB |
| DIO3OS | hsa-miR-532-3p | LIMS2 |
| DIO3OS | hsa-miR-532-3p | HSPB7 |
| DIO3OS | hsa-miR-532-3p | MRVI1 |
| DIO3OS | hsa-miR-532-3p | ITGA11 |
| DIO3OS | hsa-miR-532-3p | HMCN2 |
| DIO3OS | hsa-miR-532-3p | SYNPO2 |
| DIO3OS | hsa-miR-532-3p | GFRA1 |
| DIO3OS | hsa-miR-532-3p | MRGPRF |
| DIO3OS | hsa-miR-532-3p | TCEA3 |
| DIO3OS | hsa-miR-532-3p | FAM107A |
| DIO3OS | hsa-miR-532-3p | KLF8 |
| DIO3OS | hsa-miR-532-3p | FSBP |
| DIO3OS | hsa-miR-532-3p | SLC15A2 |
| DIO3OS | hsa-miR-532-3p | SORCS2 |
| DIO3OS | hsa-miR-532-3p | TMEM200B |
| DIO3OS | hsa-miR-532-3p | YPEL4 |
| DIO3OS | hsa-miR-532-3p | NAALAD2 |
| DIO3OS | hsa-miR-532-3p | PIANP |
| DIO3OS | hsa-miR-532-3p | LDB3 |
| DIO3OS | hsa-miR-532-3p | DUOXA1 |
| DIO3OS | hsa-miR-532-3p | DET1 |
| DIO3OS | hsa-miR-532-3p | RBFOX3 |
| DIO3OS | hsa-miR-532-3p | CCDC68 |
| DIO3OS | hsa-miR-532-3p | PPP1R12B |
| ADIRF-AS1 | hsa-miR-744-5p | ADAM11 |
| ADIRF-AS1 | hsa-miR-744-5p | HOXA13 |
| ADIRF-AS1 | hsa-miR-744-5p | GNAZ |
| ADIRF-AS1 | hsa-miR-744-5p | ASXL3 |
| ADIRF-AS1 | hsa-miR-744-5p | OSR1 |
| ADIRF-AS1 | hsa-miR-744-5p | MYL9 |
| ADIRF-AS1 | hsa-miR-744-5p | MASP1 |
| ADIRF-AS1 | hsa-miR-744-5p | PRRT2 |
| ADIRF-AS1 | hsa-miR-744-5p | HSPB3 |
| ADIRF-AS1 | hsa-miR-744-5p | KCNK3 |
| ADIRF-AS1 | hsa-miR-744-5p | CPLX1 |
| ADIRF-AS1 | hsa-miR-744-5p | LPP |
| ADIRF-AS1 | hsa-miR-744-5p | C11orf87 |
| ADIRF-AS1 | hsa-miR-744-5p | SYNGR1 |
| ADIRF-AS1 | hsa-miR-744-5p | C3orf70 |
| ADIRF-AS1 | hsa-miR-744-5p | PRELP |
| ADIRF-AS1 | hsa-miR-744-5p | ASIC3 |
| ADIRF-AS1 | hsa-miR-744-5p | ATP2B2 |
| ADIRF-AS1 | hsa-miR-744-5p | CACNA1H |
| ADIRF-AS1 | hsa-miR-744-5p | NFIX |
| ADIRF-AS1 | hsa-miR-744-5p | LDOC1 |
| ADIRF-AS1 | hsa-miR-744-5p | SLC25A27 |
| ADIRF-AS1 | hsa-miR-744-5p | RAB40A |
| ADIRF-AS1 | hsa-miR-744-5p | DES |
| ADIRF-AS1 | hsa-miR-744-5p | GPR20 |
| ADIRF-AS1 | hsa-miR-744-5p | CCDC3 |
| ADIRF-AS1 | hsa-miR-744-5p | PTCHD1 |
| ADIRF-AS1 | hsa-miR-744-5p | IGF2 |
| ADIRF-AS1 | hsa-miR-744-5p | CYS1 |
| ADIRF-AS1 | hsa-miR-744-5p | LGI2 |
| ADIRF-AS1 | hsa-miR-744-5p | CDON |
| ADIRF-AS1 | hsa-miR-744-5p | KCNG1 |
| ADIRF-AS1 | hsa-miR-744-5p | NFASC |
| ADIRF-AS1 | hsa-miR-744-5p | LIMS2 |
| ADIRF-AS1 | hsa-miR-744-5p | MRGPRF |
| ADIRF-AS1 | hsa-miR-744-5p | FAM107A |
| ADIRF-AS1 | hsa-miR-744-5p | TMEM158 |
| ADIRF-AS1 | hsa-miR-744-5p | SORCS2 |
| ADIRF-AS1 | hsa-miR-744-5p | FXYD6 |
| ADIRF-AS1 | hsa-miR-744-5p | IGFBP6 |
| ADIRF-AS1 | hsa-miR-744-5p | NDUFA4L2 |
| ADIRF-AS1 | hsa-miR-744-5p | PPP1R12B |
| AC100803.2 | hsa-miR-744-5p | ADAM11 |
| AC100803.2 | hsa-miR-744-5p | HOXA13 |
| AC100803.2 | hsa-miR-744-5p | GNAZ |
| AC100803.2 | hsa-miR-744-5p | ASXL3 |
| AC100803.2 | hsa-miR-744-5p | OSR1 |
| AC100803.2 | hsa-miR-744-5p | MYL9 |
| AC100803.2 | hsa-miR-744-5p | MASP1 |
| AC100803.2 | hsa-miR-744-5p | PRRT2 |
| AC100803.2 | hsa-miR-744-5p | HSPB3 |
| AC100803.2 | hsa-miR-744-5p | KCNK3 |
| AC100803.2 | hsa-miR-744-5p | CPLX1 |
| AC100803.2 | hsa-miR-744-5p | LPP |
| AC100803.2 | hsa-miR-744-5p | C11orf87 |
| AC100803.2 | hsa-miR-744-5p | SYNGR1 |
| AC100803.2 | hsa-miR-744-5p | C3orf70 |
| AC100803.2 | hsa-miR-744-5p | PRELP |
| AC100803.2 | hsa-miR-744-5p | ASIC3 |
| AC100803.2 | hsa-miR-744-5p | ATP2B2 |
| AC100803.2 | hsa-miR-744-5p | CACNA1H |
| AC100803.2 | hsa-miR-744-5p | NFIX |
| AC100803.2 | hsa-miR-744-5p | LDOC1 |
| AC100803.2 | hsa-miR-744-5p | SLC25A27 |
| AC100803.2 | hsa-miR-744-5p | RAB40A |
| AC100803.2 | hsa-miR-744-5p | DES |
| AC100803.2 | hsa-miR-744-5p | GPR20 |
| AC100803.2 | hsa-miR-744-5p | CCDC3 |
| AC100803.2 | hsa-miR-744-5p | PTCHD1 |
| AC100803.2 | hsa-miR-744-5p | IGF2 |
| AC100803.2 | hsa-miR-744-5p | CYS1 |
| AC100803.2 | hsa-miR-744-5p | LGI2 |
| AC100803.2 | hsa-miR-744-5p | CDON |
| AC100803.2 | hsa-miR-744-5p | KCNG1 |
| AC100803.2 | hsa-miR-744-5p | NFASC |
| AC100803.2 | hsa-miR-744-5p | LIMS2 |
| AC100803.2 | hsa-miR-744-5p | MRGPRF |
| AC100803.2 | hsa-miR-744-5p | FAM107A |
| AC100803.2 | hsa-miR-744-5p | TMEM158 |
| AC100803.2 | hsa-miR-744-5p | SORCS2 |
| AC100803.2 | hsa-miR-744-5p | FXYD6 |
| AC100803.2 | hsa-miR-744-5p | IGFBP6 |
| AC100803.2 | hsa-miR-744-5p | NDUFA4L2 |
| AC100803.2 | hsa-miR-744-5p | PPP1R12B |
| KCNMA1-AS1 | hsa-miR-744-5p | ADAM11 |
| KCNMA1-AS1 | hsa-miR-744-5p | HOXA13 |
| KCNMA1-AS1 | hsa-miR-744-5p | GNAZ |
| KCNMA1-AS1 | hsa-miR-744-5p | ASXL3 |
| KCNMA1-AS1 | hsa-miR-744-5p | OSR1 |
| KCNMA1-AS1 | hsa-miR-744-5p | MYL9 |
| KCNMA1-AS1 | hsa-miR-744-5p | MASP1 |
| KCNMA1-AS1 | hsa-miR-744-5p | PRRT2 |
| KCNMA1-AS1 | hsa-miR-744-5p | HSPB3 |
| KCNMA1-AS1 | hsa-miR-744-5p | KCNK3 |
| KCNMA1-AS1 | hsa-miR-744-5p | CPLX1 |
| KCNMA1-AS1 | hsa-miR-744-5p | LPP |
| KCNMA1-AS1 | hsa-miR-744-5p | C11orf87 |
| KCNMA1-AS1 | hsa-miR-744-5p | SYNGR1 |
| KCNMA1-AS1 | hsa-miR-744-5p | C3orf70 |
| KCNMA1-AS1 | hsa-miR-744-5p | PRELP |
| KCNMA1-AS1 | hsa-miR-744-5p | ASIC3 |
| KCNMA1-AS1 | hsa-miR-744-5p | ATP2B2 |
| KCNMA1-AS1 | hsa-miR-744-5p | CACNA1H |
| KCNMA1-AS1 | hsa-miR-744-5p | NFIX |
| KCNMA1-AS1 | hsa-miR-744-5p | LDOC1 |
| KCNMA1-AS1 | hsa-miR-744-5p | SLC25A27 |
| KCNMA1-AS1 | hsa-miR-744-5p | RAB40A |
| KCNMA1-AS1 | hsa-miR-744-5p | DES |
| KCNMA1-AS1 | hsa-miR-744-5p | GPR20 |
| KCNMA1-AS1 | hsa-miR-744-5p | CCDC3 |
| KCNMA1-AS1 | hsa-miR-744-5p | PTCHD1 |
| KCNMA1-AS1 | hsa-miR-744-5p | IGF2 |
| KCNMA1-AS1 | hsa-miR-744-5p | CYS1 |
| KCNMA1-AS1 | hsa-miR-744-5p | LGI2 |
| KCNMA1-AS1 | hsa-miR-744-5p | CDON |
| KCNMA1-AS1 | hsa-miR-744-5p | KCNG1 |
| KCNMA1-AS1 | hsa-miR-744-5p | NFASC |
| KCNMA1-AS1 | hsa-miR-744-5p | LIMS2 |
| KCNMA1-AS1 | hsa-miR-744-5p | MRGPRF |
| KCNMA1-AS1 | hsa-miR-744-5p | FAM107A |
| KCNMA1-AS1 | hsa-miR-744-5p | TMEM158 |
| KCNMA1-AS1 | hsa-miR-744-5p | SORCS2 |
| KCNMA1-AS1 | hsa-miR-744-5p | FXYD6 |
| KCNMA1-AS1 | hsa-miR-744-5p | IGFBP6 |
| KCNMA1-AS1 | hsa-miR-744-5p | NDUFA4L2 |
| KCNMA1-AS1 | hsa-miR-744-5p | PPP1R12B |
| AP000344.1 | hsa-miR-744-5p | ADAM11 |
| AP000344.1 | hsa-miR-744-5p | HOXA13 |
| AP000344.1 | hsa-miR-744-5p | GNAZ |
| AP000344.1 | hsa-miR-744-5p | ASXL3 |
| AP000344.1 | hsa-miR-744-5p | OSR1 |
| AP000344.1 | hsa-miR-744-5p | MYL9 |
| AP000344.1 | hsa-miR-744-5p | MASP1 |
| AP000344.1 | hsa-miR-744-5p | PRRT2 |
| AP000344.1 | hsa-miR-744-5p | HSPB3 |
| AP000344.1 | hsa-miR-744-5p | KCNK3 |
| AP000344.1 | hsa-miR-744-5p | CPLX1 |
| AP000344.1 | hsa-miR-744-5p | LPP |
| AP000344.1 | hsa-miR-744-5p | C11orf87 |
| AP000344.1 | hsa-miR-744-5p | SYNGR1 |
| AP000344.1 | hsa-miR-744-5p | C3orf70 |
| AP000344.1 | hsa-miR-744-5p | PRELP |
| AP000344.1 | hsa-miR-744-5p | ASIC3 |
| AP000344.1 | hsa-miR-744-5p | ATP2B2 |
| AP000344.1 | hsa-miR-744-5p | CACNA1H |
| AP000344.1 | hsa-miR-744-5p | NFIX |
| AP000344.1 | hsa-miR-744-5p | LDOC1 |
| AP000344.1 | hsa-miR-744-5p | SLC25A27 |
| AP000344.1 | hsa-miR-744-5p | RAB40A |
| AP000344.1 | hsa-miR-744-5p | DES |
| AP000344.1 | hsa-miR-744-5p | GPR20 |
| AP000344.1 | hsa-miR-744-5p | CCDC3 |
| AP000344.1 | hsa-miR-744-5p | PTCHD1 |
| AP000344.1 | hsa-miR-744-5p | IGF2 |
| AP000344.1 | hsa-miR-744-5p | CYS1 |
| AP000344.1 | hsa-miR-744-5p | LGI2 |
| AP000344.1 | hsa-miR-744-5p | CDON |
| AP000344.1 | hsa-miR-744-5p | KCNG1 |
| AP000344.1 | hsa-miR-744-5p | NFASC |
| AP000344.1 | hsa-miR-744-5p | LIMS2 |
| AP000344.1 | hsa-miR-744-5p | MRGPRF |
| AP000344.1 | hsa-miR-744-5p | FAM107A |
| AP000344.1 | hsa-miR-744-5p | TMEM158 |
| AP000344.1 | hsa-miR-744-5p | SORCS2 |
| AP000344.1 | hsa-miR-744-5p | FXYD6 |
| AP000344.1 | hsa-miR-744-5p | IGFBP6 |
| AP000344.1 | hsa-miR-744-5p | NDUFA4L2 |
| AP000344.1 | hsa-miR-744-5p | PPP1R12B |
| HRAT92 | hsa-miR-744-5p | ADAM11 |
| HRAT92 | hsa-miR-744-5p | HOXA13 |
| HRAT92 | hsa-miR-744-5p | GNAZ |
| HRAT92 | hsa-miR-744-5p | ASXL3 |
| HRAT92 | hsa-miR-744-5p | OSR1 |
| HRAT92 | hsa-miR-744-5p | MYL9 |
| HRAT92 | hsa-miR-744-5p | MASP1 |
| HRAT92 | hsa-miR-744-5p | PRRT2 |
| HRAT92 | hsa-miR-744-5p | HSPB3 |
| HRAT92 | hsa-miR-744-5p | KCNK3 |
| HRAT92 | hsa-miR-744-5p | CPLX1 |
| HRAT92 | hsa-miR-744-5p | LPP |
| HRAT92 | hsa-miR-744-5p | C11orf87 |
| HRAT92 | hsa-miR-744-5p | SYNGR1 |
| HRAT92 | hsa-miR-744-5p | C3orf70 |
| HRAT92 | hsa-miR-744-5p | PRELP |
| HRAT92 | hsa-miR-744-5p | ASIC3 |
| HRAT92 | hsa-miR-744-5p | ATP2B2 |
| HRAT92 | hsa-miR-744-5p | CACNA1H |
| HRAT92 | hsa-miR-744-5p | NFIX |
| HRAT92 | hsa-miR-744-5p | LDOC1 |
| HRAT92 | hsa-miR-744-5p | SLC25A27 |
| HRAT92 | hsa-miR-744-5p | RAB40A |
| HRAT92 | hsa-miR-744-5p | DES |
| HRAT92 | hsa-miR-744-5p | GPR20 |
| HRAT92 | hsa-miR-744-5p | CCDC3 |
| HRAT92 | hsa-miR-744-5p | PTCHD1 |
| HRAT92 | hsa-miR-744-5p | IGF2 |
| HRAT92 | hsa-miR-744-5p | CYS1 |
| HRAT92 | hsa-miR-744-5p | LGI2 |
| HRAT92 | hsa-miR-744-5p | CDON |
| HRAT92 | hsa-miR-744-5p | KCNG1 |
| HRAT92 | hsa-miR-744-5p | NFASC |
| HRAT92 | hsa-miR-744-5p | LIMS2 |
| HRAT92 | hsa-miR-744-5p | MRGPRF |
| HRAT92 | hsa-miR-744-5p | FAM107A |
| HRAT92 | hsa-miR-744-5p | TMEM158 |
| HRAT92 | hsa-miR-744-5p | SORCS2 |
| HRAT92 | hsa-miR-744-5p | FXYD6 |
| HRAT92 | hsa-miR-744-5p | IGFBP6 |
| HRAT92 | hsa-miR-744-5p | NDUFA4L2 |
| HRAT92 | hsa-miR-744-5p | PPP1R12B |
| DIO3OS | hsa-miR-758-3p | REEP1 |
| DIO3OS | hsa-miR-758-3p | CCDC80 |
| DIO3OS | hsa-miR-758-3p | HLF |
| DIO3OS | hsa-miR-758-3p | RAB9B |
| DIO3OS | hsa-miR-758-3p | NRK |
| DIO3OS | hsa-miR-758-3p | GNAZ |
| DIO3OS | hsa-miR-758-3p | PI15 |
| DIO3OS | hsa-miR-758-3p | OPRK1 |
| DIO3OS | hsa-miR-758-3p | ASXL3 |
| DIO3OS | hsa-miR-758-3p | TRPC1 |
| DIO3OS | hsa-miR-758-3p | KCNK13 |
| DIO3OS | hsa-miR-758-3p | RAB3C |
| DIO3OS | hsa-miR-758-3p | UNC5D |
| DIO3OS | hsa-miR-758-3p | FBXO27 |
| DIO3OS | hsa-miR-758-3p | HFM1 |
| DIO3OS | hsa-miR-758-3p | FAT3 |
| DIO3OS | hsa-miR-758-3p | TMEM132B |
| DIO3OS | hsa-miR-758-3p | HSPB3 |
| DIO3OS | hsa-miR-758-3p | KCNAB1 |
| DIO3OS | hsa-miR-758-3p | VAT1L |
| DIO3OS | hsa-miR-758-3p | MYRIP |
| DIO3OS | hsa-miR-758-3p | KCNK3 |
| DIO3OS | hsa-miR-758-3p | GPR37 |
| DIO3OS | hsa-miR-758-3p | GPR22 |
| DIO3OS | hsa-miR-758-3p | GAL3ST3 |
| DIO3OS | hsa-miR-758-3p | EBF1 |
| DIO3OS | hsa-miR-758-3p | PLAG1 |
| DIO3OS | hsa-miR-758-3p | FIBIN |
| DIO3OS | hsa-miR-758-3p | SYT9 |
| DIO3OS | hsa-miR-758-3p | KIAA1549L |
| DIO3OS | hsa-miR-758-3p | ST6GALNAC3 |
| DIO3OS | hsa-miR-758-3p | WSCD2 |
| DIO3OS | hsa-miR-758-3p | COL4A6 |
| DIO3OS | hsa-miR-758-3p | C3orf70 |
| DIO3OS | hsa-miR-758-3p | TSPAN18 |
| DIO3OS | hsa-miR-758-3p | SLC24A2 |
| DIO3OS | hsa-miR-758-3p | NR3C2 |
| DIO3OS | hsa-miR-758-3p | CAMK2A |
| DIO3OS | hsa-miR-758-3p | ATP2B2 |
| DIO3OS | hsa-miR-758-3p | RSPO3 |
| DIO3OS | hsa-miR-758-3p | DCX |
| DIO3OS | hsa-miR-758-3p | ANKRD36 |
| DIO3OS | hsa-miR-758-3p | PLN |
| DIO3OS | hsa-miR-758-3p | ZNF483 |
| DIO3OS | hsa-miR-758-3p | MAP2 |
| DIO3OS | hsa-miR-758-3p | GPR161 |
| DIO3OS | hsa-miR-758-3p | PBX1 |
| DIO3OS | hsa-miR-758-3p | RBM20 |
| DIO3OS | hsa-miR-758-3p | PTGFR |
| DIO3OS | hsa-miR-758-3p | LRRC1 |
| DIO3OS | hsa-miR-758-3p | SORBS1 |
| DIO3OS | hsa-miR-758-3p | SLC25A27 |
| DIO3OS | hsa-miR-758-3p | CHRDL1 |
| DIO3OS | hsa-miR-758-3p | IRS4 |
| DIO3OS | hsa-miR-758-3p | NUDT13 |
| DIO3OS | hsa-miR-758-3p | PRKG1 |
| DIO3OS | hsa-miR-758-3p | PKHD1L1 |
| DIO3OS | hsa-miR-758-3p | ARHGAP6 |
| DIO3OS | hsa-miR-758-3p | IGF2 |
| DIO3OS | hsa-miR-758-3p | LGI2 |
| DIO3OS | hsa-miR-758-3p | SEMA5A |
| DIO3OS | hsa-miR-758-3p | CDON |
| DIO3OS | hsa-miR-758-3p | NT5DC3 |
| DIO3OS | hsa-miR-758-3p | FHL1 |
| DIO3OS | hsa-miR-758-3p | FAM13A |
| DIO3OS | hsa-miR-758-3p | MAPK10 |
| DIO3OS | hsa-miR-758-3p | CDKL1 |
| DIO3OS | hsa-miR-758-3p | ST8SIA1 |
| DIO3OS | hsa-miR-758-3p | RIC3 |
| DIO3OS | hsa-miR-758-3p | MEIS2 |
| DIO3OS | hsa-miR-758-3p | NFASC |
| DIO3OS | hsa-miR-758-3p | ADAMTSL5 |
| DIO3OS | hsa-miR-758-3p | MRVI1 |
| DIO3OS | hsa-miR-758-3p | SYNPO2 |
| DIO3OS | hsa-miR-758-3p | OSR2 |
| DIO3OS | hsa-miR-758-3p | GFRA1 |
| DIO3OS | hsa-miR-758-3p | MRGPRF |
| DIO3OS | hsa-miR-758-3p | SHISA6 |
| DIO3OS | hsa-miR-758-3p | FAM107A |
| DIO3OS | hsa-miR-758-3p | KLF8 |
| DIO3OS | hsa-miR-758-3p | EPHA3 |
| DIO3OS | hsa-miR-758-3p | SLIT3 |
| DIO3OS | hsa-miR-758-3p | NAALAD2 |
| DIO3OS | hsa-miR-758-3p | FHL5 |
| DIO3OS | hsa-miR-758-3p | RBFOX3 |
| AF001548.3 | hsa-miR-433-3p | FSTL3 |
| AF001548.3 | hsa-miR-433-3p | CCDC80 |
| AF001548.3 | hsa-miR-433-3p | HOXA13 |
| AF001548.3 | hsa-miR-433-3p | FILIP1 |
| AF001548.3 | hsa-miR-433-3p | GATA5 |
| AF001548.3 | hsa-miR-433-3p | PI15 |
| AF001548.3 | hsa-miR-433-3p | ABCC9 |
| AF001548.3 | hsa-miR-433-3p | ASPA |
| AF001548.3 | hsa-miR-433-3p | KCNE4 |
| AF001548.3 | hsa-miR-433-3p | RAB3C |
| AF001548.3 | hsa-miR-433-3p | PDLIM3 |
| AF001548.3 | hsa-miR-433-3p | UNC5D |
| AF001548.3 | hsa-miR-433-3p | ACTC1 |
| AF001548.3 | hsa-miR-433-3p | TCF23 |
| AF001548.3 | hsa-miR-433-3p | EMCN |
| AF001548.3 | hsa-miR-433-3p | NPY1R |
| AF001548.3 | hsa-miR-433-3p | FAT3 |
| AF001548.3 | hsa-miR-433-3p | TMEM132B |
| AF001548.3 | hsa-miR-433-3p | GPR37 |
| AF001548.3 | hsa-miR-433-3p | GPR22 |
| AF001548.3 | hsa-miR-433-3p | LPP |
| AF001548.3 | hsa-miR-433-3p | IL20RA |
| AF001548.3 | hsa-miR-433-3p | KIAA1549L |
| AF001548.3 | hsa-miR-433-3p | DMRTA1 |
| AF001548.3 | hsa-miR-433-3p | ST6GALNAC3 |
| AF001548.3 | hsa-miR-433-3p | WSCD2 |
| AF001548.3 | hsa-miR-433-3p | C3orf70 |
| AF001548.3 | hsa-miR-433-3p | TSPAN18 |
| AF001548.3 | hsa-miR-433-3p | PRELP |
| AF001548.3 | hsa-miR-433-3p | RSPO3 |
| AF001548.3 | hsa-miR-433-3p | PLN |
| AF001548.3 | hsa-miR-433-3p | MFAP5 |
| AF001548.3 | hsa-miR-433-3p | MAP2 |
| AF001548.3 | hsa-miR-433-3p | FBXL22 |
| AF001548.3 | hsa-miR-433-3p | MYLK |
| AF001548.3 | hsa-miR-433-3p | DNM3 |
| AF001548.3 | hsa-miR-433-3p | GPR161 |
| AF001548.3 | hsa-miR-433-3p | PBX1 |
| AF001548.3 | hsa-miR-433-3p | SYPL2 |
| AF001548.3 | hsa-miR-433-3p | CDR1 |
| AF001548.3 | hsa-miR-433-3p | PTGER3 |
| AF001548.3 | hsa-miR-433-3p | SORBS1 |
| AF001548.3 | hsa-miR-433-3p | KCNB1 |
| AF001548.3 | hsa-miR-433-3p | FOXO6 |
| AF001548.3 | hsa-miR-433-3p | RAB40A |
| AF001548.3 | hsa-miR-433-3p | TRPM3 |
| AF001548.3 | hsa-miR-433-3p | MAMDC2 |
| AF001548.3 | hsa-miR-433-3p | HIF3A |
| AF001548.3 | hsa-miR-433-3p | PKHD1L1 |
| AF001548.3 | hsa-miR-433-3p | BNC2 |
| AF001548.3 | hsa-miR-433-3p | SLC22A3 |
| AF001548.3 | hsa-miR-433-3p | CDON |
| AF001548.3 | hsa-miR-433-3p | NT5DC3 |
| AF001548.3 | hsa-miR-433-3p | SPOCK1 |
| AF001548.3 | hsa-miR-433-3p | CDKL1 |
| AF001548.3 | hsa-miR-433-3p | MYL3 |
| AF001548.3 | hsa-miR-433-3p | ST8SIA1 |
| AF001548.3 | hsa-miR-433-3p | RIC3 |
| AF001548.3 | hsa-miR-433-3p | MEIS2 |
| AF001548.3 | hsa-miR-433-3p | MTMR11 |
| AF001548.3 | hsa-miR-433-3p | SYNC |
| AF001548.3 | hsa-miR-433-3p | ACTG2 |
| AF001548.3 | hsa-miR-433-3p | ADAMTSL5 |
| AF001548.3 | hsa-miR-433-3p | SEPT4 |
| AF001548.3 | hsa-miR-433-3p | SYNPO2 |
| AF001548.3 | hsa-miR-433-3p | GFRA1 |
| AF001548.3 | hsa-miR-433-3p | MRGPRF |
| AF001548.3 | hsa-miR-433-3p | KLF8 |
| AF001548.3 | hsa-miR-433-3p | PCOLCE2 |
| AF001548.3 | hsa-miR-433-3p | SLC15A2 |
| AF001548.3 | hsa-miR-433-3p | LRRC31 |
| AF001548.3 | hsa-miR-433-3p | NAALAD2 |
| AF001548.3 | hsa-miR-433-3p | PIANP |
| AF001548.3 | hsa-miR-433-3p | PHEX |
| AF001548.3 | hsa-miR-433-3p | FHL5 |
| AF001548.3 | hsa-miR-433-3p | TRERF1 |
| AF001548.3 | hsa-miR-433-3p | CDH13 |
| AF001548.3 | hsa-miR-433-3p | RBFOX3 |
| AF001548.3 | hsa-miR-433-3p | CCDC68 |
| AF001548.3 | hsa-miR-433-3p | PPP1R12B |
| TRHDE-AS1 | hsa-miR-433-3p | FSTL3 |
| TRHDE-AS1 | hsa-miR-433-3p | CCDC80 |
| TRHDE-AS1 | hsa-miR-433-3p | HOXA13 |
| TRHDE-AS1 | hsa-miR-433-3p | FILIP1 |
| TRHDE-AS1 | hsa-miR-433-3p | GATA5 |
| TRHDE-AS1 | hsa-miR-433-3p | PI15 |
| TRHDE-AS1 | hsa-miR-433-3p | ABCC9 |
| TRHDE-AS1 | hsa-miR-433-3p | ASPA |
| TRHDE-AS1 | hsa-miR-433-3p | KCNE4 |
| TRHDE-AS1 | hsa-miR-433-3p | RAB3C |
| TRHDE-AS1 | hsa-miR-433-3p | PDLIM3 |
| TRHDE-AS1 | hsa-miR-433-3p | UNC5D |
| TRHDE-AS1 | hsa-miR-433-3p | ACTC1 |
| TRHDE-AS1 | hsa-miR-433-3p | TCF23 |
| TRHDE-AS1 | hsa-miR-433-3p | EMCN |
| TRHDE-AS1 | hsa-miR-433-3p | NPY1R |
| TRHDE-AS1 | hsa-miR-433-3p | FAT3 |
| TRHDE-AS1 | hsa-miR-433-3p | TMEM132B |
| TRHDE-AS1 | hsa-miR-433-3p | GPR37 |
| TRHDE-AS1 | hsa-miR-433-3p | GPR22 |
| TRHDE-AS1 | hsa-miR-433-3p | LPP |
| TRHDE-AS1 | hsa-miR-433-3p | IL20RA |
| TRHDE-AS1 | hsa-miR-433-3p | KIAA1549L |
| TRHDE-AS1 | hsa-miR-433-3p | DMRTA1 |
| TRHDE-AS1 | hsa-miR-433-3p | ST6GALNAC3 |
| TRHDE-AS1 | hsa-miR-433-3p | WSCD2 |
| TRHDE-AS1 | hsa-miR-433-3p | C3orf70 |
| TRHDE-AS1 | hsa-miR-433-3p | TSPAN18 |
| TRHDE-AS1 | hsa-miR-433-3p | PRELP |
| TRHDE-AS1 | hsa-miR-433-3p | RSPO3 |
| TRHDE-AS1 | hsa-miR-433-3p | PLN |
| TRHDE-AS1 | hsa-miR-433-3p | MFAP5 |
| TRHDE-AS1 | hsa-miR-433-3p | MAP2 |
| TRHDE-AS1 | hsa-miR-433-3p | FBXL22 |
| TRHDE-AS1 | hsa-miR-433-3p | MYLK |
| TRHDE-AS1 | hsa-miR-433-3p | DNM3 |
| TRHDE-AS1 | hsa-miR-433-3p | GPR161 |
| TRHDE-AS1 | hsa-miR-433-3p | PBX1 |
| TRHDE-AS1 | hsa-miR-433-3p | SYPL2 |
| TRHDE-AS1 | hsa-miR-433-3p | CDR1 |
| TRHDE-AS1 | hsa-miR-433-3p | PTGER3 |
| TRHDE-AS1 | hsa-miR-433-3p | SORBS1 |
| TRHDE-AS1 | hsa-miR-433-3p | KCNB1 |
| TRHDE-AS1 | hsa-miR-433-3p | FOXO6 |
| TRHDE-AS1 | hsa-miR-433-3p | RAB40A |
| TRHDE-AS1 | hsa-miR-433-3p | TRPM3 |
| TRHDE-AS1 | hsa-miR-433-3p | MAMDC2 |
| TRHDE-AS1 | hsa-miR-433-3p | HIF3A |
| TRHDE-AS1 | hsa-miR-433-3p | PKHD1L1 |
| TRHDE-AS1 | hsa-miR-433-3p | BNC2 |
| TRHDE-AS1 | hsa-miR-433-3p | SLC22A3 |
| TRHDE-AS1 | hsa-miR-433-3p | CDON |
| TRHDE-AS1 | hsa-miR-433-3p | NT5DC3 |
| TRHDE-AS1 | hsa-miR-433-3p | SPOCK1 |
| TRHDE-AS1 | hsa-miR-433-3p | CDKL1 |
| TRHDE-AS1 | hsa-miR-433-3p | MYL3 |
| TRHDE-AS1 | hsa-miR-433-3p | ST8SIA1 |
| TRHDE-AS1 | hsa-miR-433-3p | RIC3 |
| TRHDE-AS1 | hsa-miR-433-3p | MEIS2 |
| TRHDE-AS1 | hsa-miR-433-3p | MTMR11 |
| TRHDE-AS1 | hsa-miR-433-3p | SYNC |
| TRHDE-AS1 | hsa-miR-433-3p | ACTG2 |
| TRHDE-AS1 | hsa-miR-433-3p | ADAMTSL5 |
| TRHDE-AS1 | hsa-miR-433-3p | SEPT4 |
| TRHDE-AS1 | hsa-miR-433-3p | SYNPO2 |
| TRHDE-AS1 | hsa-miR-433-3p | GFRA1 |
| TRHDE-AS1 | hsa-miR-433-3p | MRGPRF |
| TRHDE-AS1 | hsa-miR-433-3p | KLF8 |
| TRHDE-AS1 | hsa-miR-433-3p | PCOLCE2 |
| TRHDE-AS1 | hsa-miR-433-3p | SLC15A2 |
| TRHDE-AS1 | hsa-miR-433-3p | LRRC31 |
| TRHDE-AS1 | hsa-miR-433-3p | NAALAD2 |
| TRHDE-AS1 | hsa-miR-433-3p | PIANP |
| TRHDE-AS1 | hsa-miR-433-3p | PHEX |
| TRHDE-AS1 | hsa-miR-433-3p | FHL5 |
| TRHDE-AS1 | hsa-miR-433-3p | TRERF1 |
| TRHDE-AS1 | hsa-miR-433-3p | CDH13 |
| TRHDE-AS1 | hsa-miR-433-3p | RBFOX3 |
| TRHDE-AS1 | hsa-miR-433-3p | CCDC68 |
| TRHDE-AS1 | hsa-miR-433-3p | PPP1R12B |
| ADIRF-AS1 | hsa-miR-326 | INMT |
| ADIRF-AS1 | hsa-miR-326 | TLL1 |
| ADIRF-AS1 | hsa-miR-326 | REEP1 |
| ADIRF-AS1 | hsa-miR-326 | CCDC80 |
| ADIRF-AS1 | hsa-miR-326 | HLF |
| ADIRF-AS1 | hsa-miR-326 | IGFBP5 |
| ADIRF-AS1 | hsa-miR-326 | SLC5A9 |
| ADIRF-AS1 | hsa-miR-326 | NRK |
| ADIRF-AS1 | hsa-miR-326 | PTGIS |
| ADIRF-AS1 | hsa-miR-326 | ATP1B2 |
| ADIRF-AS1 | hsa-miR-326 | CNN1 |
| ADIRF-AS1 | hsa-miR-326 | GATA5 |
| ADIRF-AS1 | hsa-miR-326 | RERG |
| ADIRF-AS1 | hsa-miR-326 | ITGA7 |
| ADIRF-AS1 | hsa-miR-326 | ABCC9 |
| ADIRF-AS1 | hsa-miR-326 | SELP |
| ADIRF-AS1 | hsa-miR-326 | NGEF |
| ADIRF-AS1 | hsa-miR-326 | PDE8B |
| ADIRF-AS1 | hsa-miR-326 | ASXL3 |
| ADIRF-AS1 | hsa-miR-326 | SLC4A3 |
| ADIRF-AS1 | hsa-miR-326 | SLC2A12 |
| ADIRF-AS1 | hsa-miR-326 | MYL9 |
| ADIRF-AS1 | hsa-miR-326 | RAB3C |
| ADIRF-AS1 | hsa-miR-326 | FBXO27 |
| ADIRF-AS1 | hsa-miR-326 | HFM1 |
| ADIRF-AS1 | hsa-miR-326 | KCNIP3 |
| ADIRF-AS1 | hsa-miR-326 | TCF23 |
| ADIRF-AS1 | hsa-miR-326 | MASP1 |
| ADIRF-AS1 | hsa-miR-326 | HPGD |
| ADIRF-AS1 | hsa-miR-326 | FAT3 |
| ADIRF-AS1 | hsa-miR-326 | TMEM132B |
| ADIRF-AS1 | hsa-miR-326 | HSPB3 |
| ADIRF-AS1 | hsa-miR-326 | MYRIP |
| ADIRF-AS1 | hsa-miR-326 | KCNK3 |
| ADIRF-AS1 | hsa-miR-326 | TUB |
| ADIRF-AS1 | hsa-miR-326 | LPP |
| ADIRF-AS1 | hsa-miR-326 | C7 |
| ADIRF-AS1 | hsa-miR-326 | EBF1 |
| ADIRF-AS1 | hsa-miR-326 | BVES |
| ADIRF-AS1 | hsa-miR-326 | GPR88 |
| ADIRF-AS1 | hsa-miR-326 | CLU |
| ADIRF-AS1 | hsa-miR-326 | SLC2A4 |
| ADIRF-AS1 | hsa-miR-326 | FIBIN |
| ADIRF-AS1 | hsa-miR-326 | SYT9 |
| ADIRF-AS1 | hsa-miR-326 | KIAA1549L |
| ADIRF-AS1 | hsa-miR-326 | GDNF |
| ADIRF-AS1 | hsa-miR-326 | TNFAIP8L3 |
| ADIRF-AS1 | hsa-miR-326 | GRID1 |
| ADIRF-AS1 | hsa-miR-326 | ST6GALNAC3 |
| ADIRF-AS1 | hsa-miR-326 | COL4A6 |
| ADIRF-AS1 | hsa-miR-326 | TSPAN18 |
| ADIRF-AS1 | hsa-miR-326 | PRELP |
| ADIRF-AS1 | hsa-miR-326 | FLNC |
| ADIRF-AS1 | hsa-miR-326 | CAMK2A |
| ADIRF-AS1 | hsa-miR-326 | SNCG |
| ADIRF-AS1 | hsa-miR-326 | ATP2B2 |
| ADIRF-AS1 | hsa-miR-326 | SMOC2 |
| ADIRF-AS1 | hsa-miR-326 | ADAM33 |
| ADIRF-AS1 | hsa-miR-326 | SUSD2 |
| ADIRF-AS1 | hsa-miR-326 | CACNA1H |
| ADIRF-AS1 | hsa-miR-326 | NFIX |
| ADIRF-AS1 | hsa-miR-326 | MYLK |
| ADIRF-AS1 | hsa-miR-326 | LMOD1 |
| ADIRF-AS1 | hsa-miR-326 | DNM3 |
| ADIRF-AS1 | hsa-miR-326 | GPR161 |
| ADIRF-AS1 | hsa-miR-326 | PBX1 |
| ADIRF-AS1 | hsa-miR-326 | RBM20 |
| ADIRF-AS1 | hsa-miR-326 | SYPL2 |
| ADIRF-AS1 | hsa-miR-326 | LDOC1 |
| ADIRF-AS1 | hsa-miR-326 | CDR1 |
| ADIRF-AS1 | hsa-miR-326 | BEND6 |
| ADIRF-AS1 | hsa-miR-326 | PTGFR |
| ADIRF-AS1 | hsa-miR-326 | APCDD1L |
| ADIRF-AS1 | hsa-miR-326 | KCNB1 |
| ADIRF-AS1 | hsa-miR-326 | CHRDL1 |
| ADIRF-AS1 | hsa-miR-326 | HYI |
| ADIRF-AS1 | hsa-miR-326 | WISP2 |
| ADIRF-AS1 | hsa-miR-326 | JPH2 |
| ADIRF-AS1 | hsa-miR-326 | DES |
| ADIRF-AS1 | hsa-miR-326 | PRKG1 |
| ADIRF-AS1 | hsa-miR-326 | TRPM3 |
| ADIRF-AS1 | hsa-miR-326 | GPR20 |
| ADIRF-AS1 | hsa-miR-326 | PKHD1L1 |
| ADIRF-AS1 | hsa-miR-326 | CCDC3 |
| ADIRF-AS1 | hsa-miR-326 | PTCHD1 |
| ADIRF-AS1 | hsa-miR-326 | ADRA1D |
| ADIRF-AS1 | hsa-miR-326 | DCLK1 |
| ADIRF-AS1 | hsa-miR-326 | BNC2 |
| ADIRF-AS1 | hsa-miR-326 | SMOC1 |
| ADIRF-AS1 | hsa-miR-326 | CYS1 |
| ADIRF-AS1 | hsa-miR-326 | LGI2 |
| ADIRF-AS1 | hsa-miR-326 | SEMA5A |
| ADIRF-AS1 | hsa-miR-326 | CDON |
| ADIRF-AS1 | hsa-miR-326 | PRIMA1 |
| ADIRF-AS1 | hsa-miR-326 | NOX4 |
| ADIRF-AS1 | hsa-miR-326 | SPOCK1 |
| ADIRF-AS1 | hsa-miR-326 | FAM13A |
| ADIRF-AS1 | hsa-miR-326 | MAPK10 |
| ADIRF-AS1 | hsa-miR-326 | ST8SIA1 |
| ADIRF-AS1 | hsa-miR-326 | RIC3 |
| ADIRF-AS1 | hsa-miR-326 | ZNF208 |
| ADIRF-AS1 | hsa-miR-326 | SRL |
| ADIRF-AS1 | hsa-miR-326 | NFASC |
| ADIRF-AS1 | hsa-miR-326 | DGKB |
| ADIRF-AS1 | hsa-miR-326 | SYNC |
| ADIRF-AS1 | hsa-miR-326 | LIMS2 |
| ADIRF-AS1 | hsa-miR-326 | ITGA11 |
| ADIRF-AS1 | hsa-miR-326 | SEPT4 |
| ADIRF-AS1 | hsa-miR-326 | GFRA1 |
| ADIRF-AS1 | hsa-miR-326 | MRGPRF |
| ADIRF-AS1 | hsa-miR-326 | SHISA6 |
| ADIRF-AS1 | hsa-miR-326 | TCEA3 |
| ADIRF-AS1 | hsa-miR-326 | PLK5 |
| ADIRF-AS1 | hsa-miR-326 | MUC15 |
| ADIRF-AS1 | hsa-miR-326 | FAM107A |
| ADIRF-AS1 | hsa-miR-326 | SLC15A2 |
| ADIRF-AS1 | hsa-miR-326 | TMEM158 |
| ADIRF-AS1 | hsa-miR-326 | SORCS2 |
| ADIRF-AS1 | hsa-miR-326 | SLIT3 |
| ADIRF-AS1 | hsa-miR-326 | PHEX |
| ADIRF-AS1 | hsa-miR-326 | FHL5 |
| ADIRF-AS1 | hsa-miR-326 | LDB3 |
| ADIRF-AS1 | hsa-miR-326 | AHNAK2 |
| ADIRF-AS1 | hsa-miR-326 | DET1 |
| ADIRF-AS1 | hsa-miR-326 | AOC3 |
| ADIRF-AS1 | hsa-miR-326 | PPP1R12B |
| AC100803.2 | hsa-miR-326 | INMT |
| AC100803.2 | hsa-miR-326 | TLL1 |
| AC100803.2 | hsa-miR-326 | REEP1 |
| AC100803.2 | hsa-miR-326 | CCDC80 |
| AC100803.2 | hsa-miR-326 | HLF |
| AC100803.2 | hsa-miR-326 | IGFBP5 |
| AC100803.2 | hsa-miR-326 | SLC5A9 |
| AC100803.2 | hsa-miR-326 | NRK |
| AC100803.2 | hsa-miR-326 | PTGIS |
| AC100803.2 | hsa-miR-326 | ATP1B2 |
| AC100803.2 | hsa-miR-326 | CNN1 |
| AC100803.2 | hsa-miR-326 | GATA5 |
| AC100803.2 | hsa-miR-326 | RERG |
| AC100803.2 | hsa-miR-326 | ITGA7 |
| AC100803.2 | hsa-miR-326 | ABCC9 |
| AC100803.2 | hsa-miR-326 | SELP |
| AC100803.2 | hsa-miR-326 | NGEF |
| AC100803.2 | hsa-miR-326 | PDE8B |
| AC100803.2 | hsa-miR-326 | ASXL3 |
| AC100803.2 | hsa-miR-326 | SLC4A3 |
| AC100803.2 | hsa-miR-326 | SLC2A12 |
| AC100803.2 | hsa-miR-326 | MYL9 |
| AC100803.2 | hsa-miR-326 | RAB3C |
| AC100803.2 | hsa-miR-326 | FBXO27 |
| AC100803.2 | hsa-miR-326 | HFM1 |
| AC100803.2 | hsa-miR-326 | KCNIP3 |
| AC100803.2 | hsa-miR-326 | TCF23 |
| AC100803.2 | hsa-miR-326 | MASP1 |
| AC100803.2 | hsa-miR-326 | HPGD |
| AC100803.2 | hsa-miR-326 | FAT3 |
| AC100803.2 | hsa-miR-326 | TMEM132B |
| AC100803.2 | hsa-miR-326 | HSPB3 |
| AC100803.2 | hsa-miR-326 | MYRIP |
| AC100803.2 | hsa-miR-326 | KCNK3 |
| AC100803.2 | hsa-miR-326 | TUB |
| AC100803.2 | hsa-miR-326 | LPP |
| AC100803.2 | hsa-miR-326 | C7 |
| AC100803.2 | hsa-miR-326 | EBF1 |
| AC100803.2 | hsa-miR-326 | BVES |
| AC100803.2 | hsa-miR-326 | GPR88 |
| AC100803.2 | hsa-miR-326 | CLU |
| AC100803.2 | hsa-miR-326 | SLC2A4 |
| AC100803.2 | hsa-miR-326 | FIBIN |
| AC100803.2 | hsa-miR-326 | SYT9 |
| AC100803.2 | hsa-miR-326 | KIAA1549L |
| AC100803.2 | hsa-miR-326 | GDNF |
| AC100803.2 | hsa-miR-326 | TNFAIP8L3 |
| AC100803.2 | hsa-miR-326 | GRID1 |
| AC100803.2 | hsa-miR-326 | ST6GALNAC3 |
| AC100803.2 | hsa-miR-326 | COL4A6 |
| AC100803.2 | hsa-miR-326 | TSPAN18 |
| AC100803.2 | hsa-miR-326 | PRELP |
| AC100803.2 | hsa-miR-326 | FLNC |
| AC100803.2 | hsa-miR-326 | CAMK2A |
| AC100803.2 | hsa-miR-326 | SNCG |
| AC100803.2 | hsa-miR-326 | ATP2B2 |
| AC100803.2 | hsa-miR-326 | SMOC2 |
| AC100803.2 | hsa-miR-326 | ADAM33 |
| AC100803.2 | hsa-miR-326 | SUSD2 |
| AC100803.2 | hsa-miR-326 | CACNA1H |
| AC100803.2 | hsa-miR-326 | NFIX |
| AC100803.2 | hsa-miR-326 | MYLK |
| AC100803.2 | hsa-miR-326 | LMOD1 |
| AC100803.2 | hsa-miR-326 | DNM3 |
| AC100803.2 | hsa-miR-326 | GPR161 |
| AC100803.2 | hsa-miR-326 | PBX1 |
| AC100803.2 | hsa-miR-326 | RBM20 |
| AC100803.2 | hsa-miR-326 | SYPL2 |
| AC100803.2 | hsa-miR-326 | LDOC1 |
| AC100803.2 | hsa-miR-326 | CDR1 |
| AC100803.2 | hsa-miR-326 | BEND6 |
| AC100803.2 | hsa-miR-326 | PTGFR |
| AC100803.2 | hsa-miR-326 | APCDD1L |
| AC100803.2 | hsa-miR-326 | KCNB1 |
| AC100803.2 | hsa-miR-326 | CHRDL1 |
| AC100803.2 | hsa-miR-326 | HYI |
| AC100803.2 | hsa-miR-326 | WISP2 |
| AC100803.2 | hsa-miR-326 | JPH2 |
| AC100803.2 | hsa-miR-326 | DES |
| AC100803.2 | hsa-miR-326 | PRKG1 |
| AC100803.2 | hsa-miR-326 | TRPM3 |
| AC100803.2 | hsa-miR-326 | GPR20 |
| AC100803.2 | hsa-miR-326 | PKHD1L1 |
| AC100803.2 | hsa-miR-326 | CCDC3 |
| AC100803.2 | hsa-miR-326 | PTCHD1 |
| AC100803.2 | hsa-miR-326 | ADRA1D |
| AC100803.2 | hsa-miR-326 | DCLK1 |
| AC100803.2 | hsa-miR-326 | BNC2 |
| AC100803.2 | hsa-miR-326 | SMOC1 |
| AC100803.2 | hsa-miR-326 | CYS1 |
| AC100803.2 | hsa-miR-326 | LGI2 |
| AC100803.2 | hsa-miR-326 | SEMA5A |
| AC100803.2 | hsa-miR-326 | CDON |
| AC100803.2 | hsa-miR-326 | PRIMA1 |
| AC100803.2 | hsa-miR-326 | NOX4 |
| AC100803.2 | hsa-miR-326 | SPOCK1 |
| AC100803.2 | hsa-miR-326 | FAM13A |
| AC100803.2 | hsa-miR-326 | MAPK10 |
| AC100803.2 | hsa-miR-326 | ST8SIA1 |
| AC100803.2 | hsa-miR-326 | RIC3 |
| AC100803.2 | hsa-miR-326 | ZNF208 |
| AC100803.2 | hsa-miR-326 | SRL |
| AC100803.2 | hsa-miR-326 | NFASC |
| AC100803.2 | hsa-miR-326 | DGKB |
| AC100803.2 | hsa-miR-326 | SYNC |
| AC100803.2 | hsa-miR-326 | LIMS2 |
| AC100803.2 | hsa-miR-326 | ITGA11 |
| AC100803.2 | hsa-miR-326 | SEPT4 |
| AC100803.2 | hsa-miR-326 | GFRA1 |
| AC100803.2 | hsa-miR-326 | MRGPRF |
| AC100803.2 | hsa-miR-326 | SHISA6 |
| AC100803.2 | hsa-miR-326 | TCEA3 |
| AC100803.2 | hsa-miR-326 | PLK5 |
| AC100803.2 | hsa-miR-326 | MUC15 |
| AC100803.2 | hsa-miR-326 | FAM107A |
| AC100803.2 | hsa-miR-326 | SLC15A2 |
| AC100803.2 | hsa-miR-326 | TMEM158 |
| AC100803.2 | hsa-miR-326 | SORCS2 |
| AC100803.2 | hsa-miR-326 | SLIT3 |
| AC100803.2 | hsa-miR-326 | PHEX |
| AC100803.2 | hsa-miR-326 | FHL5 |
| AC100803.2 | hsa-miR-326 | LDB3 |
| AC100803.2 | hsa-miR-326 | AHNAK2 |
| AC100803.2 | hsa-miR-326 | DET1 |
| AC100803.2 | hsa-miR-326 | AOC3 |
| AC100803.2 | hsa-miR-326 | PPP1R12B |
| AP003355.2 | hsa-miR-326 | INMT |
| AP003355.2 | hsa-miR-326 | TLL1 |
| AP003355.2 | hsa-miR-326 | REEP1 |
| AP003355.2 | hsa-miR-326 | CCDC80 |
| AP003355.2 | hsa-miR-326 | HLF |
| AP003355.2 | hsa-miR-326 | IGFBP5 |
| AP003355.2 | hsa-miR-326 | SLC5A9 |
| AP003355.2 | hsa-miR-326 | NRK |
| AP003355.2 | hsa-miR-326 | PTGIS |
| AP003355.2 | hsa-miR-326 | ATP1B2 |
| AP003355.2 | hsa-miR-326 | CNN1 |
| AP003355.2 | hsa-miR-326 | GATA5 |
| AP003355.2 | hsa-miR-326 | RERG |
| AP003355.2 | hsa-miR-326 | ITGA7 |
| AP003355.2 | hsa-miR-326 | ABCC9 |
| AP003355.2 | hsa-miR-326 | SELP |
| AP003355.2 | hsa-miR-326 | NGEF |
| AP003355.2 | hsa-miR-326 | PDE8B |
| AP003355.2 | hsa-miR-326 | ASXL3 |
| AP003355.2 | hsa-miR-326 | SLC4A3 |
| AP003355.2 | hsa-miR-326 | SLC2A12 |
| AP003355.2 | hsa-miR-326 | MYL9 |
| AP003355.2 | hsa-miR-326 | RAB3C |
| AP003355.2 | hsa-miR-326 | FBXO27 |
| AP003355.2 | hsa-miR-326 | HFM1 |
| AP003355.2 | hsa-miR-326 | KCNIP3 |
| AP003355.2 | hsa-miR-326 | TCF23 |
| AP003355.2 | hsa-miR-326 | MASP1 |
| AP003355.2 | hsa-miR-326 | HPGD |
| AP003355.2 | hsa-miR-326 | FAT3 |
| AP003355.2 | hsa-miR-326 | TMEM132B |
| AP003355.2 | hsa-miR-326 | HSPB3 |
| AP003355.2 | hsa-miR-326 | MYRIP |
| AP003355.2 | hsa-miR-326 | KCNK3 |
| AP003355.2 | hsa-miR-326 | TUB |
| AP003355.2 | hsa-miR-326 | LPP |
| AP003355.2 | hsa-miR-326 | C7 |
| AP003355.2 | hsa-miR-326 | EBF1 |
| AP003355.2 | hsa-miR-326 | BVES |
| AP003355.2 | hsa-miR-326 | GPR88 |
| AP003355.2 | hsa-miR-326 | CLU |
| AP003355.2 | hsa-miR-326 | SLC2A4 |
| AP003355.2 | hsa-miR-326 | FIBIN |
| AP003355.2 | hsa-miR-326 | SYT9 |
| AP003355.2 | hsa-miR-326 | KIAA1549L |
| AP003355.2 | hsa-miR-326 | GDNF |
| AP003355.2 | hsa-miR-326 | TNFAIP8L3 |
| AP003355.2 | hsa-miR-326 | GRID1 |
| AP003355.2 | hsa-miR-326 | ST6GALNAC3 |
| AP003355.2 | hsa-miR-326 | COL4A6 |
| AP003355.2 | hsa-miR-326 | TSPAN18 |
| AP003355.2 | hsa-miR-326 | PRELP |
| AP003355.2 | hsa-miR-326 | FLNC |
| AP003355.2 | hsa-miR-326 | CAMK2A |
| AP003355.2 | hsa-miR-326 | SNCG |
| AP003355.2 | hsa-miR-326 | ATP2B2 |
| AP003355.2 | hsa-miR-326 | SMOC2 |
| AP003355.2 | hsa-miR-326 | ADAM33 |
| AP003355.2 | hsa-miR-326 | SUSD2 |
| AP003355.2 | hsa-miR-326 | CACNA1H |
| AP003355.2 | hsa-miR-326 | NFIX |
| AP003355.2 | hsa-miR-326 | MYLK |
| AP003355.2 | hsa-miR-326 | LMOD1 |
| AP003355.2 | hsa-miR-326 | DNM3 |
| AP003355.2 | hsa-miR-326 | GPR161 |
| AP003355.2 | hsa-miR-326 | PBX1 |
| AP003355.2 | hsa-miR-326 | RBM20 |
| AP003355.2 | hsa-miR-326 | SYPL2 |
| AP003355.2 | hsa-miR-326 | LDOC1 |
| AP003355.2 | hsa-miR-326 | CDR1 |
| AP003355.2 | hsa-miR-326 | BEND6 |
| AP003355.2 | hsa-miR-326 | PTGFR |
| AP003355.2 | hsa-miR-326 | APCDD1L |
| AP003355.2 | hsa-miR-326 | KCNB1 |
| AP003355.2 | hsa-miR-326 | CHRDL1 |
| AP003355.2 | hsa-miR-326 | HYI |
| AP003355.2 | hsa-miR-326 | WISP2 |
| AP003355.2 | hsa-miR-326 | JPH2 |
| AP003355.2 | hsa-miR-326 | DES |
| AP003355.2 | hsa-miR-326 | PRKG1 |
| AP003355.2 | hsa-miR-326 | TRPM3 |
| AP003355.2 | hsa-miR-326 | GPR20 |
| AP003355.2 | hsa-miR-326 | PKHD1L1 |
| AP003355.2 | hsa-miR-326 | CCDC3 |
| AP003355.2 | hsa-miR-326 | PTCHD1 |
| AP003355.2 | hsa-miR-326 | ADRA1D |
| AP003355.2 | hsa-miR-326 | DCLK1 |
| AP003355.2 | hsa-miR-326 | BNC2 |
| AP003355.2 | hsa-miR-326 | SMOC1 |
| AP003355.2 | hsa-miR-326 | CYS1 |
| AP003355.2 | hsa-miR-326 | LGI2 |
| AP003355.2 | hsa-miR-326 | SEMA5A |
| AP003355.2 | hsa-miR-326 | CDON |
| AP003355.2 | hsa-miR-326 | PRIMA1 |
| AP003355.2 | hsa-miR-326 | NOX4 |
| AP003355.2 | hsa-miR-326 | SPOCK1 |
| AP003355.2 | hsa-miR-326 | FAM13A |
| AP003355.2 | hsa-miR-326 | MAPK10 |
| AP003355.2 | hsa-miR-326 | ST8SIA1 |
| AP003355.2 | hsa-miR-326 | RIC3 |
| AP003355.2 | hsa-miR-326 | ZNF208 |
| AP003355.2 | hsa-miR-326 | SRL |
| AP003355.2 | hsa-miR-326 | NFASC |
| AP003355.2 | hsa-miR-326 | DGKB |
| AP003355.2 | hsa-miR-326 | SYNC |
| AP003355.2 | hsa-miR-326 | LIMS2 |
| AP003355.2 | hsa-miR-326 | ITGA11 |
| AP003355.2 | hsa-miR-326 | SEPT4 |
| AP003355.2 | hsa-miR-326 | GFRA1 |
| AP003355.2 | hsa-miR-326 | MRGPRF |
| AP003355.2 | hsa-miR-326 | SHISA6 |
| AP003355.2 | hsa-miR-326 | TCEA3 |
| AP003355.2 | hsa-miR-326 | PLK5 |
| AP003355.2 | hsa-miR-326 | MUC15 |
| AP003355.2 | hsa-miR-326 | FAM107A |
| AP003355.2 | hsa-miR-326 | SLC15A2 |
| AP003355.2 | hsa-miR-326 | TMEM158 |
| AP003355.2 | hsa-miR-326 | SORCS2 |
| AP003355.2 | hsa-miR-326 | SLIT3 |
| AP003355.2 | hsa-miR-326 | PHEX |
| AP003355.2 | hsa-miR-326 | FHL5 |
| AP003355.2 | hsa-miR-326 | LDB3 |
| AP003355.2 | hsa-miR-326 | AHNAK2 |
| AP003355.2 | hsa-miR-326 | DET1 |
| AP003355.2 | hsa-miR-326 | AOC3 |
| AP003355.2 | hsa-miR-326 | PPP1R12B |
| DIO3OS | hsa-miR-326 | INMT |
| DIO3OS | hsa-miR-326 | TLL1 |
| DIO3OS | hsa-miR-326 | REEP1 |
| DIO3OS | hsa-miR-326 | CCDC80 |
| DIO3OS | hsa-miR-326 | HLF |
| DIO3OS | hsa-miR-326 | IGFBP5 |
| DIO3OS | hsa-miR-326 | SLC5A9 |
| DIO3OS | hsa-miR-326 | NRK |
| DIO3OS | hsa-miR-326 | PTGIS |
| DIO3OS | hsa-miR-326 | ATP1B2 |
| DIO3OS | hsa-miR-326 | CNN1 |
| DIO3OS | hsa-miR-326 | GATA5 |
| DIO3OS | hsa-miR-326 | RERG |
| DIO3OS | hsa-miR-326 | ITGA7 |
| DIO3OS | hsa-miR-326 | ABCC9 |
| DIO3OS | hsa-miR-326 | SELP |
| DIO3OS | hsa-miR-326 | NGEF |
| DIO3OS | hsa-miR-326 | PDE8B |
| DIO3OS | hsa-miR-326 | ASXL3 |
| DIO3OS | hsa-miR-326 | SLC4A3 |
| DIO3OS | hsa-miR-326 | SLC2A12 |
| DIO3OS | hsa-miR-326 | MYL9 |
| DIO3OS | hsa-miR-326 | RAB3C |
| DIO3OS | hsa-miR-326 | FBXO27 |
| DIO3OS | hsa-miR-326 | HFM1 |
| DIO3OS | hsa-miR-326 | KCNIP3 |
| DIO3OS | hsa-miR-326 | TCF23 |
| DIO3OS | hsa-miR-326 | MASP1 |
| DIO3OS | hsa-miR-326 | HPGD |
| DIO3OS | hsa-miR-326 | FAT3 |
| DIO3OS | hsa-miR-326 | TMEM132B |
| DIO3OS | hsa-miR-326 | HSPB3 |
| DIO3OS | hsa-miR-326 | MYRIP |
| DIO3OS | hsa-miR-326 | KCNK3 |
| DIO3OS | hsa-miR-326 | TUB |
| DIO3OS | hsa-miR-326 | LPP |
| DIO3OS | hsa-miR-326 | C7 |
| DIO3OS | hsa-miR-326 | EBF1 |
| DIO3OS | hsa-miR-326 | BVES |
| DIO3OS | hsa-miR-326 | GPR88 |
| DIO3OS | hsa-miR-326 | CLU |
| DIO3OS | hsa-miR-326 | SLC2A4 |
| DIO3OS | hsa-miR-326 | FIBIN |
| DIO3OS | hsa-miR-326 | SYT9 |
| DIO3OS | hsa-miR-326 | KIAA1549L |
| DIO3OS | hsa-miR-326 | GDNF |
| DIO3OS | hsa-miR-326 | TNFAIP8L3 |
| DIO3OS | hsa-miR-326 | GRID1 |
| DIO3OS | hsa-miR-326 | ST6GALNAC3 |
| DIO3OS | hsa-miR-326 | COL4A6 |
| DIO3OS | hsa-miR-326 | TSPAN18 |
| DIO3OS | hsa-miR-326 | PRELP |
| DIO3OS | hsa-miR-326 | FLNC |
| DIO3OS | hsa-miR-326 | CAMK2A |
| DIO3OS | hsa-miR-326 | SNCG |
| DIO3OS | hsa-miR-326 | ATP2B2 |
| DIO3OS | hsa-miR-326 | SMOC2 |
| DIO3OS | hsa-miR-326 | ADAM33 |
| DIO3OS | hsa-miR-326 | SUSD2 |
| DIO3OS | hsa-miR-326 | CACNA1H |
| DIO3OS | hsa-miR-326 | NFIX |
| DIO3OS | hsa-miR-326 | MYLK |
| DIO3OS | hsa-miR-326 | LMOD1 |
| DIO3OS | hsa-miR-326 | DNM3 |
| DIO3OS | hsa-miR-326 | GPR161 |
| DIO3OS | hsa-miR-326 | PBX1 |
| DIO3OS | hsa-miR-326 | RBM20 |
| DIO3OS | hsa-miR-326 | SYPL2 |
| DIO3OS | hsa-miR-326 | LDOC1 |
| DIO3OS | hsa-miR-326 | CDR1 |
| DIO3OS | hsa-miR-326 | BEND6 |
| DIO3OS | hsa-miR-326 | PTGFR |
| DIO3OS | hsa-miR-326 | APCDD1L |
| DIO3OS | hsa-miR-326 | KCNB1 |
| DIO3OS | hsa-miR-326 | CHRDL1 |
| DIO3OS | hsa-miR-326 | HYI |
| DIO3OS | hsa-miR-326 | WISP2 |
| DIO3OS | hsa-miR-326 | JPH2 |
| DIO3OS | hsa-miR-326 | DES |
| DIO3OS | hsa-miR-326 | PRKG1 |
| DIO3OS | hsa-miR-326 | TRPM3 |
| DIO3OS | hsa-miR-326 | GPR20 |
| DIO3OS | hsa-miR-326 | PKHD1L1 |
| DIO3OS | hsa-miR-326 | CCDC3 |
| DIO3OS | hsa-miR-326 | PTCHD1 |
| DIO3OS | hsa-miR-326 | ADRA1D |
| DIO3OS | hsa-miR-326 | DCLK1 |
| DIO3OS | hsa-miR-326 | BNC2 |
| DIO3OS | hsa-miR-326 | SMOC1 |
| DIO3OS | hsa-miR-326 | CYS1 |
| DIO3OS | hsa-miR-326 | LGI2 |
| DIO3OS | hsa-miR-326 | SEMA5A |
| DIO3OS | hsa-miR-326 | CDON |
| DIO3OS | hsa-miR-326 | PRIMA1 |
| DIO3OS | hsa-miR-326 | NOX4 |
| DIO3OS | hsa-miR-326 | SPOCK1 |
| DIO3OS | hsa-miR-326 | FAM13A |
| DIO3OS | hsa-miR-326 | MAPK10 |
| DIO3OS | hsa-miR-326 | ST8SIA1 |
| DIO3OS | hsa-miR-326 | RIC3 |
| DIO3OS | hsa-miR-326 | ZNF208 |
| DIO3OS | hsa-miR-326 | SRL |
| DIO3OS | hsa-miR-326 | NFASC |
| DIO3OS | hsa-miR-326 | DGKB |
| DIO3OS | hsa-miR-326 | SYNC |
| DIO3OS | hsa-miR-326 | LIMS2 |
| DIO3OS | hsa-miR-326 | ITGA11 |
| DIO3OS | hsa-miR-326 | SEPT4 |
| DIO3OS | hsa-miR-326 | GFRA1 |
| DIO3OS | hsa-miR-326 | MRGPRF |
| DIO3OS | hsa-miR-326 | SHISA6 |
| DIO3OS | hsa-miR-326 | TCEA3 |
| DIO3OS | hsa-miR-326 | PLK5 |
| DIO3OS | hsa-miR-326 | MUC15 |
| DIO3OS | hsa-miR-326 | FAM107A |
| DIO3OS | hsa-miR-326 | SLC15A2 |
| DIO3OS | hsa-miR-326 | TMEM158 |
| DIO3OS | hsa-miR-326 | SORCS2 |
| DIO3OS | hsa-miR-326 | SLIT3 |
| DIO3OS | hsa-miR-326 | PHEX |
| DIO3OS | hsa-miR-326 | FHL5 |
| DIO3OS | hsa-miR-326 | LDB3 |
| DIO3OS | hsa-miR-326 | AHNAK2 |
| DIO3OS | hsa-miR-326 | DET1 |
| DIO3OS | hsa-miR-326 | AOC3 |
| DIO3OS | hsa-miR-326 | PPP1R12B |
